# Supplementary material for: Chiral acid-catalysed enantioselective C−H functionalization of toluene and its derivatives driven by visible light
Source: Nat Commun. 2019 Apr 16;10:1774. doi: 10.1038/s41467-019-09857-9 (PMC6467922; doi:10.1038/s41467-019-09857-9)
Supplement: Supplementary file 1 — Supplementary Information [file 41467_2019_9857_MOESM1_ESM.pdf]

Supporting Information

Chiral acid-catalysed enantioselective C−H functionalization of toluene and its derivatives driven by visible light

Li et al.

Supplementary Figures

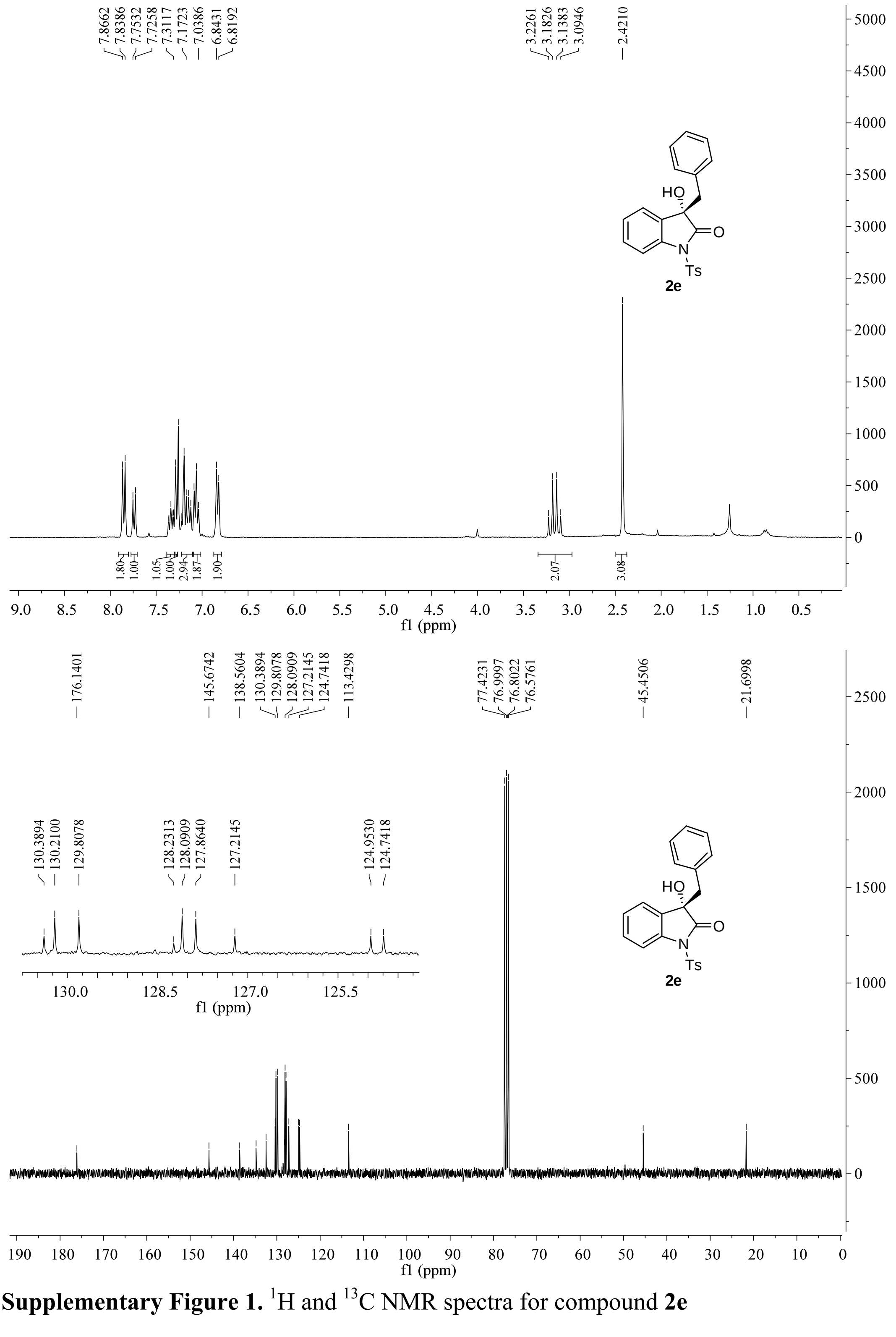

Supplementary Figure 1. ^{1}H and ^{13}C NMR spectra for compound **2e**

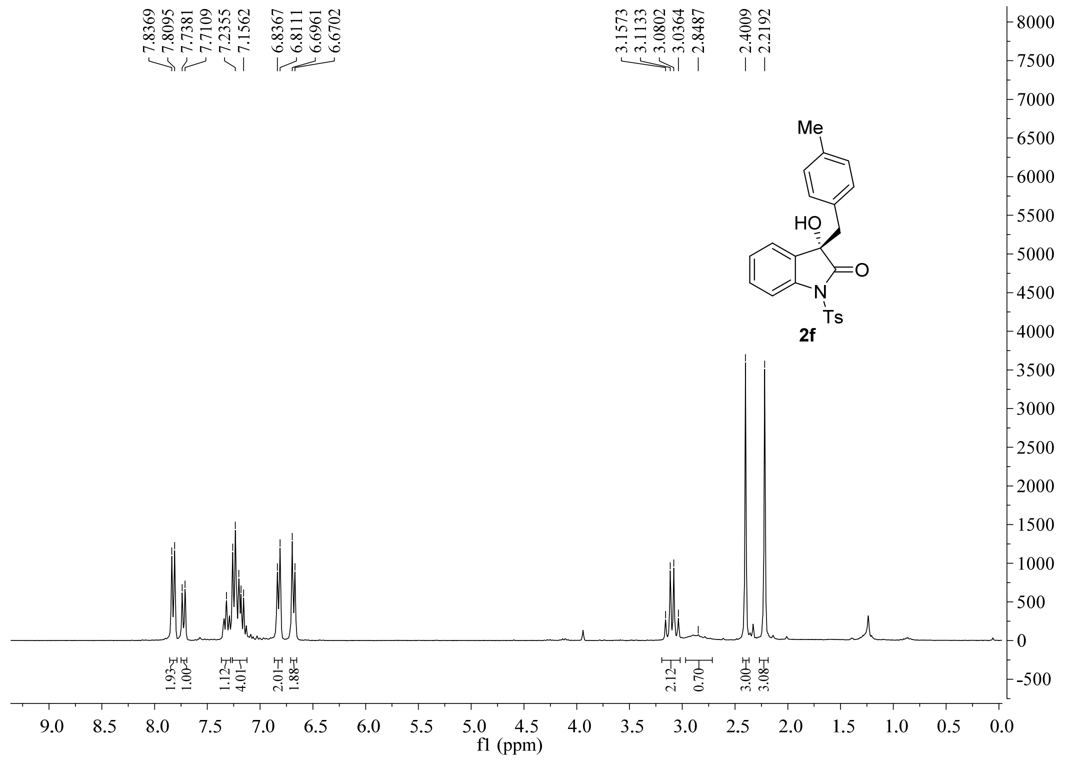

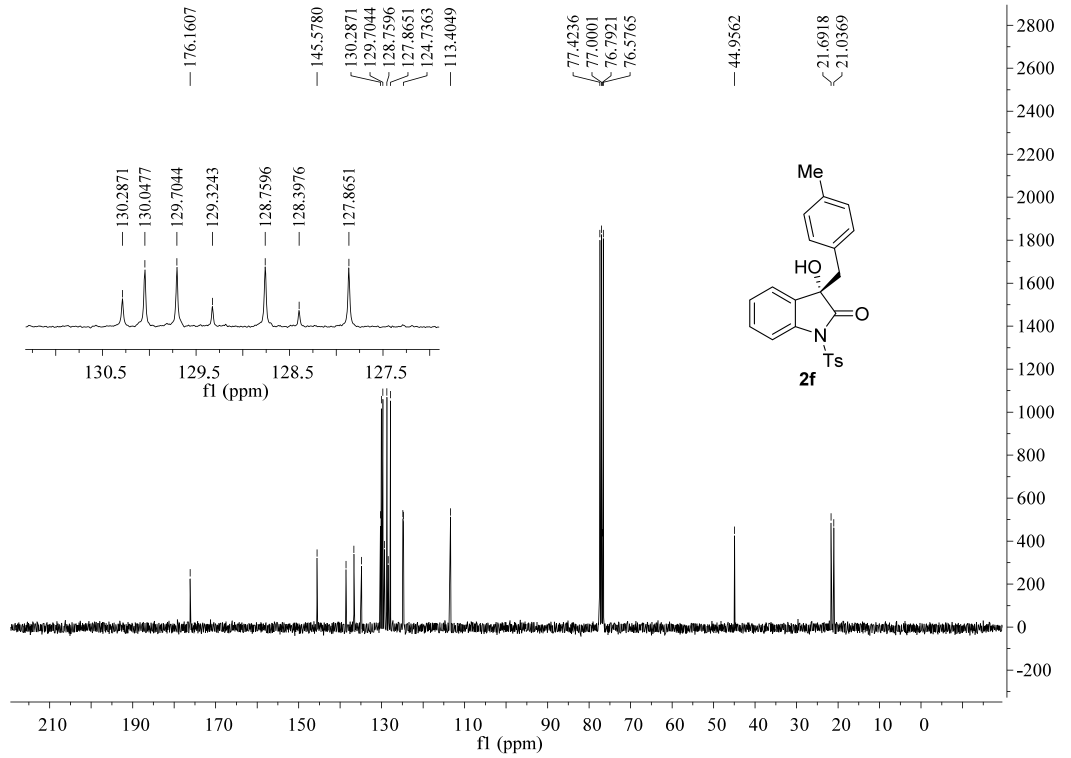

Supplementary Figure 2. ^{1}H and ^{13}C NMR spectra for compound **2f**

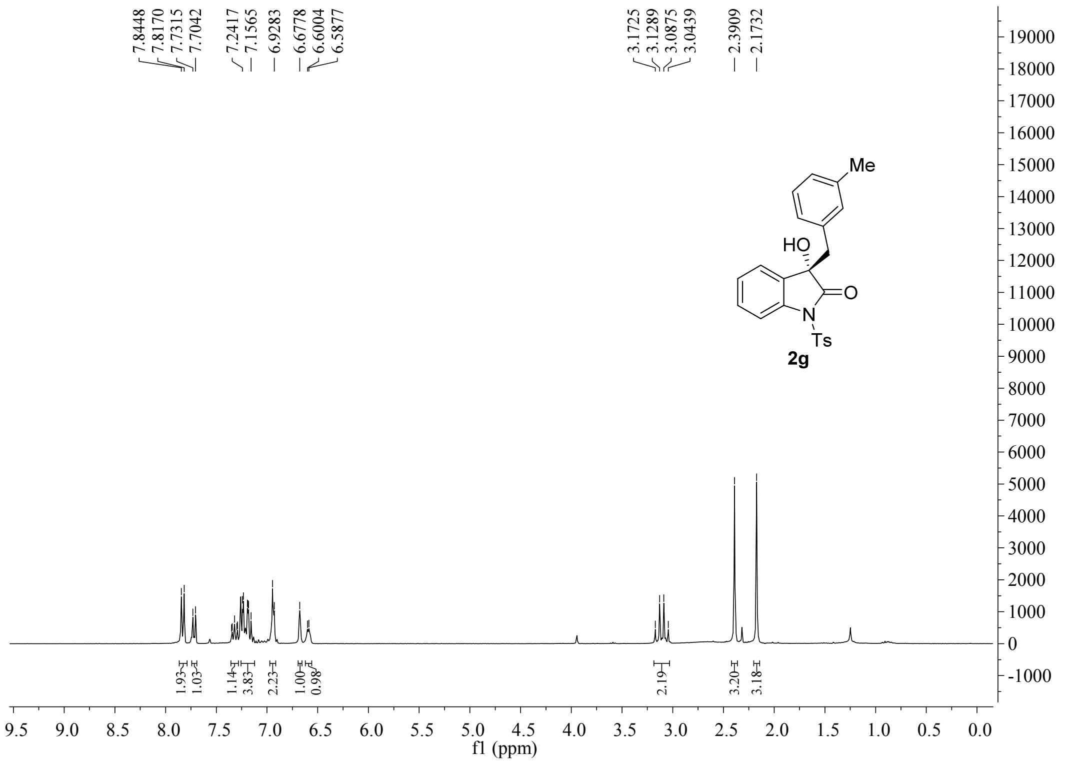

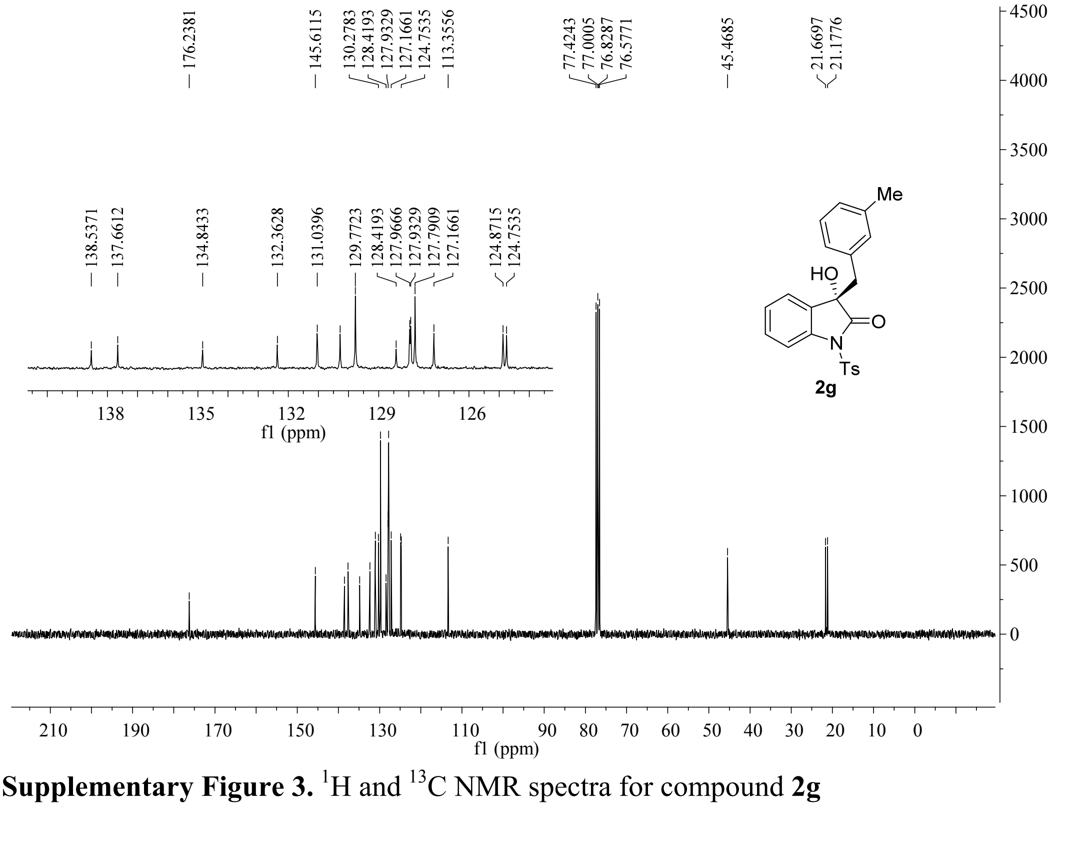

Supplementary Figure 3. ^{1}H and ^{13}C NMR spectra for compound **2g**

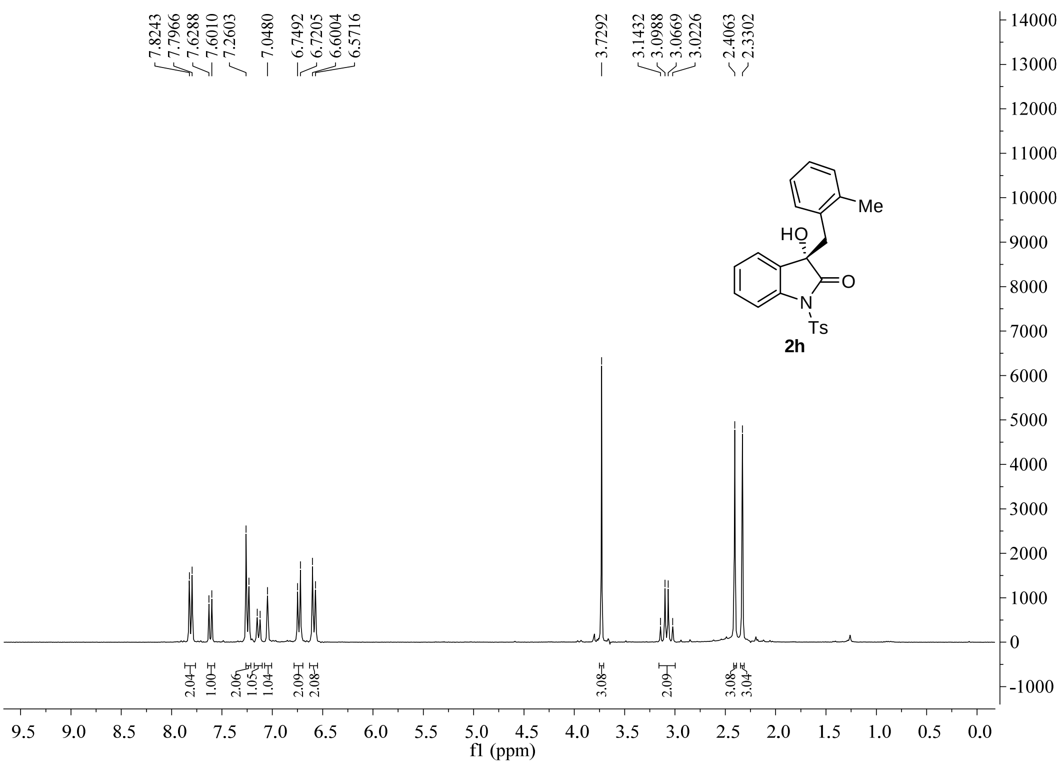

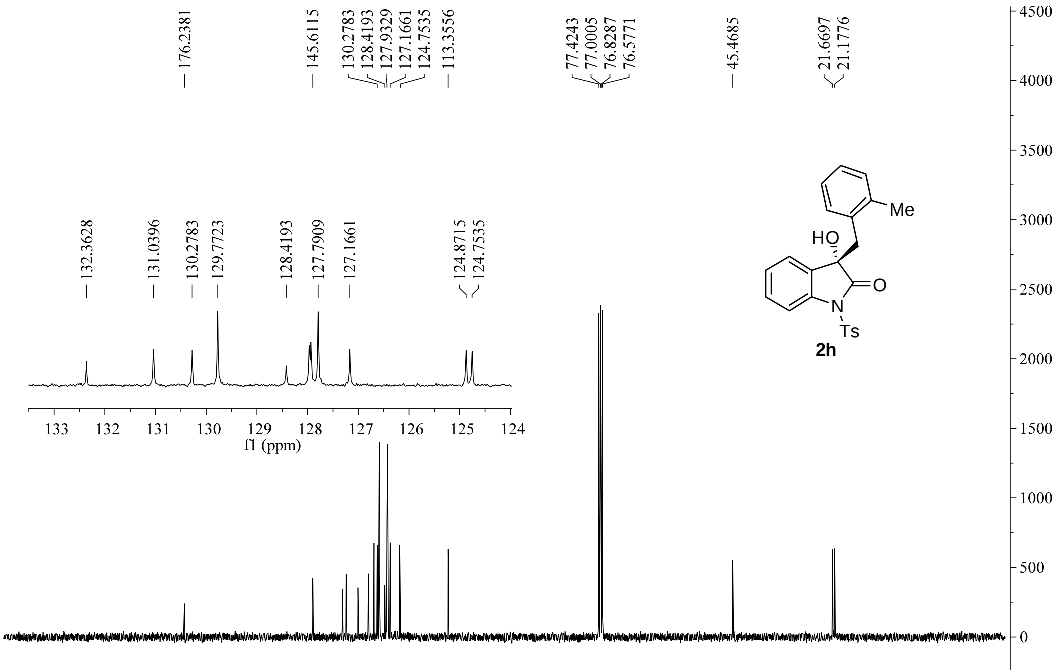

Supplementary Figure 4. ^{1}H and ^{13}C NMR spectra for compound **2h**

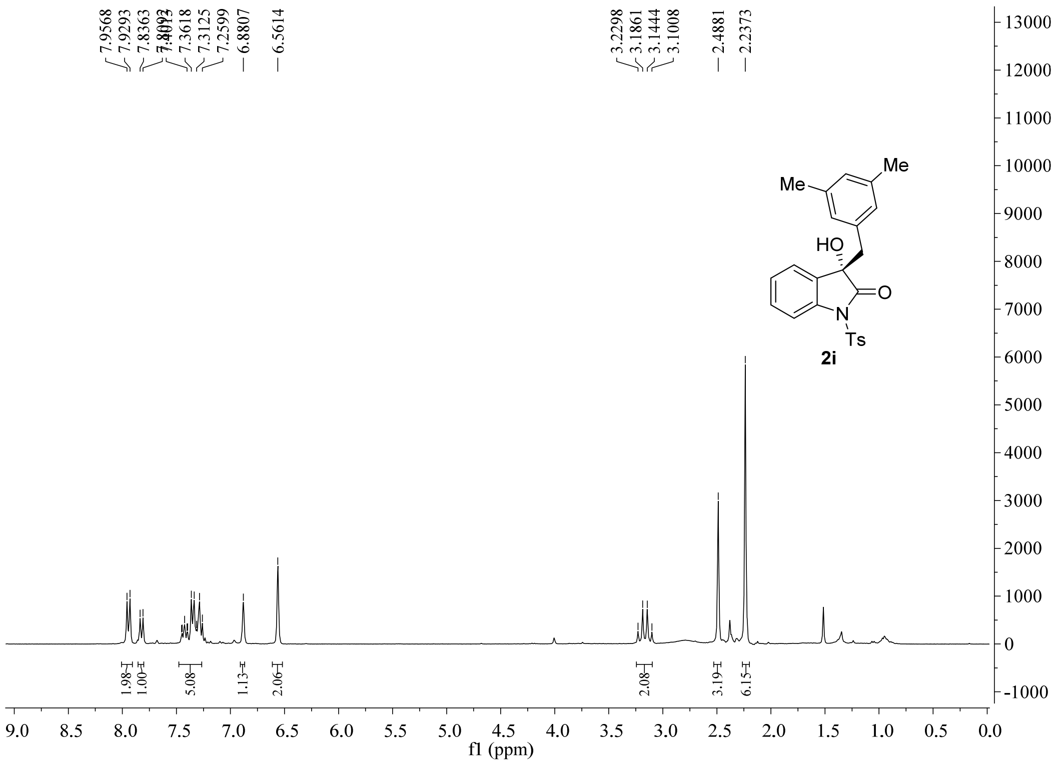

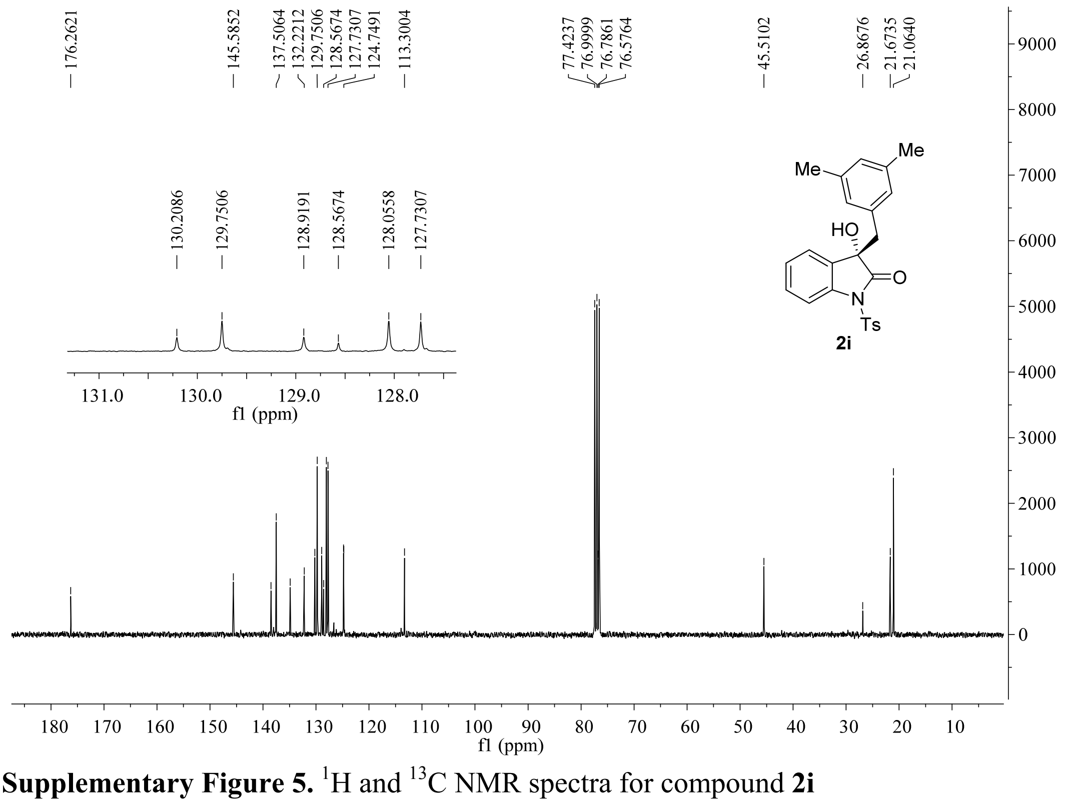

Supplementary Figure 5. ^{1}H and ^{13}C NMR spectra for compound **2i**

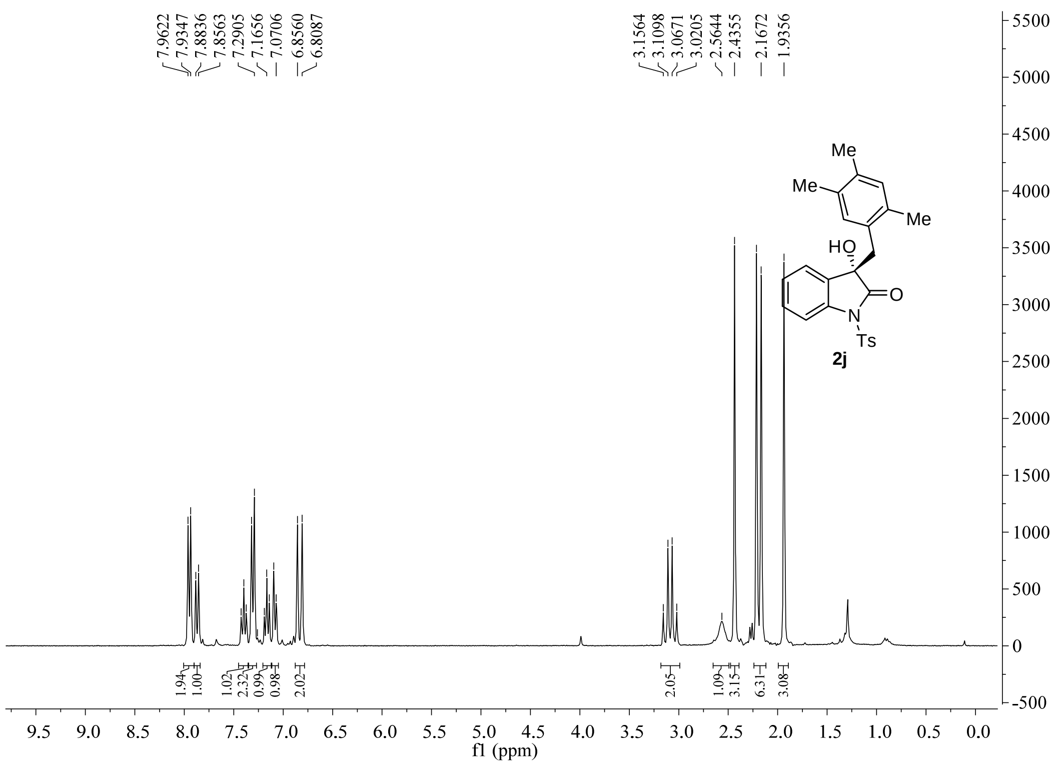

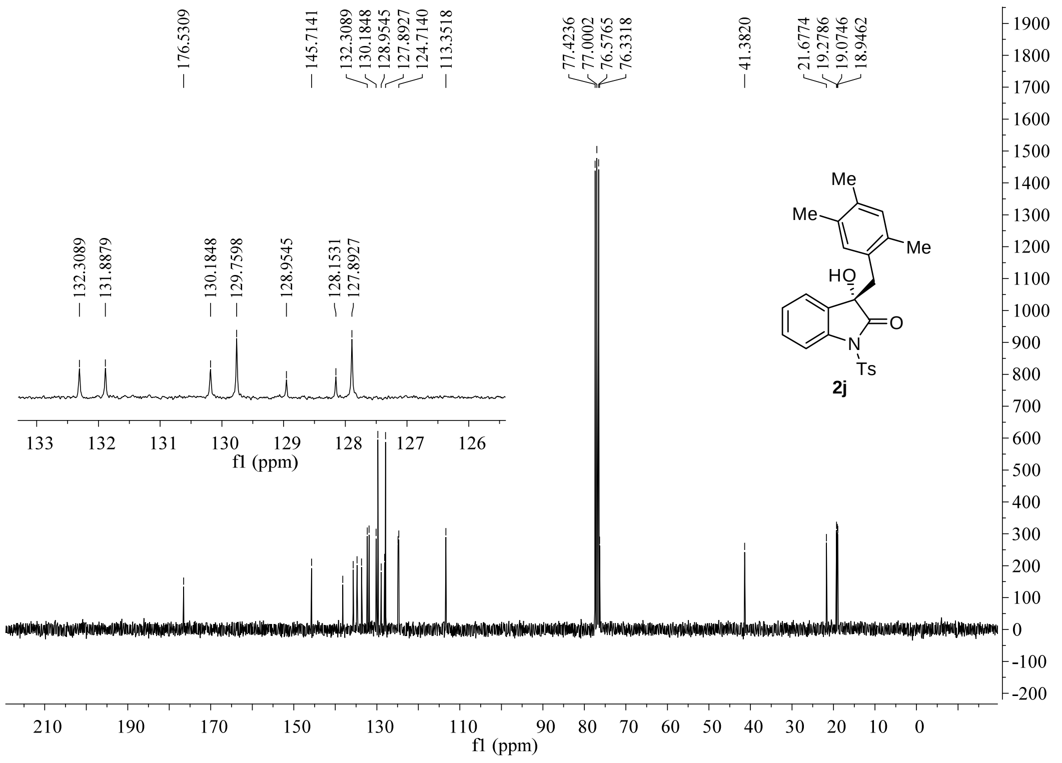

Supplementary Figure 6. ^{1}H and ^{13}C NMR spectra for compound **2j**

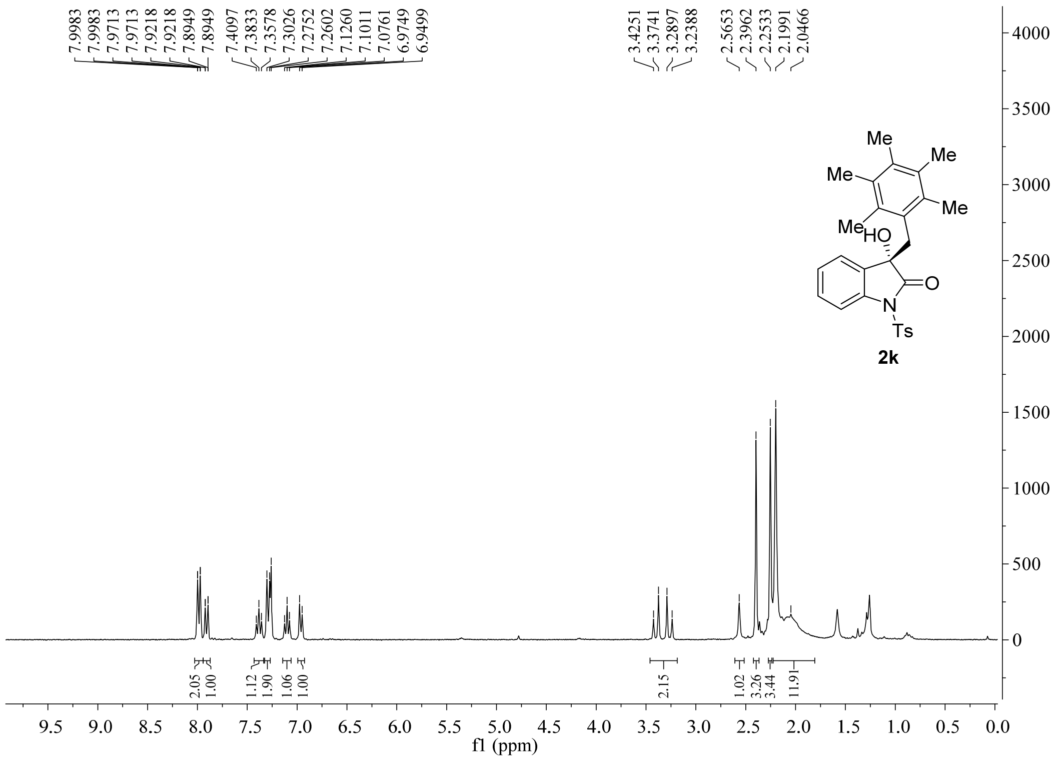

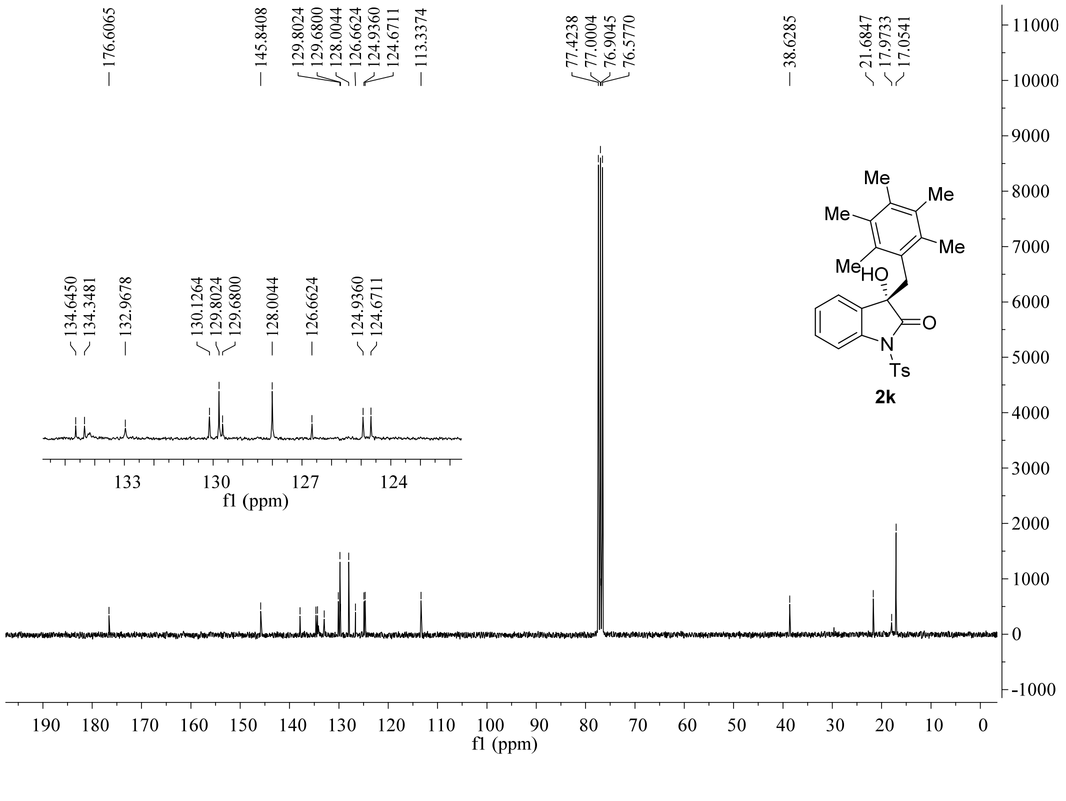

Supplementary Figure 7. ^{1}H and ^{13}C NMR spectra for compound **2k**

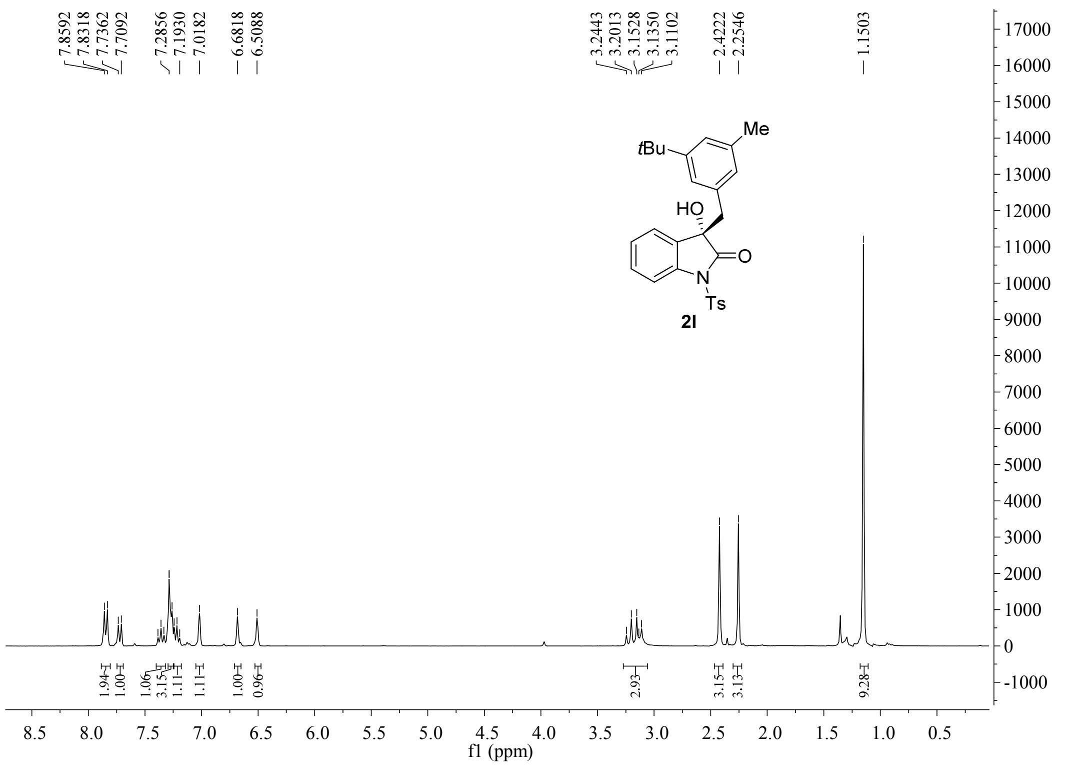

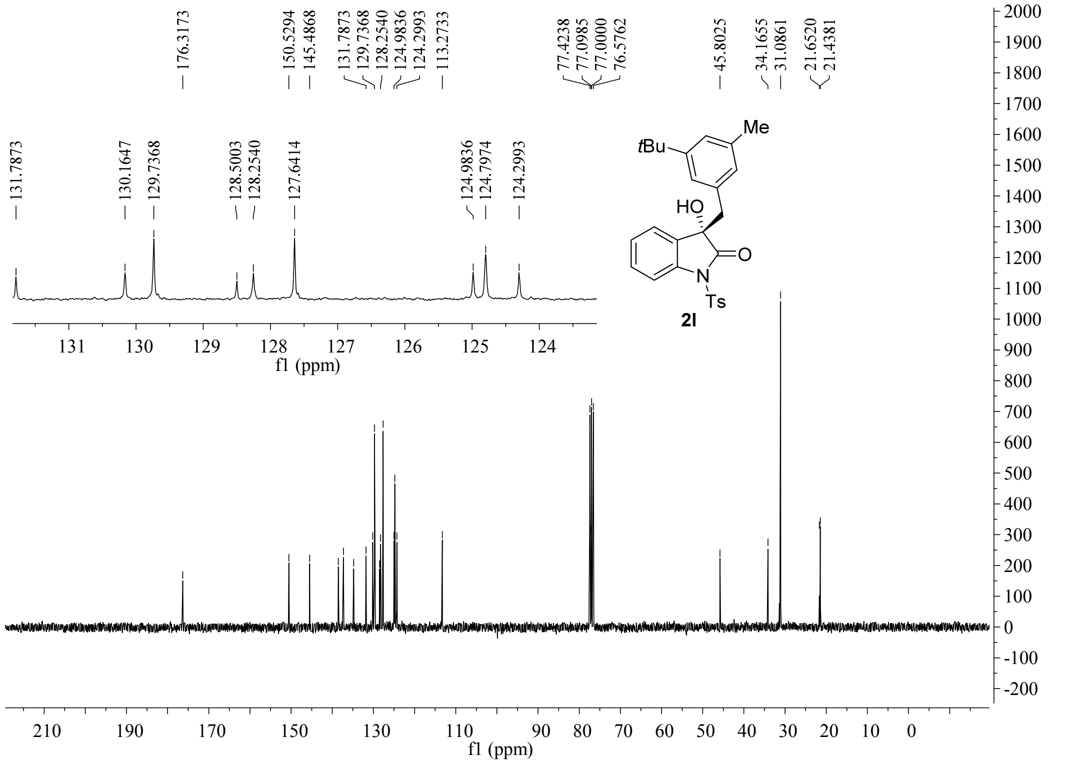

Supplementary Figure 8. ^{1}H and ^{13}C NMR spectra for compound **2l**

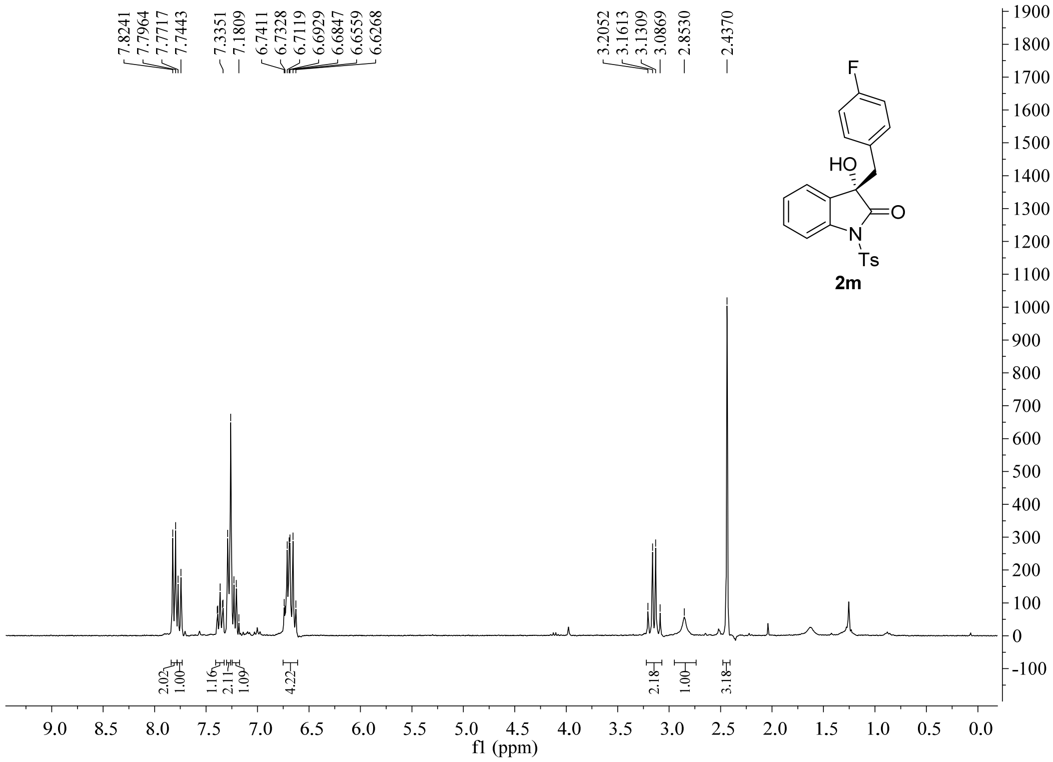

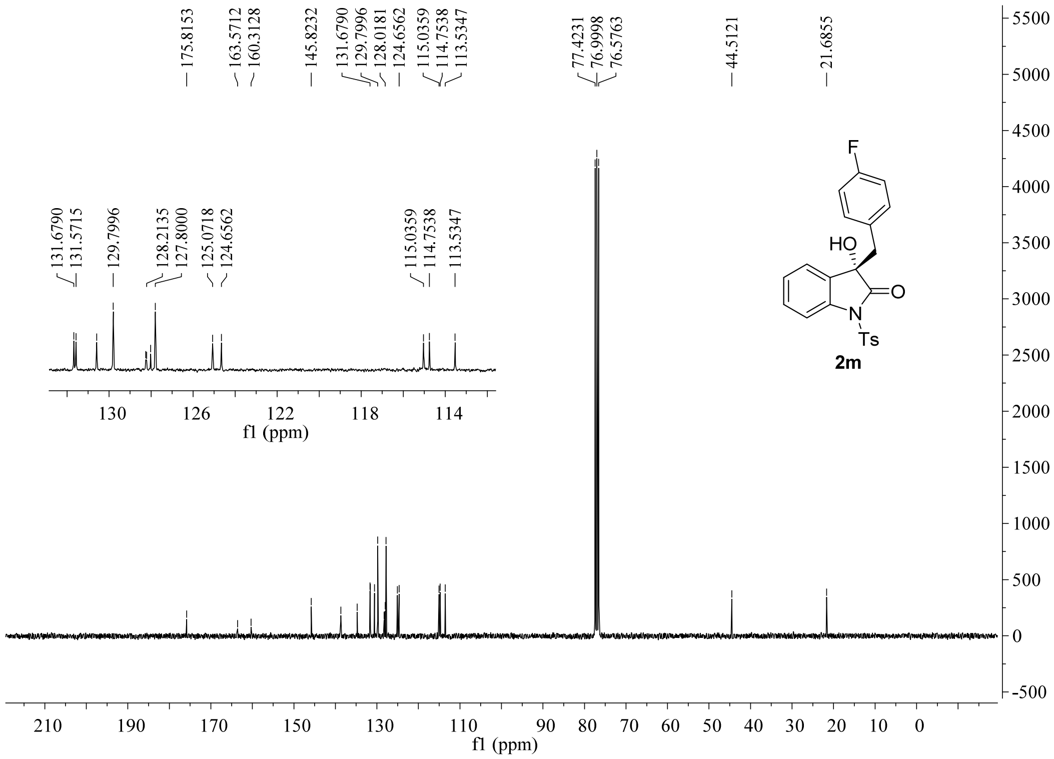

Supplementary Figure 9. ^{1}H and ^{13}C NMR spectra for compound **2m**

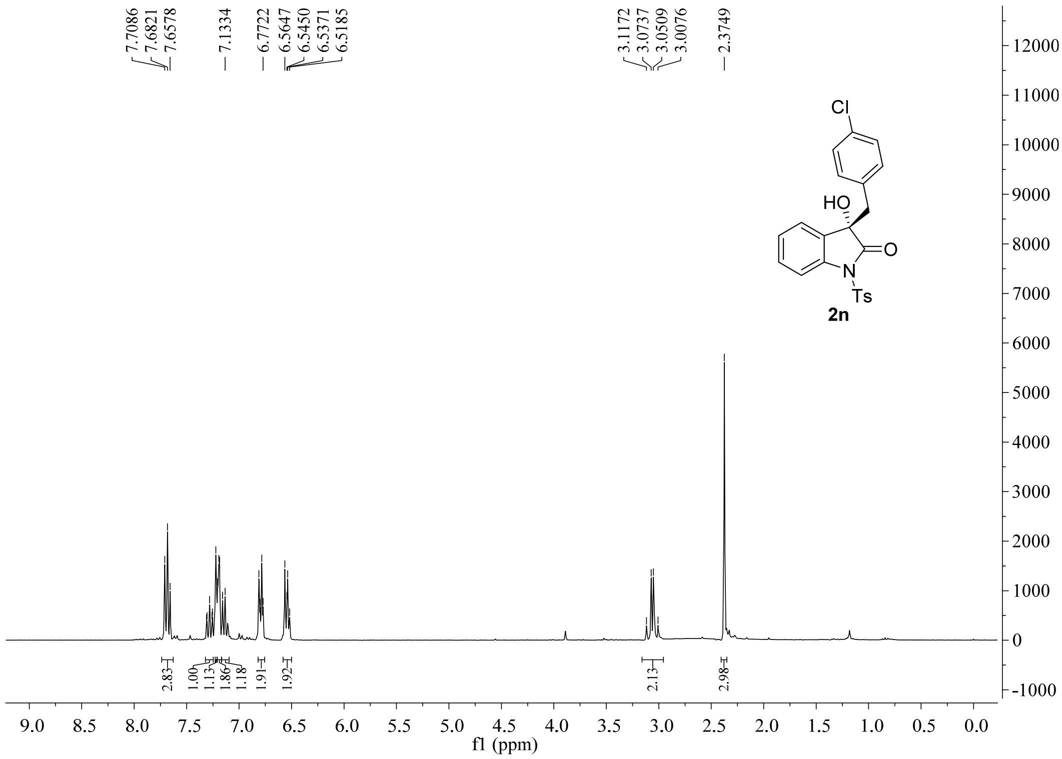

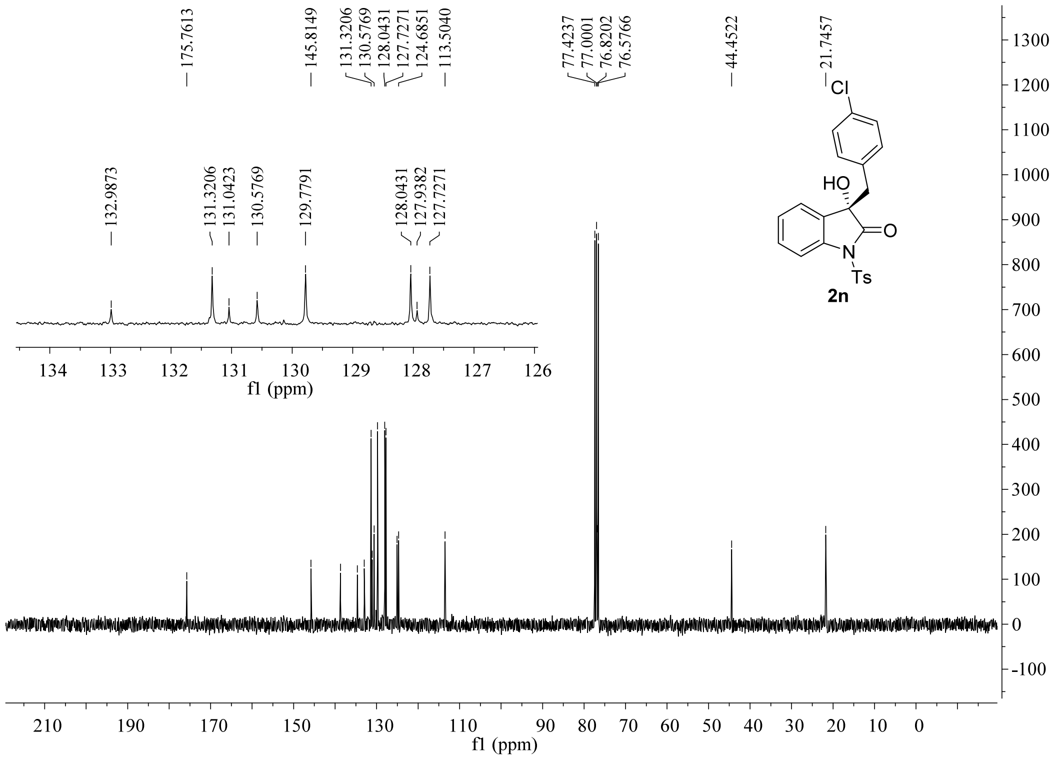

Supplementary Figure 10. ^{1}H and ^{13}C NMR spectra for compound **2n**

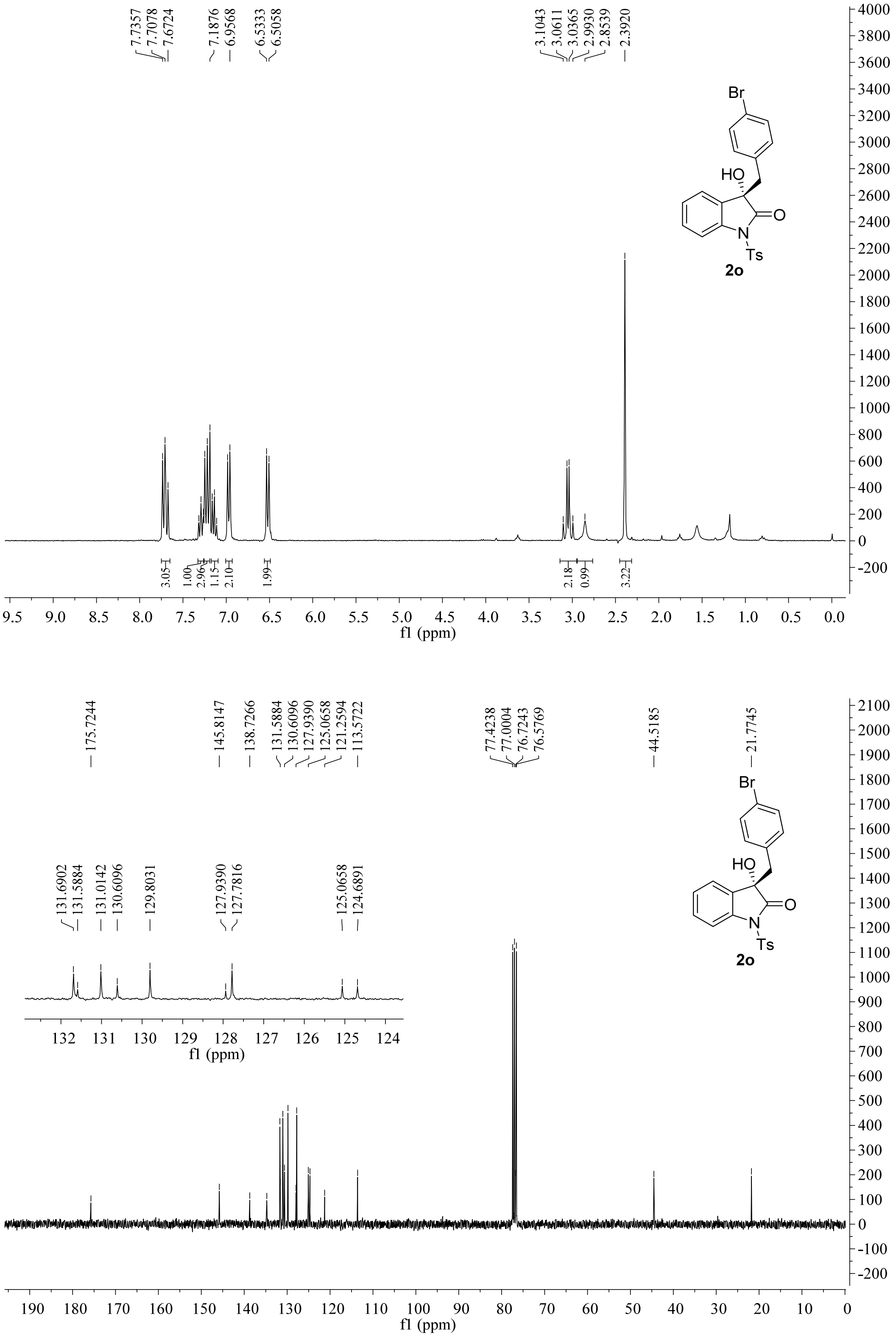

Supplementary Figure 11. ^{1}H and ^{13}C NMR spectra for compound **2o**

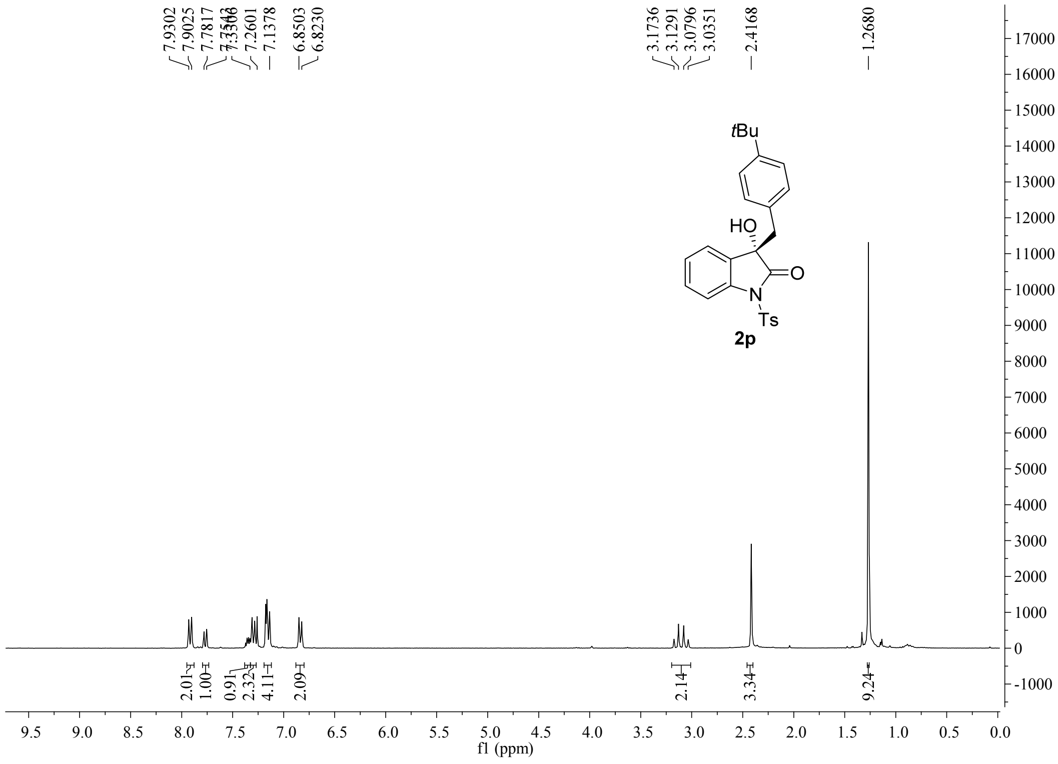

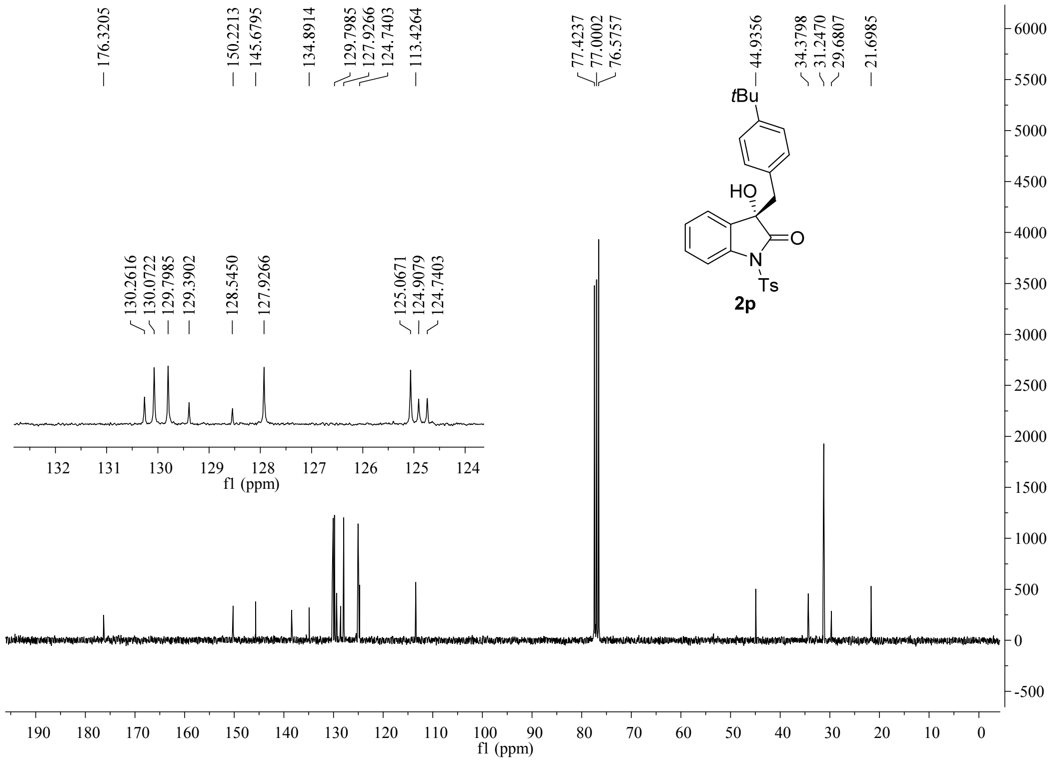

Supplementary Figure 12. ^{1}H and ^{13}C NMR spectra for compound **2p**

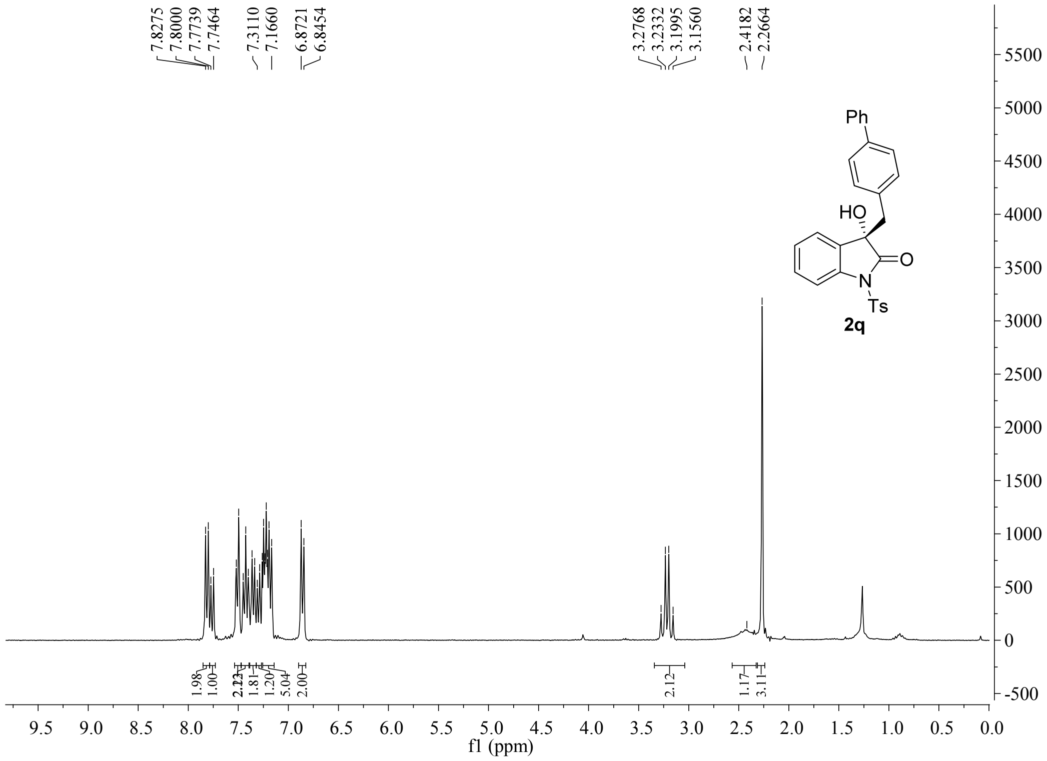

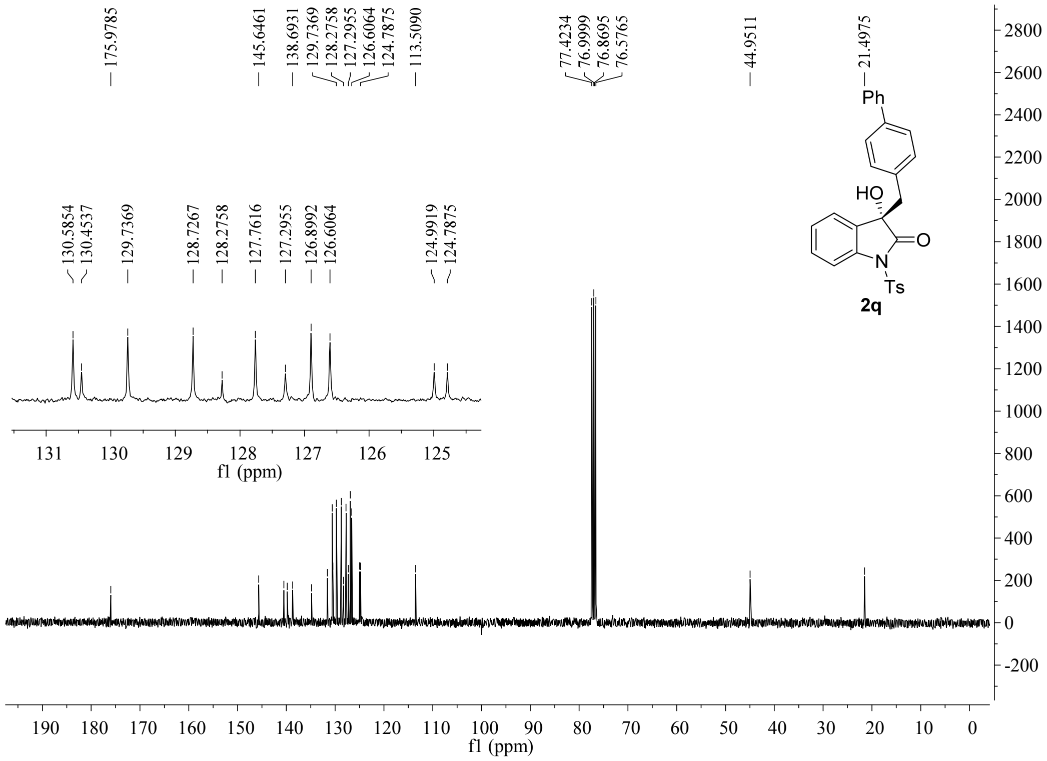

Supplementary Figure 13. ^{1}H and ^{13}C NMR spectra for compound **2q**

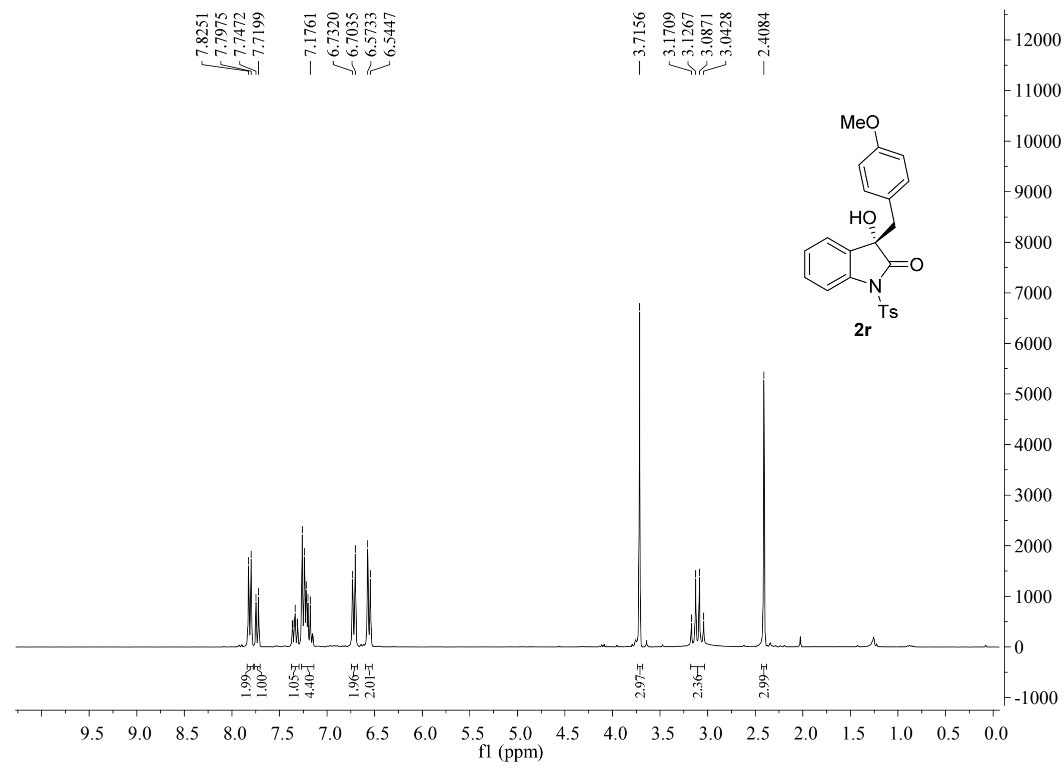

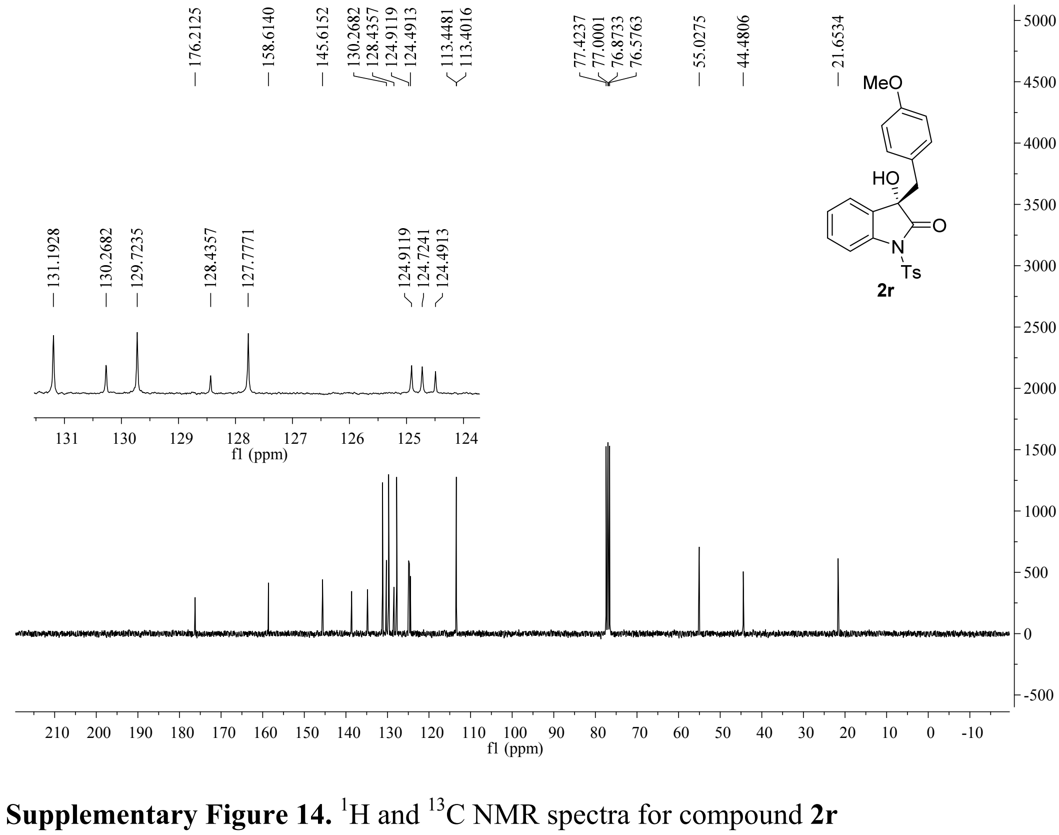

Supplementary Figure 14. ^{1}H and ^{13}C NMR spectra for compound **2r**

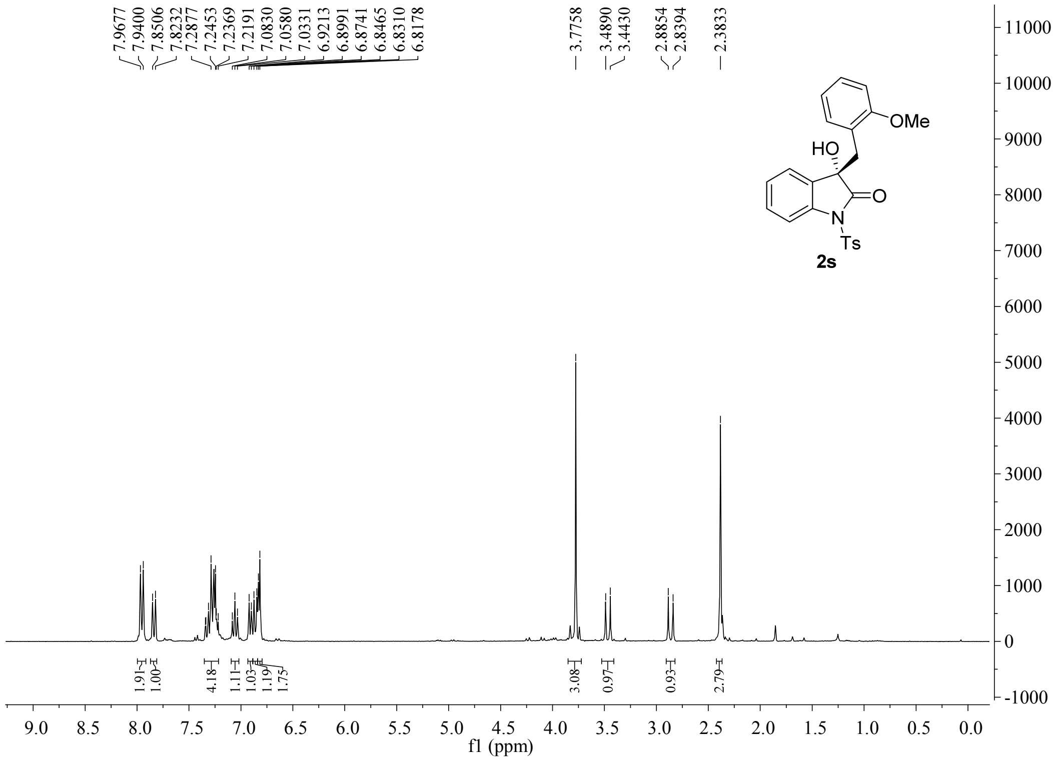

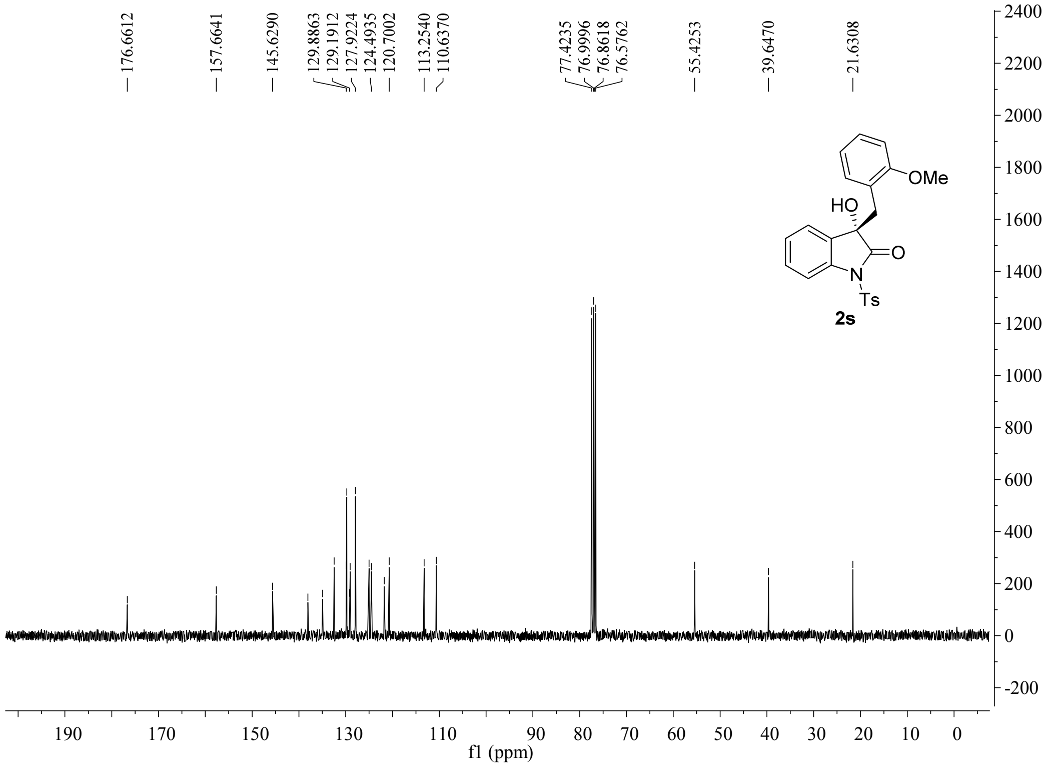

Supplementary Figure 15. ^{1}H and ^{13}C NMR spectra for compound **2s**

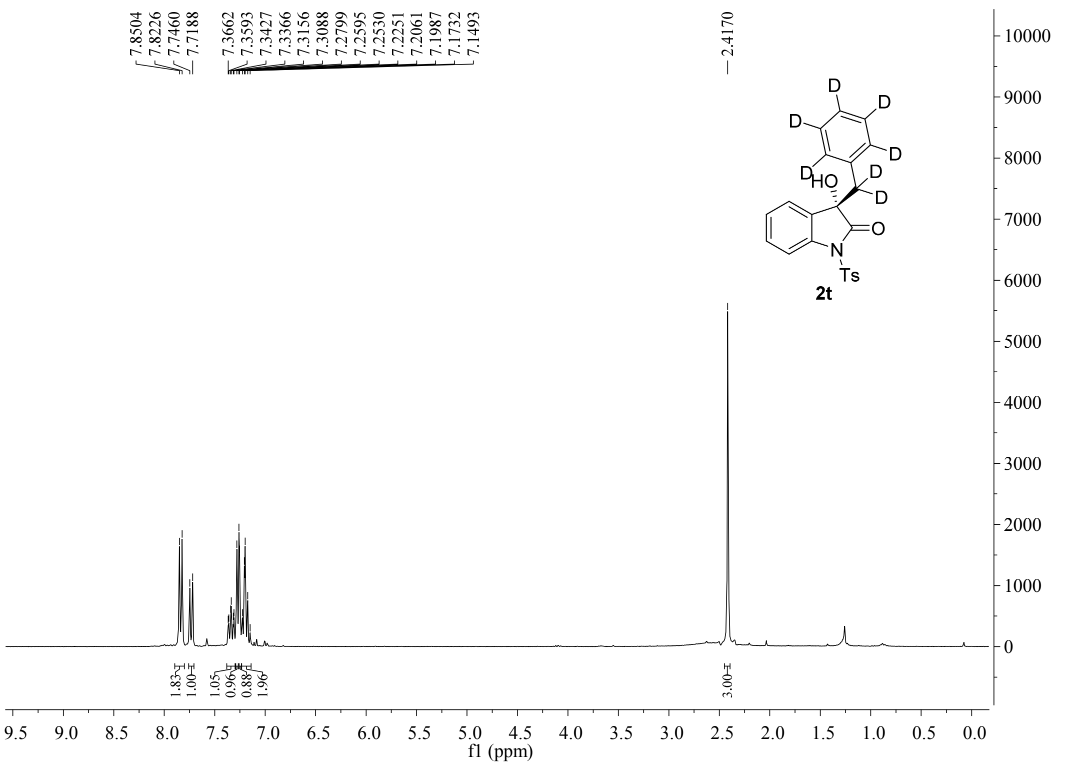

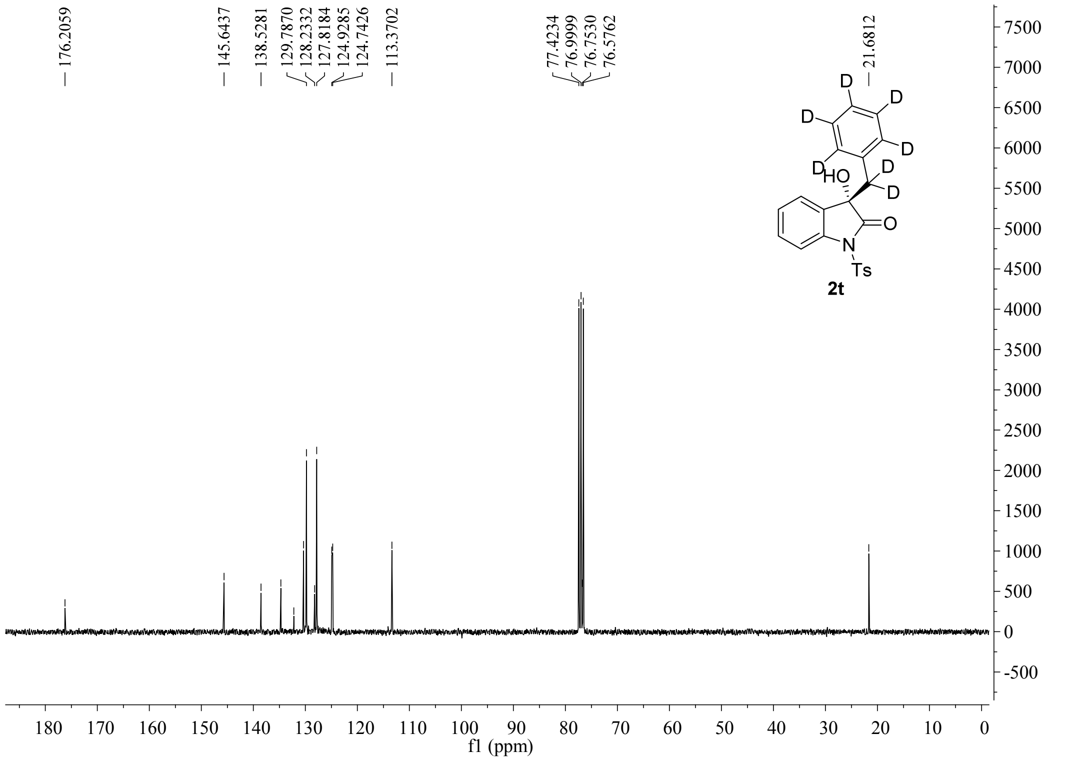

Supplementary Figure 16. ^{1}H and ^{13}C NMR spectra for compound **2t**

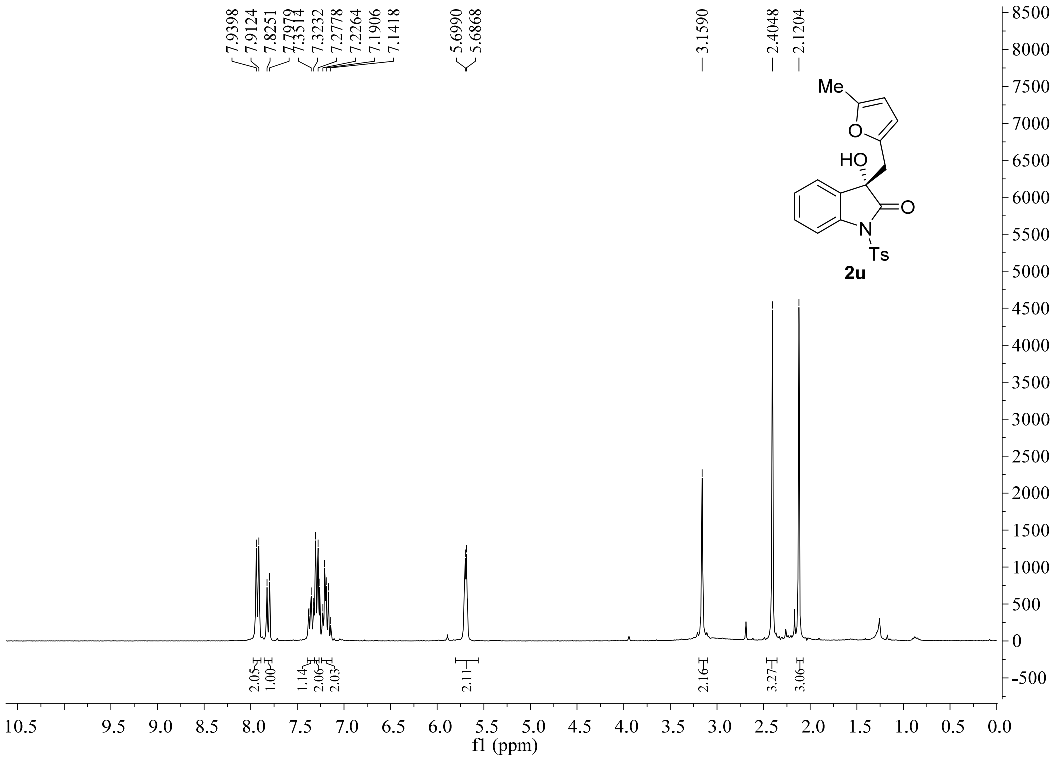

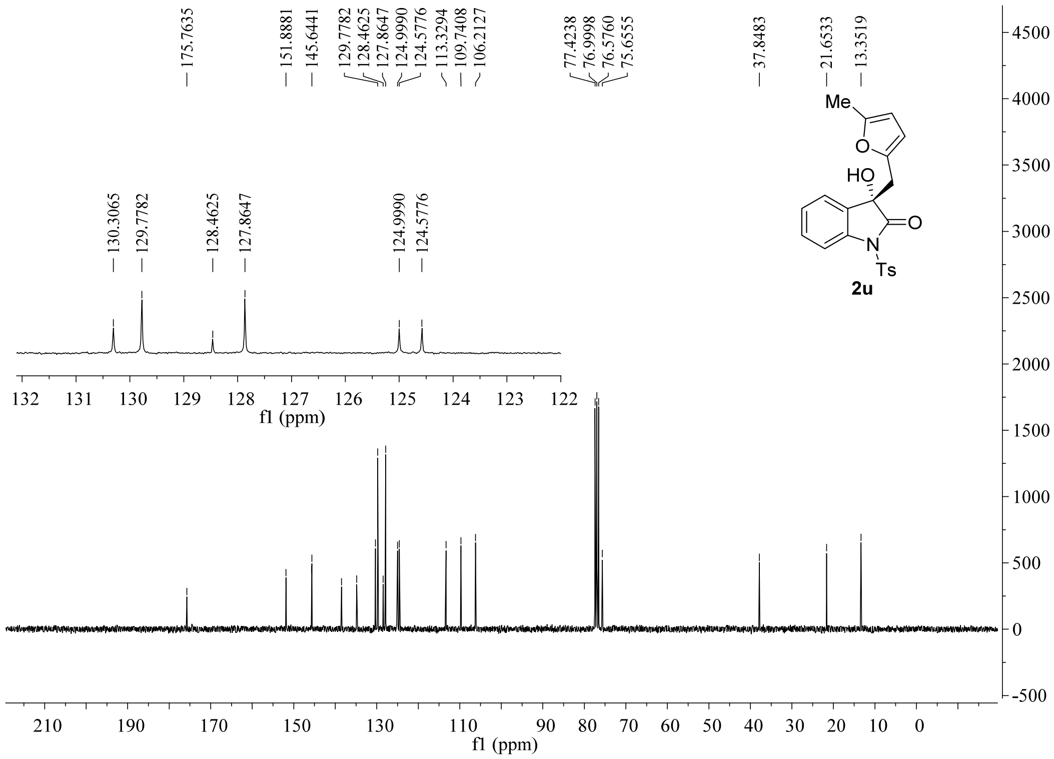

Supplementary Figure 17. ^{1}H and ^{13}C NMR spectra for compound **2u**

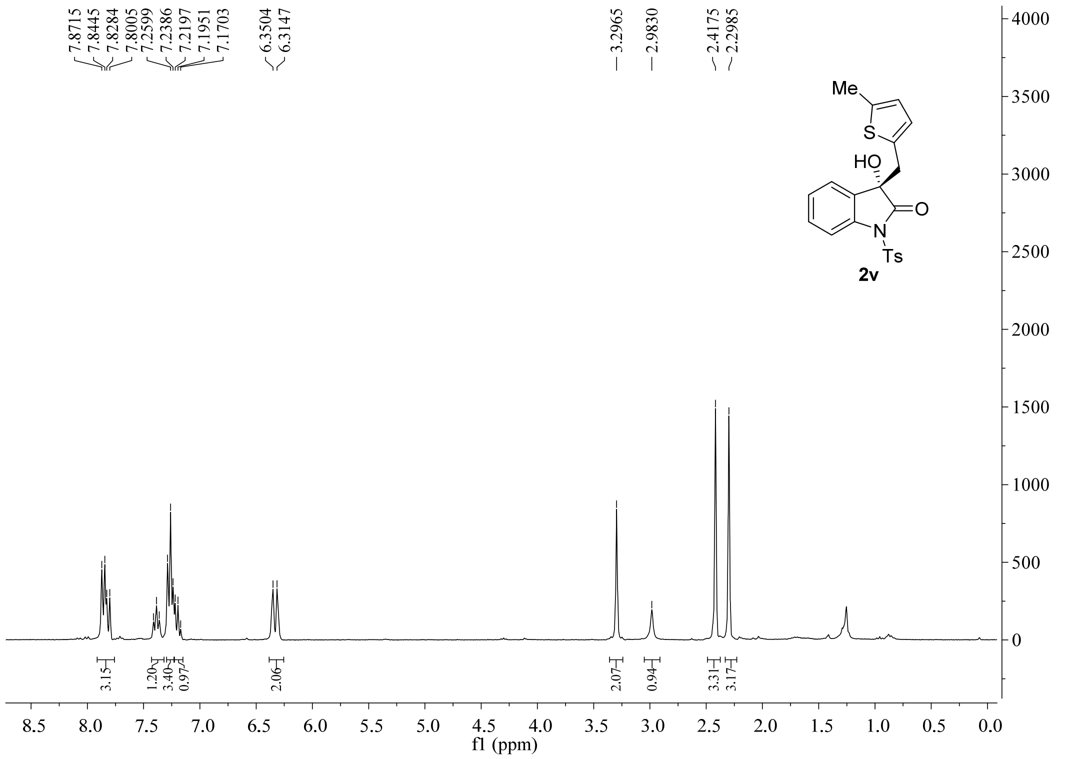

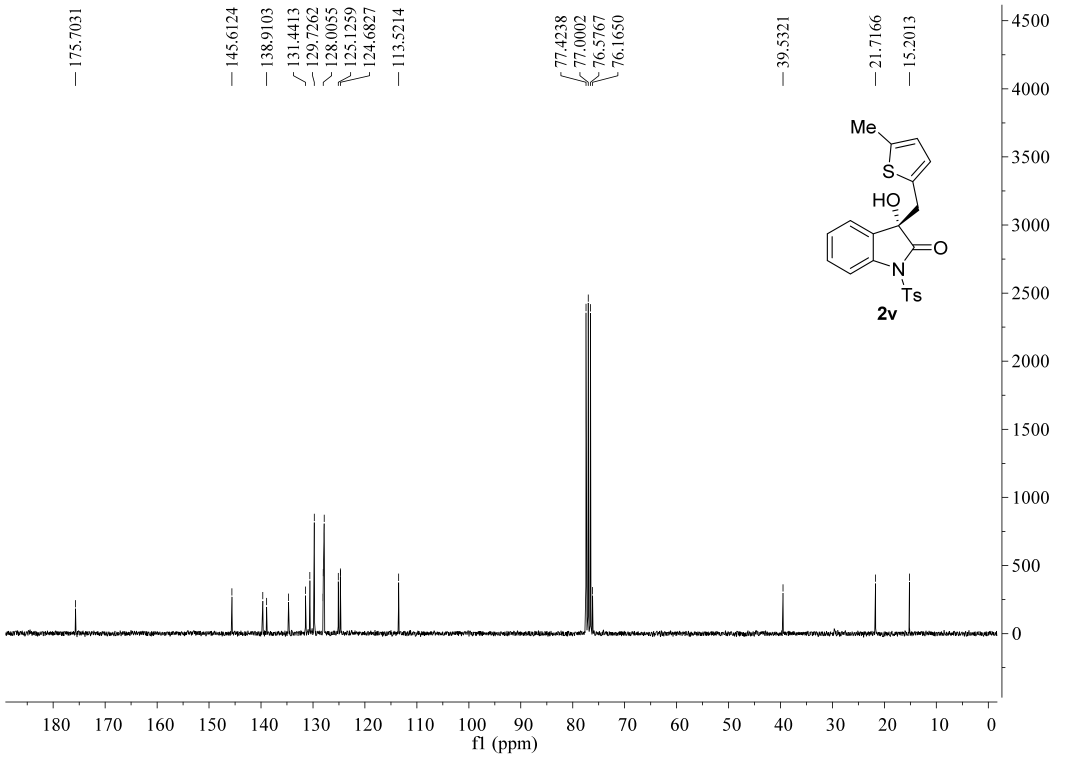

Supplementary Figure 18. ^{1}H and ^{13}C NMR spectra for compound **2v**

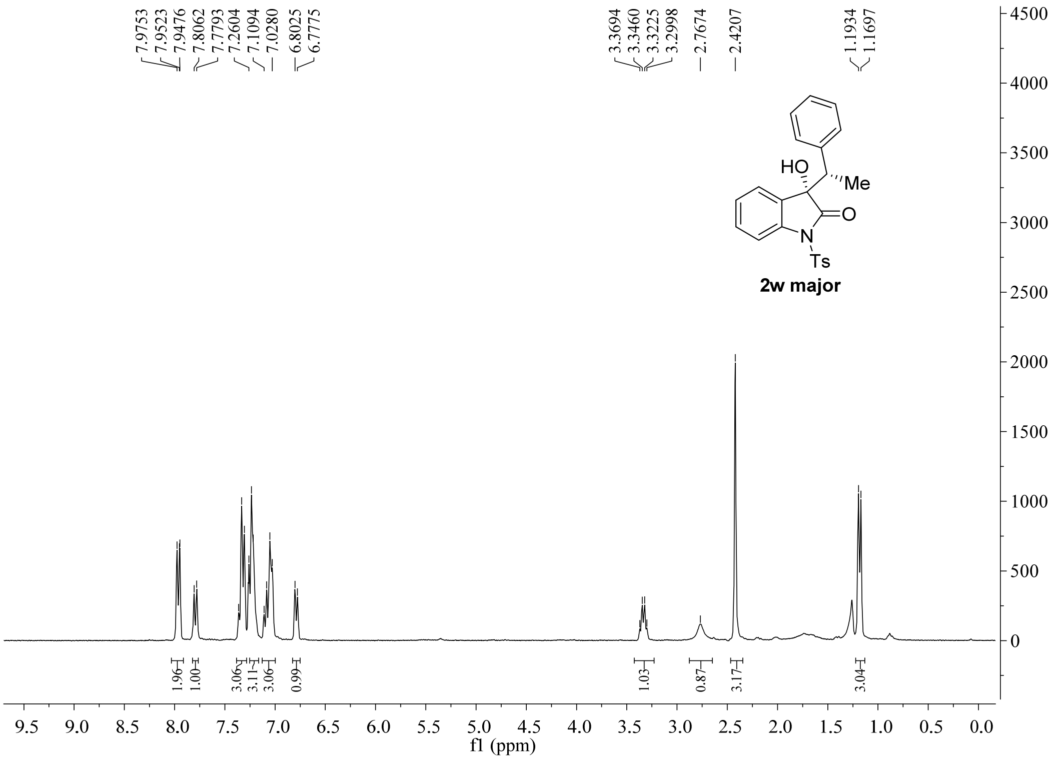

7.9753
7.9523
7.9476
7.8062
7.7793
7.2604
7.1094
7.0280
6.8025
6.7775
3.3694
3.3460
3.3225
3.2998
2.7674
2.4207
1.1934
1.1697
HO
Me
O
N
Ts
2w major
1.96
1.00
3.06
3.11
3.06
0.99
1.03
0.87
3.17
3.04
f1 (ppm)

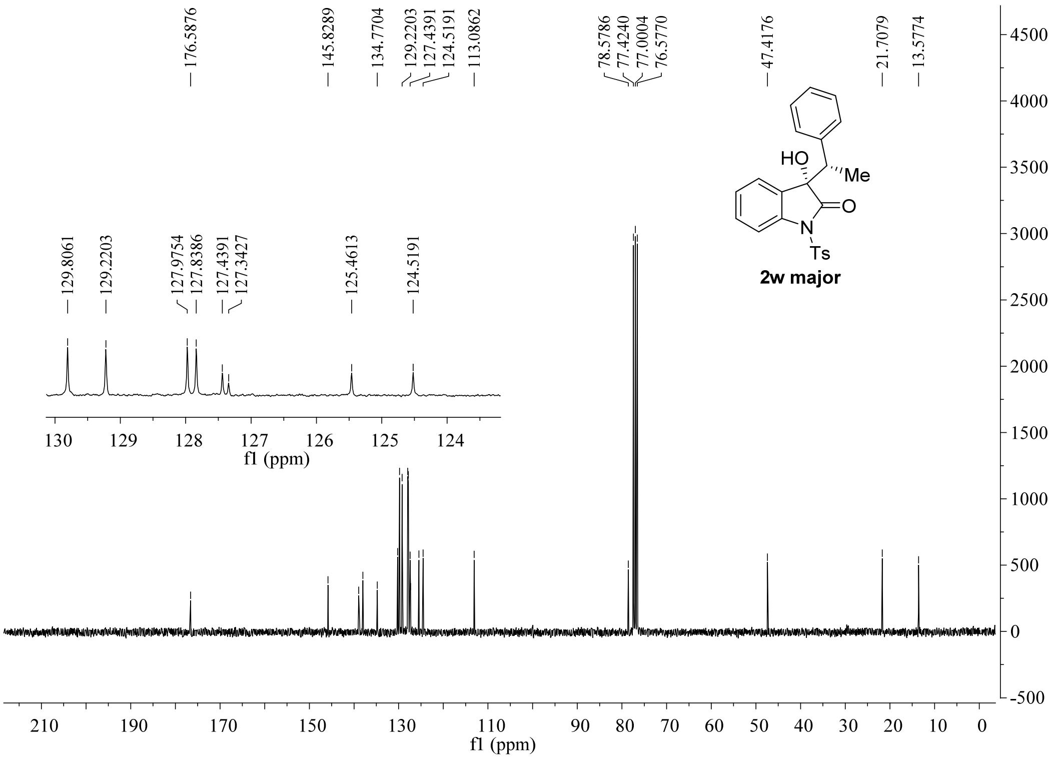

176.5876
145.8289
134.7704
129.2203
127.4391
124.5191
113.0862
78.5786
77.4240
77.0004
76.5770
47.4176
21.7079
13.5774
129.8061
129.2203
127.9754
127.8386
127.4391
127.3427
125.4613
124.5191
HO
Me
O
N
Ts
2w major
f1 (ppm)

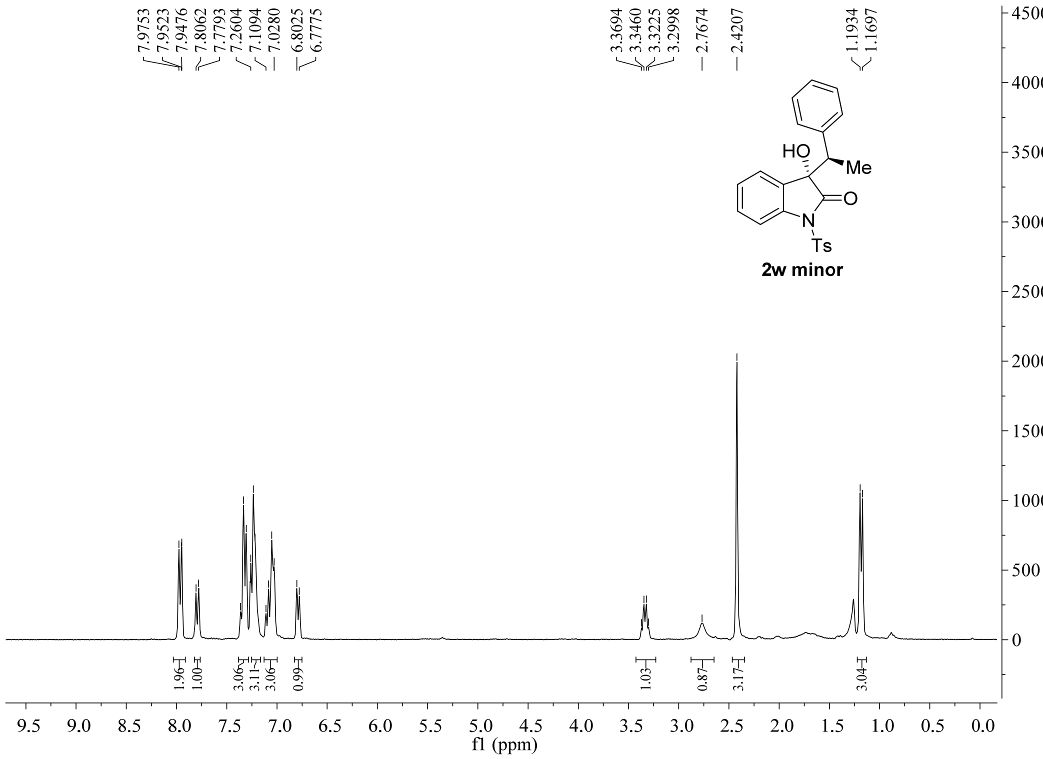

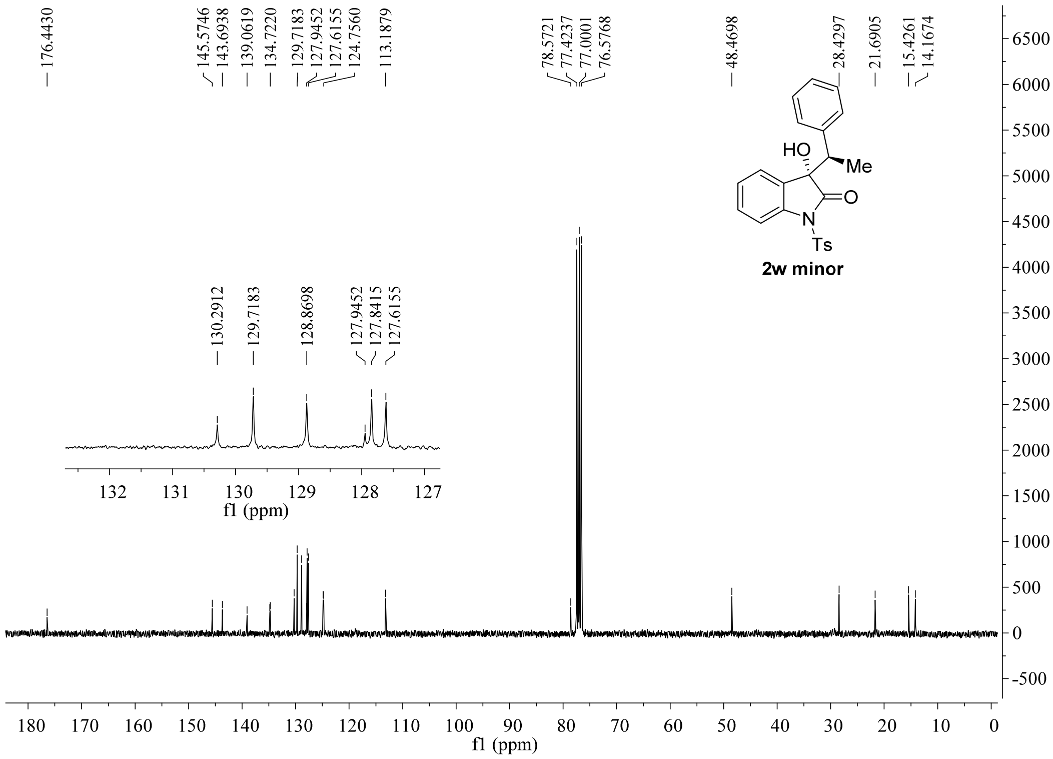

Supplementary Figure 19. ^{1}H and ^{13}C NMR spectra for compound **2w**

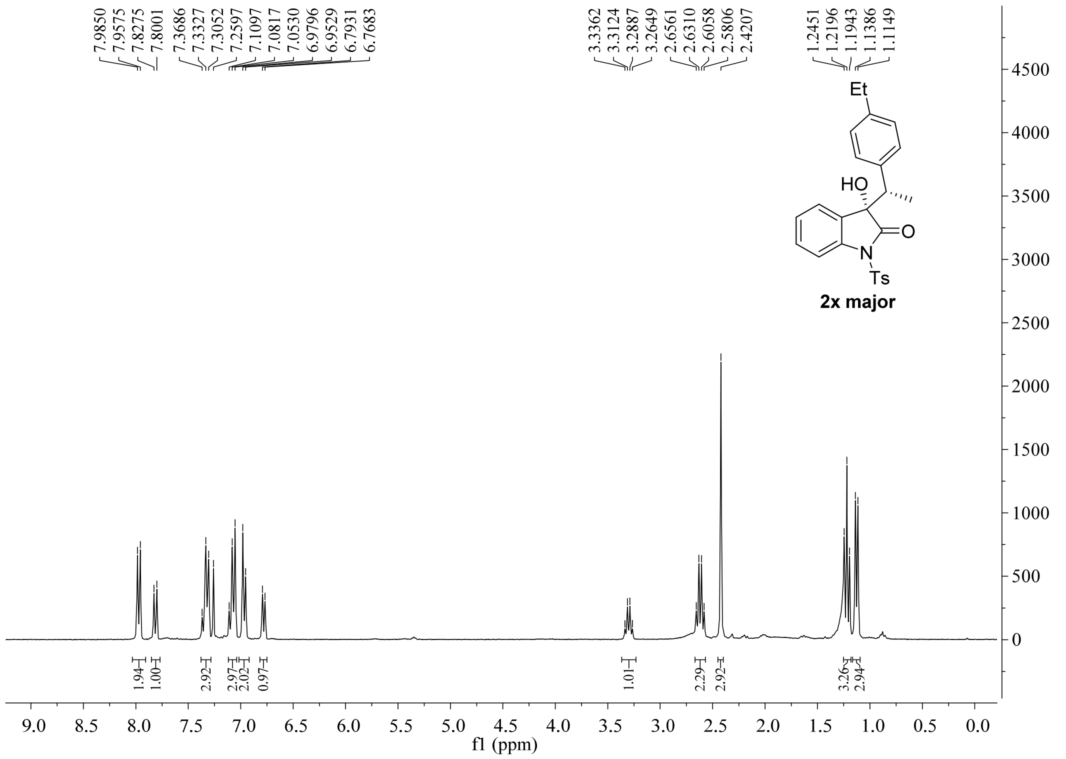
7.9850
7.9575
7.8275
7.8001
7.3686
7.3327
7.3052
7.2597
7.1097
7.0817
7.0530
6.9796
6.9529
6.7931
6.7683
3.3362
3.3124
3.2887
3.2649
2.6561
2.6310
2.6058
2.5806
2.4207
1.2451
1.2196
1.1943
1.1386
1.1149
Et
HO
O
N
Ts
2x major
1.94
1.00
2.92
2.97
2.02
0.97
1.01
2.29
2.92
3.26
2.94
f1 (ppm)

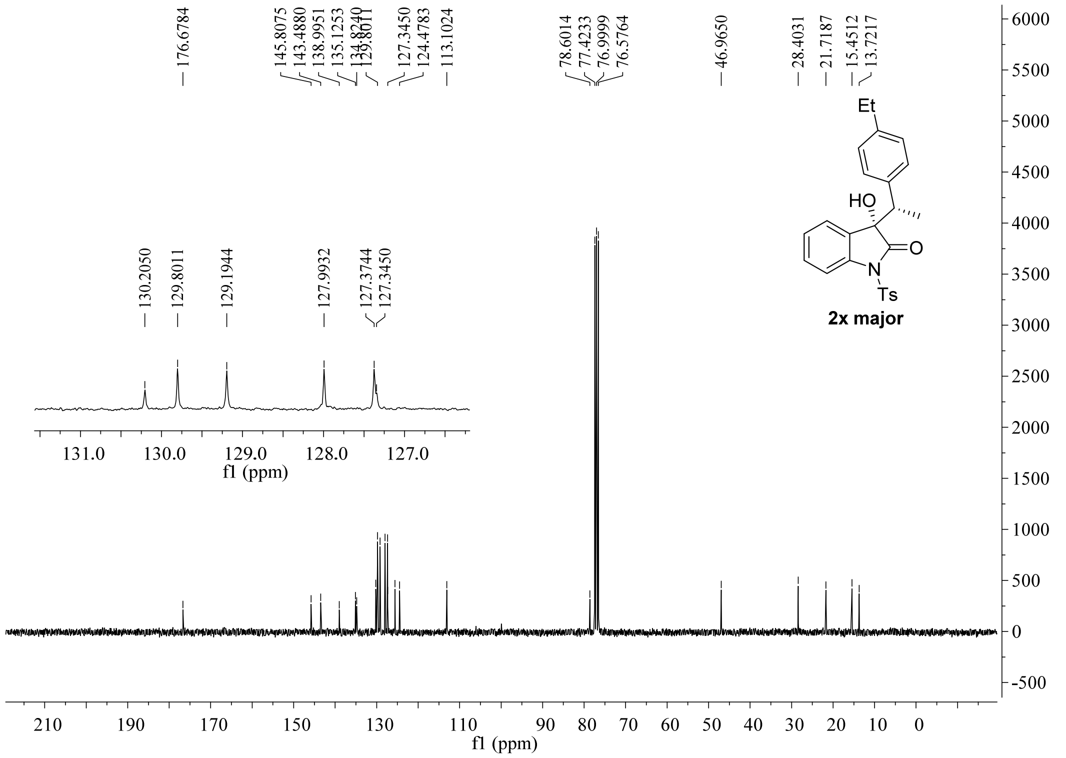
176.6784
145.8075
143.4880
138.9951
135.1253
134.8240
129.8011
127.3450
124.4783
113.1024
78.6014
77.4233
76.9999
76.5764
46.9650
28.4031
21.7187
15.4512
13.7217
Et
HO
O
N
Ts
2x major
130.2050
129.8011
129.1944
127.9932
127.3744
127.3450
f1 (ppm)
f1 (ppm)

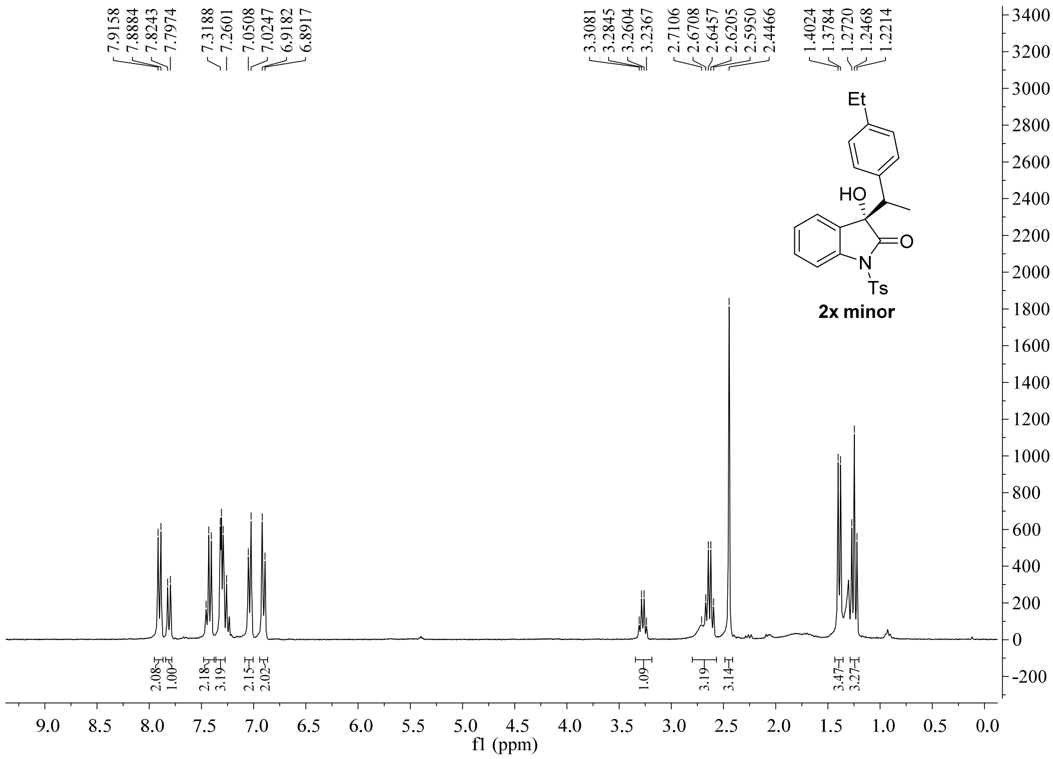

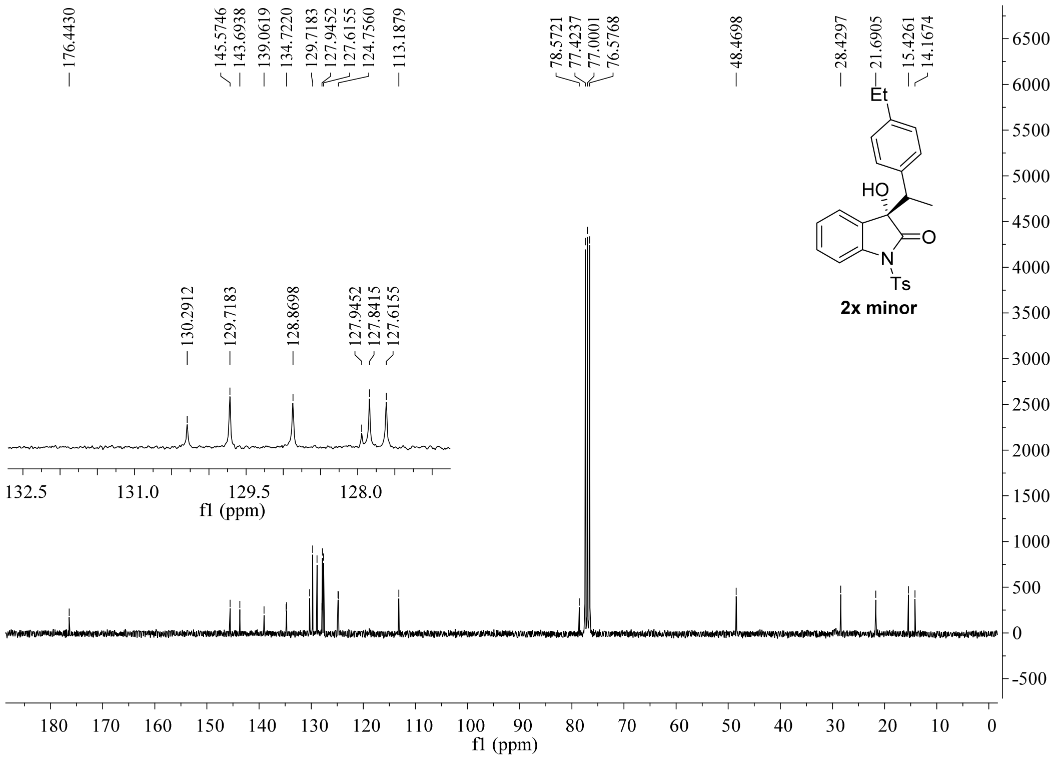

Supplementary Figure 20. ^{1}H and ^{13}C NMR spectra for compound **2x**

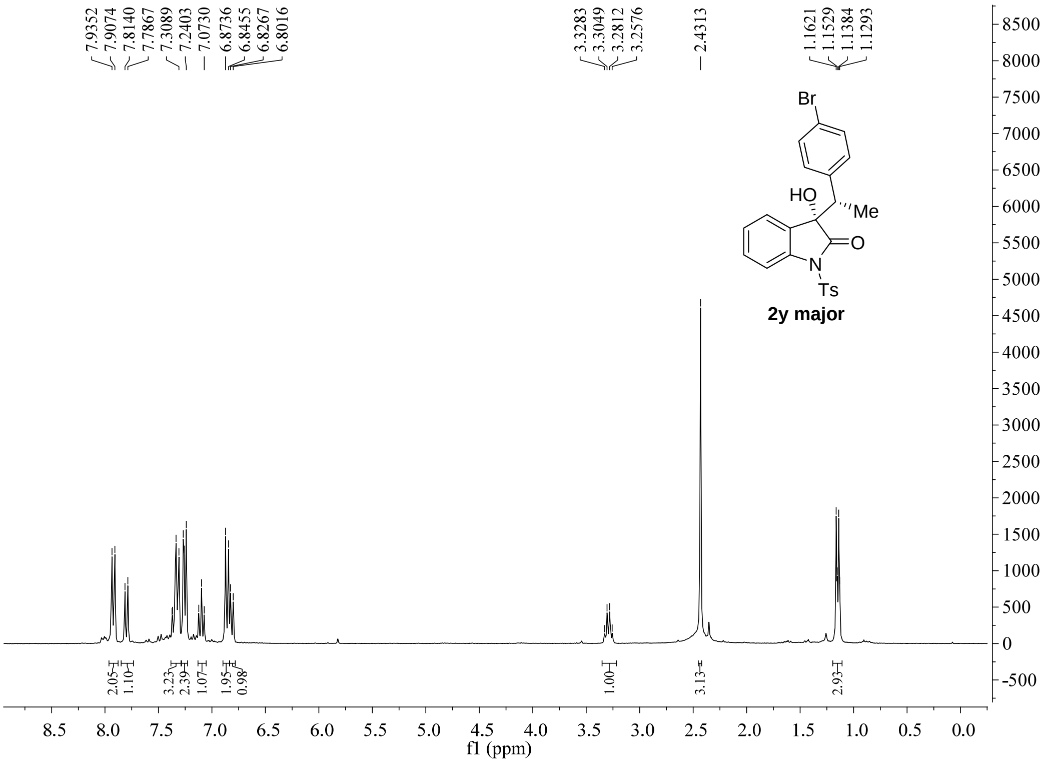
7.9352
7.9074
7.8140
7.7867
7.3089
7.2403
7.0730
6.8736
6.8455
6.8267
6.8016
3.3283
3.3049
3.2812
3.2576
2.4313
1.1621
1.1529
1.1384
1.1293
Br
HO
Me
O
N
Ts
2y major
2.05
1.10
3.23
2.39
1.07
1.95
0.98
1.00
3.13
2.93
f1 (ppm)

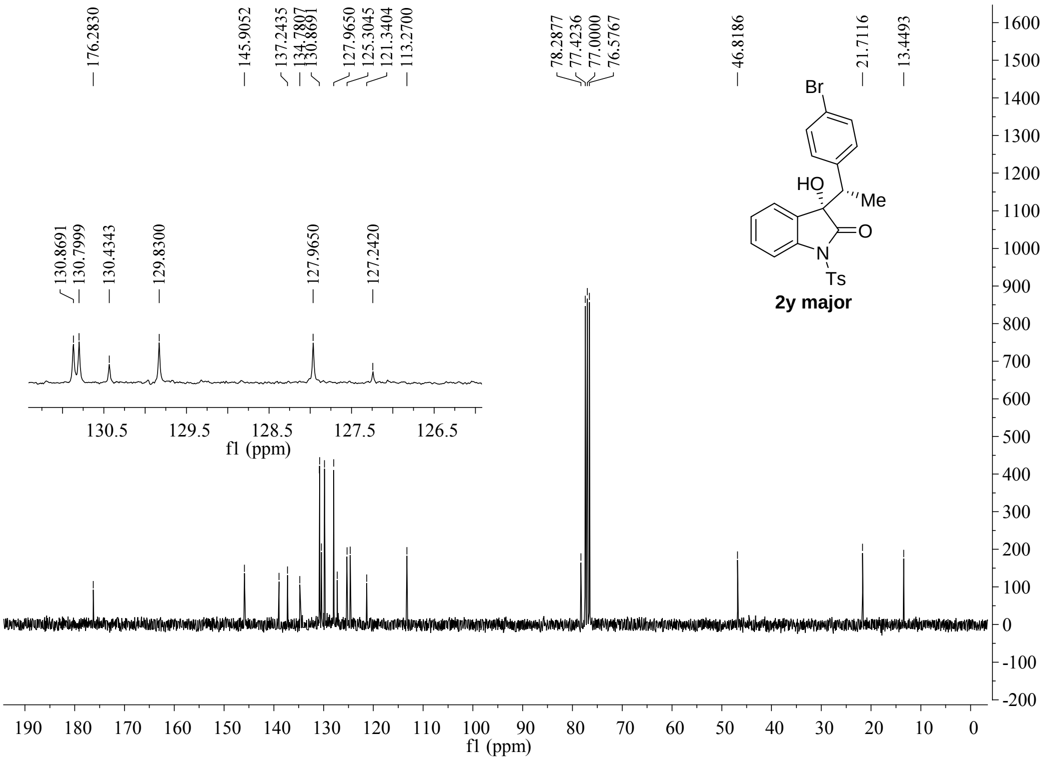
176.2830
145.9052
137.2435
134.7807
130.8691
127.9650
125.3045
121.3404
113.2700
78.2877
77.4236
77.0000
76.5767
46.8186
21.7116
13.4493
130.8691
130.7999
130.4343
129.8300
127.9650
127.2420
f1 (ppm)
Br
HO
Me
O
N
Ts
2y major
f1 (ppm)

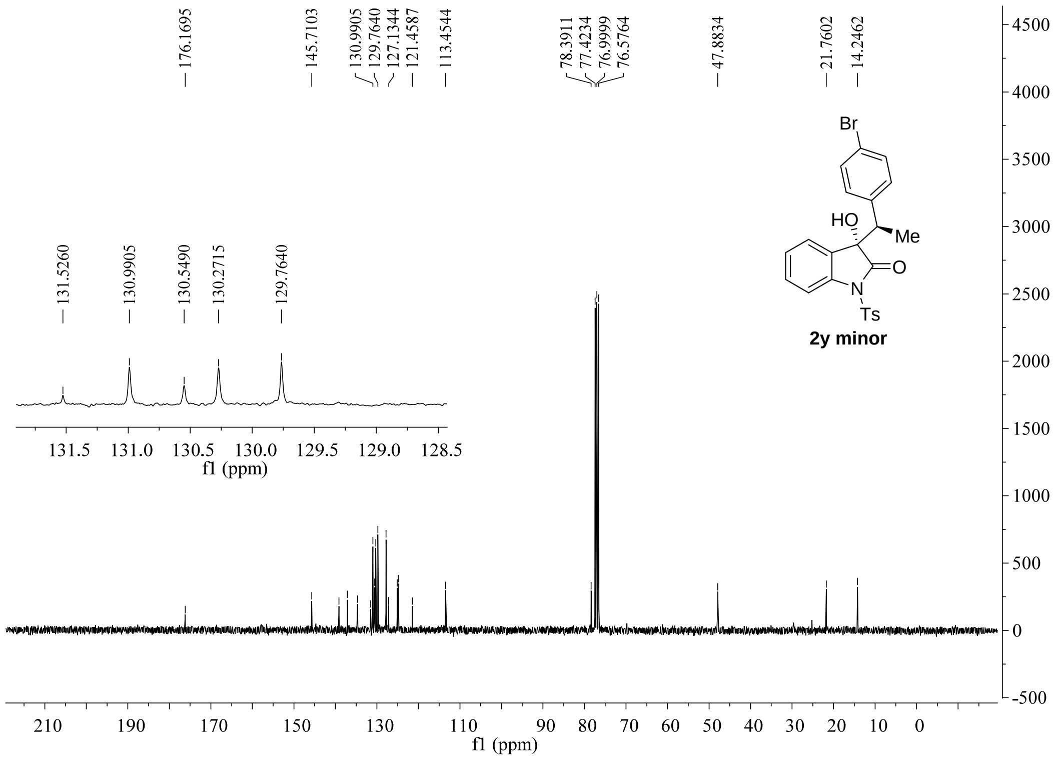

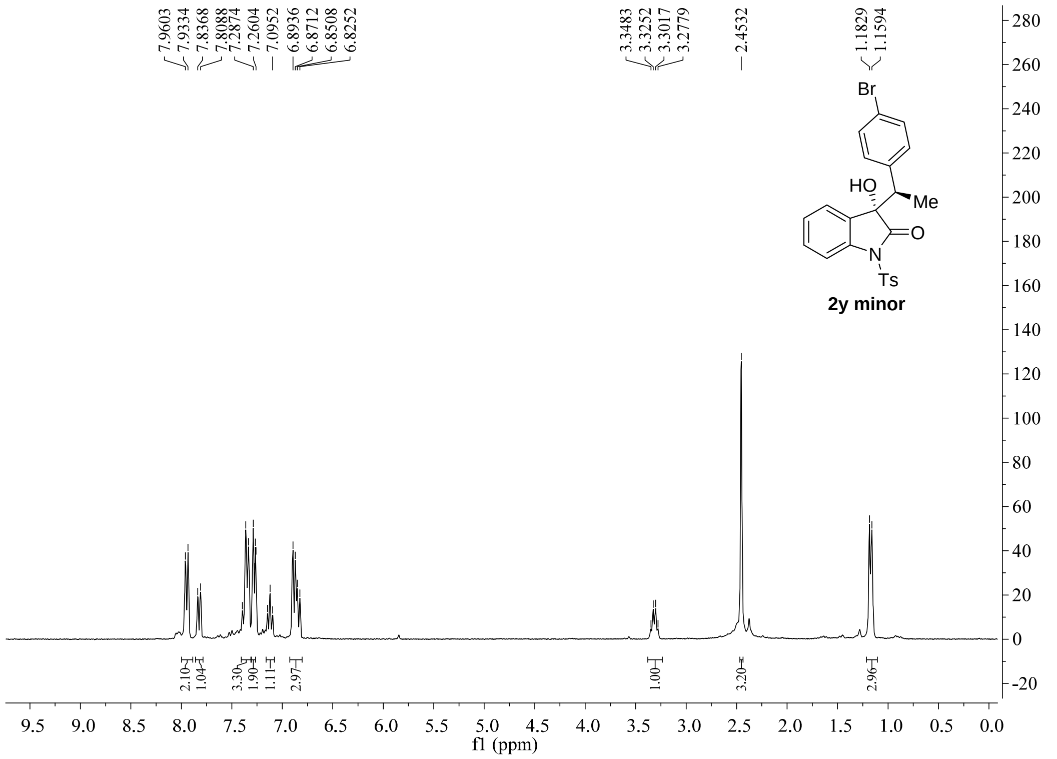

Supplementary Figure 21. ^{1}H and ^{13}C NMR spectra for compound **2y**

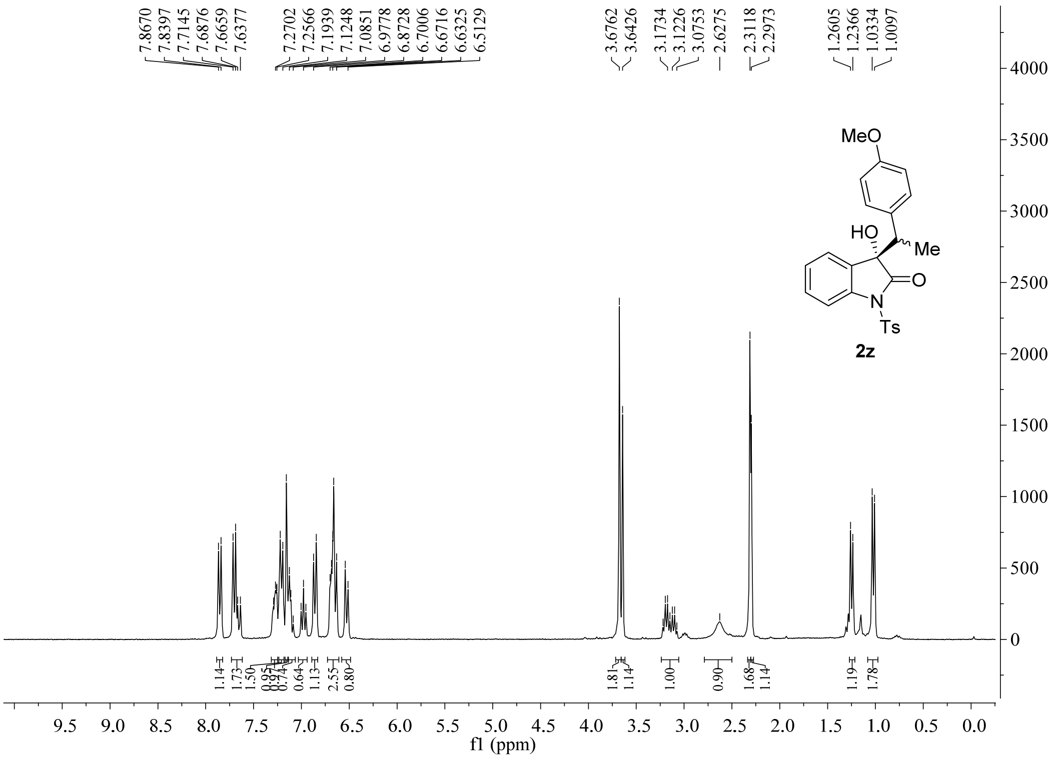

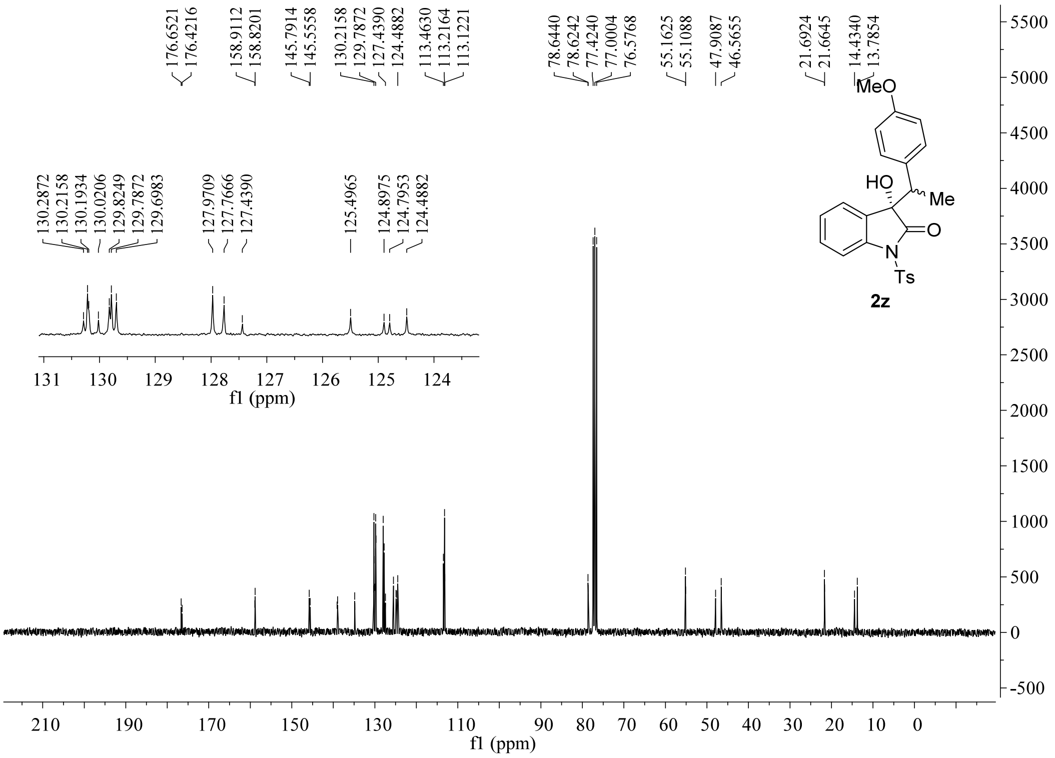

Supplementary Figure 22. ^{1}H and ^{13}C NMR spectra for compound **2z**

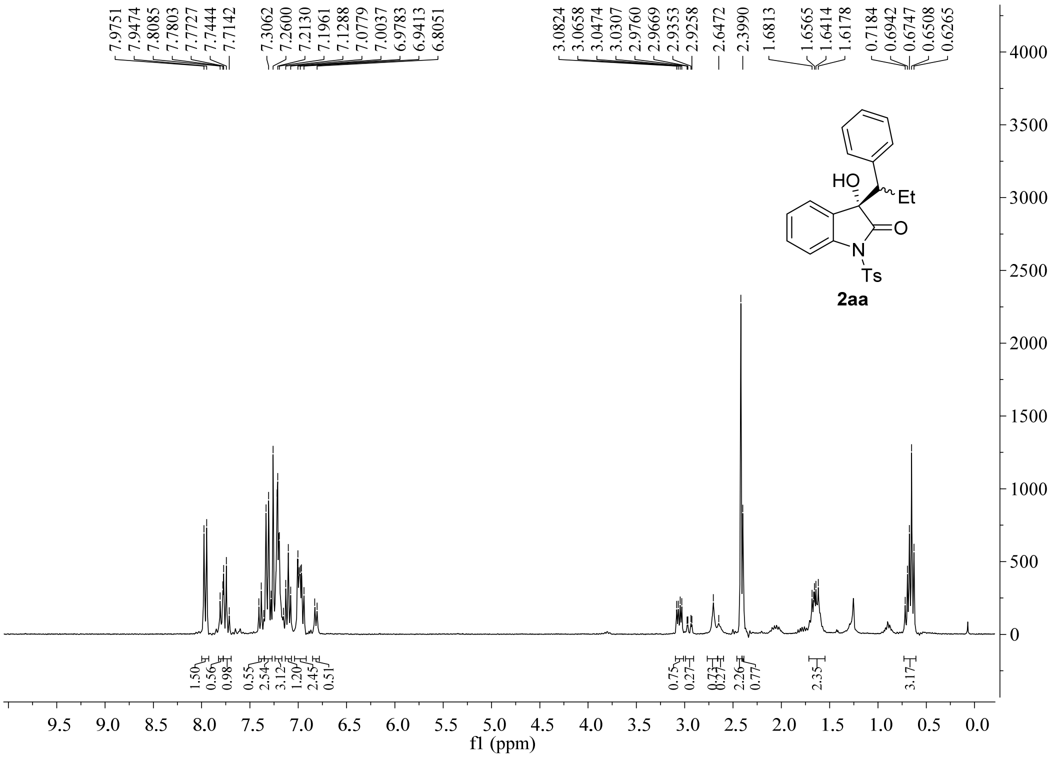

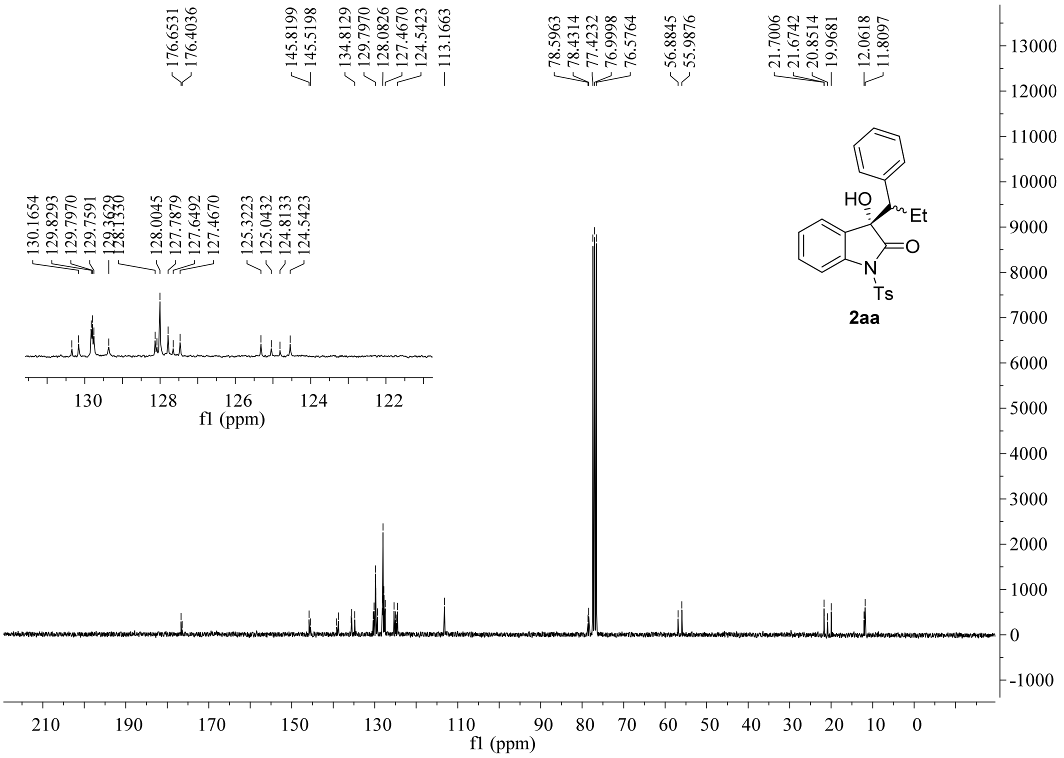

Supplementary Figure 23. ^{1}H and ^{13}C NMR spectra for compound **2aa**

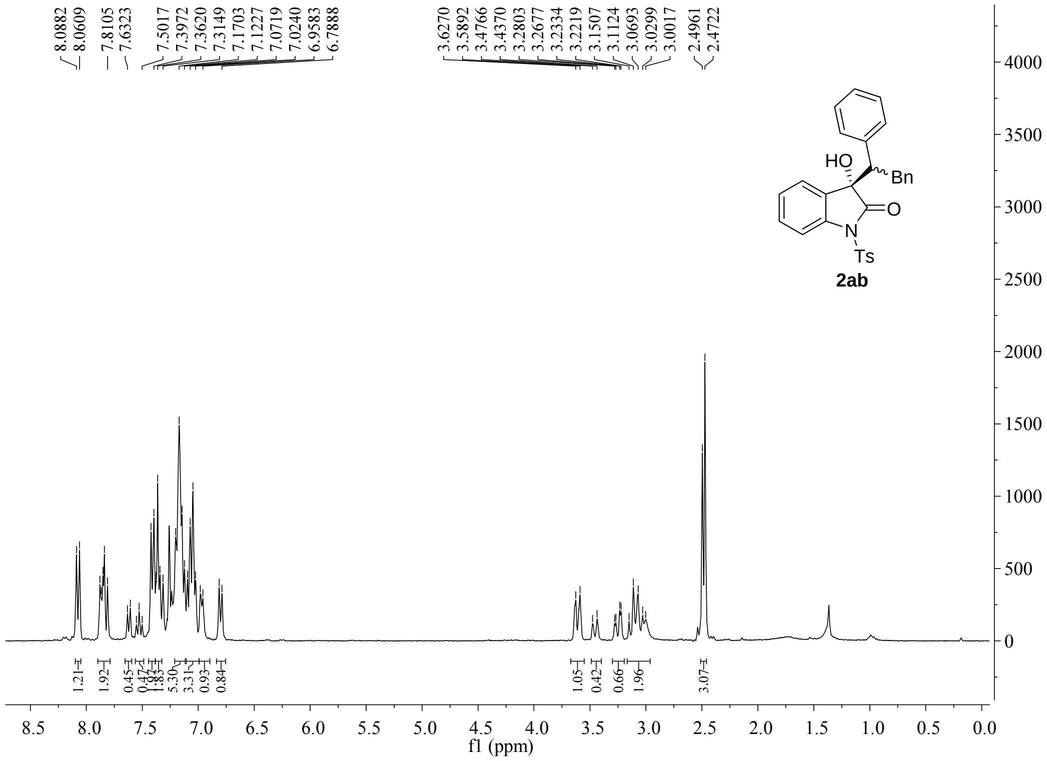

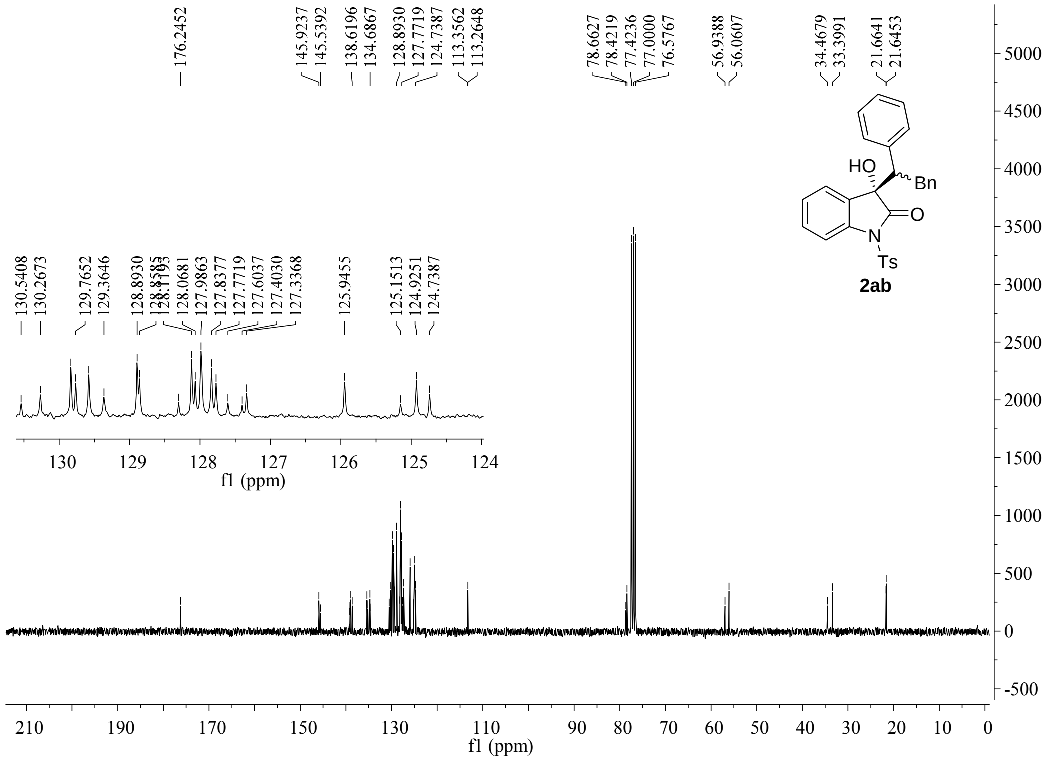

Supplementary Figure 24. ^{1}H and ^{13}C NMR spectra for compound **2ab**

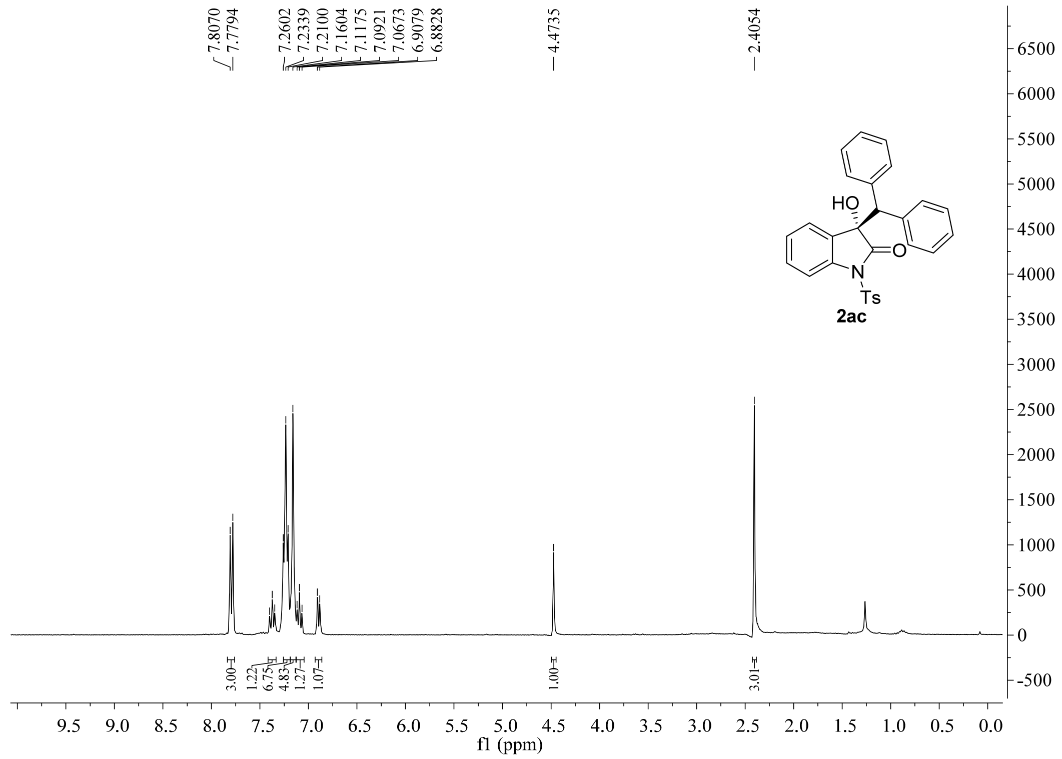

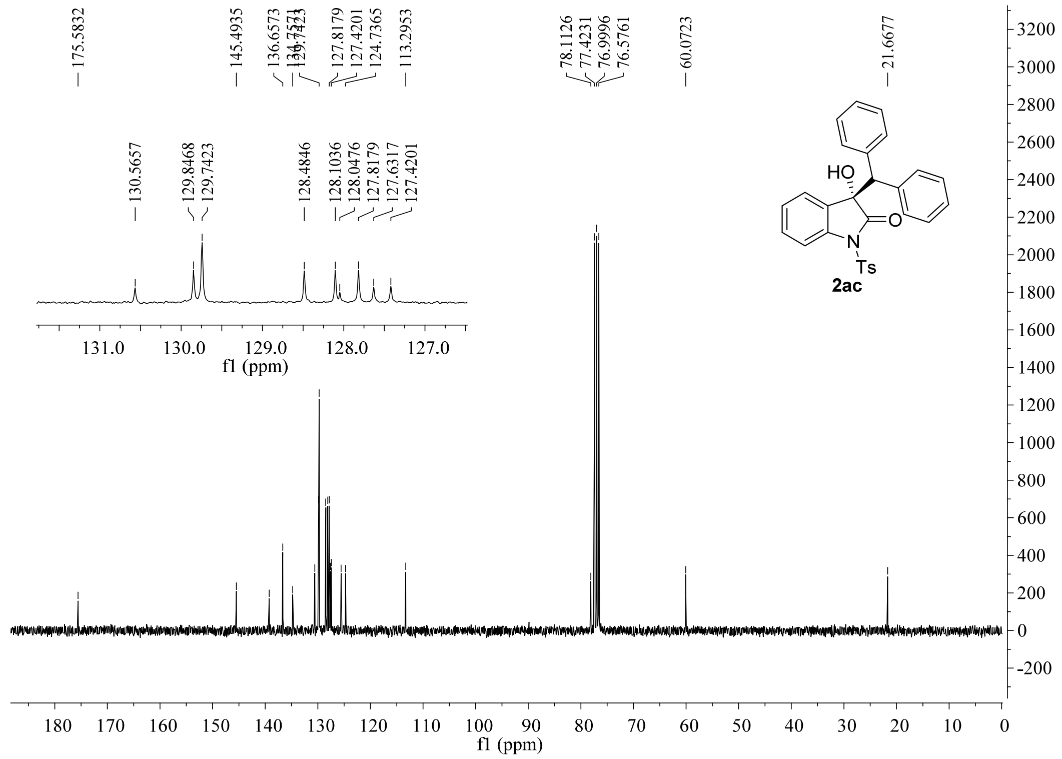

Supplementary Figure 25. ^{1}H and ^{13}C NMR spectra for compound **2ac**

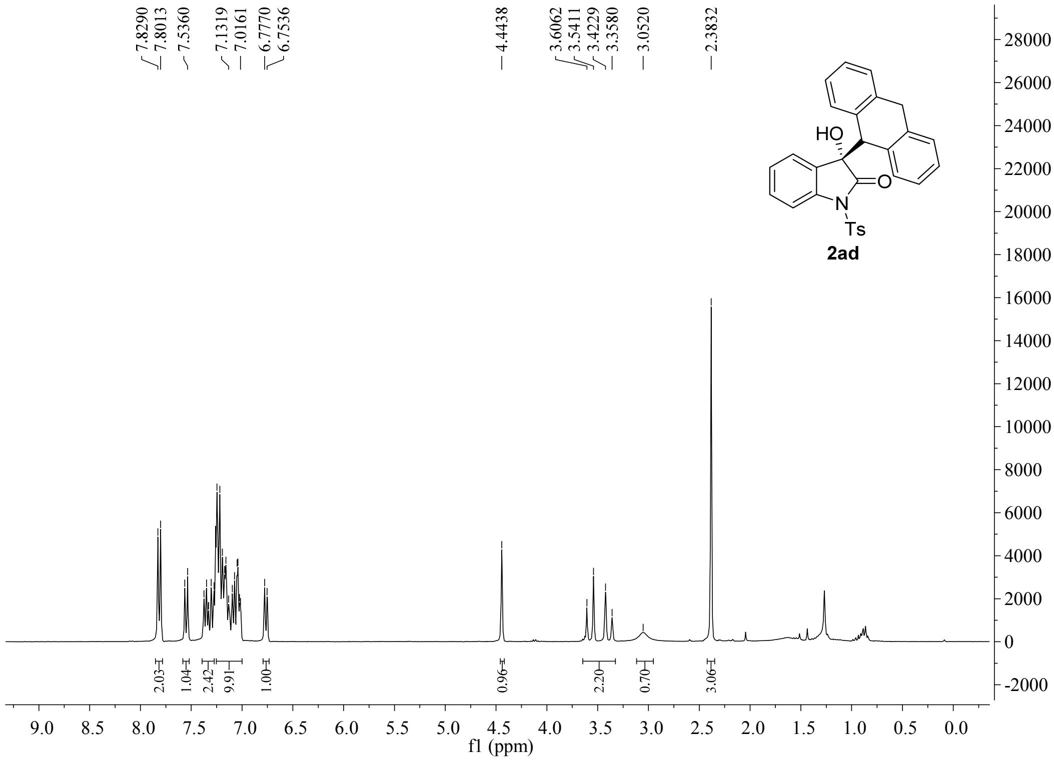

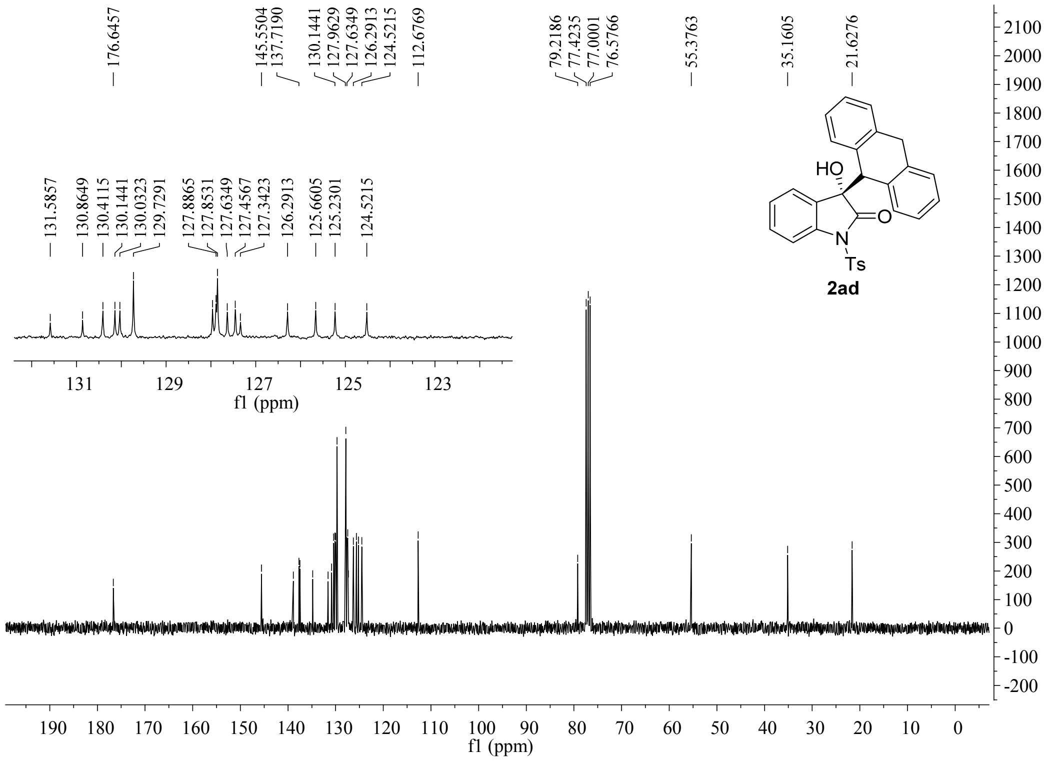

Supplementary Figure 26. ^{1}H and ^{13}C NMR spectra for compound **2ac**

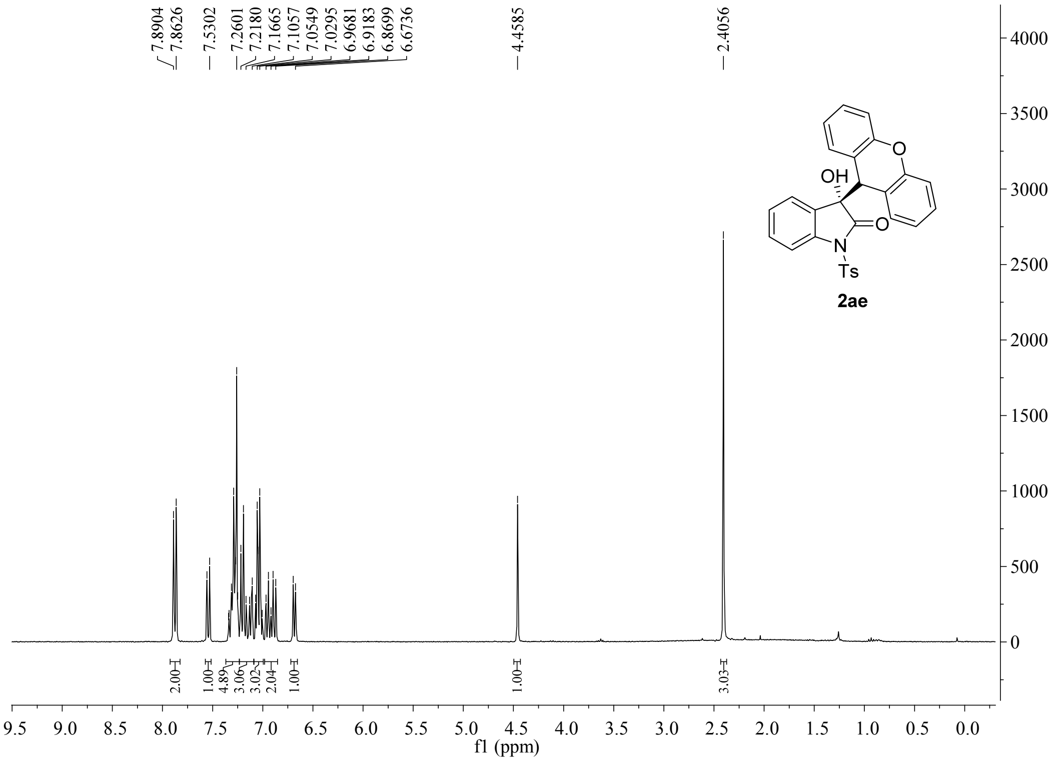

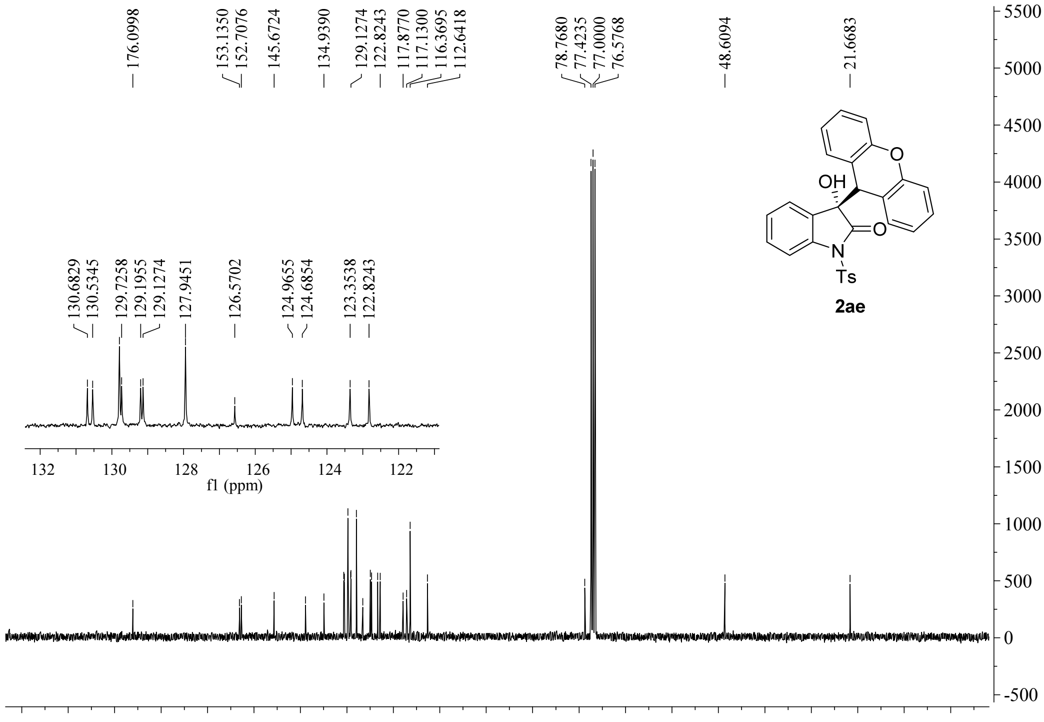

Supplementary Figure 27. ^{1}H and ^{13}C NMR spectra for compound **2ae**

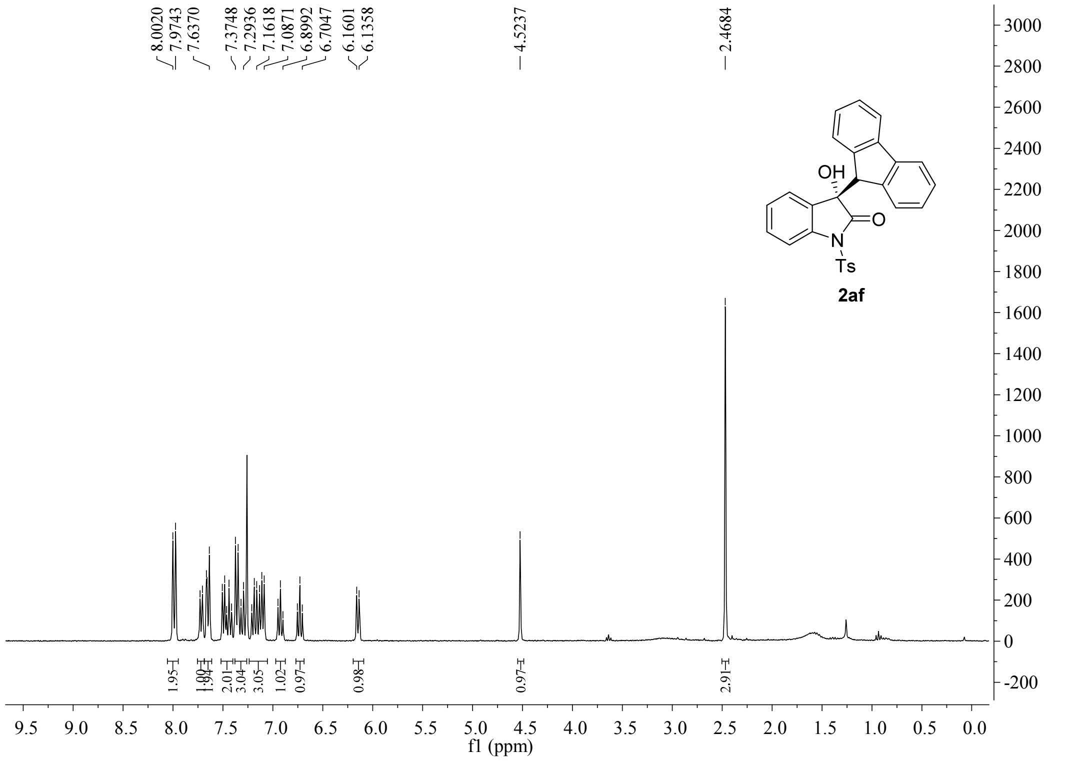

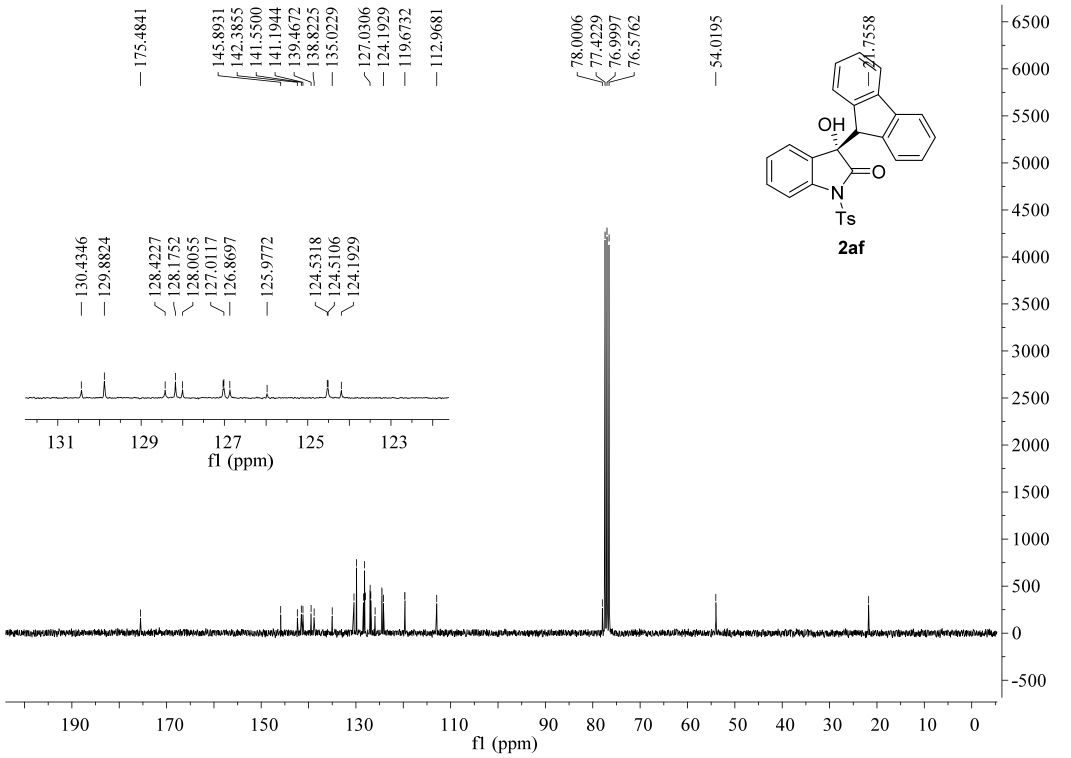

Supplementary Figure 28. ^{1}H and ^{13}C NMR spectra for compound **2af**

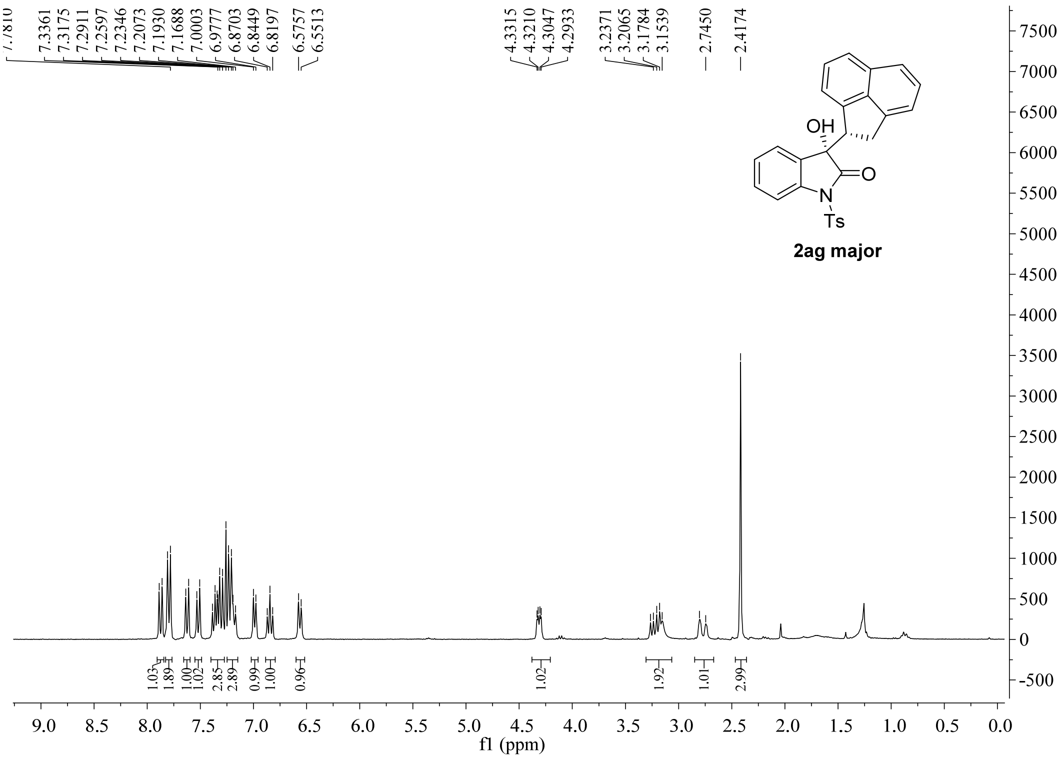
7.7810
7.3361
7.3175
7.2911
7.2597
7.2346
7.2073
7.1930
7.1688
7.0003
6.9777
6.8703
6.8449
6.8197
6.5757
6.5513
4.3315
4.3210
4.3047
4.2933
3.2371
3.2065
3.1784
3.1539
2.7450
2.4174
OH
O
N
Ts
2ag major
1.03
1.89
1.00
1.02
2.85
2.89
0.99
1.00
0.96
1.02
1.92
1.01
2.99
f1 (ppm)

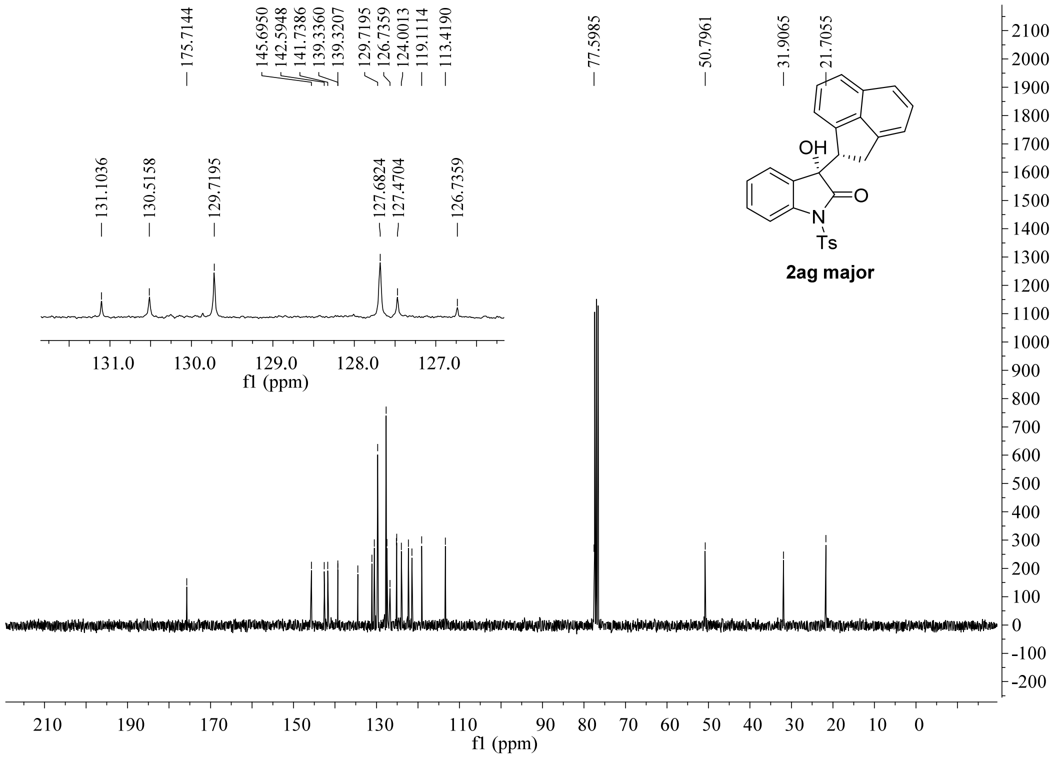
175.7144
145.6950
142.5948
141.7386
139.3360
139.3207
129.7195
126.7359
124.0013
119.1114
113.4190
77.5985
50.7961
31.9065
21.7055
131.1036
130.5158
129.7195
127.6824
127.4704
126.7359
f1 (ppm)
OH
O
N
Ts
2ag major
f1 (ppm)

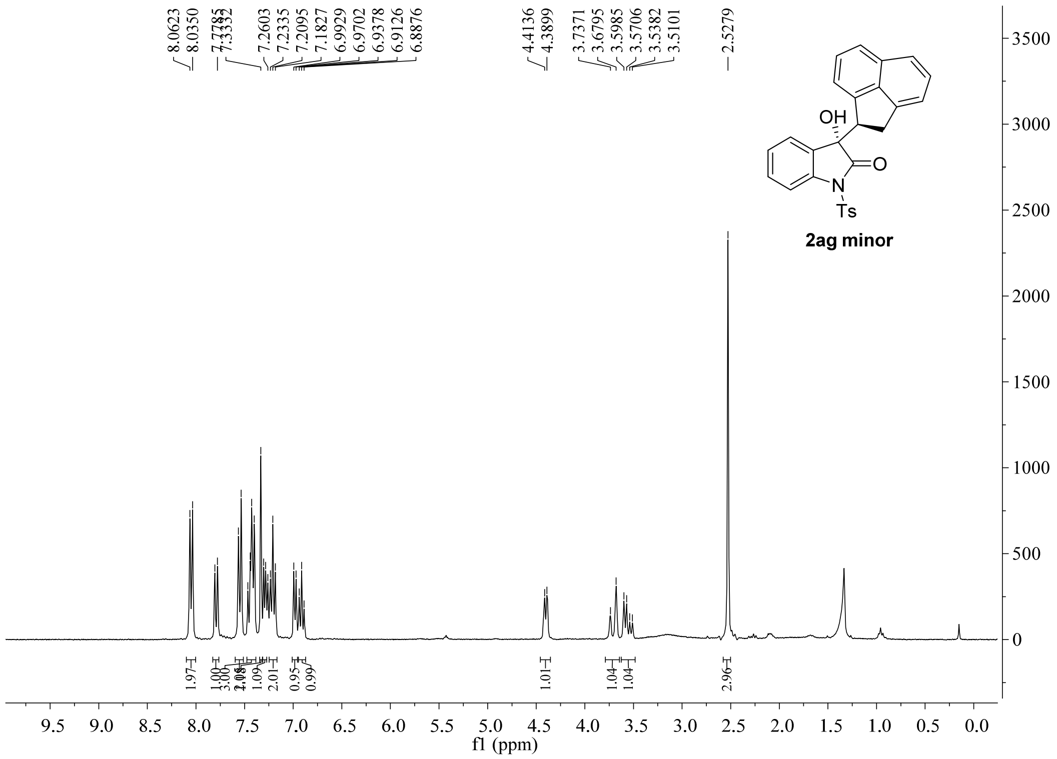

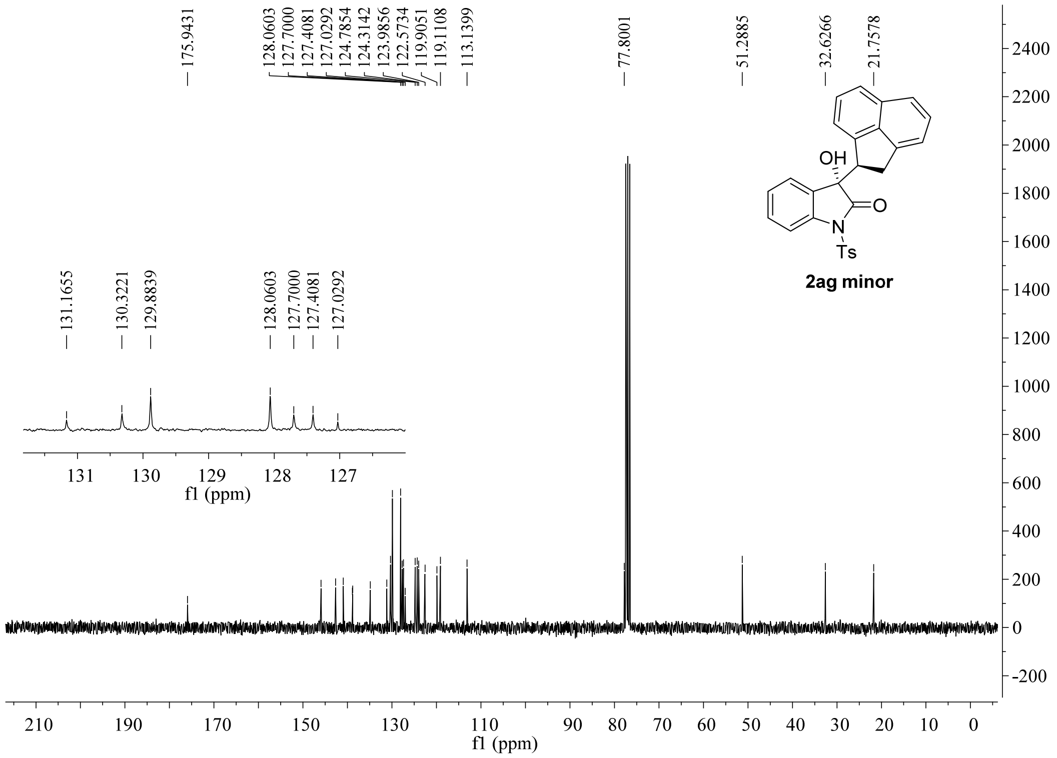

Supplementary Figure 29. ^{1}H and ^{13}C NMR spectra for compound **2ag**

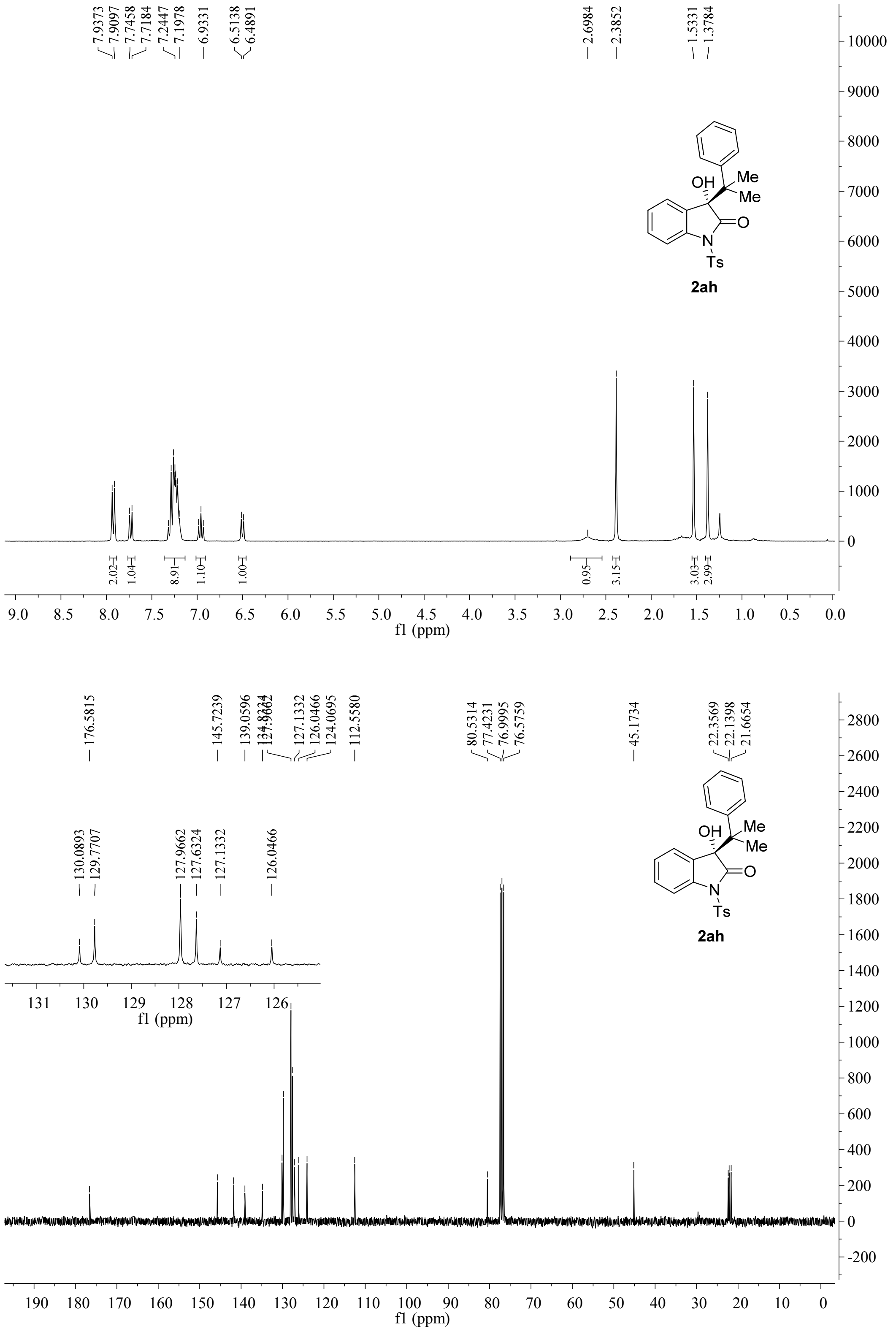

Supplementary Figure 30. ^{1}H and ^{13}C NMR spectra for compound **2ah**

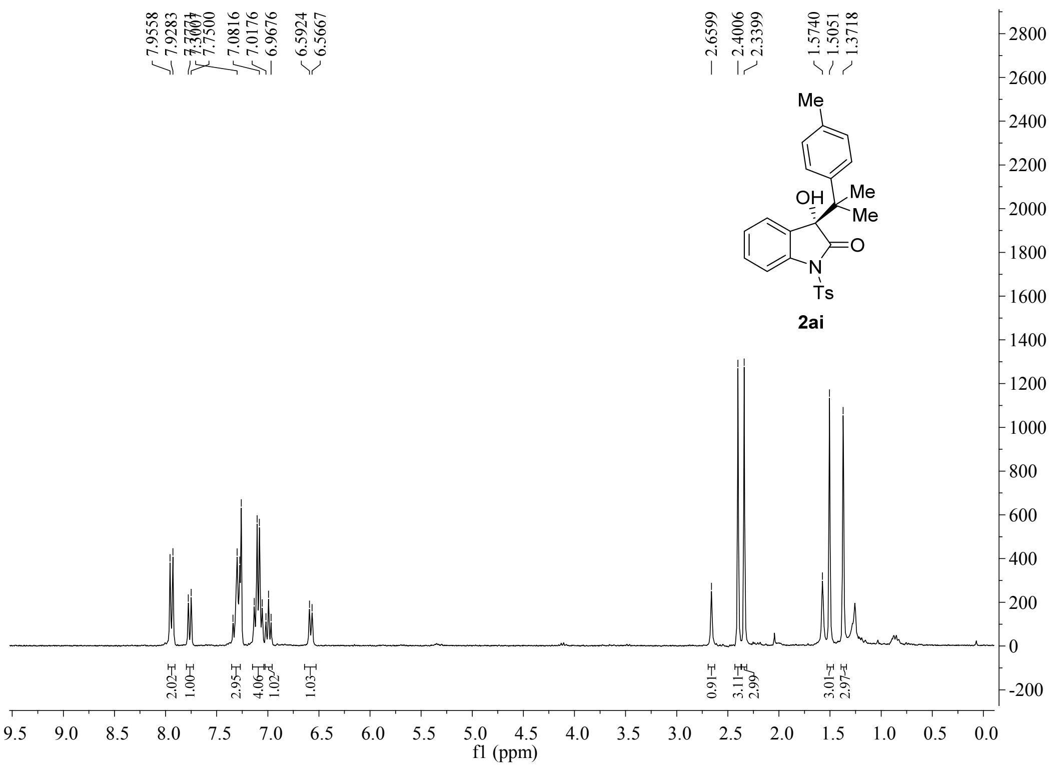

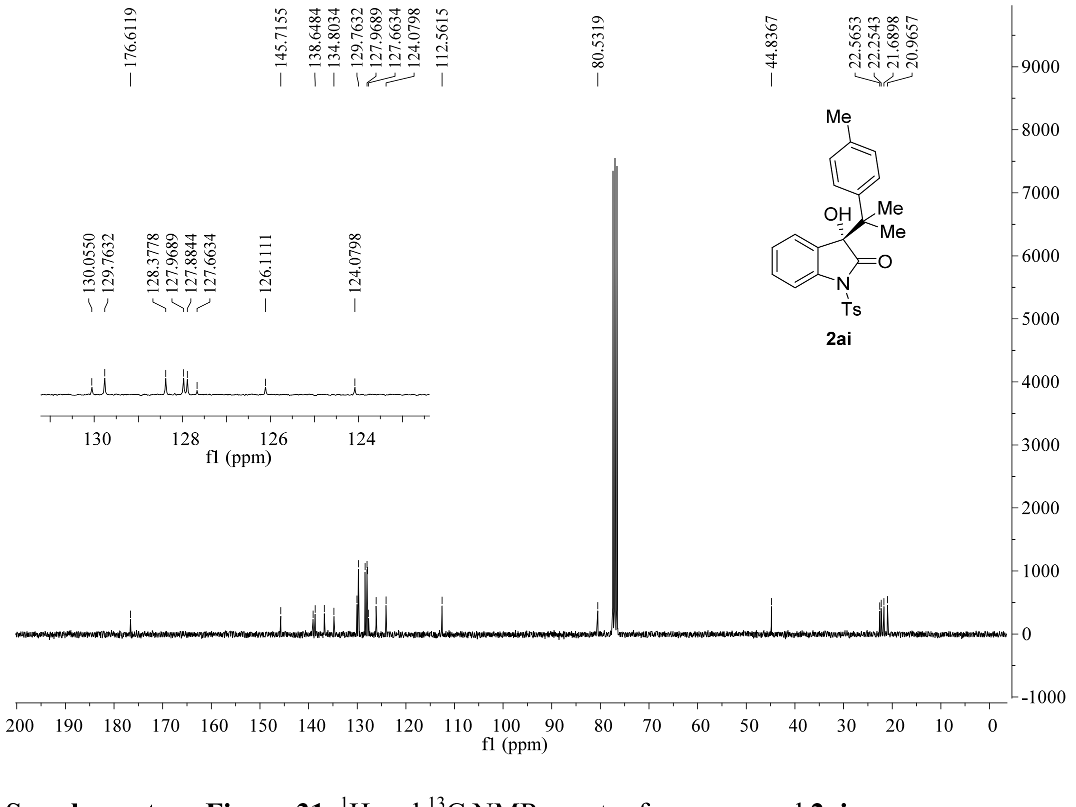

Supplementary Figure 31. ^{1}H and ^{13}C NMR spectra for compound **2ai**

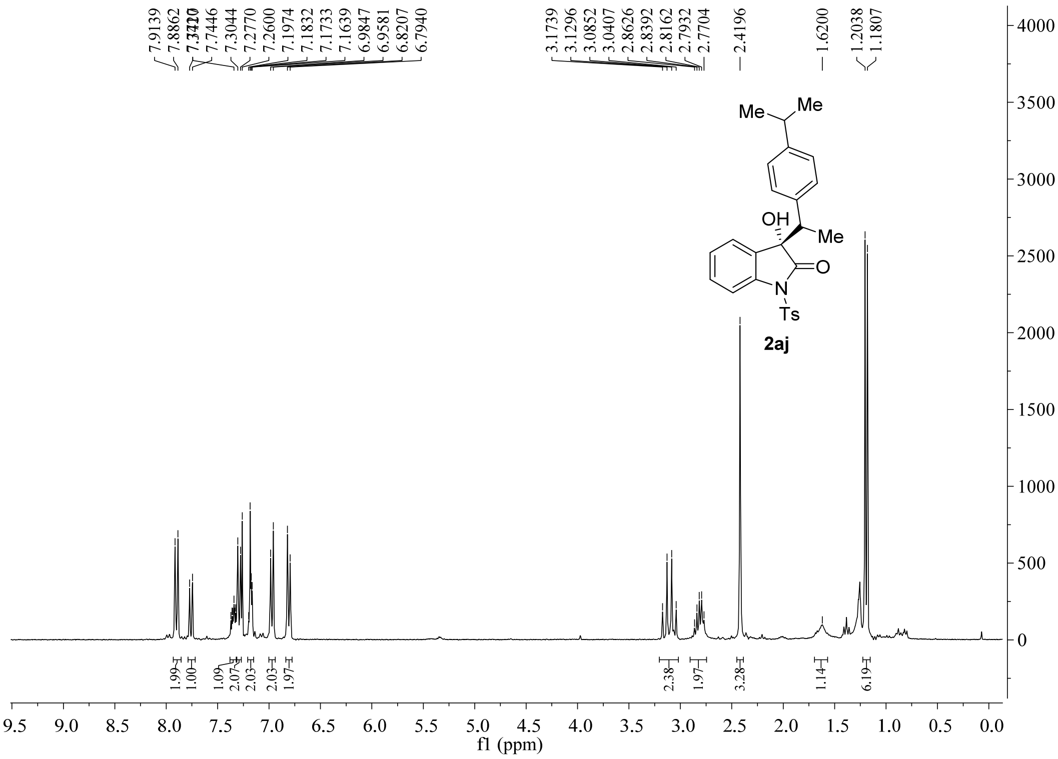

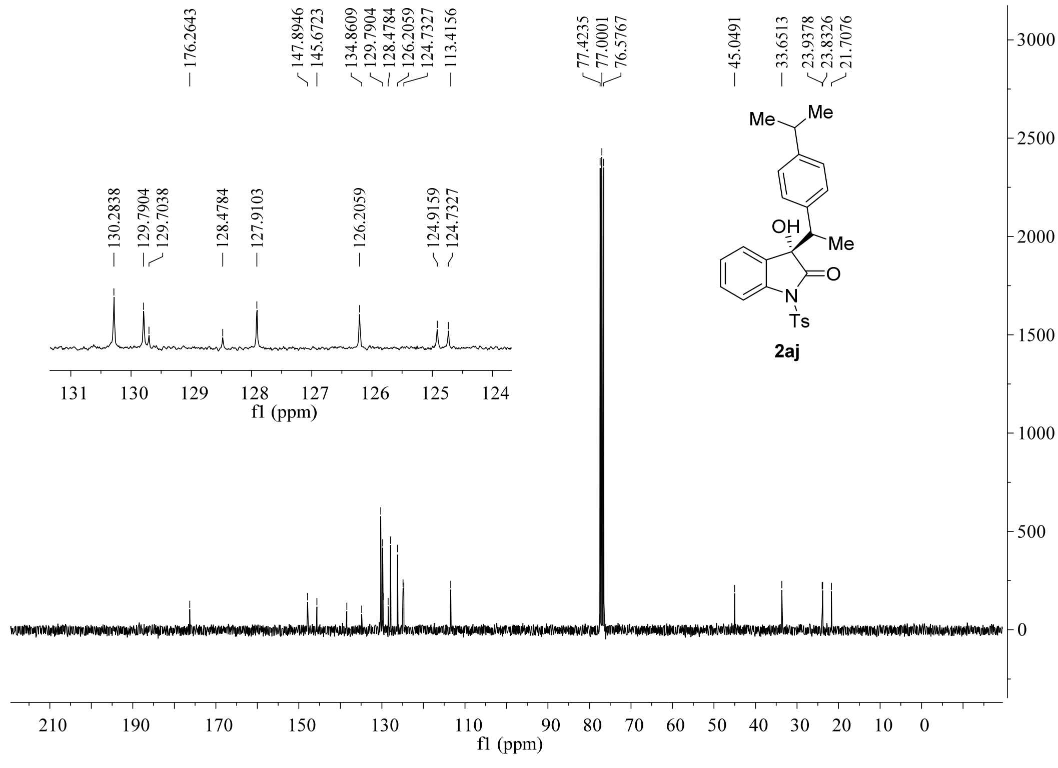

Supplementary Figure 32. ^{1}H and ^{13}C NMR spectra for compound **2aj**

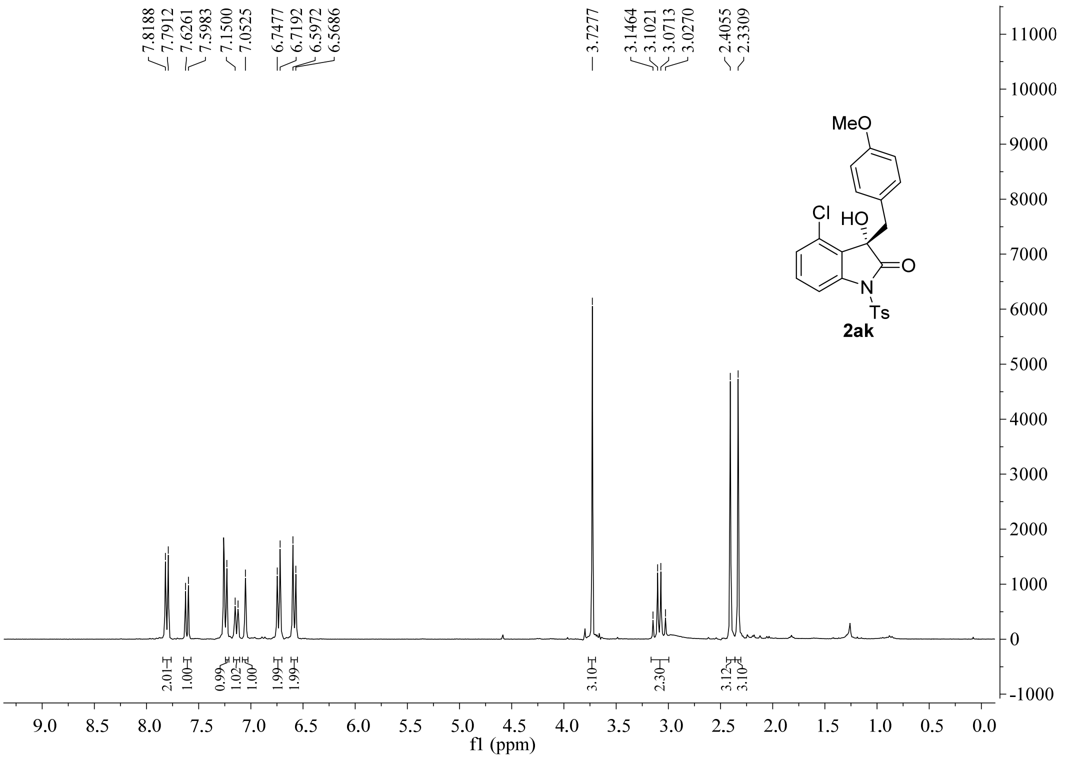

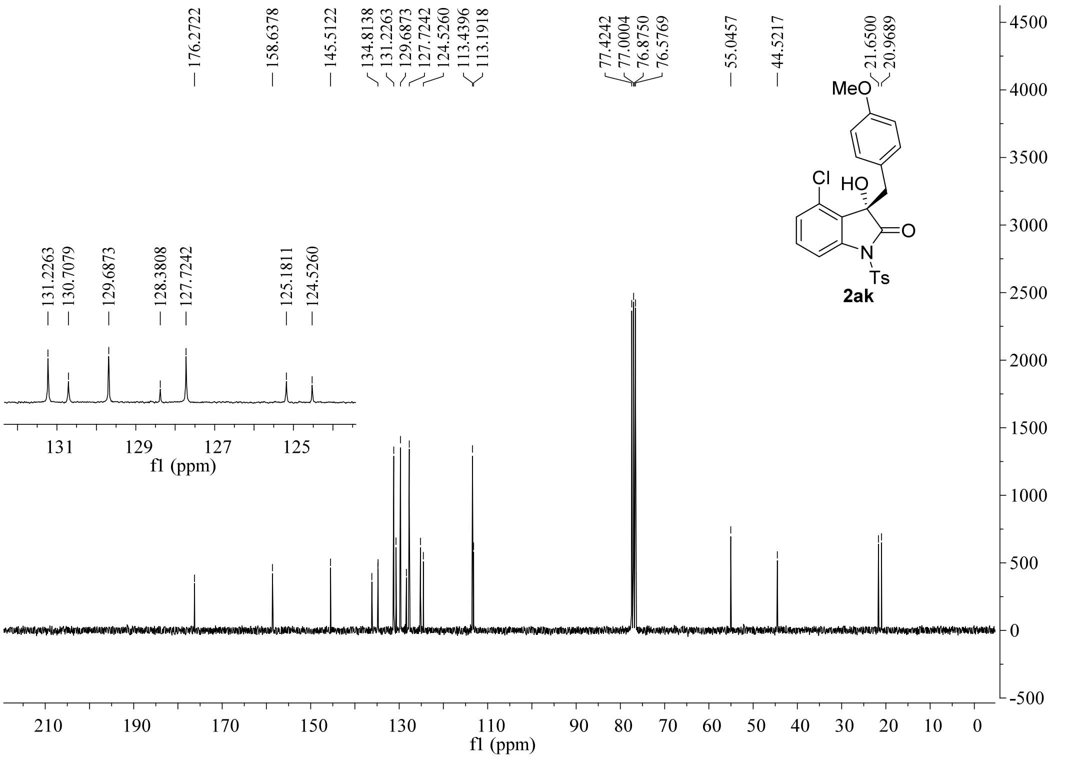

Supplementary Figure 33. ^{1}H and ^{13}C NMR spectra for compound **2ak**

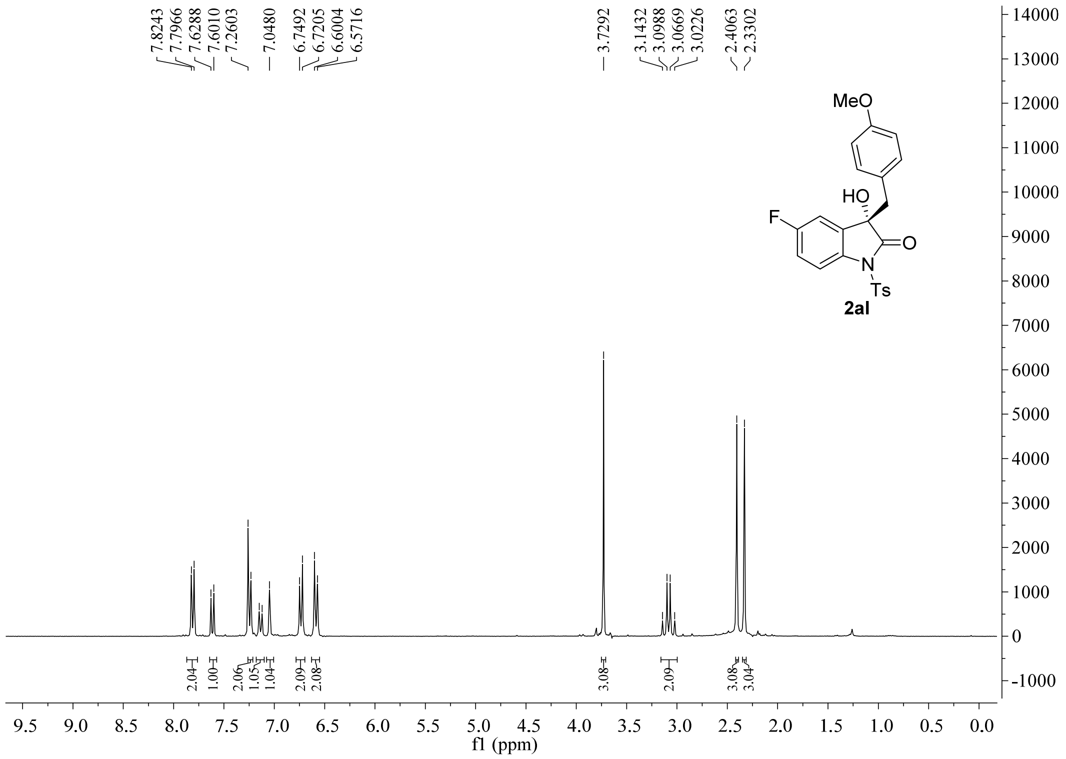

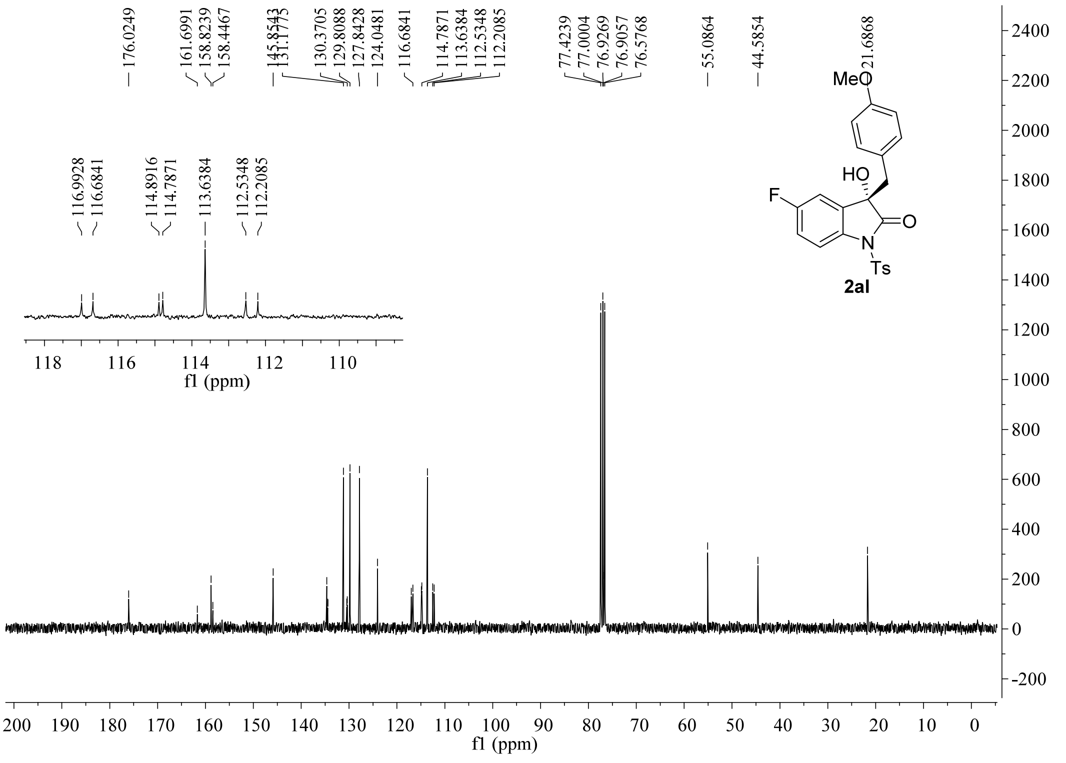

Supplementary Figure 34. ^{1}H and ^{13}C NMR spectra for compound **2al**

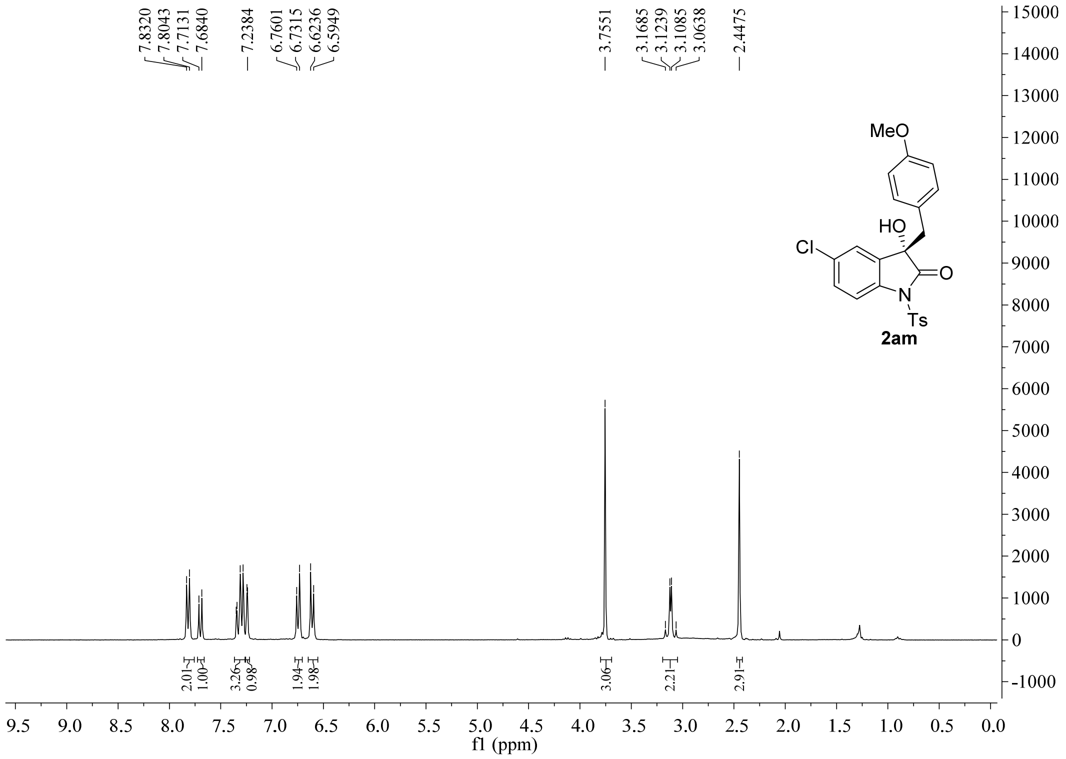

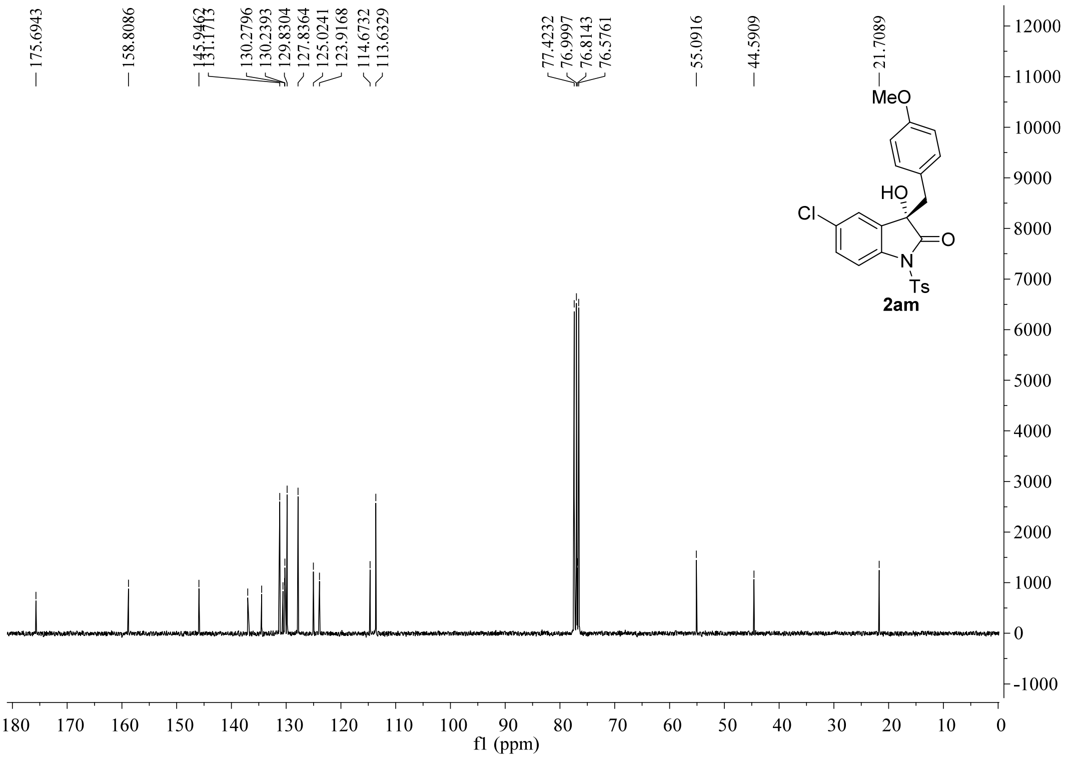

Supplementary Figure 35. ^{1}H and ^{13}C NMR spectra for compound **2am**

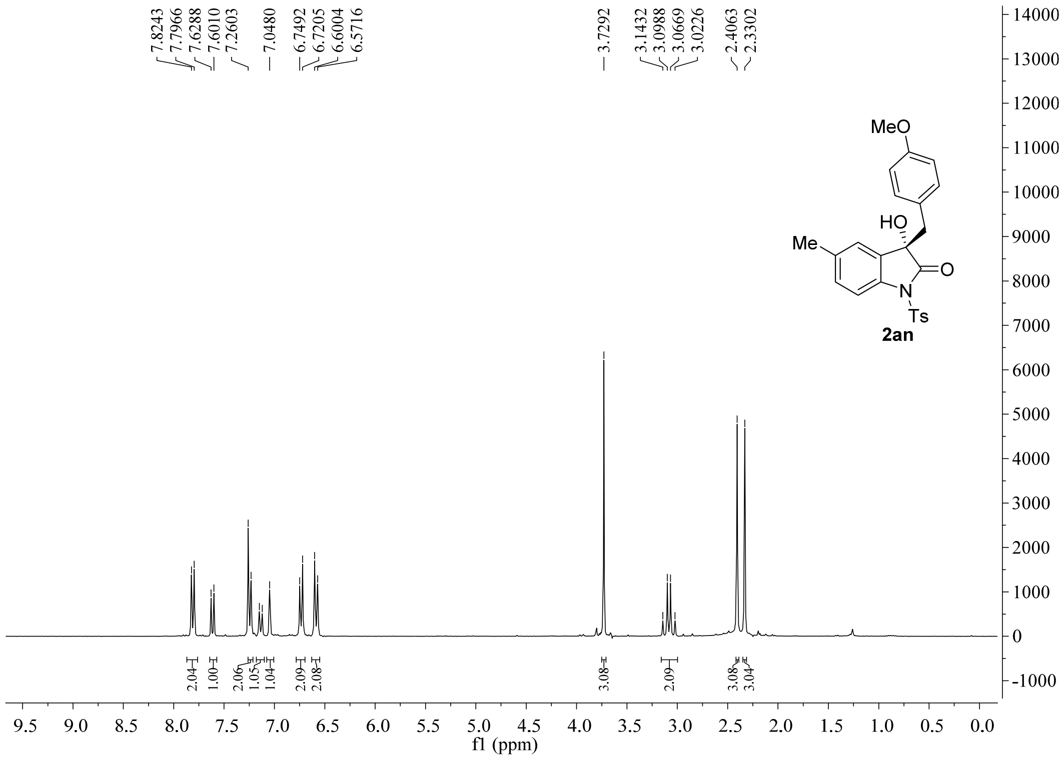

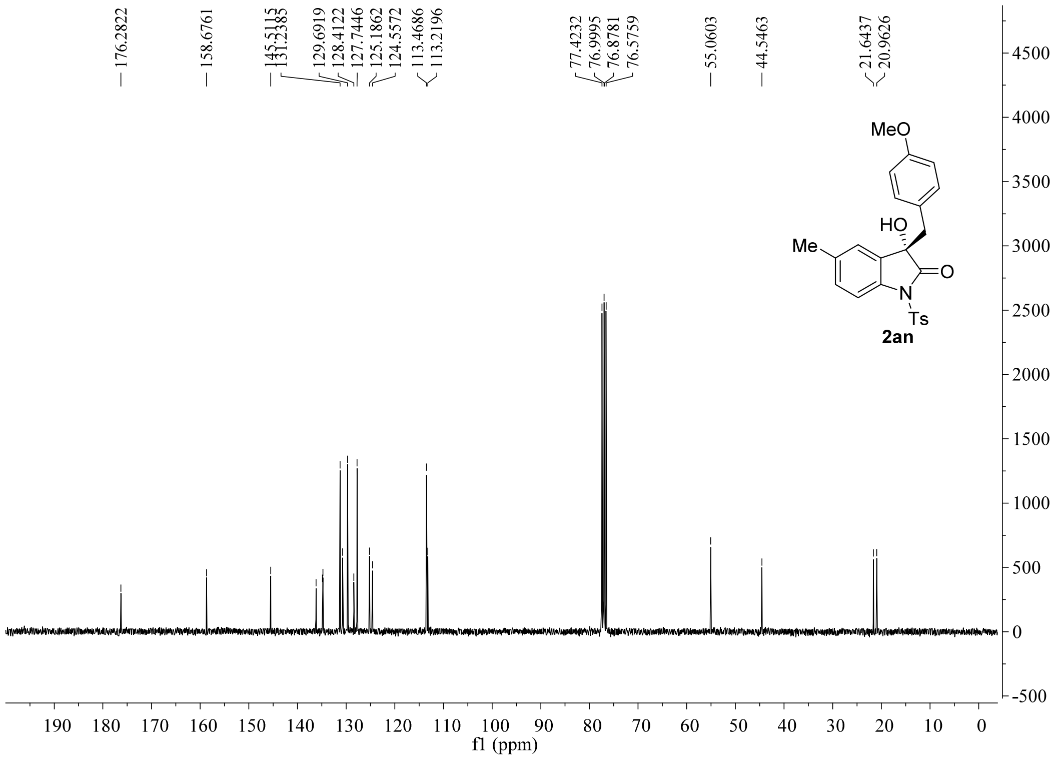

Supplementary Figure 36. ^{1}H and ^{13}C NMR spectra for compound **2an**

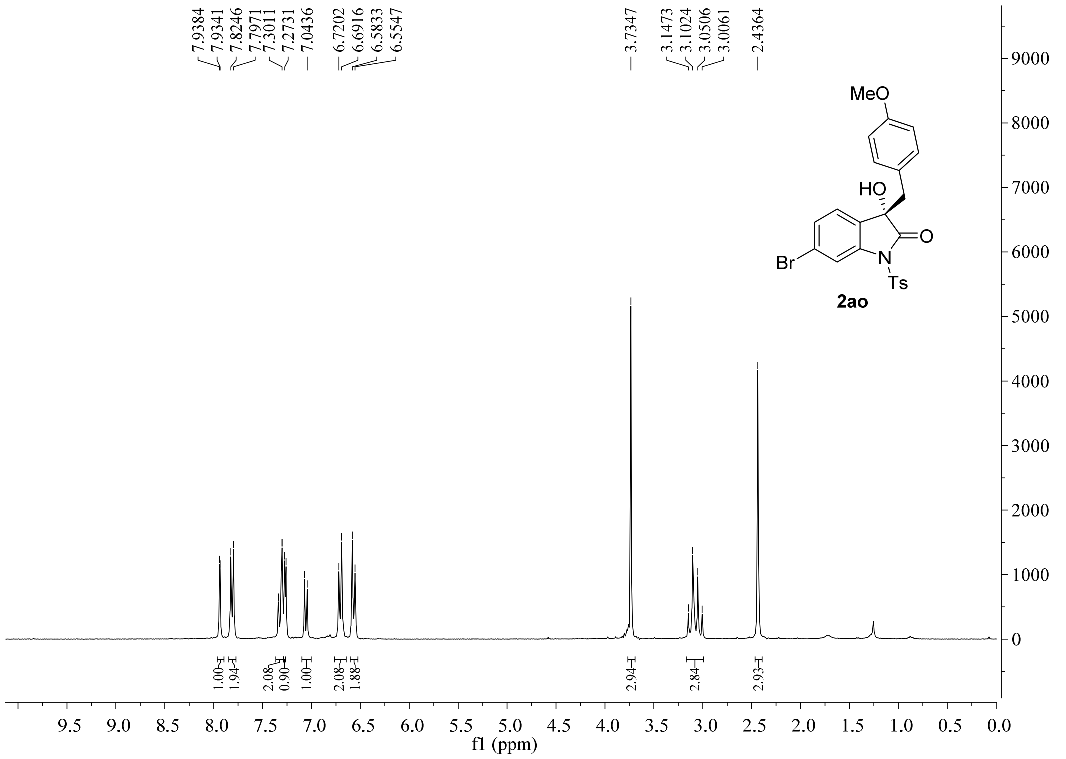

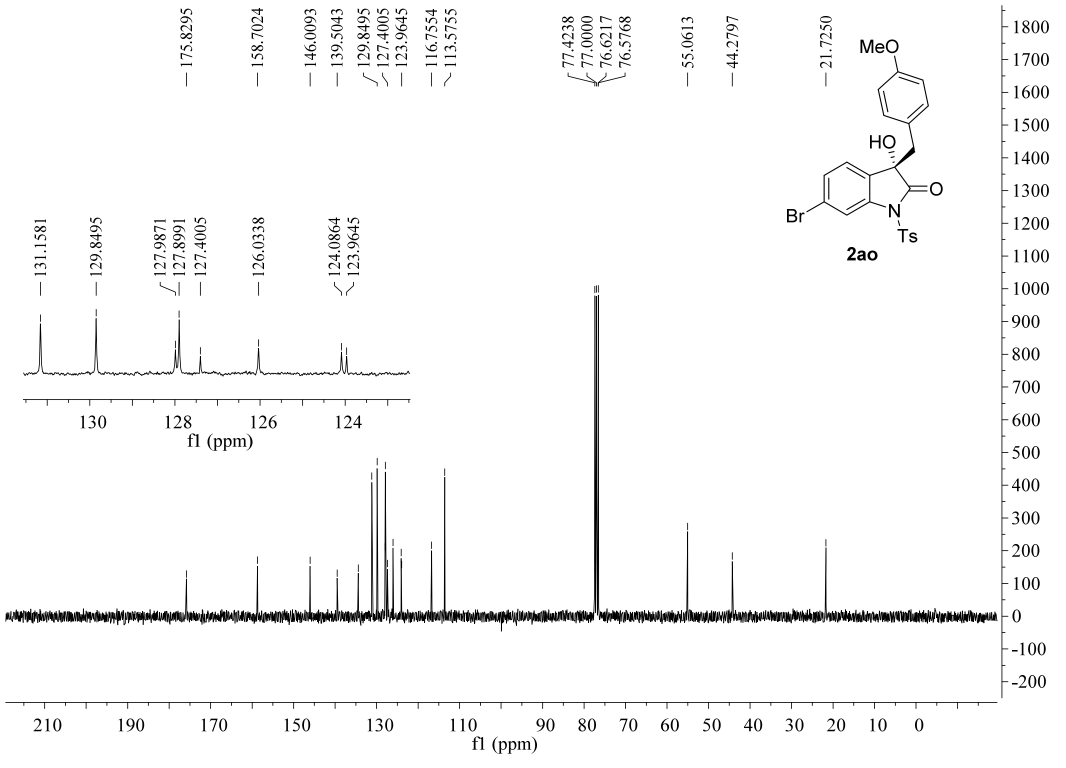

Supplementary Figure 37. ^{1}H and ^{13}C NMR spectra for compound **2ao**

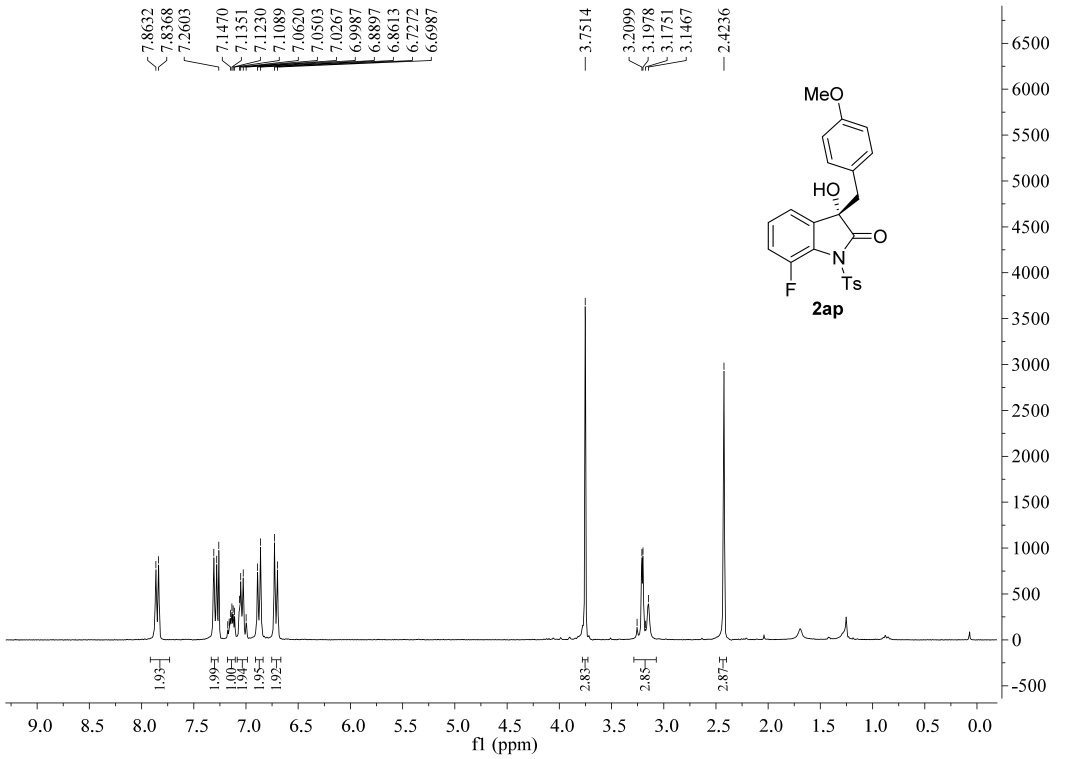

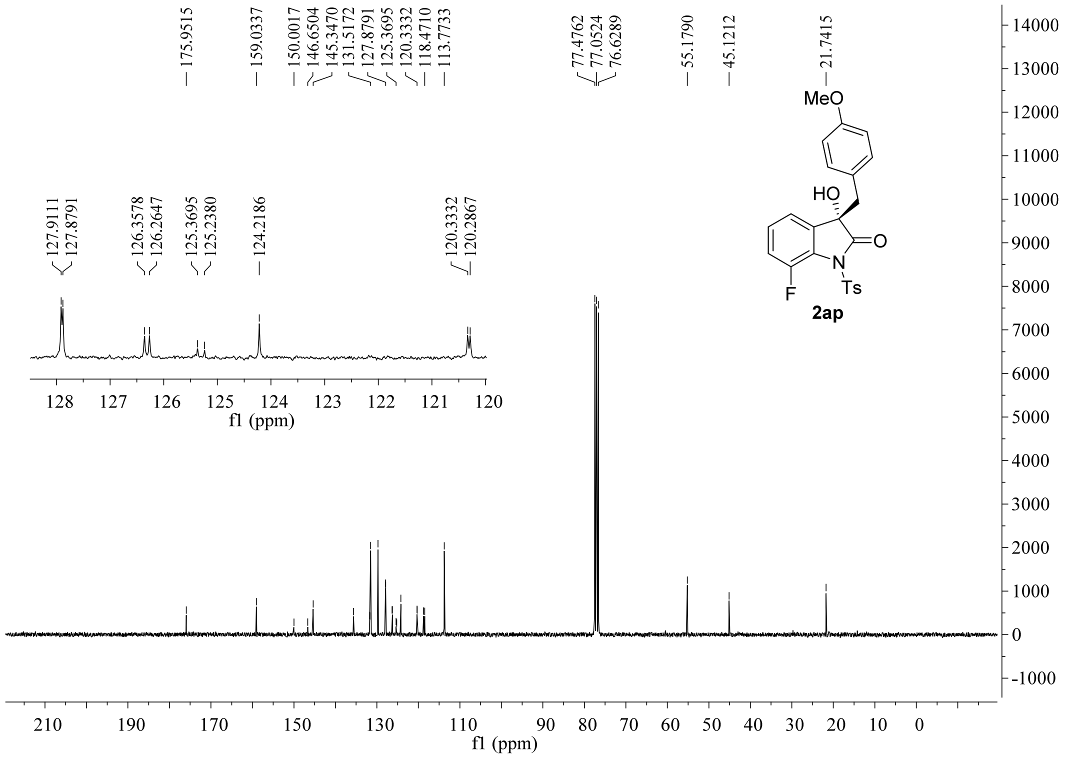

Supplementary Figure 38. ^{1}H and ^{13}C NMR spectra for compound **2ap**

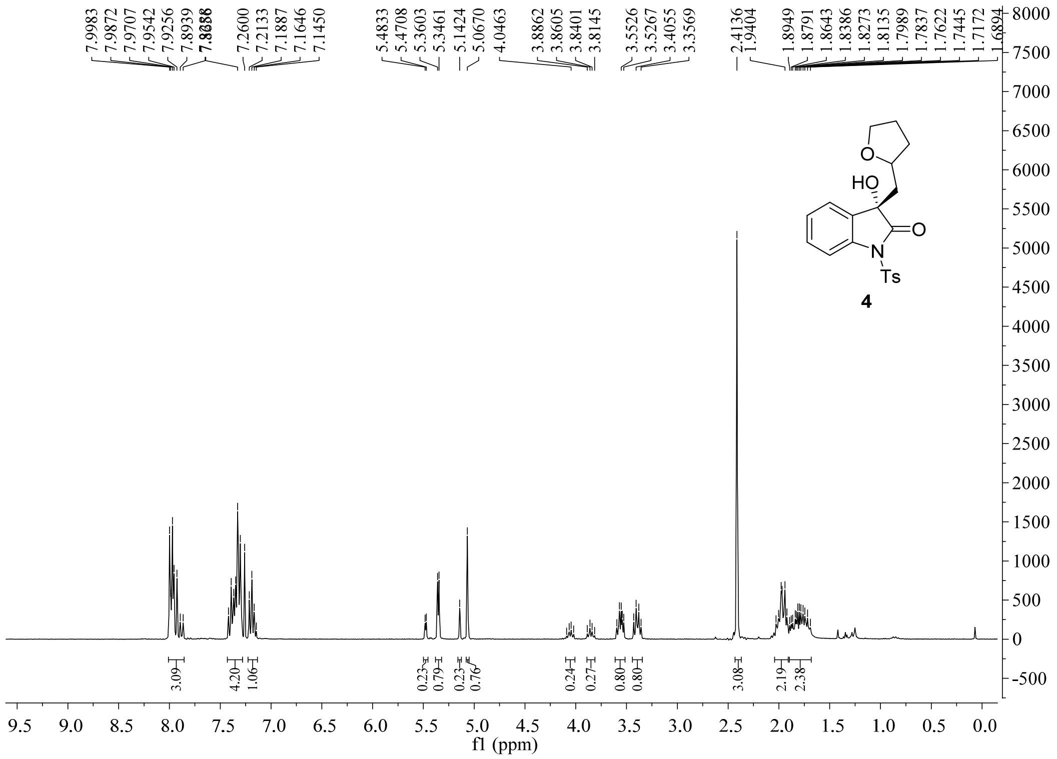

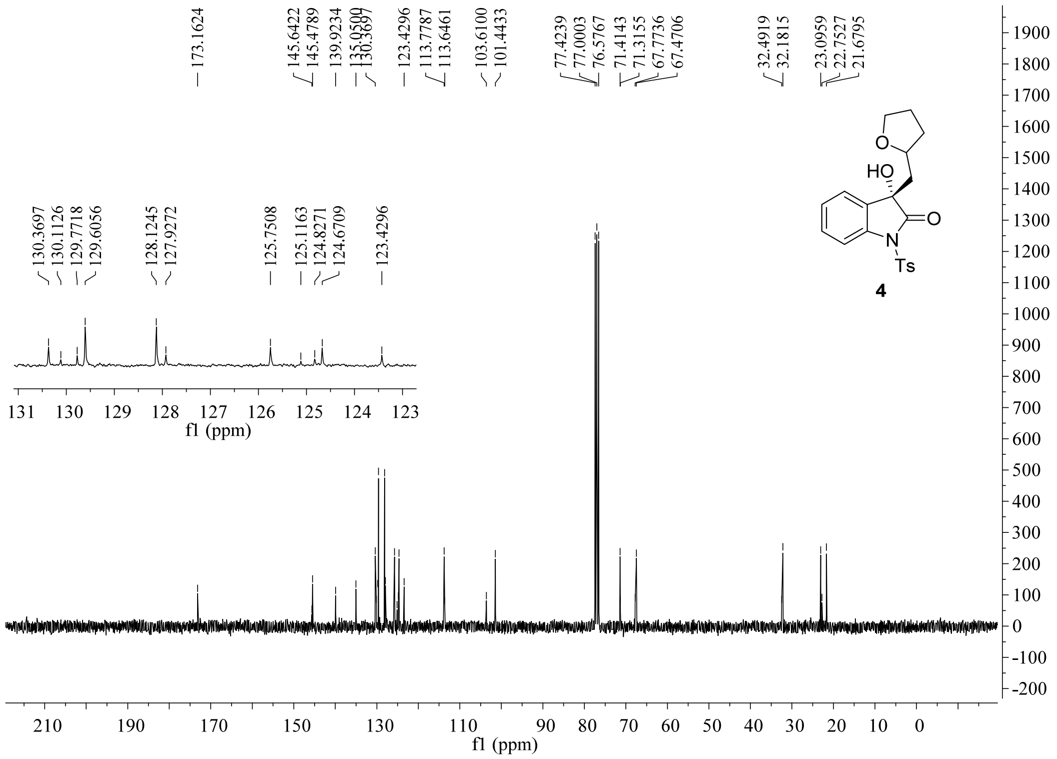

Supplementary Figure 39. ^{1}H and ^{13}C NMR spectra for compound **3**

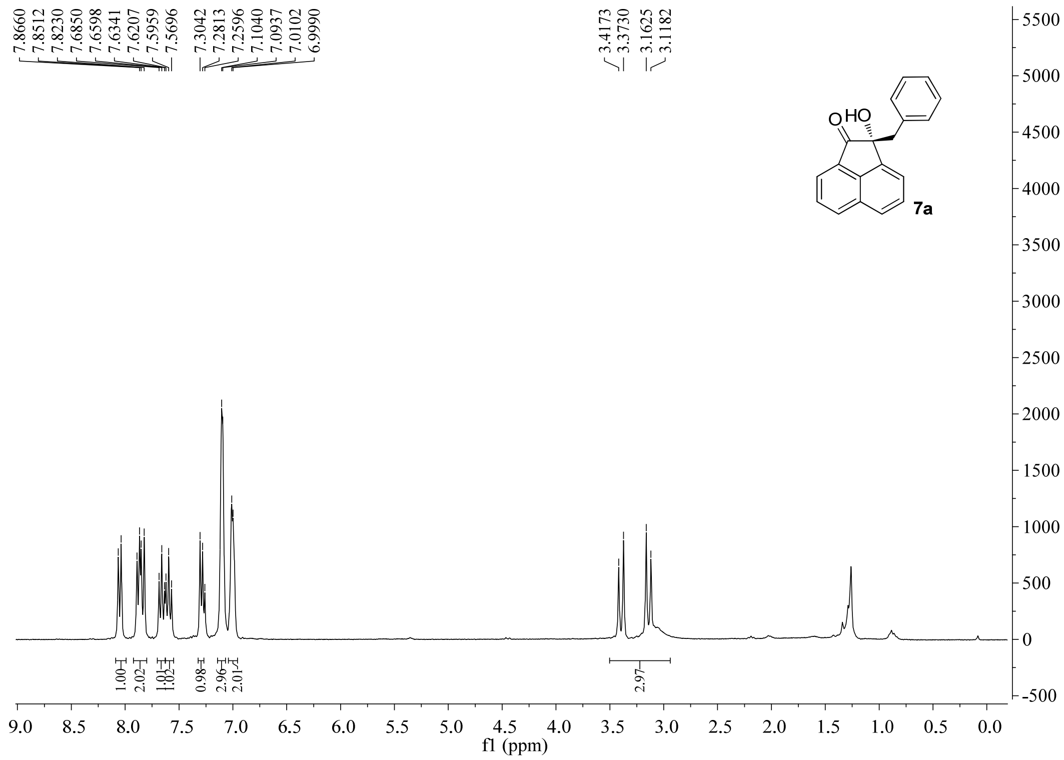

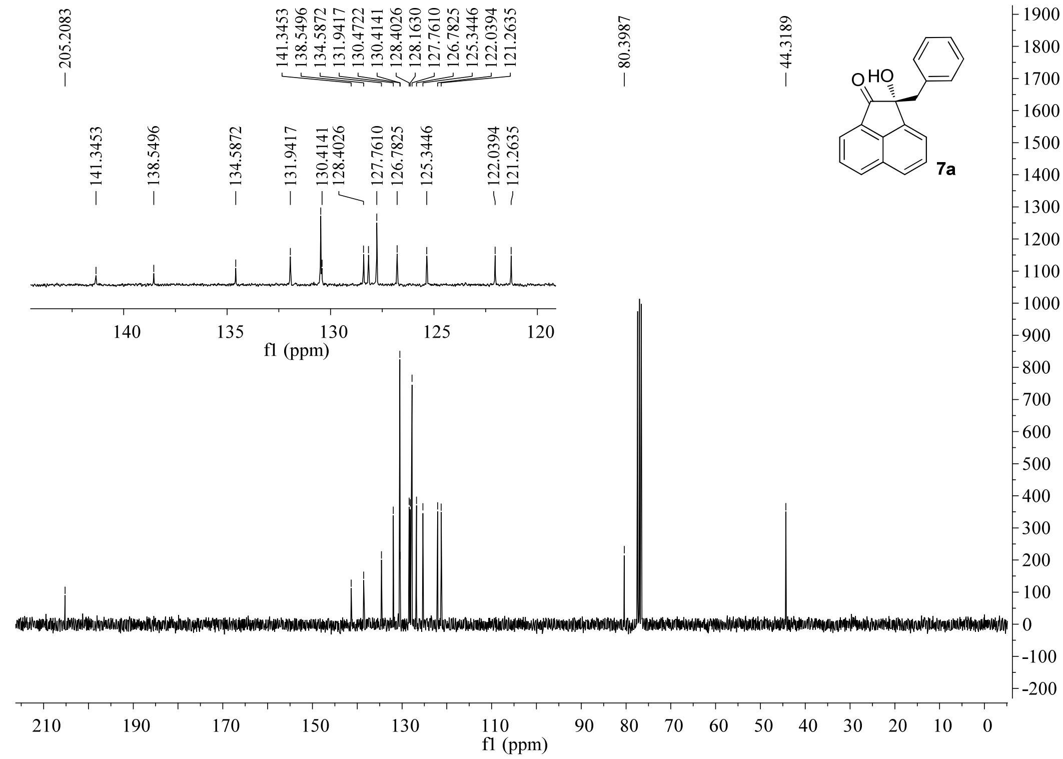

Supplementary Figure 40. ^{1}H and ^{13}C NMR spectra for compound **7a**

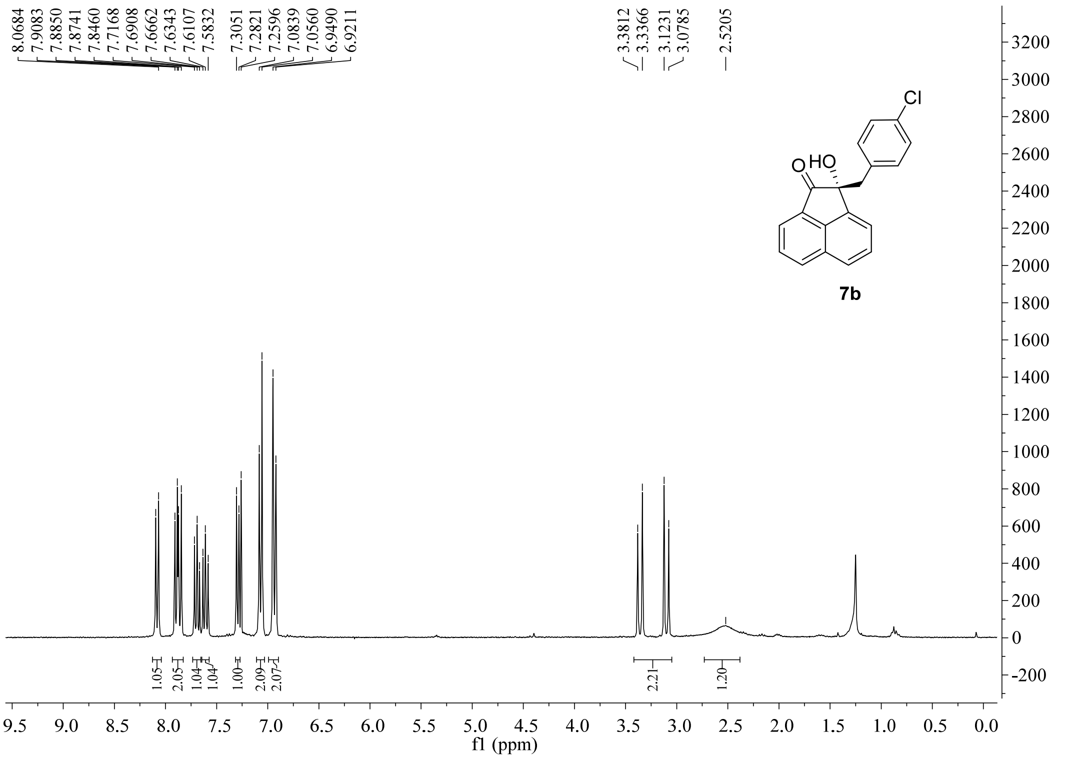

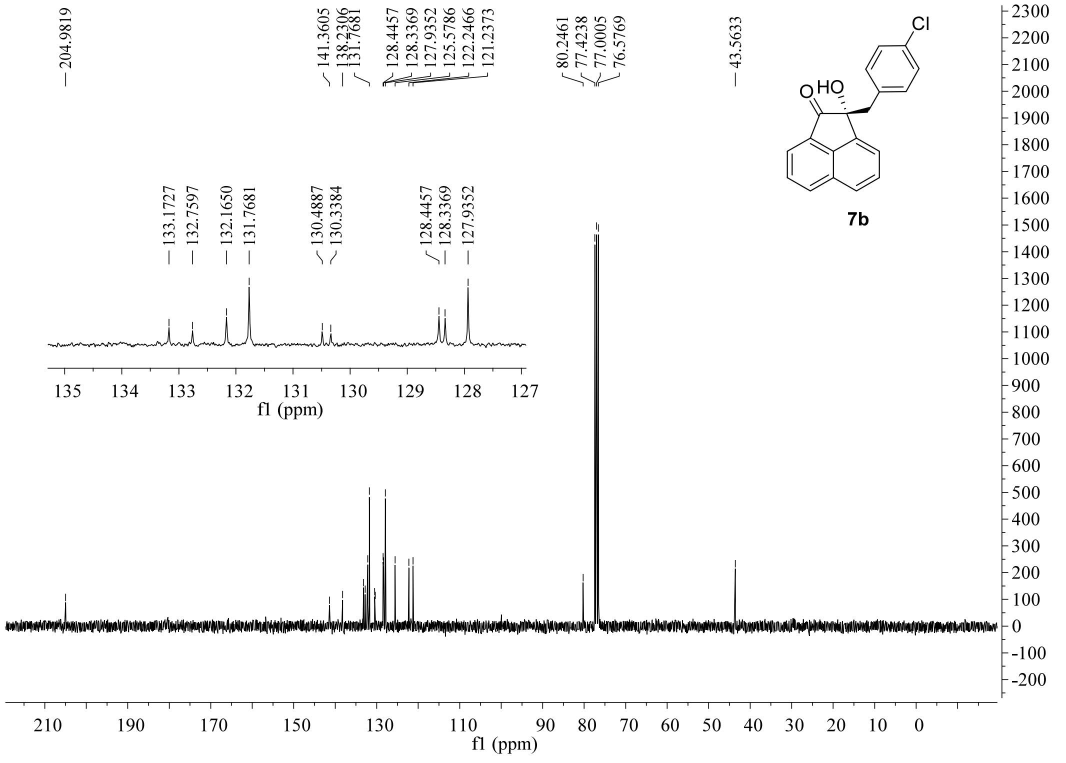

Supplementary Figure 41. ^{1}H and ^{13}C NMR spectra for compound **7b**

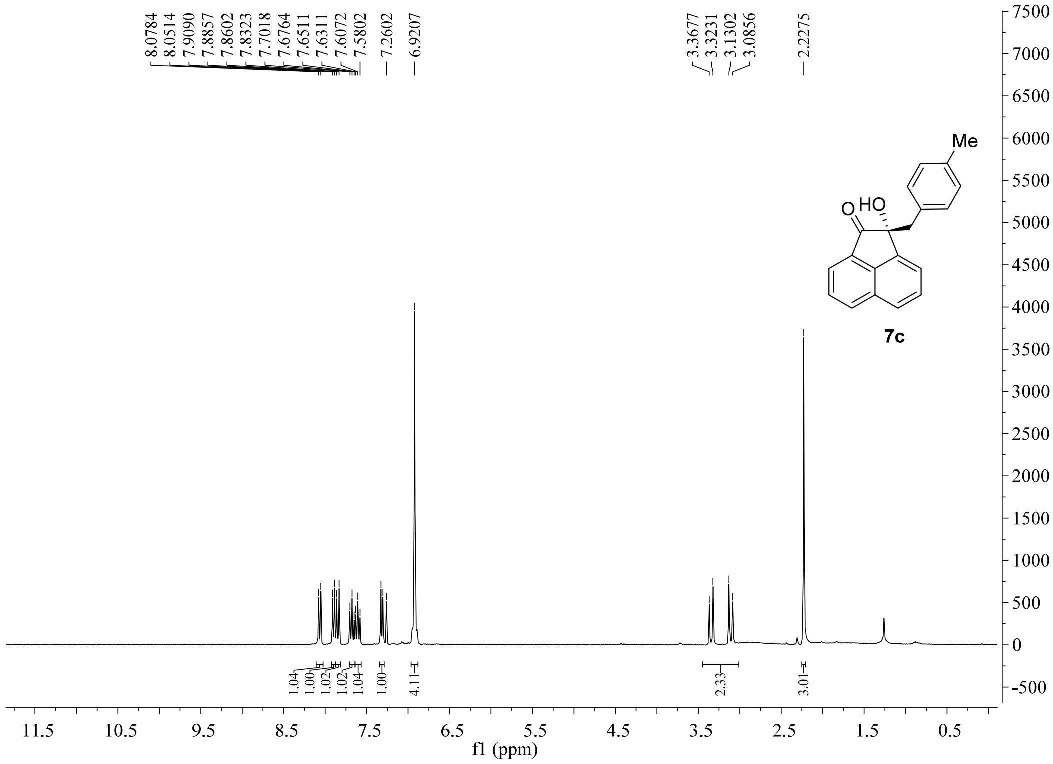

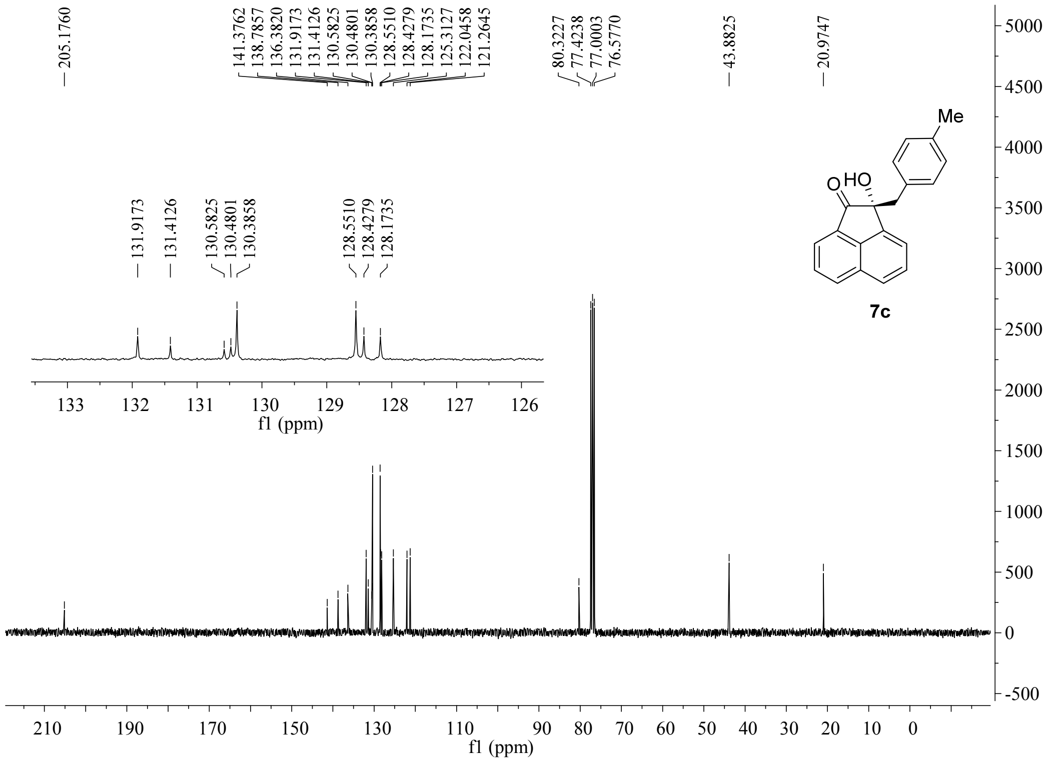

Supplementary Figure 42. ^{1}H and ^{13}C NMR spectra for compound **7c**

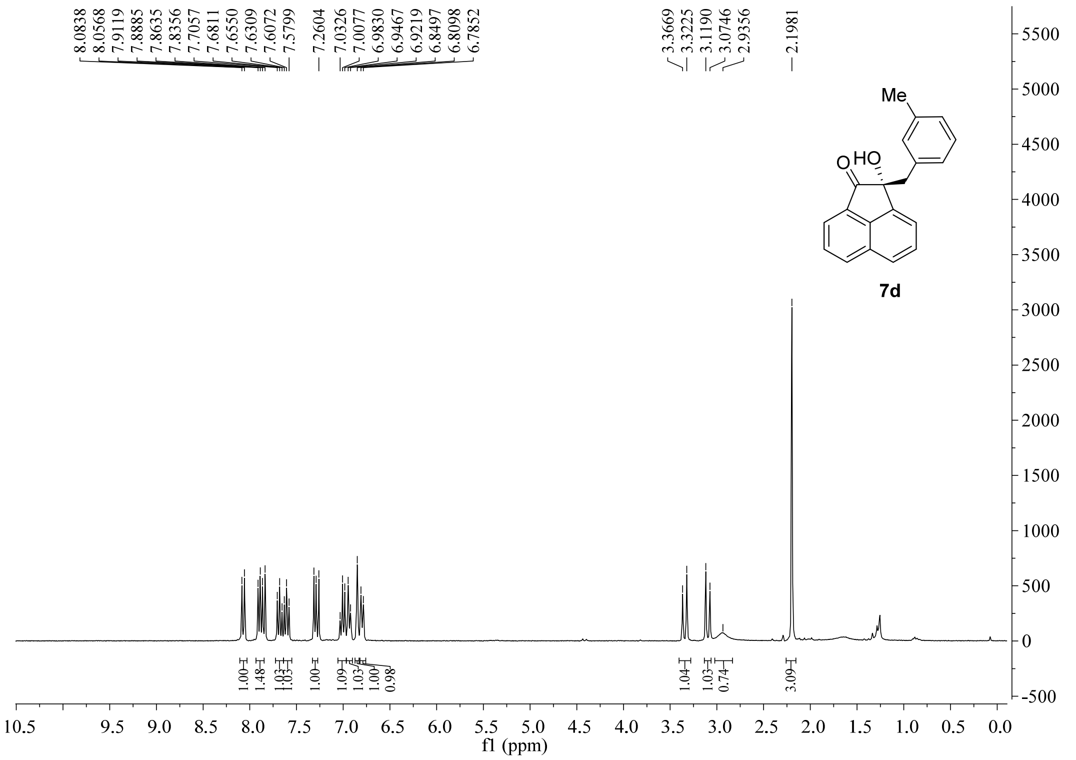

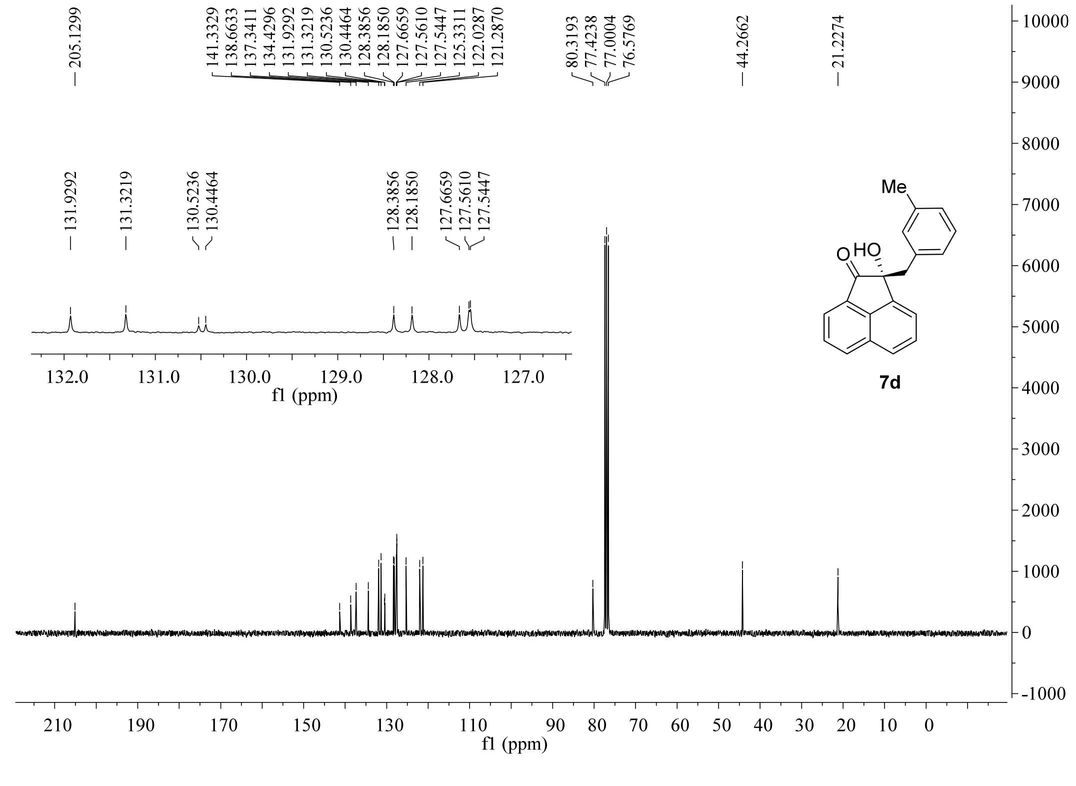

Supplementary Figure 43. ^{1}H and ^{13}C NMR spectra for compound **7d**

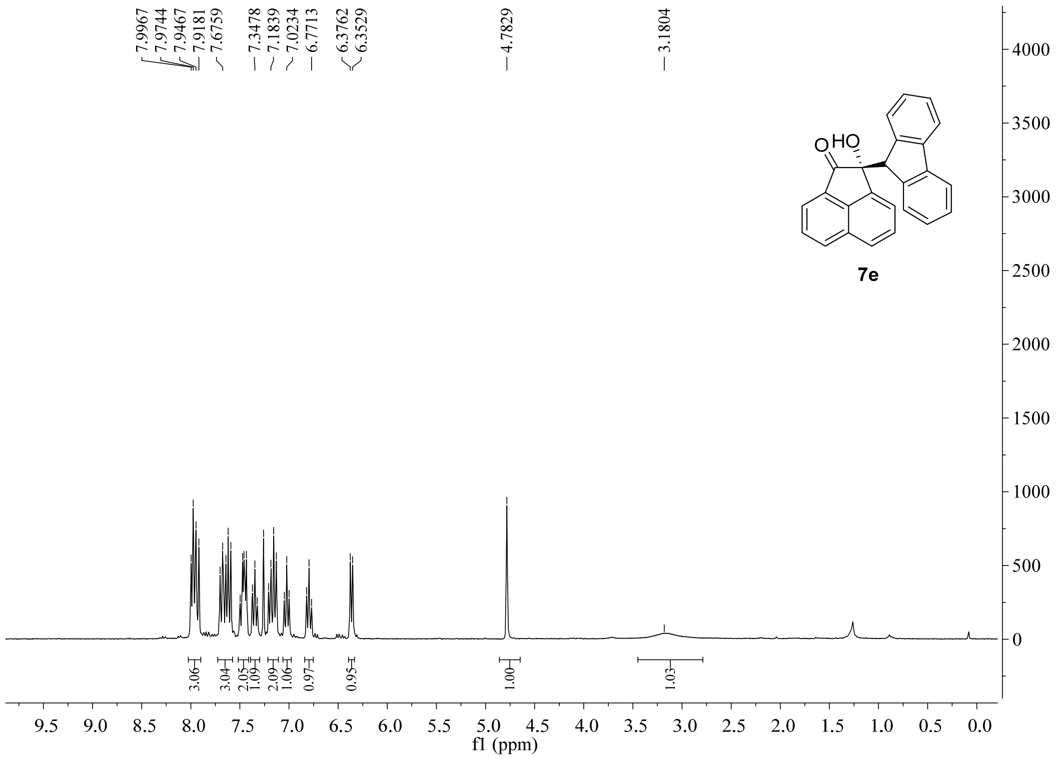

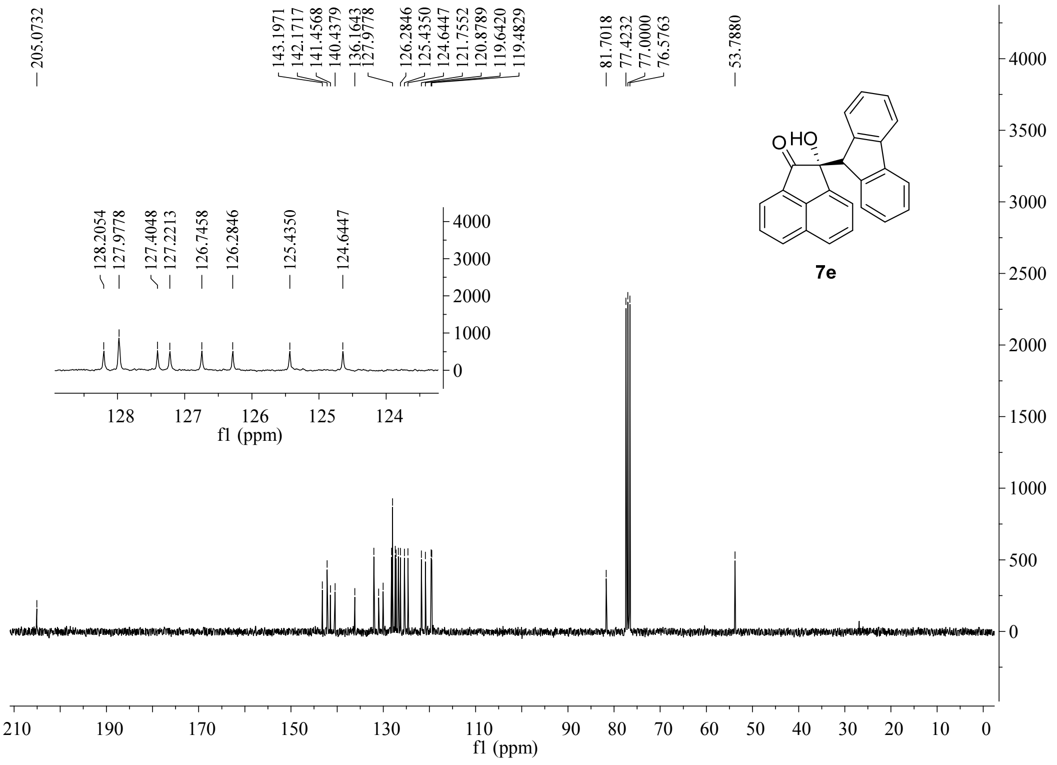

Supplementary Figure 44. ^{1}H and ^{13}C NMR spectra for compound **7e**

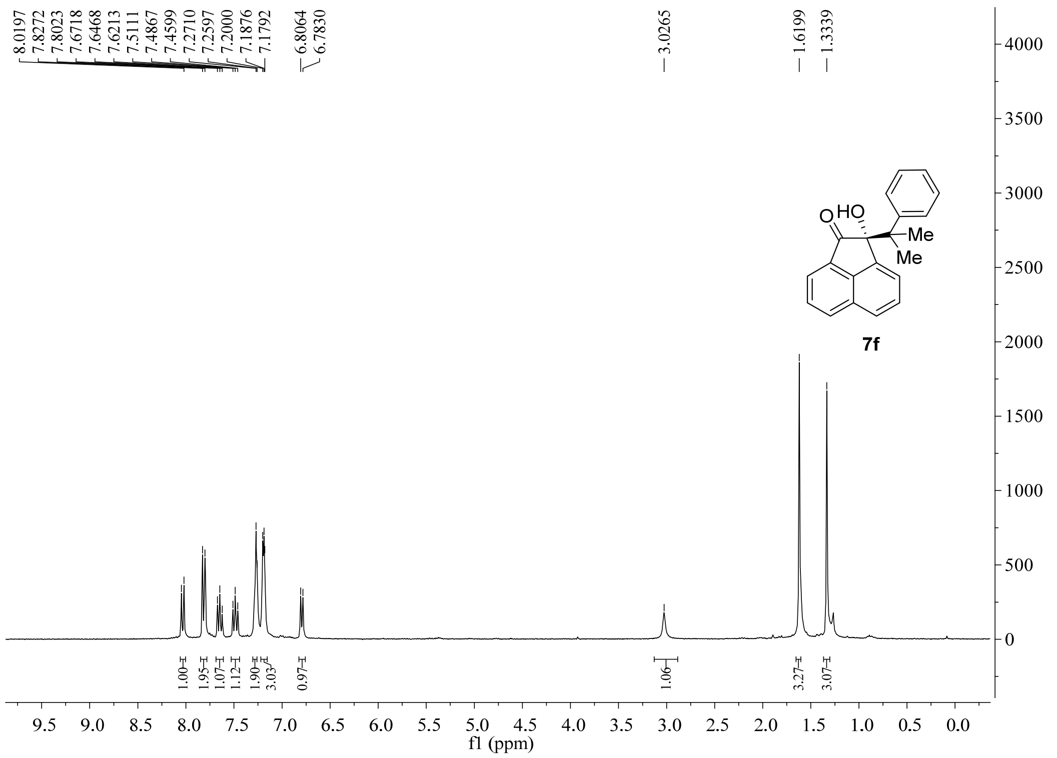

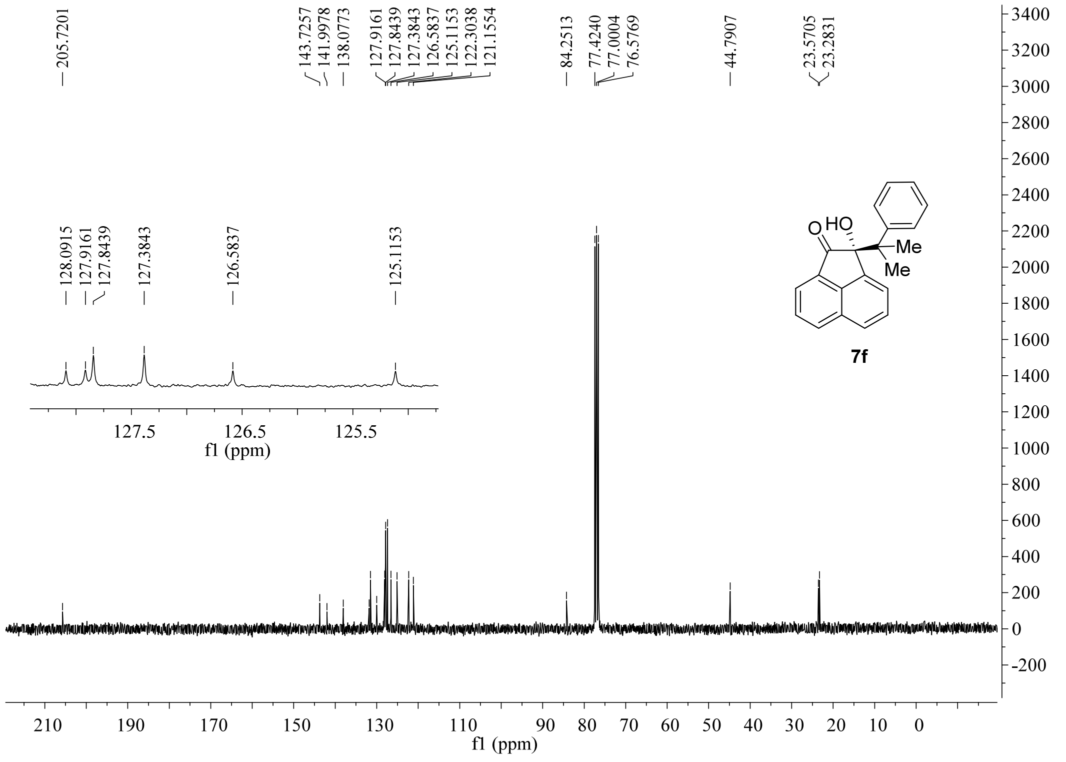

Supplementary Figure 45. ^{1}H and ^{13}C NMR spectra for compound **7f**

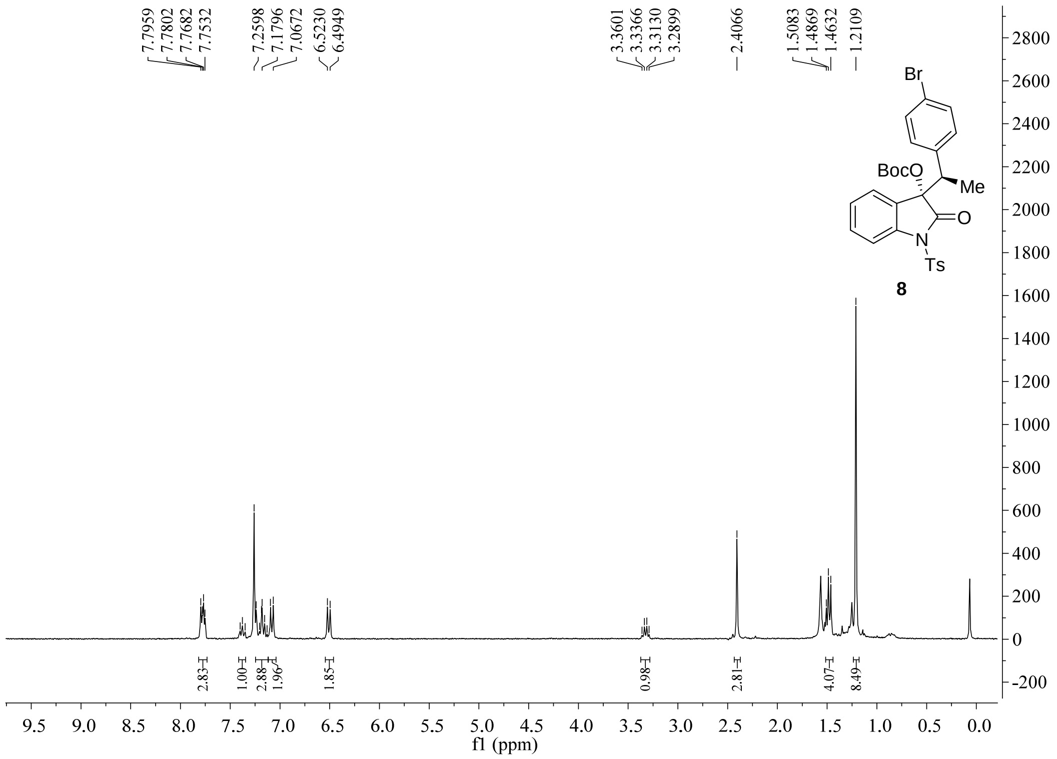

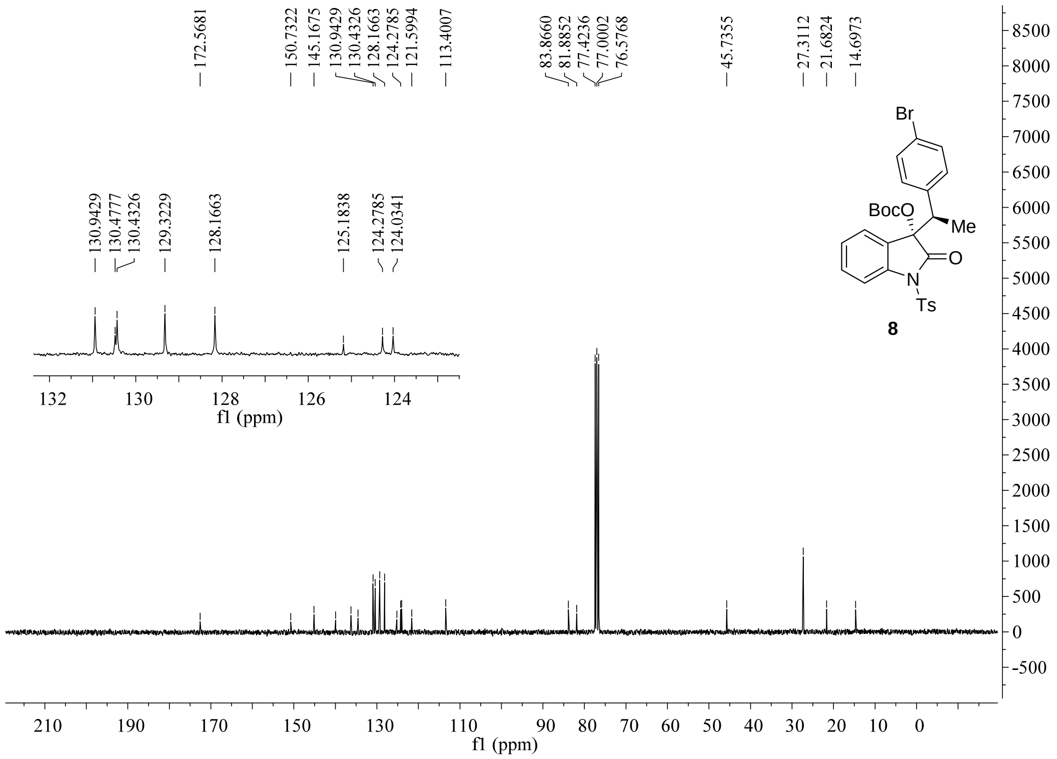

Supplementary Figure 46. ^{1}H and ^{13}C NMR spectra for **8**

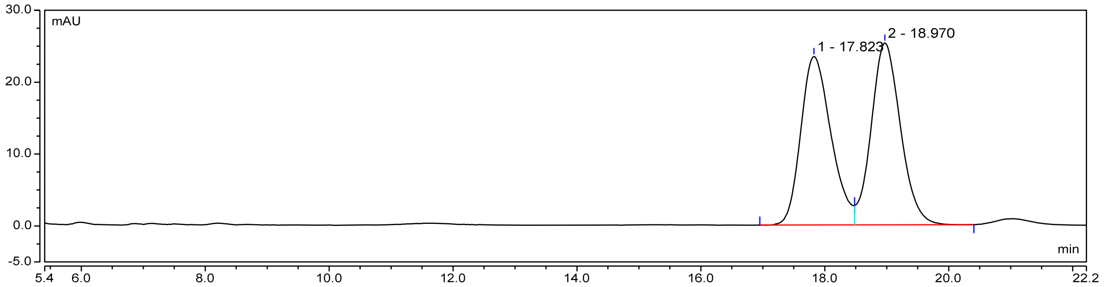

Entry	Retention Time	Area	Height	%Area
1	17.823	13.2484	23.44	48.91
2	18.970	13.8400	25.31	51.09

Racemic **2e**

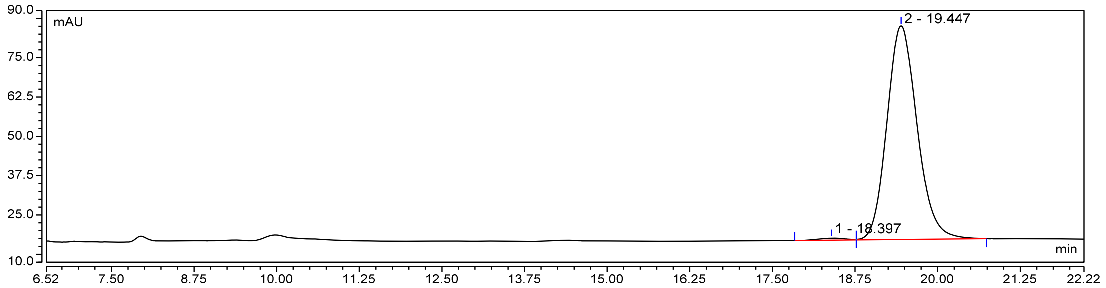

Entry	Retention Time	Area	Height	%Area
1	18.397	0.3086	0.66	0.85
2	19.447	36.0830	67.94	99.15

Enantiomerically enriched **2e** (In PhCl)

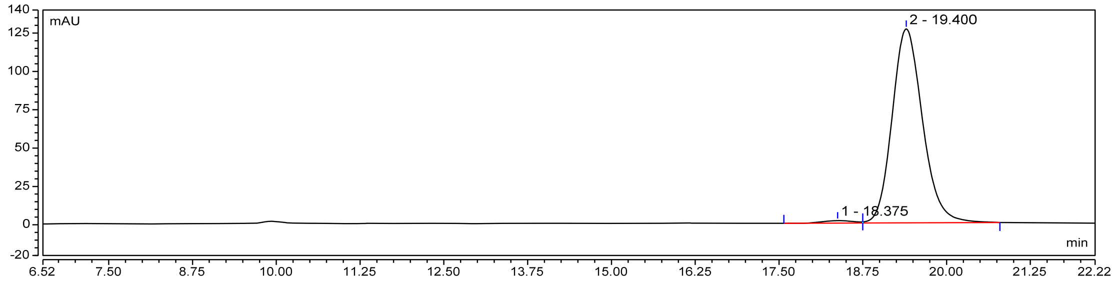

Entry	Retention Time	Area	Height	%Area
1	18.375	0.8585	1.66	1.27
2	19.400	66.6330	126.45	98.73

Enantiomerically enriched **2e** (In neat)

Supplementary Figure 47. HPLC spectra for compound **2e**

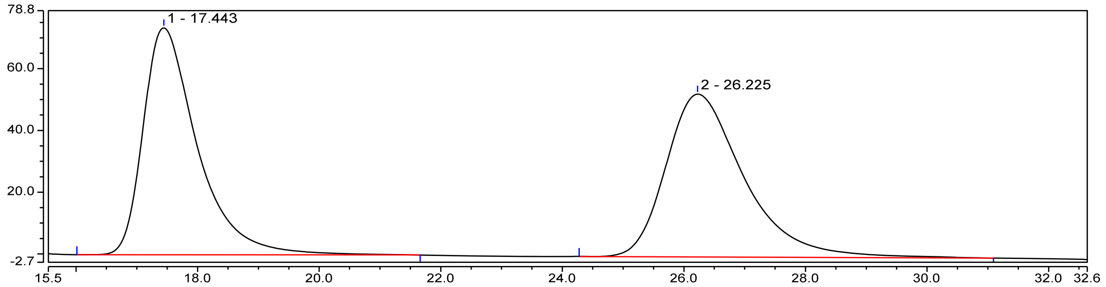

Entry	Retention Time	Area	Height	%Area
1	17.443	75.8832	73.50	49.10
2	26.225	78.6530	52.76	50.90

Racemic **2f**

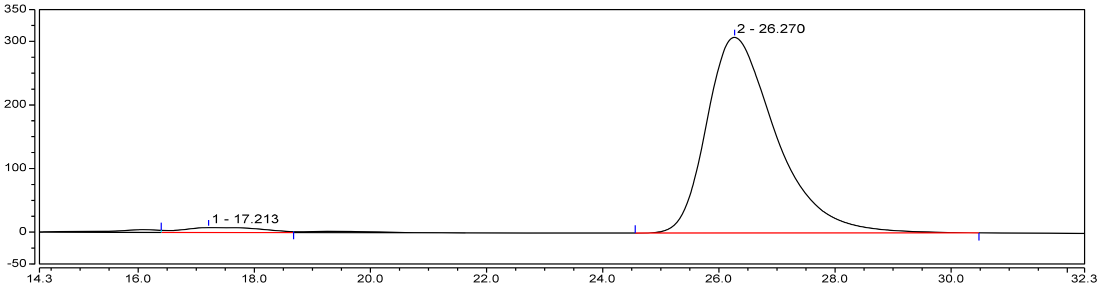

Entry	Retention Time	Area	Height	%Area
1	17.213	12.3041	7.64	2.80
2	26.270	426.6896	307.57	97.20

Enantiomerically enriched **2f** (In PhCl)

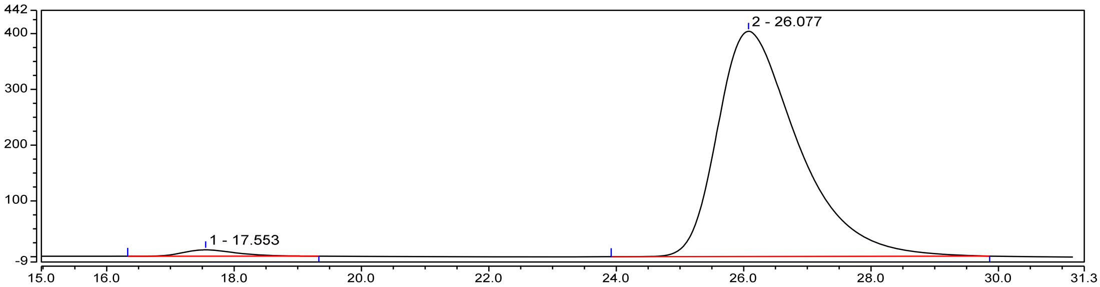

Entry	Retention Time	Area	Height	%Area
1	17.553	10.9505	11.35	1.82
2	26.077	591.8385	403.62	98.18

Enantiomerically enriched **2f** (In neat)

Supplementary Figure 48. HPLC spectra for compound **2f**

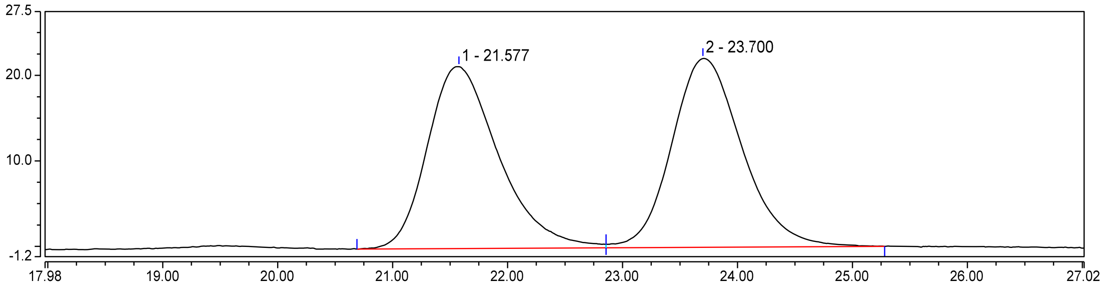

Entry	Retention Time	Area	Height	%Area
1	21.577	15.3372	21.27	49.40
2	23.700	15.7102	22.08	50.60

Racemic **2g**

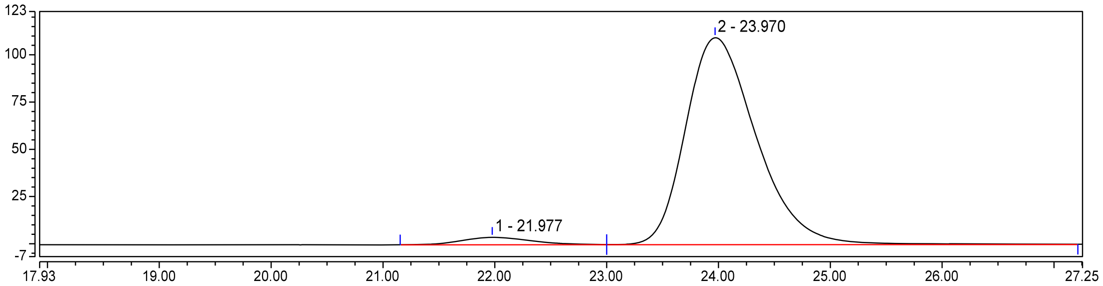

Entry	Retention Time	Area	Height	%Area
1	21.977	2.8888	3.95	3.46
2	23.970	80.5706	109.58	96.54

Enantiomerically enriched **2g** (In PhCl)

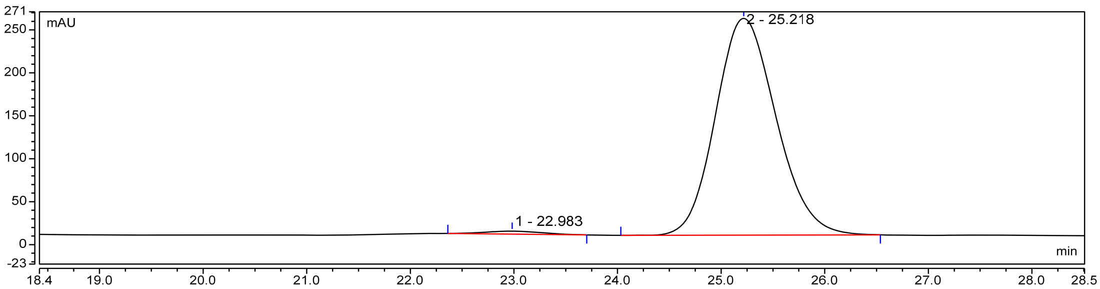

Entry	Retention Time	Area	Height	%Area
1	22.983	2.1776	3.48	1.26
2	25.218	170.8641	252.29	98.74

Enantiomerically enriched **2g** (In neat)

Supplementary Figure 49. HPLC spectra for compound **2g**

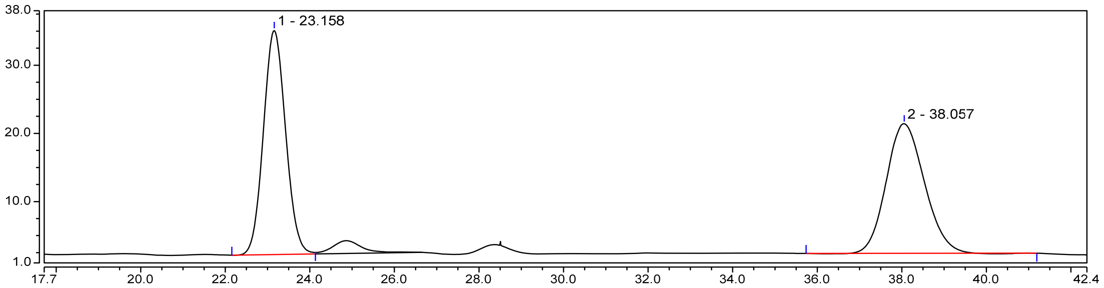

Entry	Retention Time	Area	Height	%Area
1	23.158	20.0202	32.88	50.23
2	38.057	19.8395	19.03	49.77

Racemic **2h**

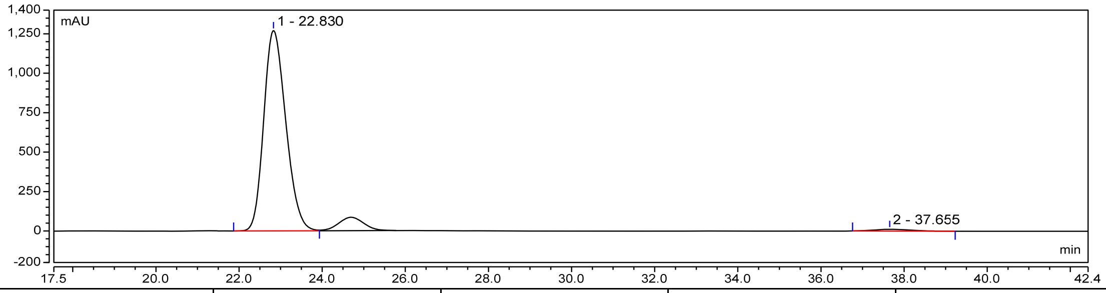

Entry	Retention Time	Area	Height	%Area
1	22.830	780.4875	1270.94	98.50
2	37.655	11.8896	11.51	1.50

Enantiomerically enriched **2h** (In PhCl)

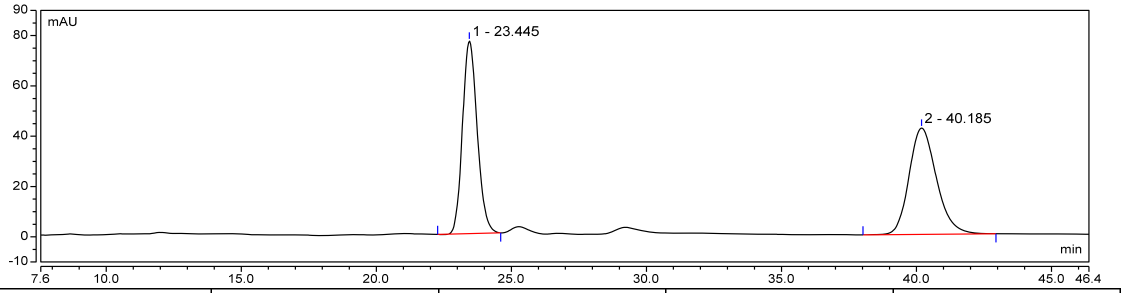

Entry	Retention Time	Area	Height	%Area
1	23.445	48.4732	76.58	49.40
2	40.185	49.6534	42.25	50.60

Racemic **2h**

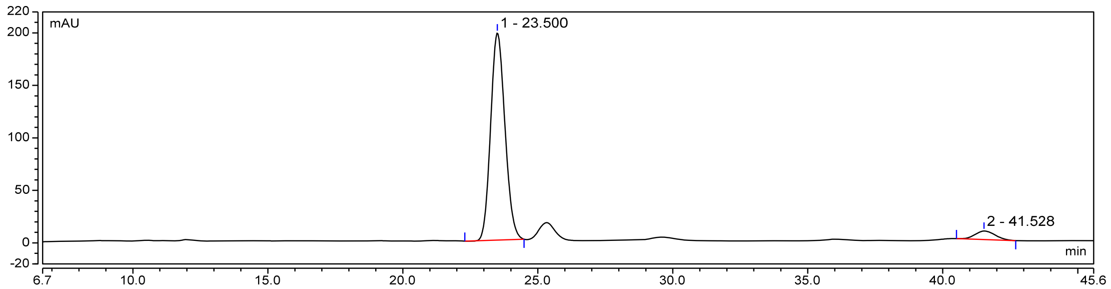

Entry	Retention Time	Area	Height	%Area
1	23.500	121.4695	197.39	94.55
2	41.528	7.0051	8.09	5.45

Enantiomerically enriched **2h** (In neat)

Supplementary Figure 50. HPLC spectra for compound **2h**

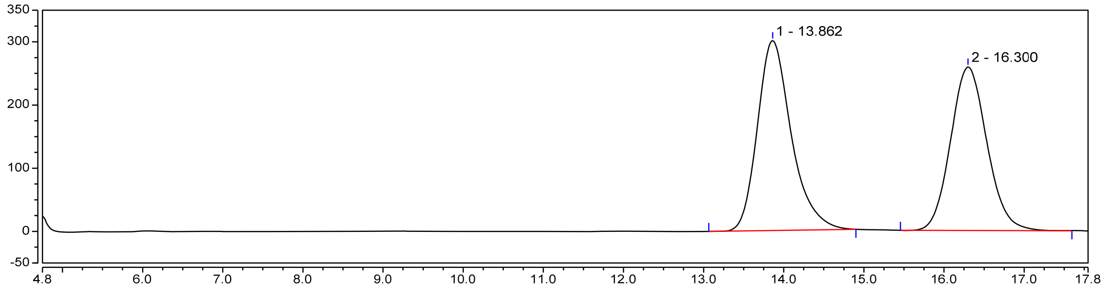

Entry	Retention Time	Area	Height	%Area
1	13.862	142.4142	297.57	51.69
2	16.300	133.0816	257.06	48.31

Racemic **2i**

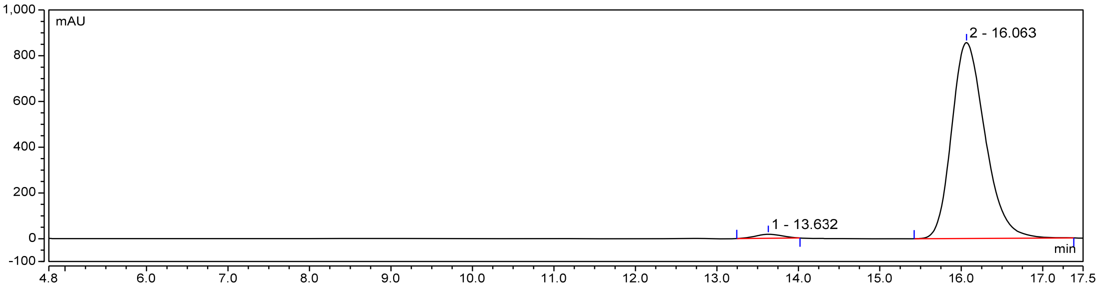

Entry	Retention Time	Area	Height	%Area
1	13.632	6.5658	17.40	1.59
2	16.063	407.4129	856.48	98.41

Enantiomerically enriched **2i** (In PhCl)

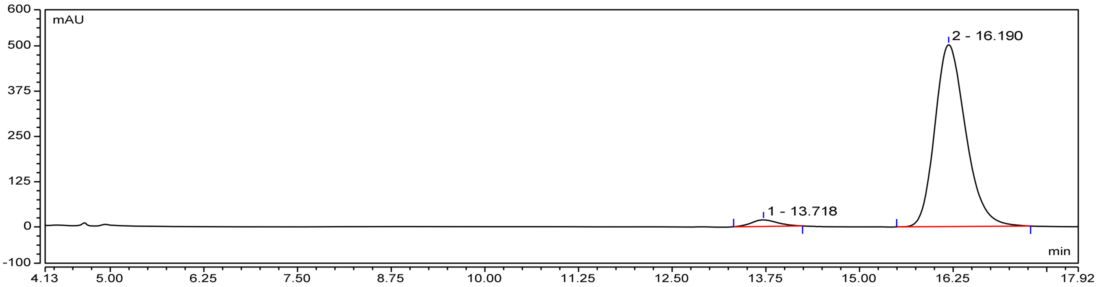

Entry	Retention Time	Area	Height	%Area
1	13.718	7.3538	18.04	2.98
2	16.190	239.7987	502.45	97.02

Enantiomerically enriched **2i** (In neat)

Supplementary Figure 51. HPLC spectra for compound **2i**

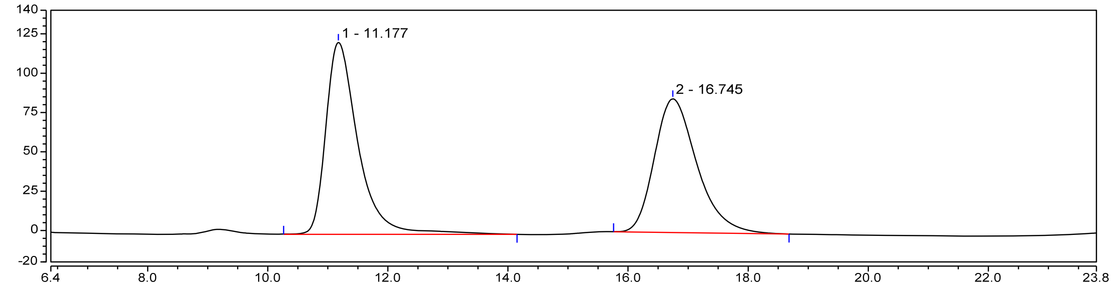

Entry	Retention Time	Area	Height	%Area
1	11.177	75.9813	121.95	52.09
2	16.745	69.8704	84.92	47.91

Racemic **2j**

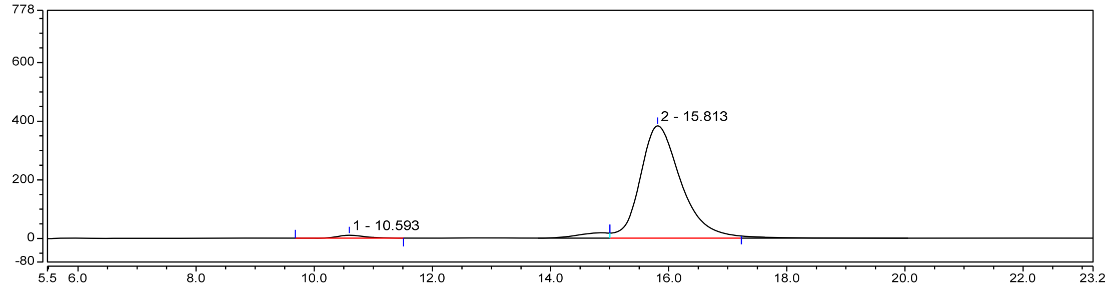

Entry	Retention Time	Area	Height	%Area
1	10.593	5.2819	9.91	1.71
2	15.813	304.3871	382.56	98.29

Enantiomerically enriched **2j** (In PhCl)

Supplementary Figure 52. HPLC spectra for compound **2j**

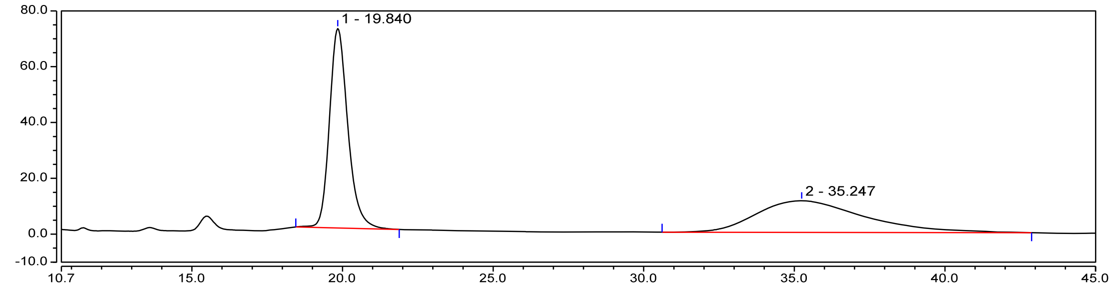

Entry	Retention Time	Area	Height	%Area
1	19.840	50.6503	71.47	51.82
2	35.247	47.0923	11.35	48.18

Racemic **2k**

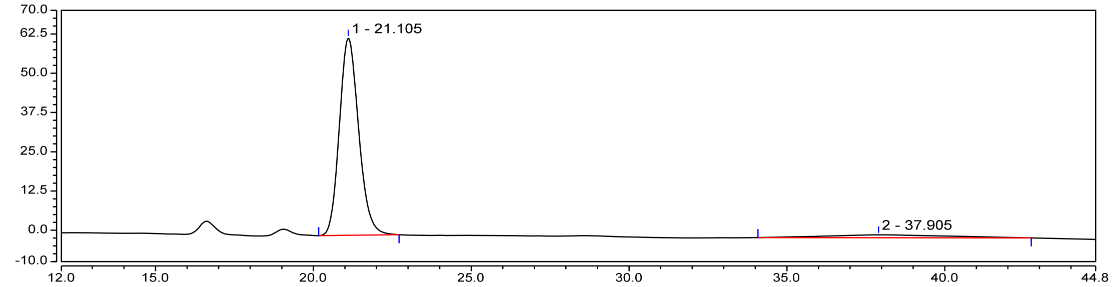

Entry	Retention Time	Area	Height	%Area

1	21.105	44.6252	62.80	91.41
2	37.905	4.1933	0.93	8.59

Enantiomerically enriched **2k** (In PhCl)

Supplementary Figure 53. HPLC spectra for compound **2k**

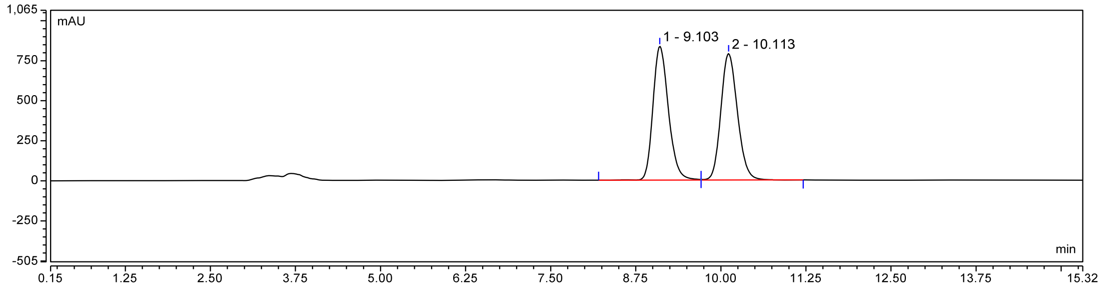

Entry	Retention Time	Area	Height	%Area
1	9.103	221.1542	835.27	49.76
2	10.113	223.3132	789.30	50.24

Racemic **2l**

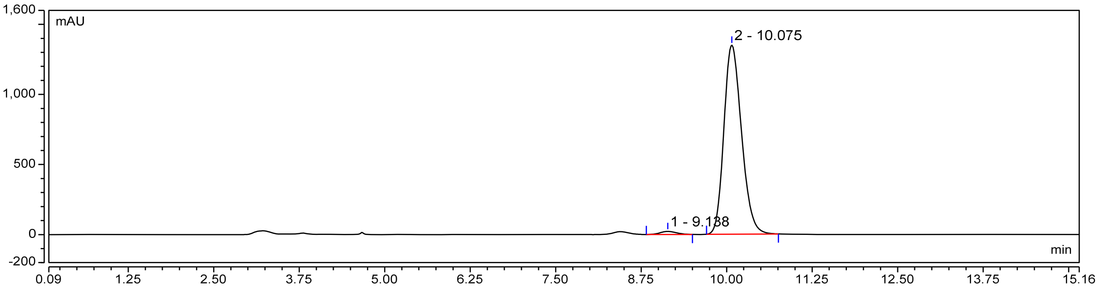

Entry	Retention Time	Area	Height	%Area
1	9.138	5.9045	21.72	1.44
2	10.075	403.2286	1349.43	98.56

Enantiomerically enriched **2l** (In PhCl)

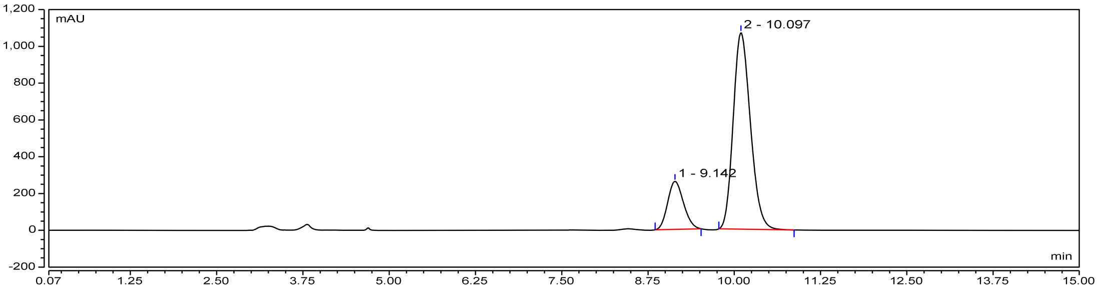

Entry	Retention Time	Area	Height	%Area
1	9.142	69.4602	260.90	18.37
2	10.097	308.6689	1068.15	81.63

Enantiomerically enriched **2l** (In neat)

Supplementary Figure 54. HPLC spectra for compound **2l**

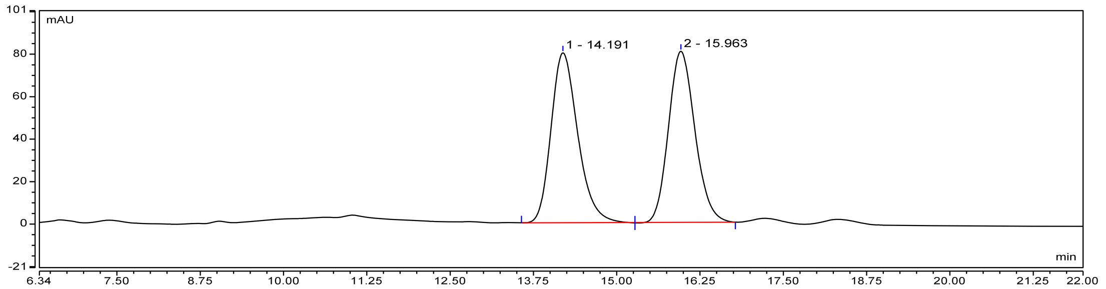

Entry	Retention Time	Area	Height	%Area
1	14.191	37.1334	79.96	50.09
2	15.963	37.0025	80.53	49.91

Racemic **2m**

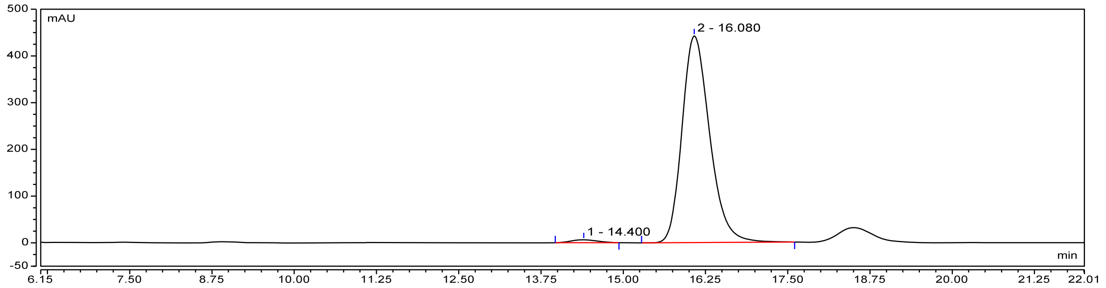

Entry	Retention Time	Area	Height	%Area
1	14.400	2.8188	6.29	1.29
2	16.080	215.6133	442.23	98.71

Enantiomerically enriched **2m** (In PhCl)

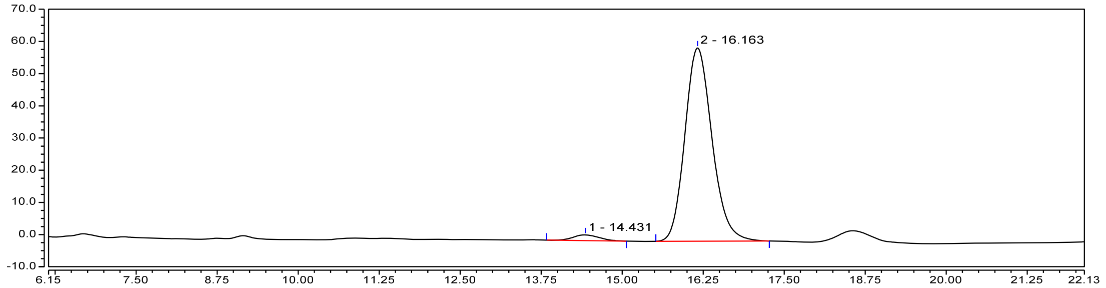

Entry	Retention Time	Area	Height	%Area
1	14.431	0.8147	1.79	2.71
2	16.163	29.2065	60.05	97.29

Enantiomerically enriched **2m** (In neat)

Supplementary Figure 55. HPLC spectra for compound **2m**

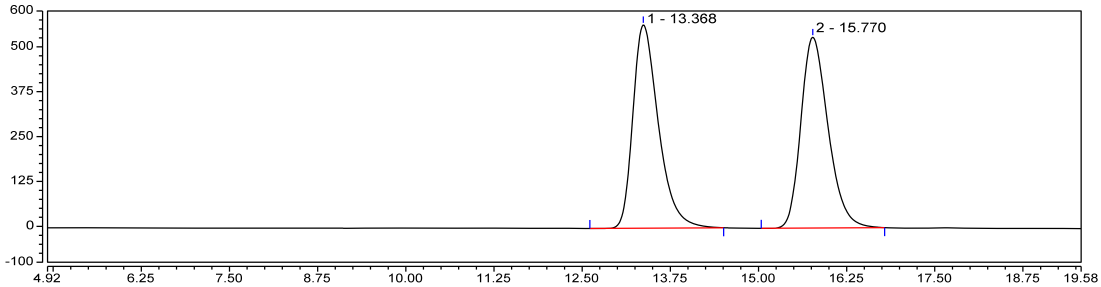

Entry	Retention Time	Area	Height	%Area
1	13.368	237.7969	566.27	50.00
2	15.770	237.7920	531.43	50.00

Racemic **2n**

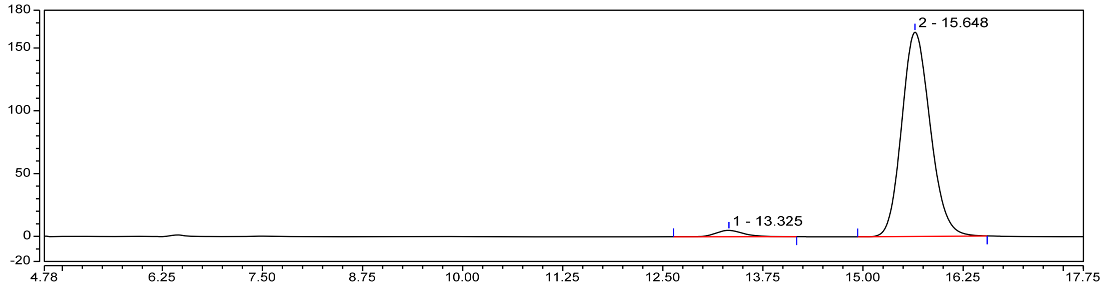

Entry	Retention Time	Area	Height	%Area
1	13.325	1.9115	4.96	2.76
2	15.648	67.2548	162.62	97.24

Enantiomerically enriched **2n** (In PhCl)

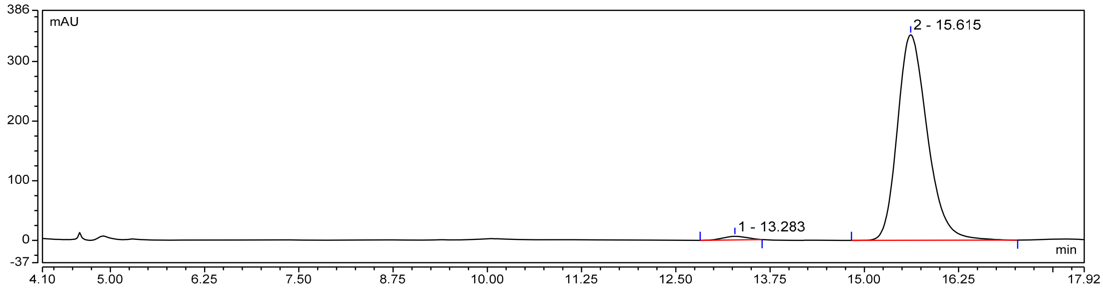

Entry	Retention Time	Area	Height	%Area
1	13.283	2.2147	6.02	1.38
2	15.615	158.5665	344.59	98.62

Enantiomerically enriched **2n** (In neat)

Supplementary Figure 56. HPLC spectra for compound **2n**

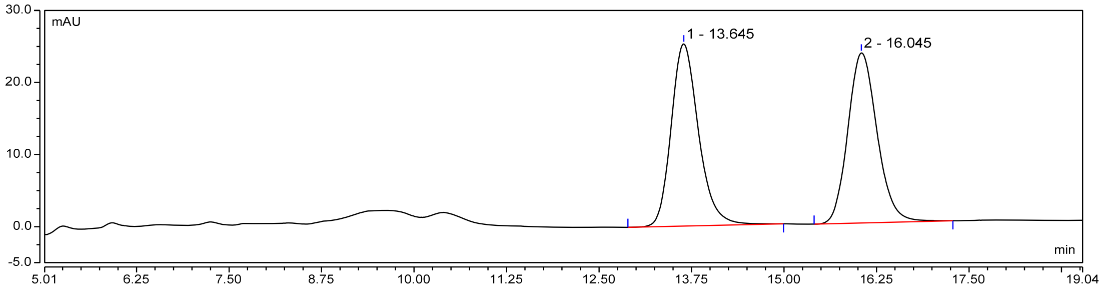

Entry	Retention Time	Area	Height	%Area
1	13.645	10.8525	25.28	50.11
2	16.045	10.8068	23.59	49.89

Racemic **2o**

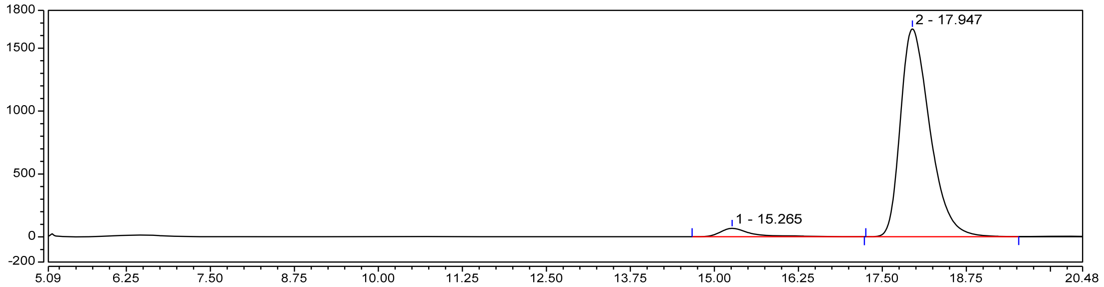

Entry	Retention Time	Area	Height	%Area
1	15.265	36.1024	66.28	4.11

2	17.947	841.2463	1651.33	95.89

Enantiomerically enriched **2o** (In PhCl)

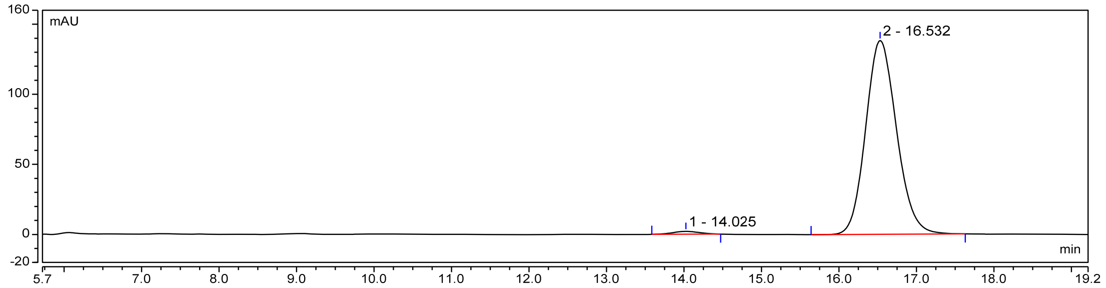

Entry	Retention Time	Area	Height	%Area
1	14.025	0.8166	2.06	1.26
2	16.532	63.8678	138.25	98.74

Enantiomerically enriched **2o** (In neat)

Supplementary Figure 57. HPLC spectra for compound **2o**

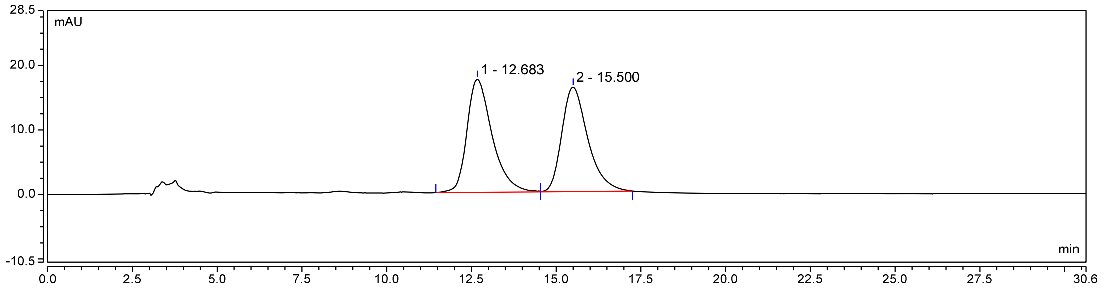

Entry	Retention Time	Area	Height	%Area
1	12.683	15.1535	17.53	51.23
2	15.500	14.4277	16.19	48.77

Racemic **2p**

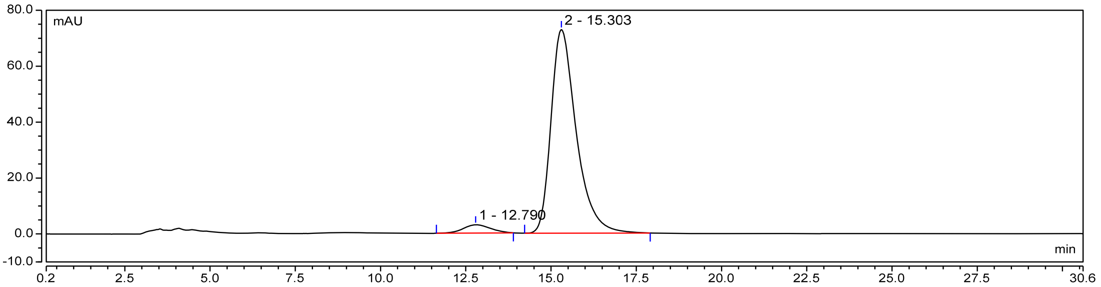

Entry	Retention Time	Area	Height	%Area
1	12.790	2.7380	2.98	4.19
2	15.303	62.5742	72.85	95.81

Enantiomerically enriched **2p** (In PhCl)

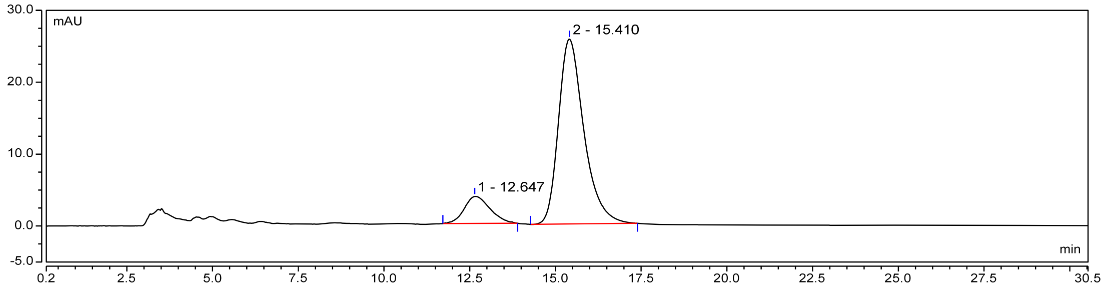

Entry	Retention Time	Area	Height	%Area

1	12.647	3.2782	3.77	12.91
2	15.410	22.1186	25.73	87.09

Enantiomerically enriched **2p** (In neat)

Supplementary Figure 58. HPLC spectra for compound **2p**

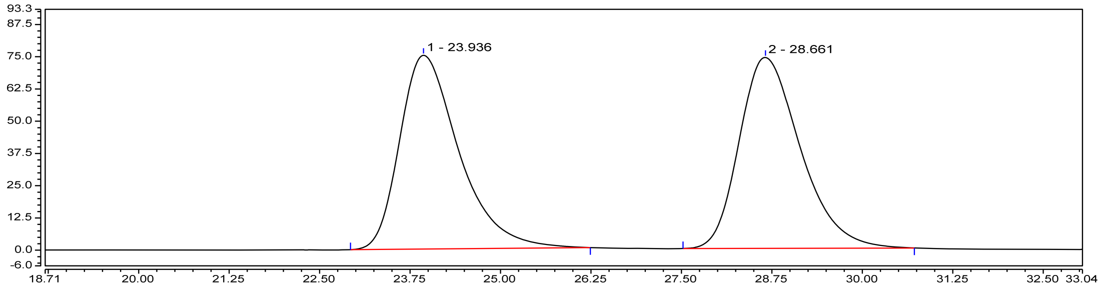

Entry	Retention Time	Area	Height	%Area
1	23.936	71.4305	75.09	49.59
2	28.661	72.6238	74.02	50.41

Racemic **2q**

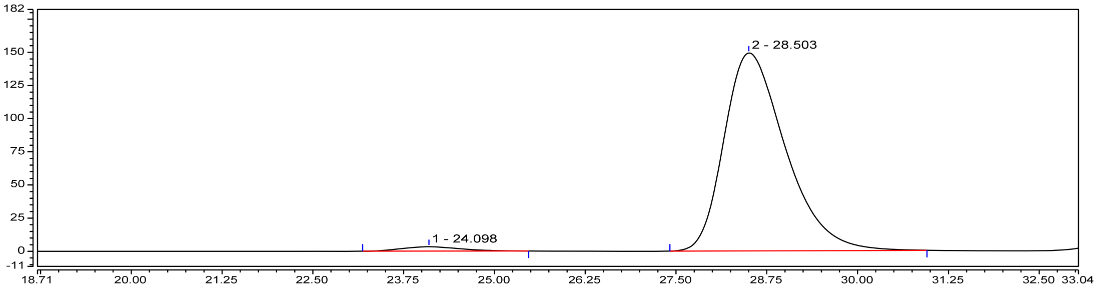

Entry	Retention Time	Area	Height	%Area
1	24.098	3.0827	3.32	2.06
2	28.503	146.4014	149.17	97.94

Enantiomerically enriched **2q** (In PhCl)

Supplementary Figure 59. HPLC spectra for compound **2q**

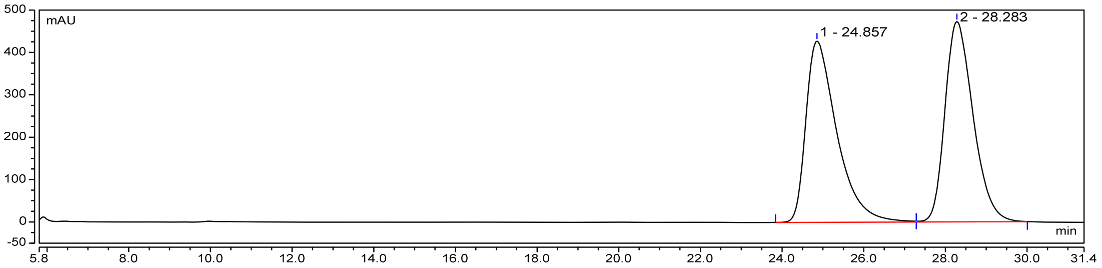

Entry	Retention Time	Area	Height	%Area
1	24.857	375.0520	427.31	49.84
2	28.283	377.4787	472.37	50.16

Racemic **2r**

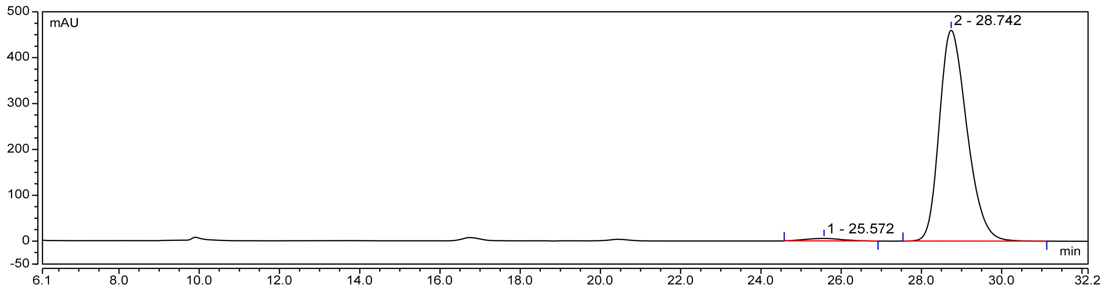

Entry	Retention Time	Area	Height	%Area
1	25.572	5.8429	5.52	1.57
2	28.742	366.3694	459.63	98.43

Enantiomerically enriched **2r** (In PhCl)

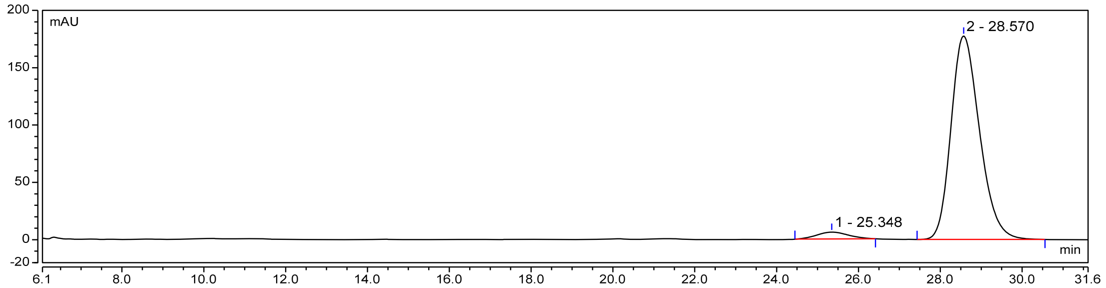

Entry	Retention Time	Area	Height	%Area
1	25.348	5.4647	6.02	3.67
2	28.570	143.5460	177.48	96.33

Enantiomerically enriched **2r** (In neat)

Supplementary Figure 60. HPLC spectra for compound **2r**

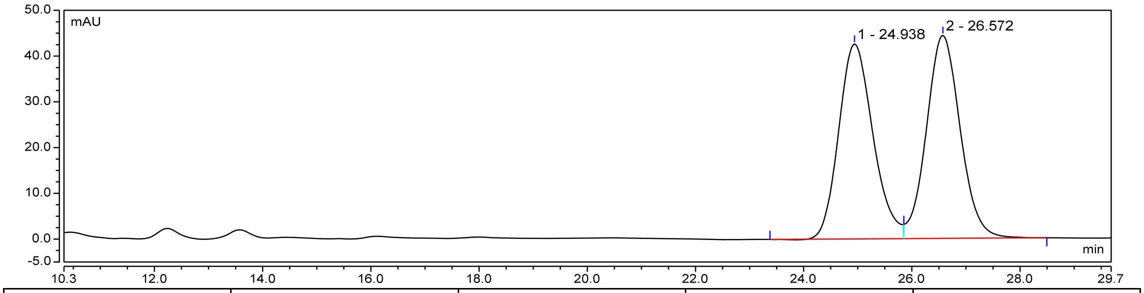

Entry	Retention Time	Area	Height	%Area
1	24.972	28.0358	38.17	49.56
2	26.663	28.5306	39.42	50.44

Racemic **2s**

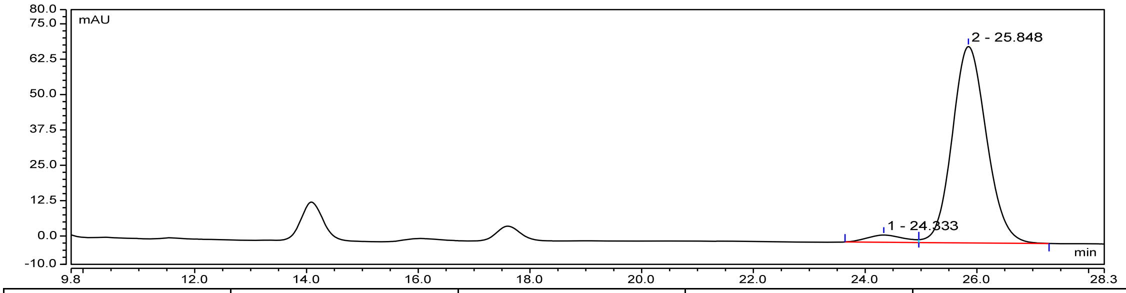

Entry	Retention Time	Area	Height	%Area
1	24.333	1.9406	2.56	3.77
2	25.848	49.4707	69.41	96.23

Enantiomerically enriched **2s** (In PhCl)

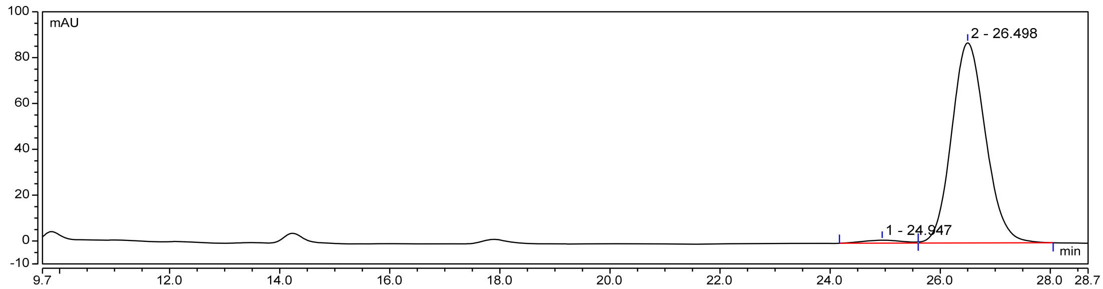

Entry	Retention Time	Area	Height	%Area
1	24.947	1.0190	1.33	1.61
2	26.498	62.2115	87.25	98.39

Enantiomerically enriched **2s** (In neat)

Supplementary Figure 61. HPLC spectra for compound **2s**

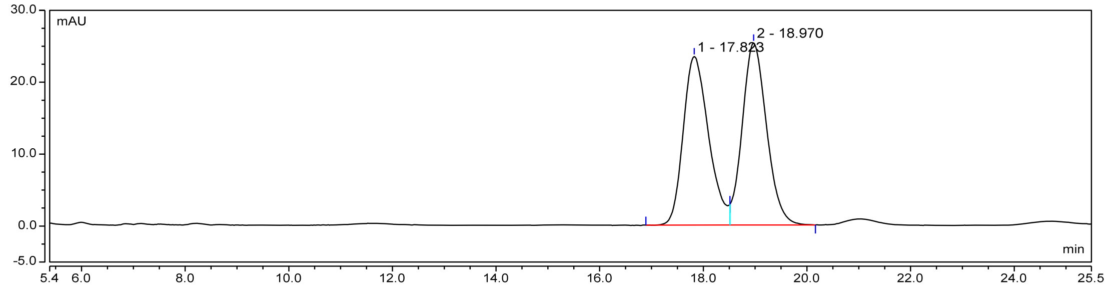

Entry	Retention Time	Area	Height	%Area
1	17.823	13.3433	23.44	49.22
2	18.970	13.7676	25.32	50.78

Racemic **2t**

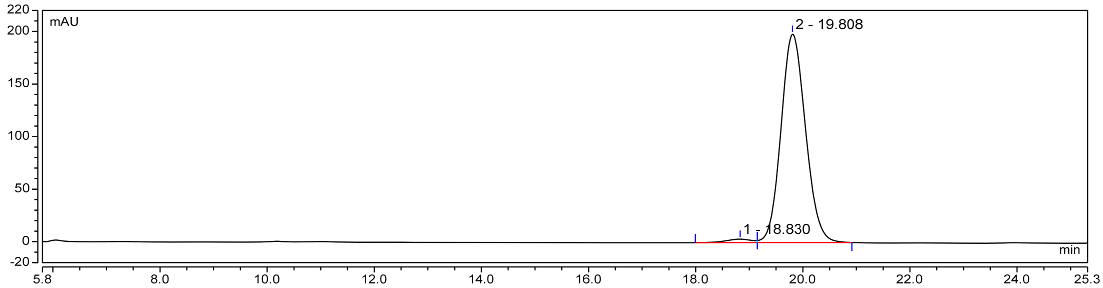

Entry	Retention Time	Area	Height	%Area
1	18.830	1.8089	3.27	1.69
2	19.808	105.1553	198.29	98.31

Enantiomerically enriched **2t** (In PhCl)

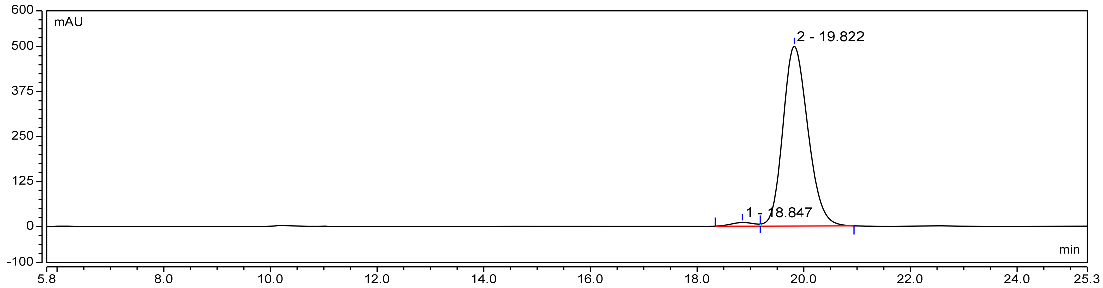

Entry	Retention Time	Area	Height	%Area
1	18.847	5.0546	9.97	1.83
2	19.822	271.4252	499.64	98.17

Enantiomerically enriched **2t** (In neat)

Supplementary Figure 62. HPLC spectra for compound **2t**

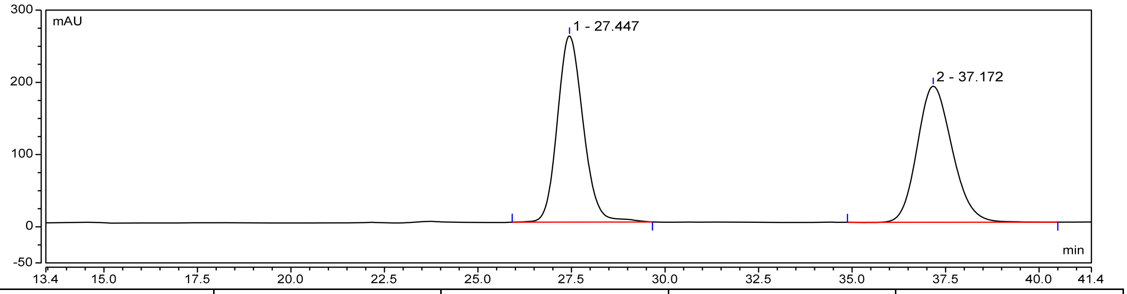

Entry	Retention Time	Area	Height	%Area
1	27.447	206.8220	257.91	50.31
2	37.172	204.3024	188.37	49.69

Racemic **2u**

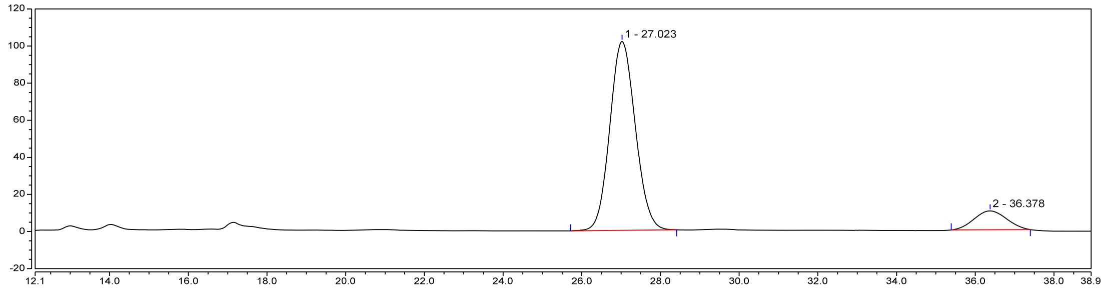

Entry	Retention Time	Area	Height	%Area
1	27.022	88.7	101.8	88.76
2	36.377	11.2	10.1	11.24

Enantiomerically enriched **2u** (In PhCl)

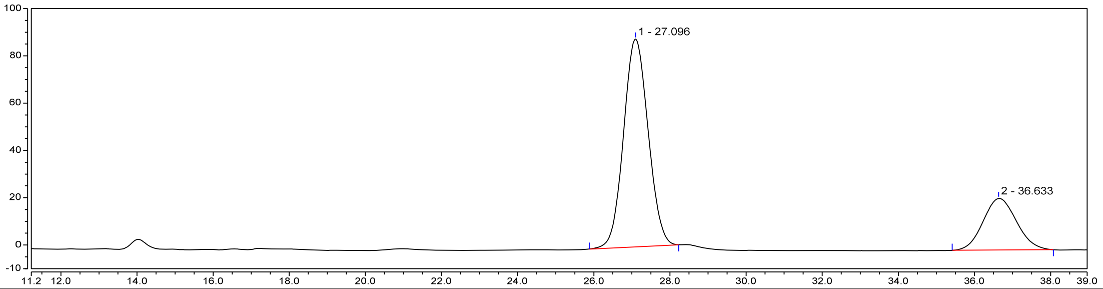

Entry	Retention Time	Area	Height	%Area
1	27.095	65.8	87.9	74.86
2	36.632	22.1	21.8	25.14

Enantiomerically enriched **2u** (In neat)

Supplementary Figure 63. HPLC spectra for compound **2u**

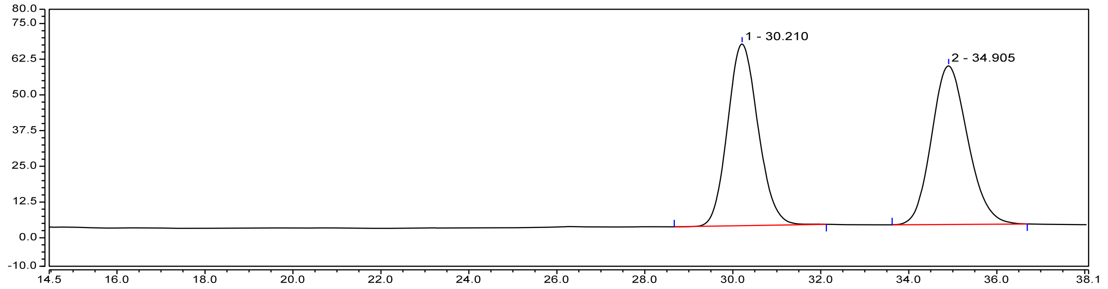

Entry	Retention Time	Area	Height	%Area
1	30.210	51.1885	63.60	49.18
2	34.905	52.9008	55.48	50.82

Racemic **2v**

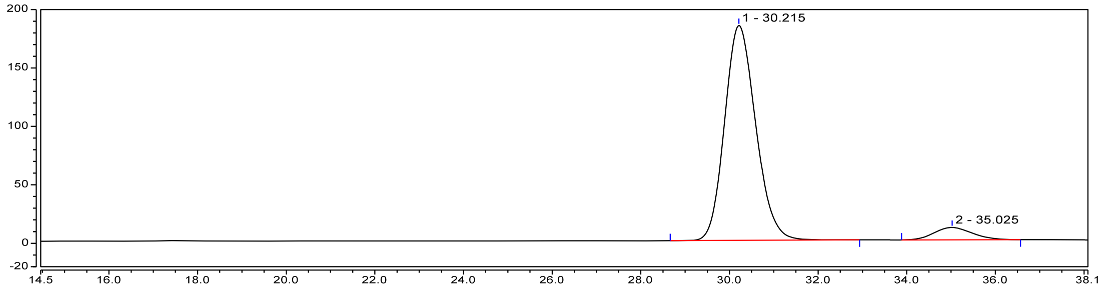

Entry	Retention Time	Area	Height	%Area
1	30.215	150.1572	183.92	93.46
2	35.025	10.5069	10.59	6.54

Enantiomerically enriched **2v** (In PhCl)

Supplementary Figure 64. HPLC spectra for compound **2v**

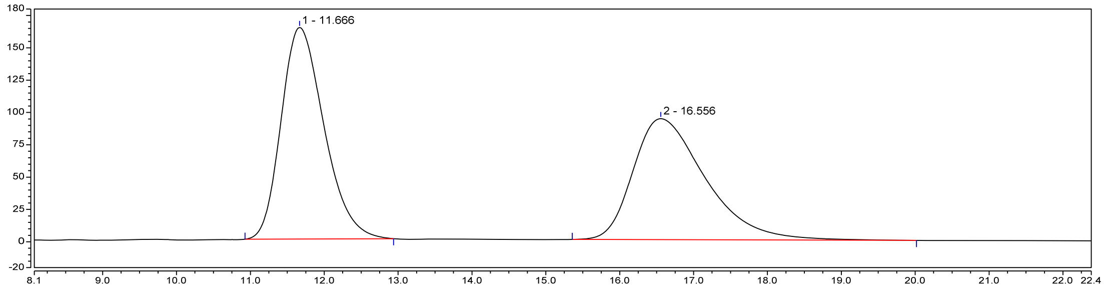

Entry	Retention Time	Area	Height	%Area
1	11.666	110.0765	163.55	50.95
2	16.556	105.9586	93.63	49.05

Racemic **2w** (major)

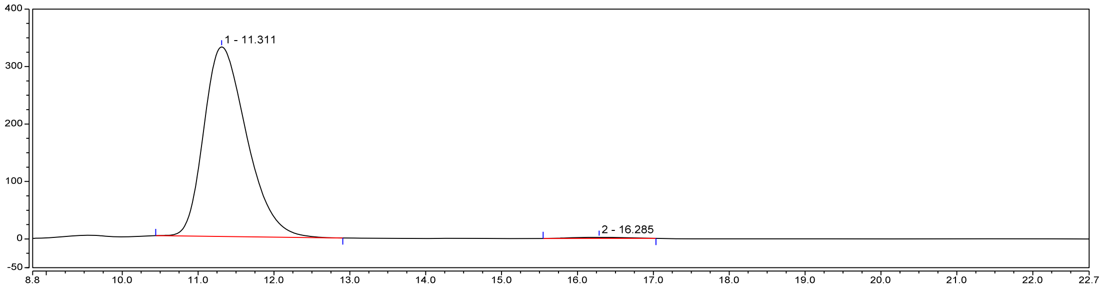

Entry	Retention Time	Area	Height	%Area
1	11.311	216.1418	329.72	99.24
2	16.285	1.6578	2.03	0.76

Enantiomerically enriched **2w** (In PhCl: major)

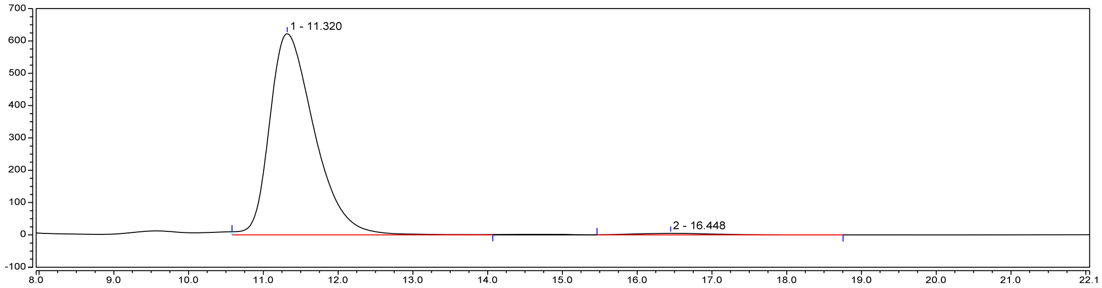

Entry	Retention Time	Area	Height	%Area
1	11.320	430.8668	622.35	98.62
2	16.448	6.0326	5.18	1.38

Enantiomerically enriched **2w** (In neat: major)

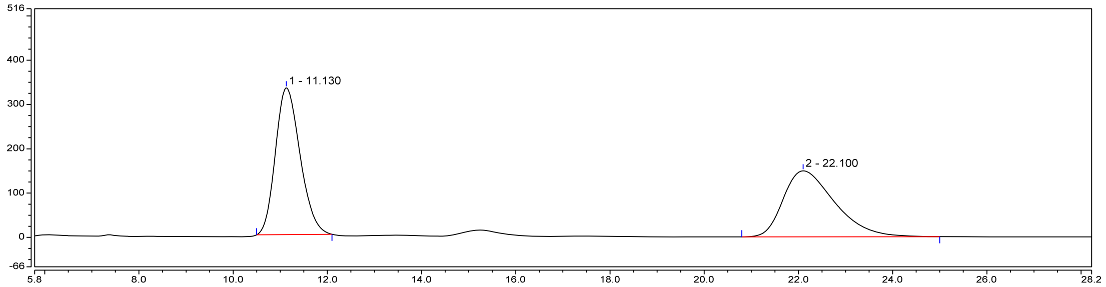

Entry	Retention Time	Area	Height	%Area
1	11.130	201.0517	331.18	51.69
2	22.100	187.9166	148.88	48.31

Racemic **2w** (minor)

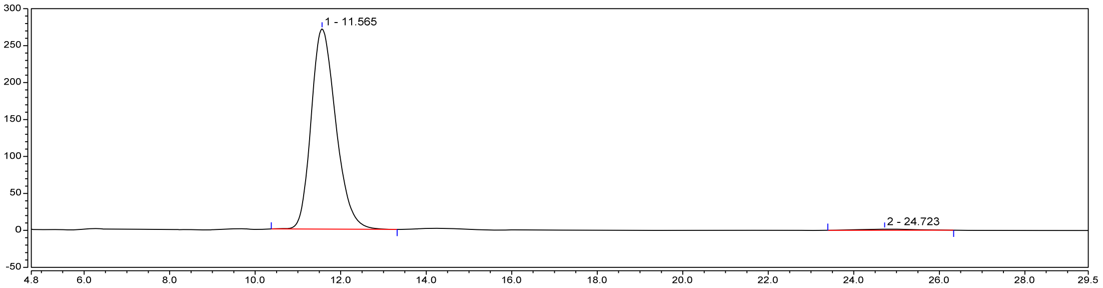

Entry	Retention Time	Area	Height	%Area
1	11.565	181.4090	270.84	98.90
2	24.723	2.0097	1.47	1.10

Enantiomerically enriched **2w** (In PhCl: minor)

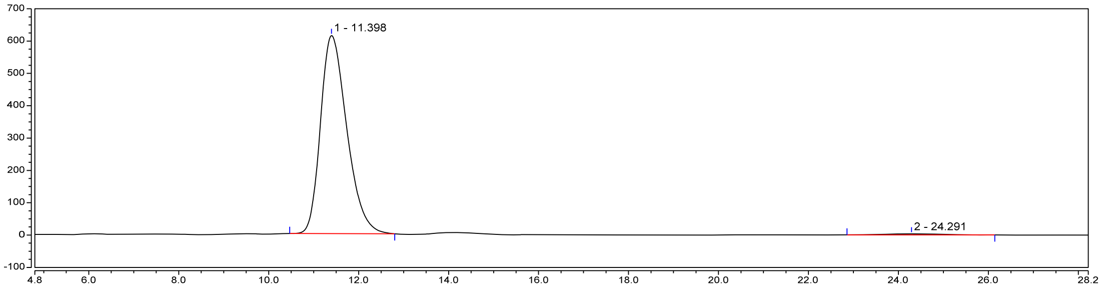

Entry	Retention Time	Area	Height	%Area
1	11.398	416.4246	613.42	99.07
2	24.291	3.9065	3.18	0.93

Enantiomerically enriched **2w** (In neat: minor)

Supplementary Figure 65. HPLC spectra for compound **2w**

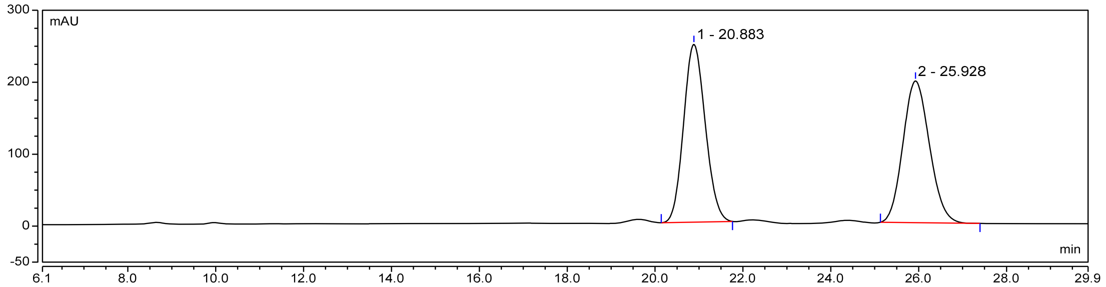

Entry	Retention Time	Area	Height	%Area
1	20.883	141.6299	247.14	50.60
2	25.928	138.2458	197.02	49.40

Racemic **2x** (major)

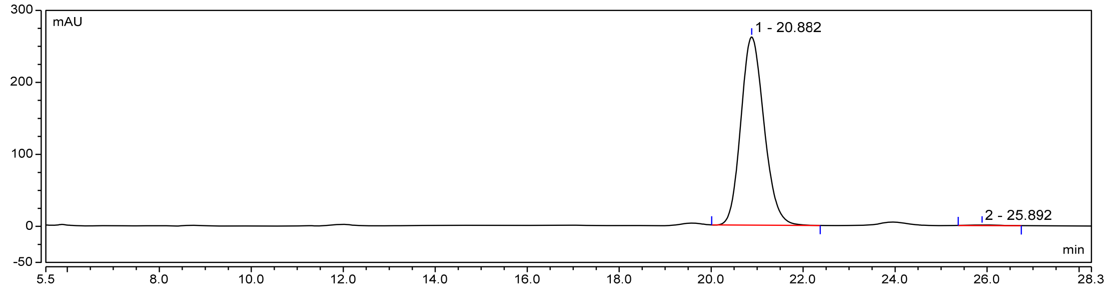

Entry	Retention Time	Area	Height	%Area
1	20.882	152.7887	261.33	99.58
2	25.892	0.6417	1.03	0.42

Enantiomerically enriched **2x** (In PhCl: major)

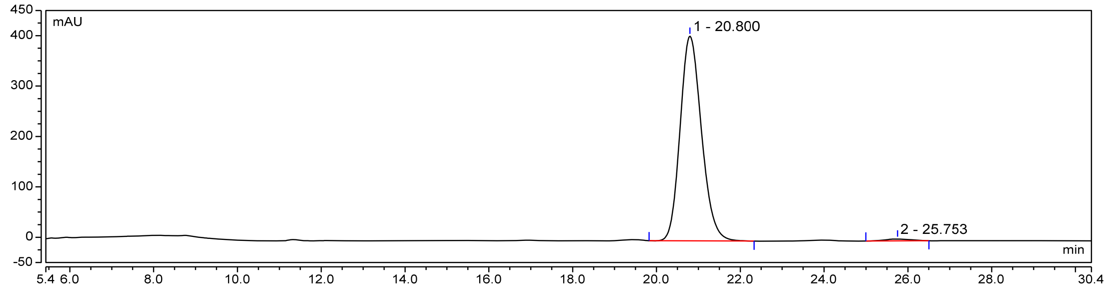

Entry	Retention Time	Area	Height	%Area
1	20.800	240.1602	406.18	98.93
2	25.753	2.6069	3.84	1.07

Enantiomerically enriched **2x** (In neat: major)

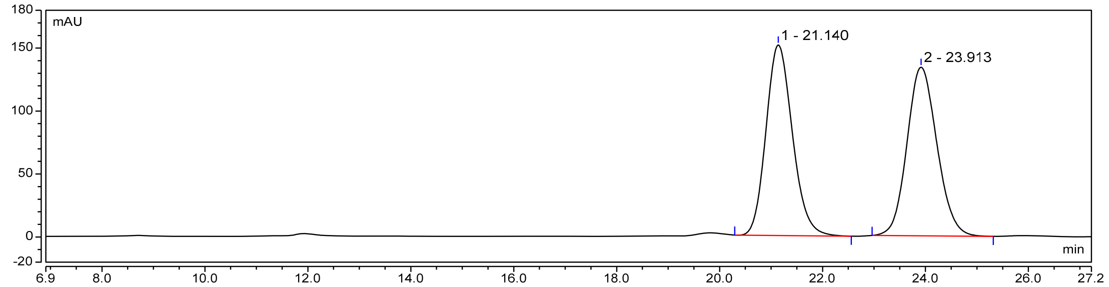

Entry	Retention Time	Area	Height	%Area
1	21.140	90.2047	151.30	49.93
2	23.913	90.4744	133.93	50.07

Racemic **2x** (minor)

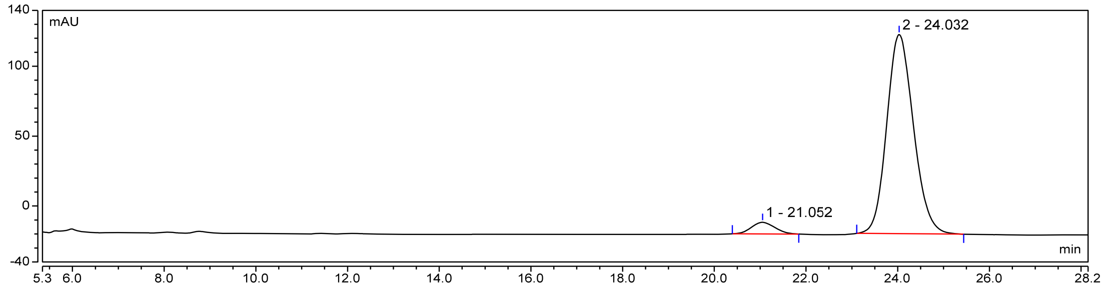

Entry	Retention Time	Area	Height	%Area
1	21.052	4.9467	8.34	4.88
2	24.032	96.4494	142.55	95.12

Enantiomerically enriched **2x** (In PhCl: minor)

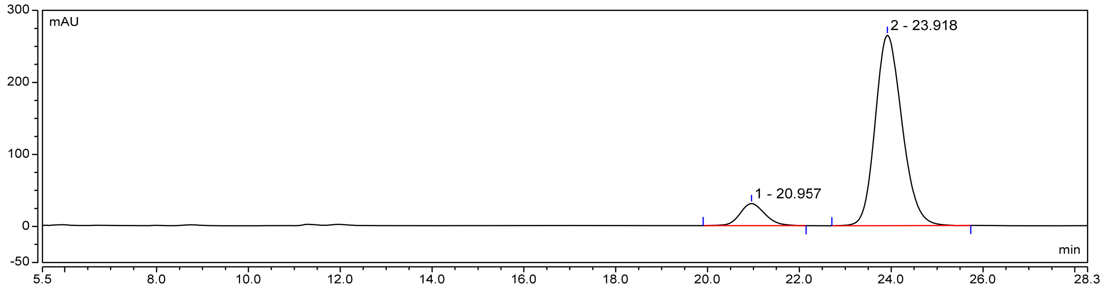

Entry	Retention Time	Area	Height	%Area
1	20.957	19.2449	30.60	9.71
2	23.918	178.9324	264.39	90.29

Enantiomerically enriched **2x** (In neat: minor)

Supplementary Figure 66. HPLC spectra for compound **2x**

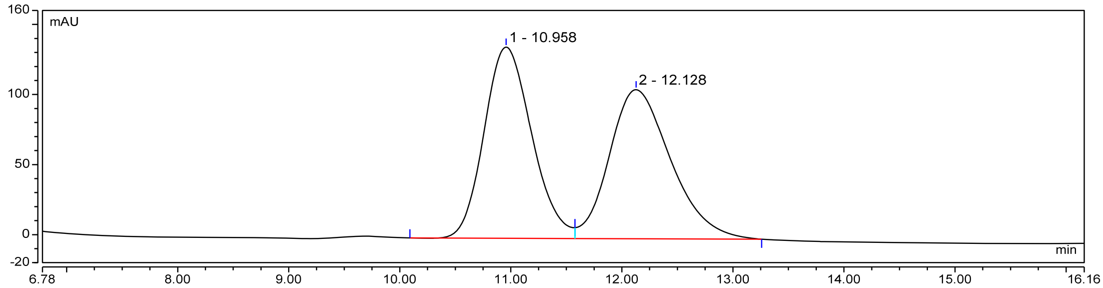

Entry	Retention Time	Area	Height	%Area
1	10.958	67.8059	136.36	49.85
2	12.128	68.2189	106.34	50.15

Racemic **2y** (major)

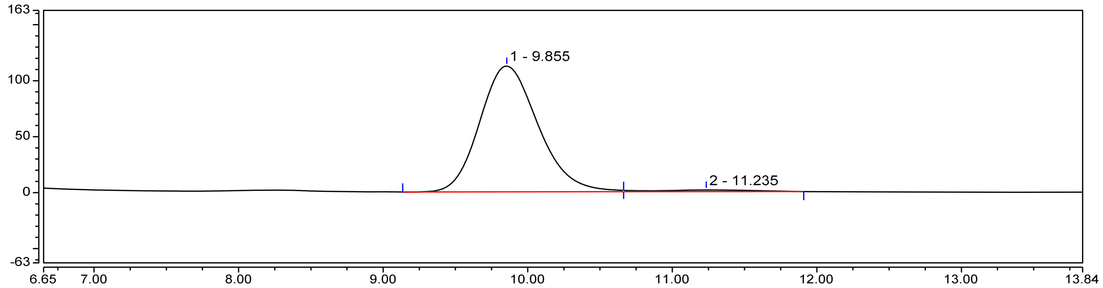

Entry	Retention Time	Area	Height	%Area
1	9.855	52.5429	112.31	97.84
2	11.235	1.1604	1.50	2.16

Enantiomerically enriched **2y** (In PhCl: major)

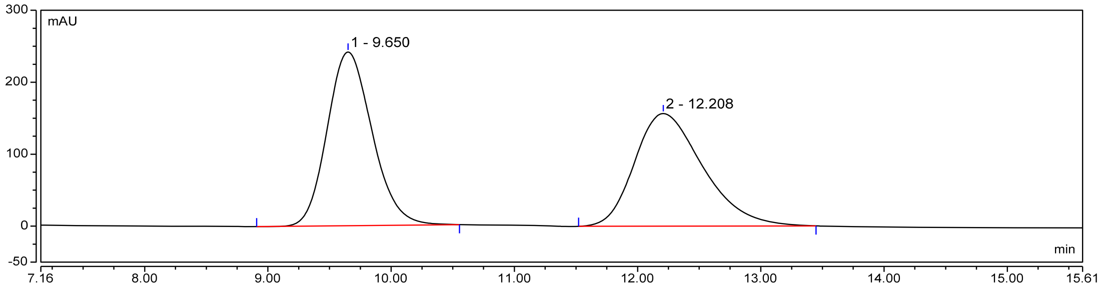

Entry	Retention Time	Area	Height	%Area
1	9.650	102.3132	241.42	50.38
2	12.208	100.7646	156.50	49.62

Racemic **2y** (minor)

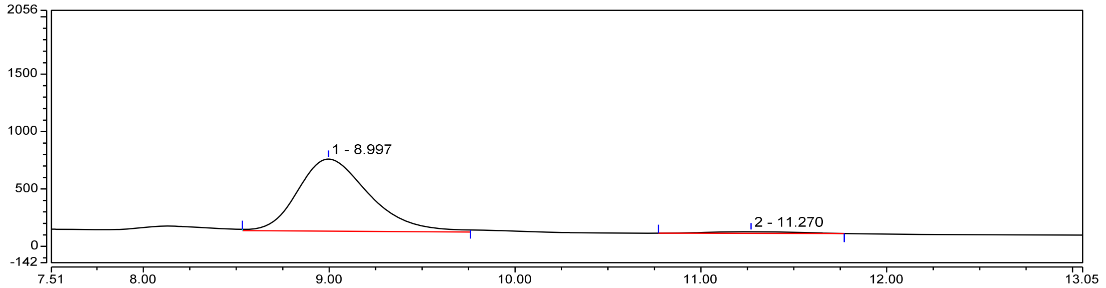

Entry	Retention Time	Area	Height	%Area
1	8.997	280.0803	628.22	97.26
2	11.270	7.8898	14.97	2.74

Enantiomerically enriched **2y** (In PhCl: minor)

Supplementary Figure 67. HPLC spectra for compound **2y**

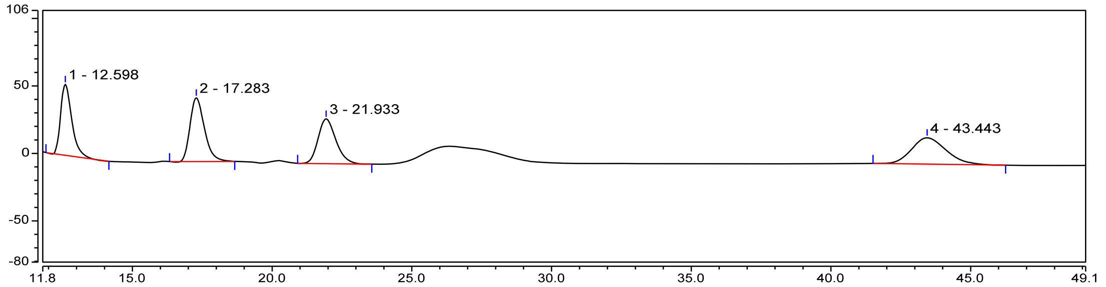

Entry	Retention Time	Area	Height	%Area
1	12.598	24.3551	52.51	23.12
2	17.283	28.2382	47.24	26.80
3	21.933	24.5918	33.13	23.34
4	43.443	28.1644	19.54	26.73

Racemic **2z**

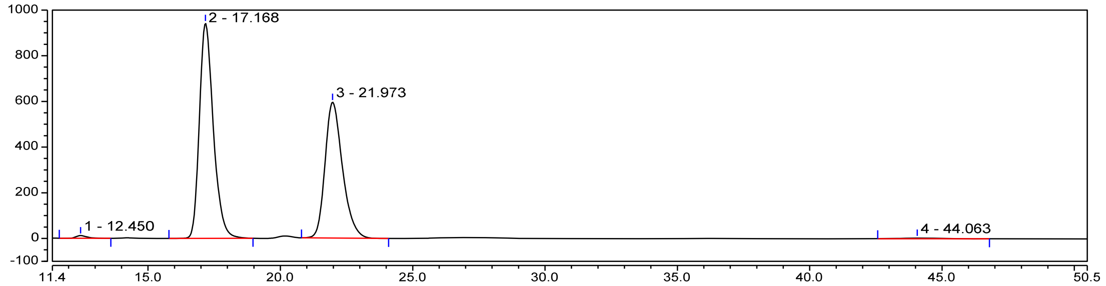

Entry	Retention Time	Area	Height	%Area
1	12.450	5.6806	11.90	0.55

2	17.168	576.3981	942.41	55.30
3	21.973	455.1090	595.09	43.67
4	44.063	5.0819	3.54	0.49

Enantiomerically enriched **2z** (In PhCl)

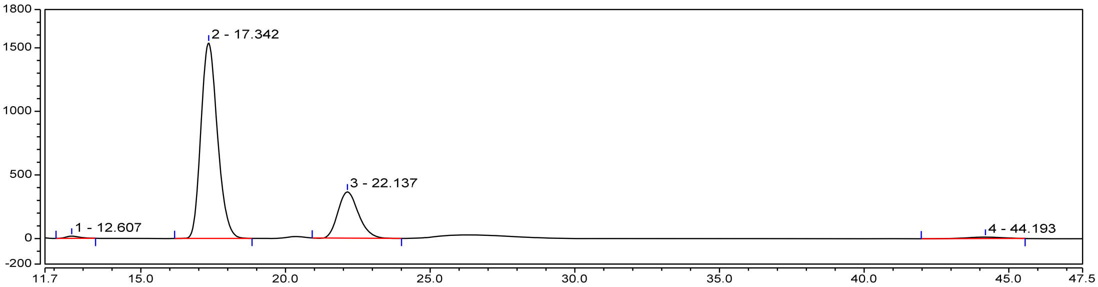

Entry	Retention Time	Area	Height	%Area
1	12.607	9.6988	17.84	0.72
2	17.342	1017.8629	1536.92	75.85
3	22.137	297.9273	363.53	22.20
4	44.193	16.4233	11.58	1.22

Enantiomerically enriched **2z** (In neat)

Supplementary Figure 68. HPLC spectra for compound **2z**

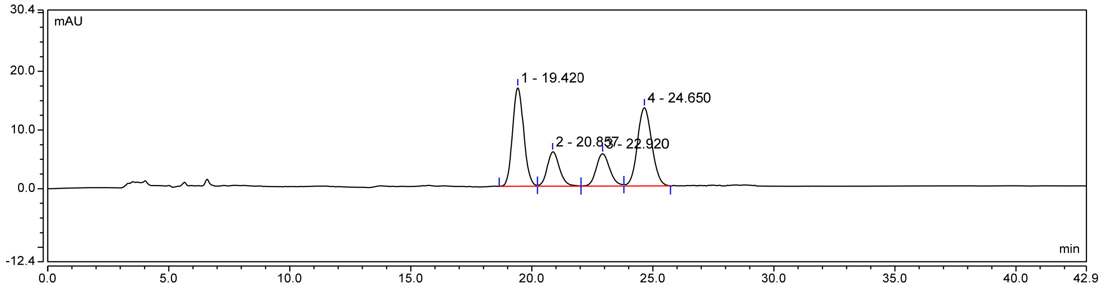

Entry	Retention Time	Area	Height	%Area
1	19.420	9.0213	16.80	35.80
2	20.857	3.4722	5.87	13.78
3	22.920	3.5585	5.49	14.12
4	24.650	9.1442	13.34	36.29

Racemic **2aa**

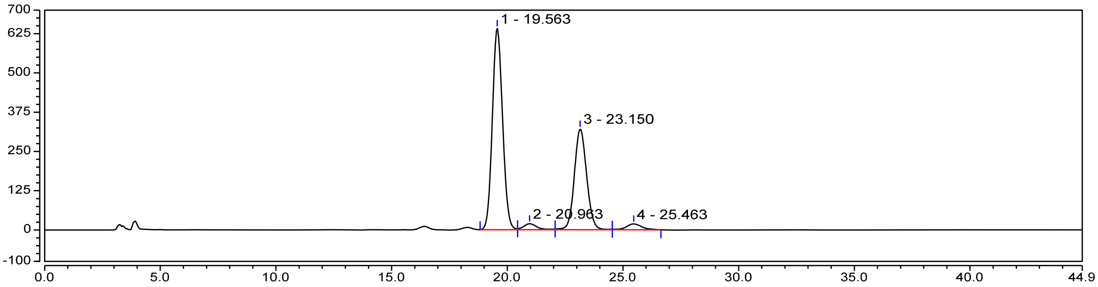

Entry	Retention Time	Area	Height	%Area
1	19.563	328.7173	640.74	59.39
2	20.963	12.4512	18.97	2.25
3	23.150	197.9316	320.91	35.76
4	25.463	14.3753	18.77	2.60

Enantiomerically enriched **2aa** (In PhCl)

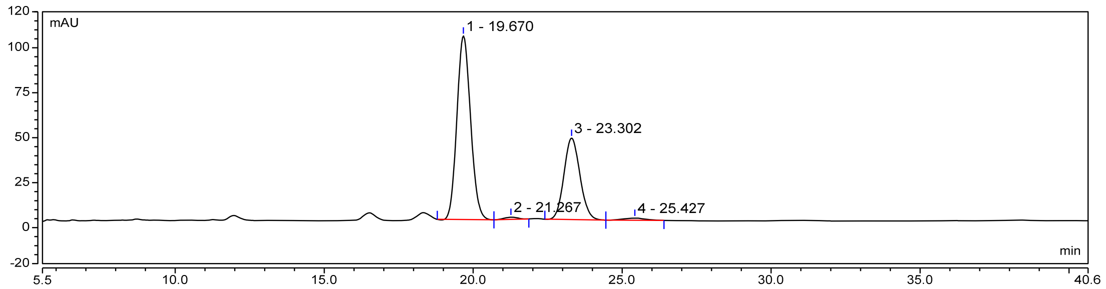

Entry	Retention Time	Area	Height	%Area
1	19.670	54.4040	102.12	64.58
2	21.267	0.6309	1.22	0.75
3	23.302	28.2248	45.32	33.50
4	25.427	0.9833	1.18	1.17

Enantiomerically enriched **2aa** (In neat)

Supplementary Figure 69. HPLC spectra for compound **2aa**

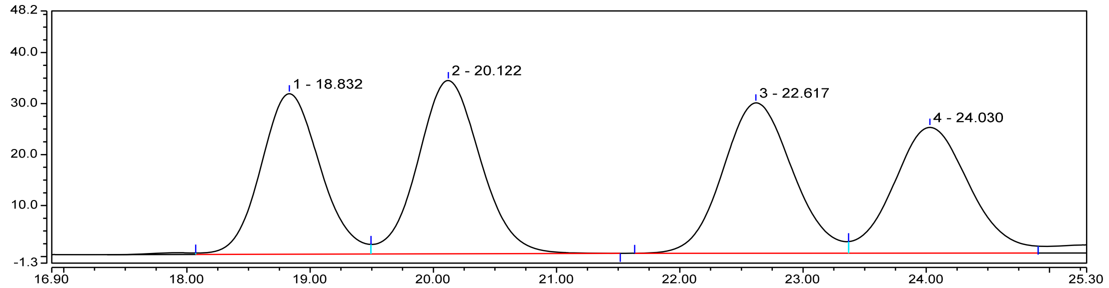

Entry	Retention Time	Area	Height	%Area
1	18.832	17.5318	31.49	23.54
2	20.122	19.9913	34.00	26.84
3	22.617	19.3960	29.50	26.05
4	24.030	17.5511	24.71	23.57

Racemic **2ab**

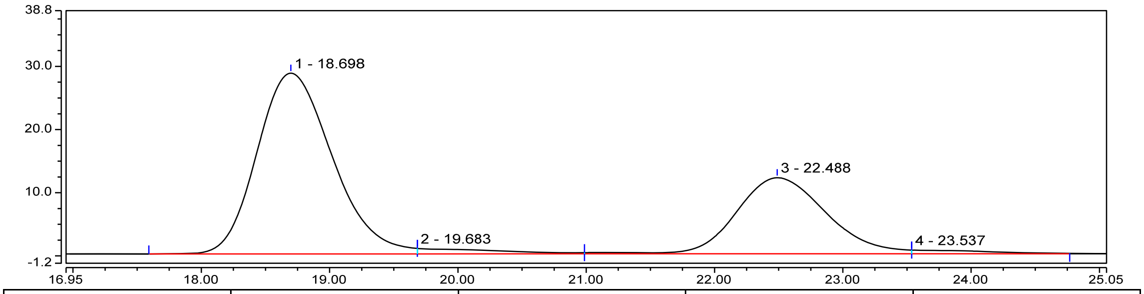

Entry	Retention Time	Area	Height	%Area
1	18.698	19.5066	28.64	63.95
2	19.690	0.6462	0.89	2.12
3	22.488	9.8378	12.08	32.25
4	23.482	0.5138	0.68	1.68

Enantiomerically enriched **2ab** (In PhCl)

Supplementary Figure 70. HPLC spectra for compound **2ab**

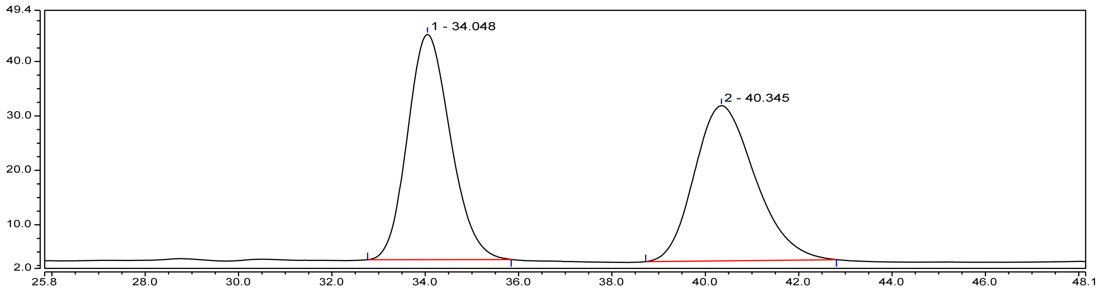

Entry	Retention Time	Area	Height	%Area
1	34.048	44.2191	41.40	50.63
2	40.345	43.1109	28.53	49.37

Racemic **2ac**

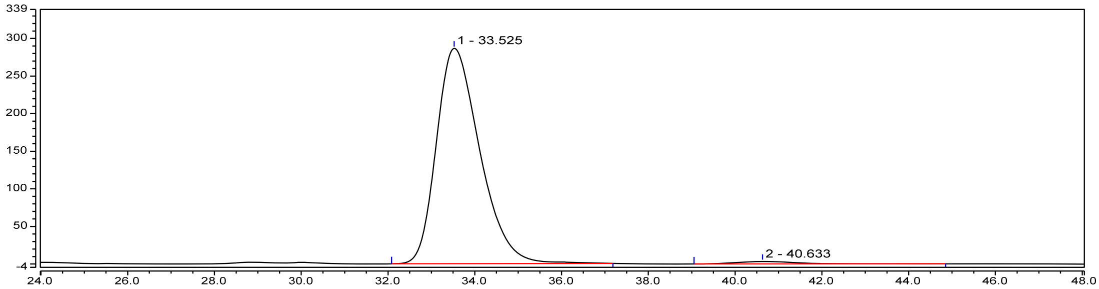

Entry	Retention Time	Area	Height	%Area
1	33.525	332.4946	286.58	98.61
2	40.633	4.6963	3.28	1.39

Enantiomerically enriched **2ac** (In PhCl)

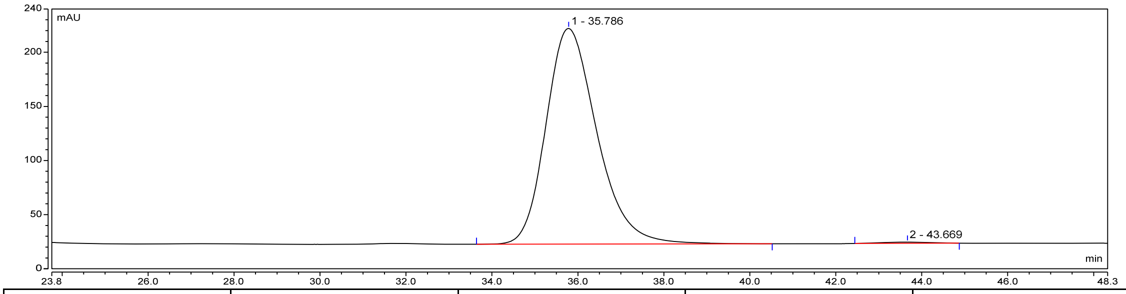

Entry	Retention Time	Area	Height	%Area
1	35.786	273.7329	199.20	99.47
2	43.669	1.4559	1.1633	0.53

Enantiomerically enriched **2ac** (In neat)

Supplementary Figure 71. HPLC spectra for compound **2ac**

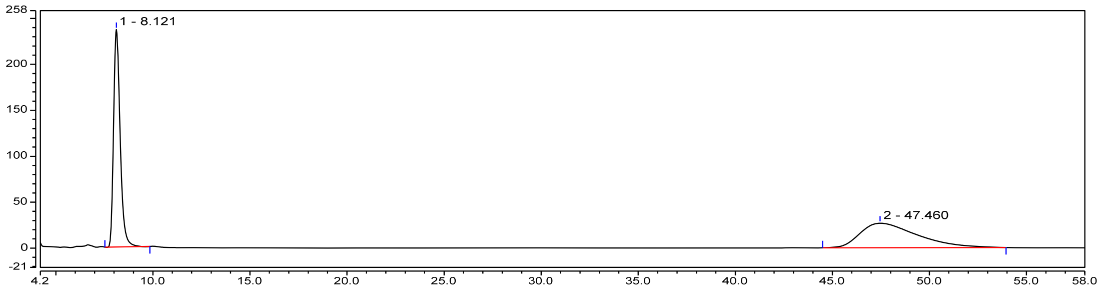

Entry	Retention Time	Area	Height	%Area
1	8.121	92.2013	236.64	50.32

2	47.460	91.0374	26.74	49.68

Racemic **2ad**

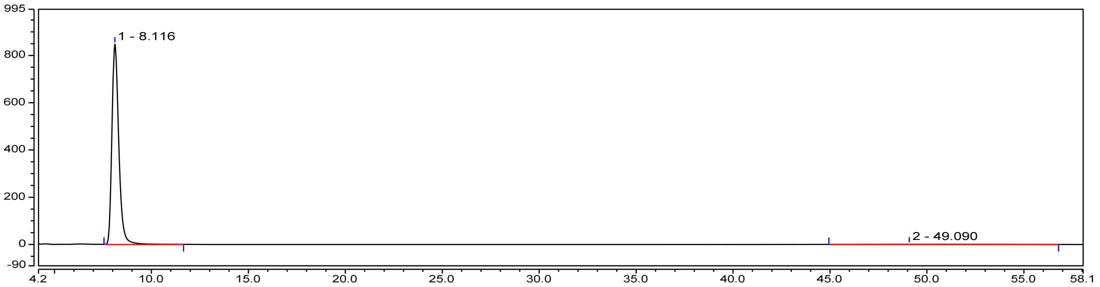

Entry	Retention Time	Area	Height	%Area
1	8.116	346.6097	847.26	98.50
2	49.090	5.2960	1.41	1.50

Enantiomerically enriched **2ad** (In PhCl)

Supplementary Figure 72. HPLC spectra for compound **2ad**

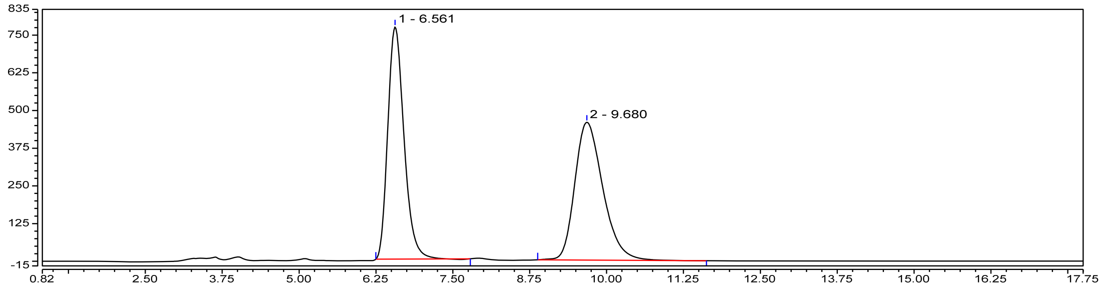

Entry	Retention Time	Area	Height	%Area
1	6.561	235.4918	770.13	49.32
2	9.680	242.0015	457.07	50.68

Racemic **2ae**

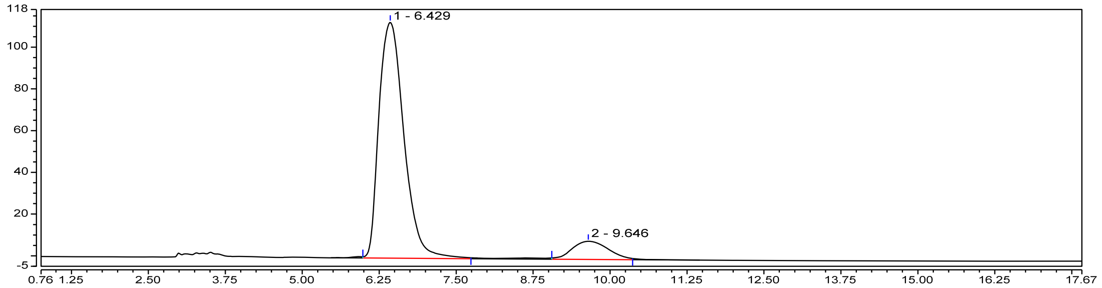

Entry	Retention Time	Area	Height	%Area
1	6.429	53.2041	113.02	90.03
2	9.646	5.8891	8.64	9.97

Enantiomerically enriched **2ae** (In PhCl)

Supplementary Figure 73. HPLC spectra for compound **2ae**

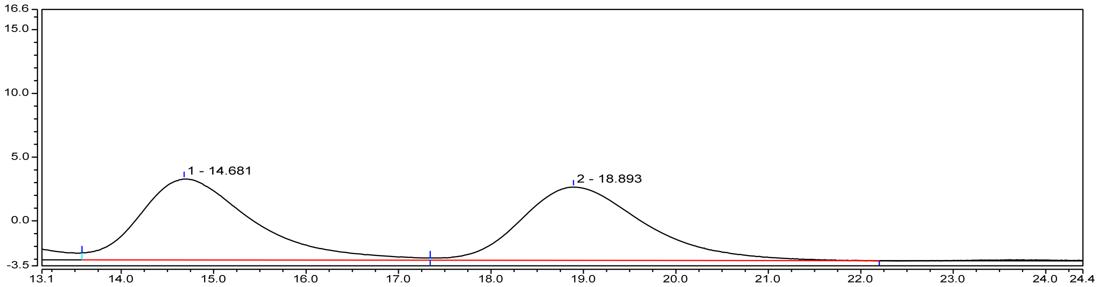

Entry	Retention Time	Area	Height	%Area
1	14.681	9.0191	6.36	50.37
2	18.893	8.8864	5.75	49.63

Racemic **2af**

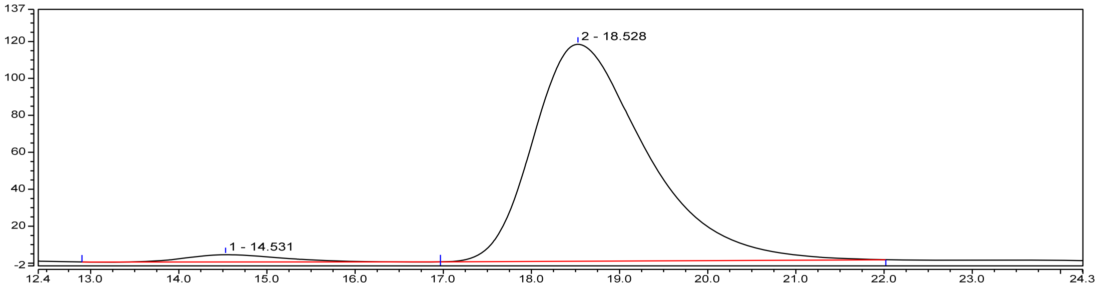

Entry	Retention Time	Area	Height	%Area
1	14.531	4.9053	3.94	2.73
2	18.528	174.6500	117.51	97.27

Enantiomerically enriched **2af** (In PhCl)

Supplementary Figure 74. HPLC spectra for compound **2af**

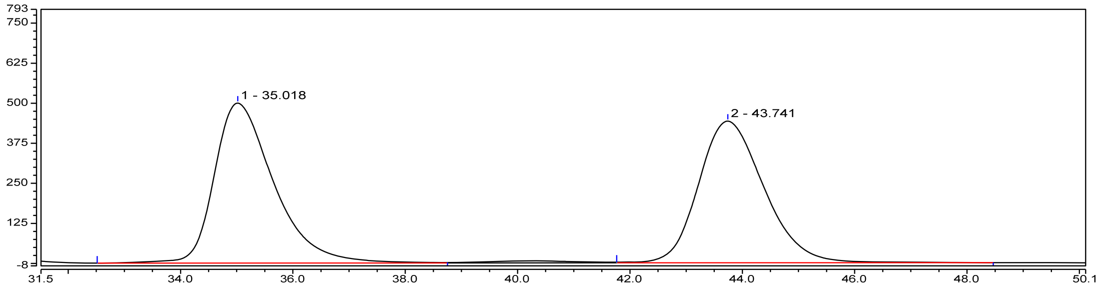

Entry	Retention Time	Area	Height	%Area
1	35.018	614.0368	499.37	50.67
2	43.741	597.7736	442.09	49.33

Racemic **2ag** (major)

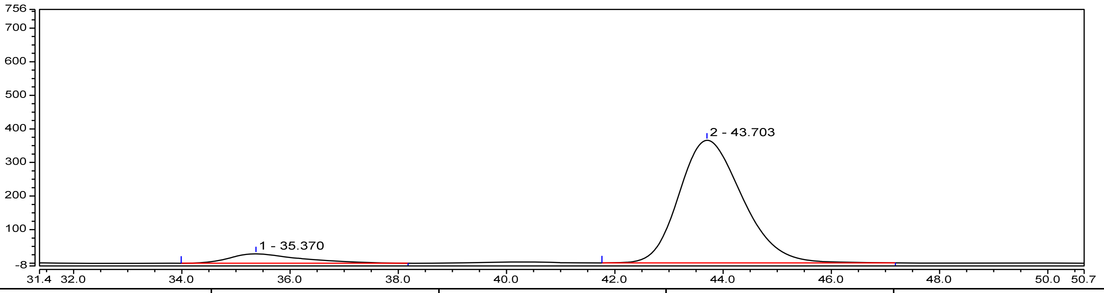

Entry	Retention Time	Area	Height	%Area
1	35.370	42.3469	27.96	7.89
2	43.703	494.4429	365.33	92.11

Enantiomerically enriched **2ag** (In PhCl: major)

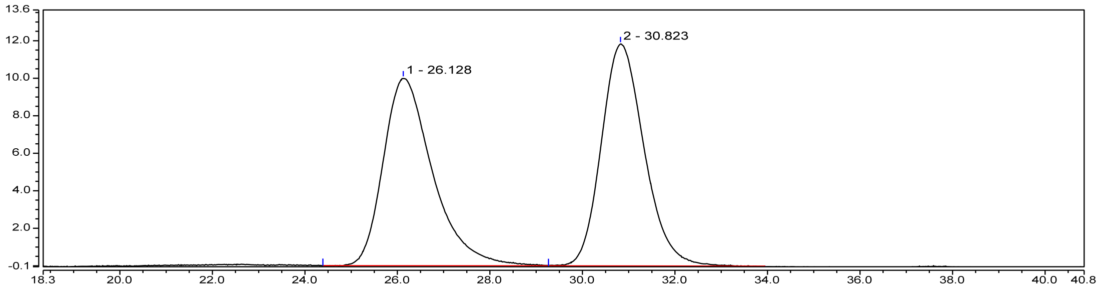

Entry	Retention Time	Area	Height	%Area
1	26.128	12.3861	10.00	49.38
2	30.823	12.6990	11.84	50.62

Racemic **2ag** (minor)

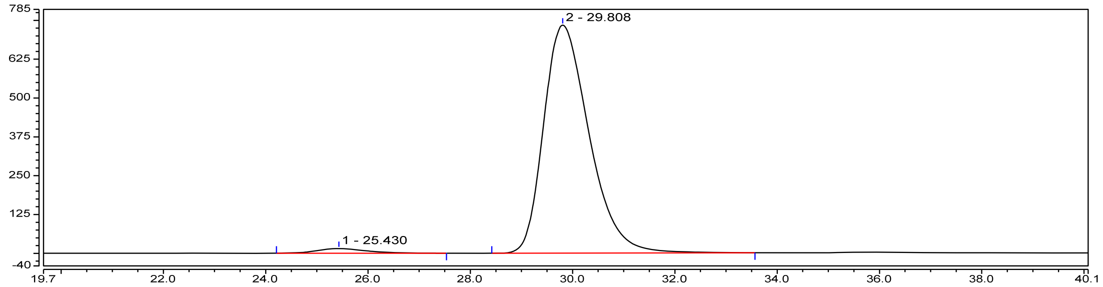

Entry	Retention Time	Area	Height	%Area
1	25.430	16.7126	15.07	2.17
2	29.808	754.6319	733.57	97.83

Enantiomerically enriched **2ag** (In PhCl: minor)

Supplementary Figure 75. HPLC spectra for compound **2ag**

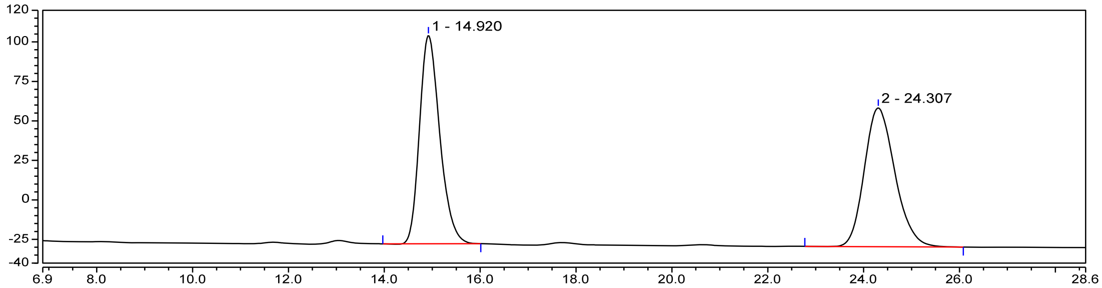

Entry	Retention Time	Area	Height	%Area
1	14.967	46.1312	99.29	50.36
2	19.013	45.4679	75.67	49.64

Racemic **2ah**

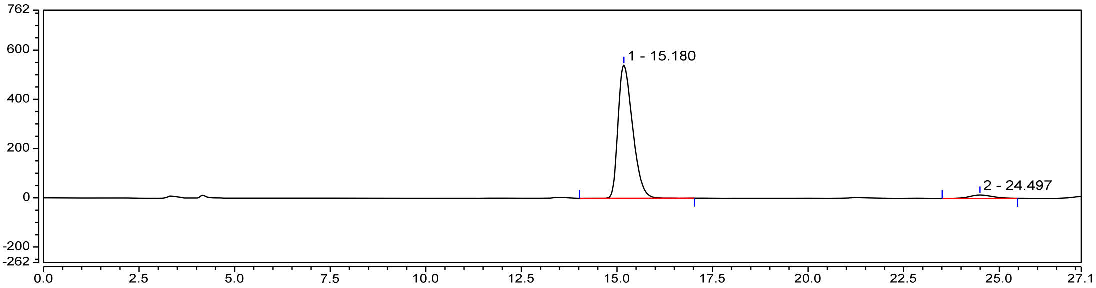

Entry	Retention Time	Area	Height	%Area
1	15.180	238.0419	540.40	96.20

2	24.497	9.3900	13.66	3.80

Enantiomerically enriched **2ah** (In PhCl)

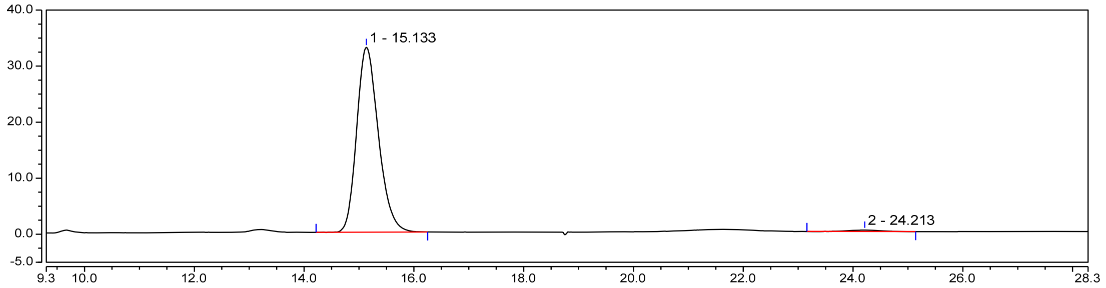

Entry	Retention Time	Area	Height	%Area
1	15.133	15.1450	32.99	98.91
2	24.213	0.1669	0.27	1.09

Enantiomerically enriched **2ah** (In neat)

Supplementary Figure 76. HPLC spectra for compound **2ah**

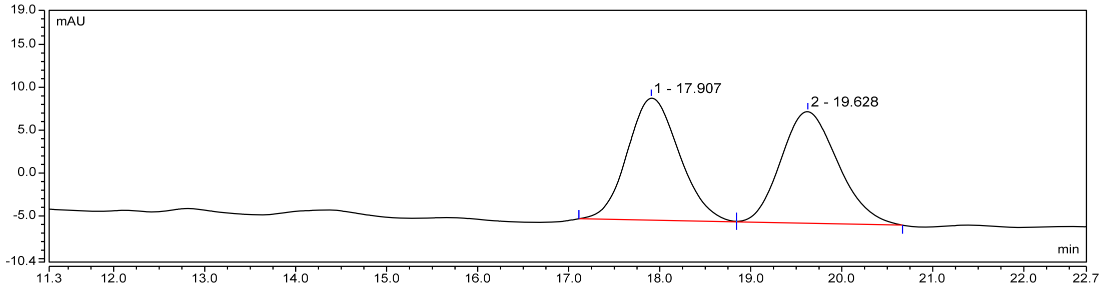

Entry	Retention Time	Area	Height	%Area
1	17.907	9.4125	14.24	49.92
2	19.628	9.4446	13.02	50.08

Racemic **2ai**

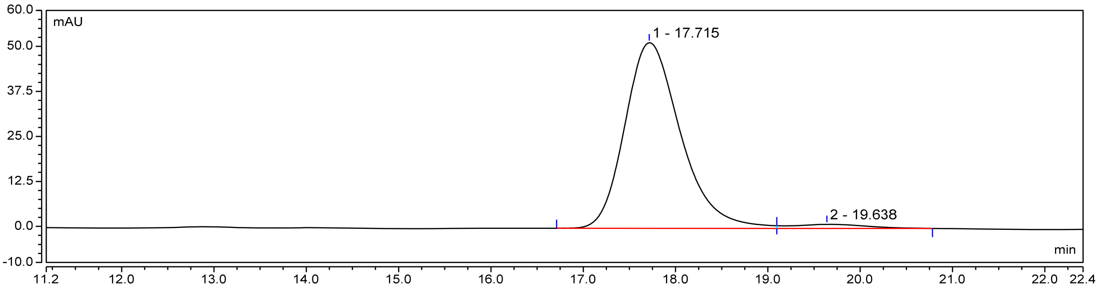

Entry	Retention Time	Area	Height	%Area
1	17.715	36.0709	51.56	97.21
2	19.638	1.0368	1.13	2.79

Enantiomerically enriched **2ai** (In PhCl)

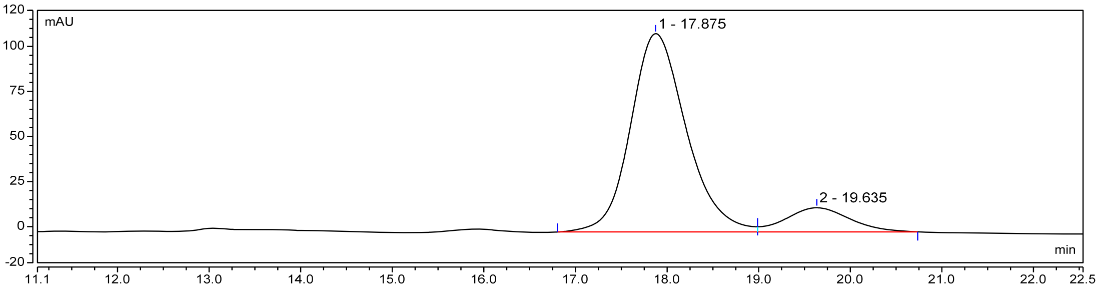

Entry	Retention Time	Area	Height	%Area

1	17.875	77.5771	110.07	87.79
2	19.635	10.7872	13.40	12.21

Enantiomerically enriched **2ai** (In neat)

Supplementary Figure 77. HPLC spectra for compound **2ai**

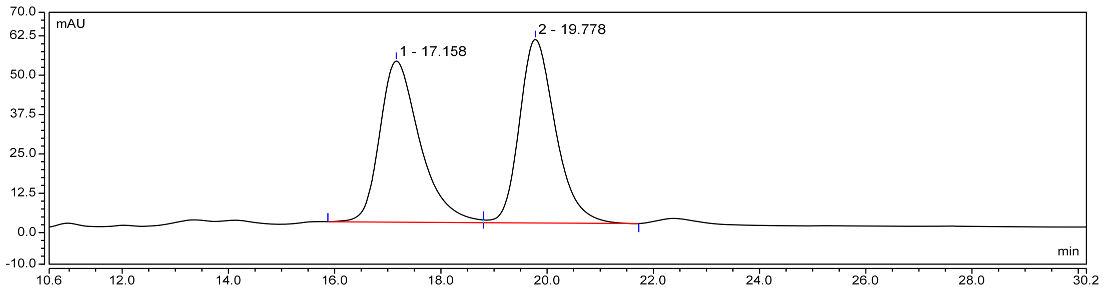

Entry	Retention Time	Area	Height	%Area
1	17.158	44.8969	51.15	49.45
2	19.778	45.8969	58.32	50.55

Racemic **2aj**

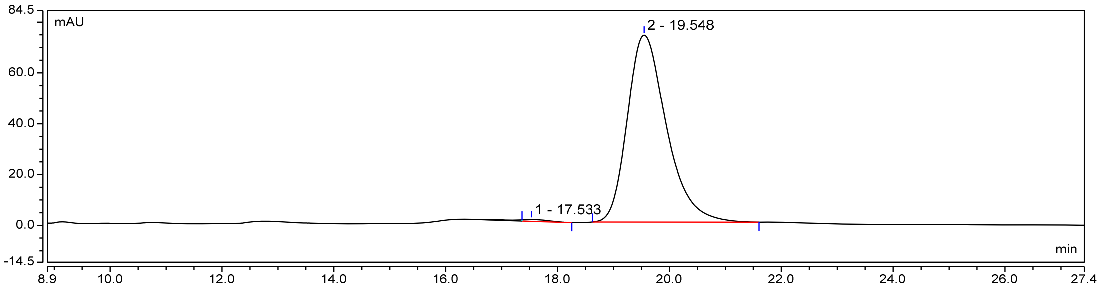

Entry	Retention Time	Area	Height	%Area
1	17.533	0.3532	0.69	0.59
2	19.548	59.7350	73.58	99.41

Enantiomerically enriched **2aj** (In PhCl)

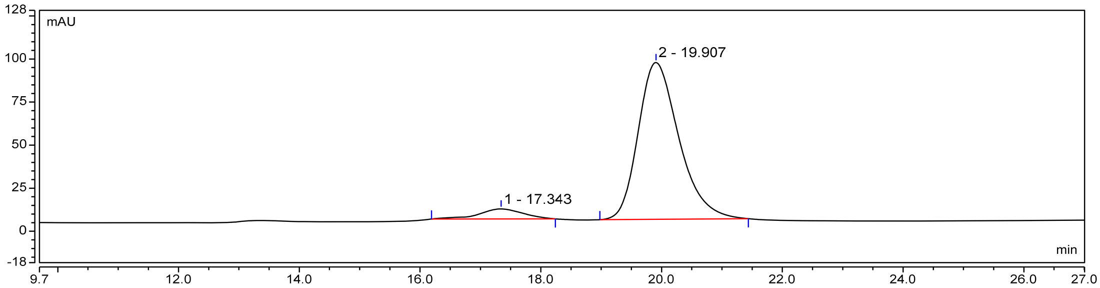

Entry	Retention Time	Area	Height	%Area
1	17.343	4.9191	5.79	6.46
2	19.907	71.1757	91.00	93.54

Enantiomerically enriched **2aj** (In neat)

Supplementary Figure 78. HPLC spectra for compound **2aj**

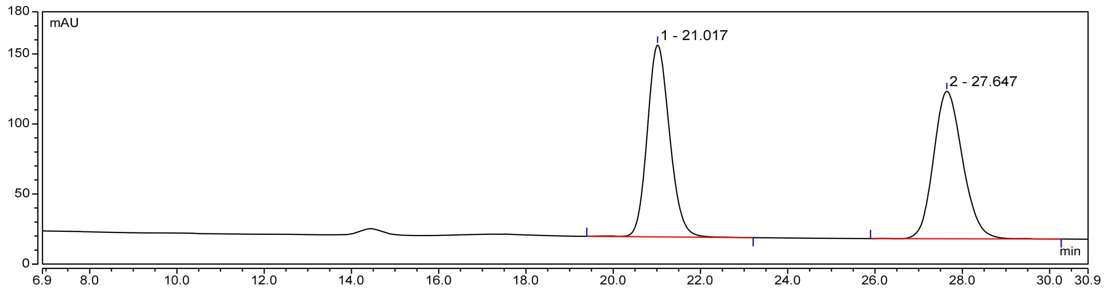

Entry	Retention Time	Area	Height	%Area
1	21.017	81.1822	136.89	50.04
2	27.647	81.0472	105.09	49.96

Racemic **2ak**

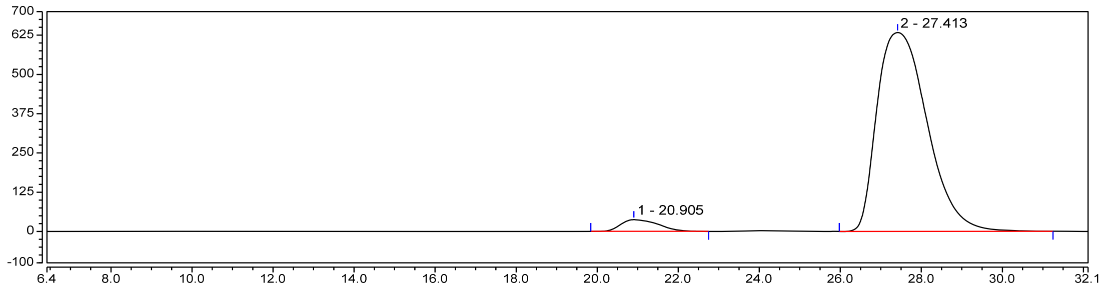

Entry	Retention Time	Area	Height	%Area
1	20.905	39.8883	37.30	4.21
2	27.413	907.8114	633.13	95.79

Enantiomerically enriched **2ak** (In PhCl)

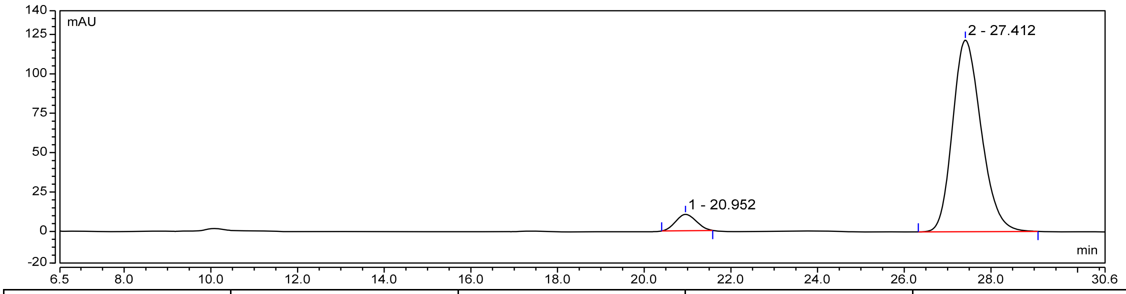

Entry	Retention Time	Area	Height	%Area
1	20.952	5.7028	10.38	5.77
2	27.412	93.1392	121.47	94.23

Enantiomerically enriched **2ak** (In neat)

Supplementary Figure 79. HPLC spectra for compound **2ak**

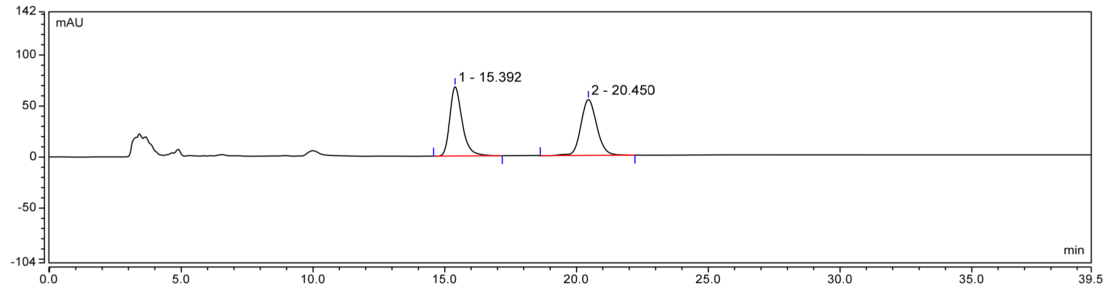

Entry	Retention Time	Area	Height	%Area
1	15.392	36.9607	67.87	49.22
2	20.450	38.1377	54.66	50.78

Racemic **2al**

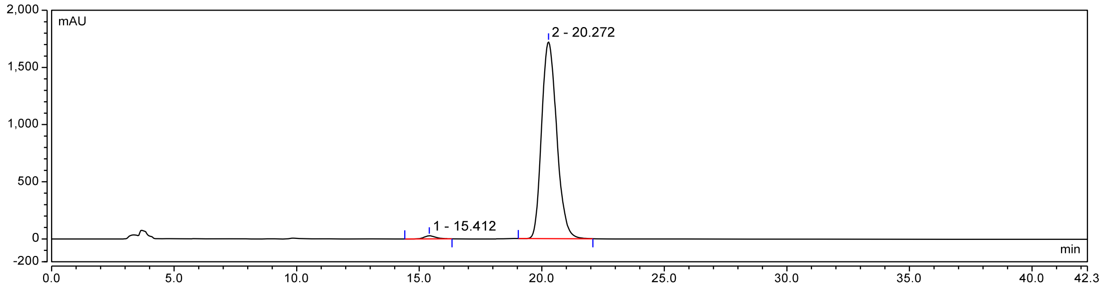

Entry	Retention Time	Area	Height	%Area
1	15.412	14.5732	27.09	1.17
2	20.272	1226.8726	1721.08	98.83

Enantiomerically enriched **2al** (In PhCl)

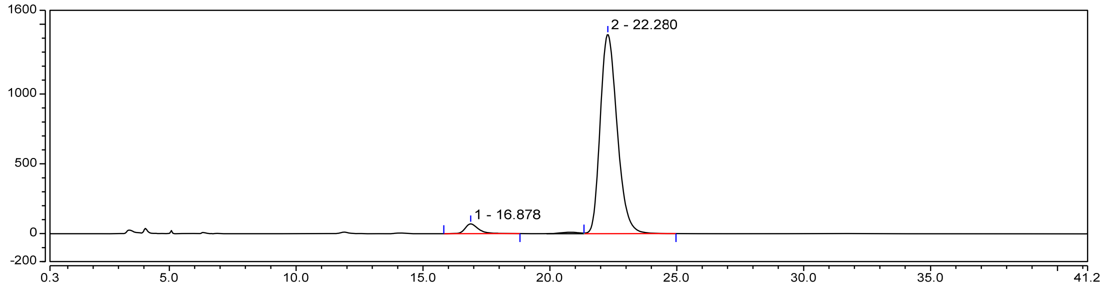

Entry	Retention Time	Area	Height	%Area
1	16.878	43.3034	69.93	3.77
2	22.280	1104.7810	1427.38	96.23

Enantiomerically enriched **2al** (In neat)

Supplementary Figure 80. HPLC spectra for compound **2al**

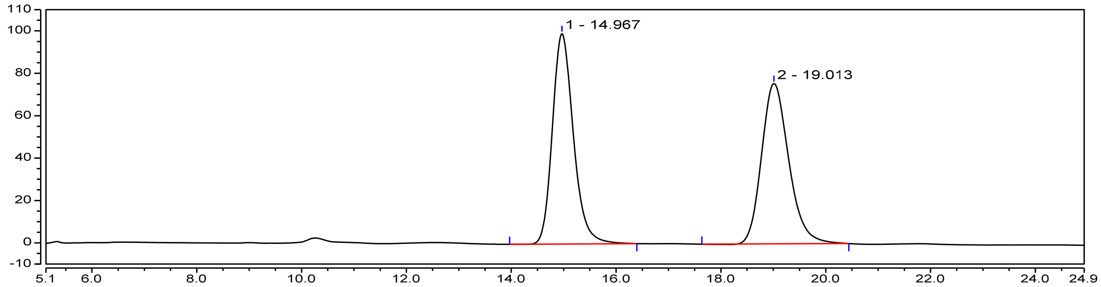

Entry	Retention Time	Area	Height	%Area
1	14.967	46.1312	99.29	50.36
2	19.013	45.4679	75.67	49.64

Racemic **2am**

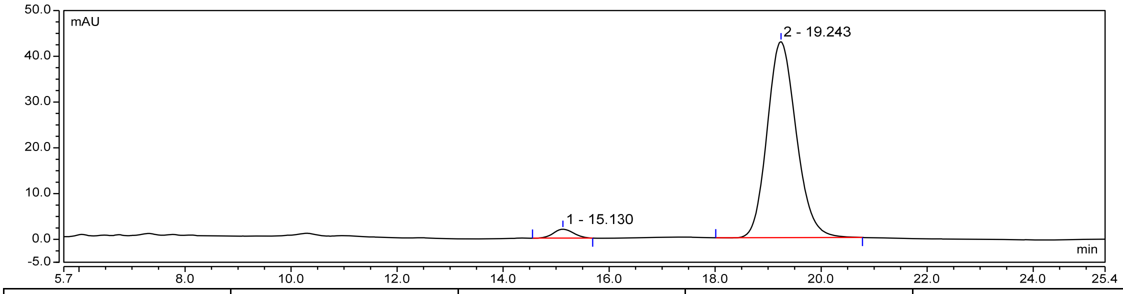

Entry	Retention Time	Area	Height	%Area
1	15.130	0.8516	1.94	3.03

2	19.243	27.2466	42.80	96.97

Enantiomerically enriched **2am** (In PhCl)

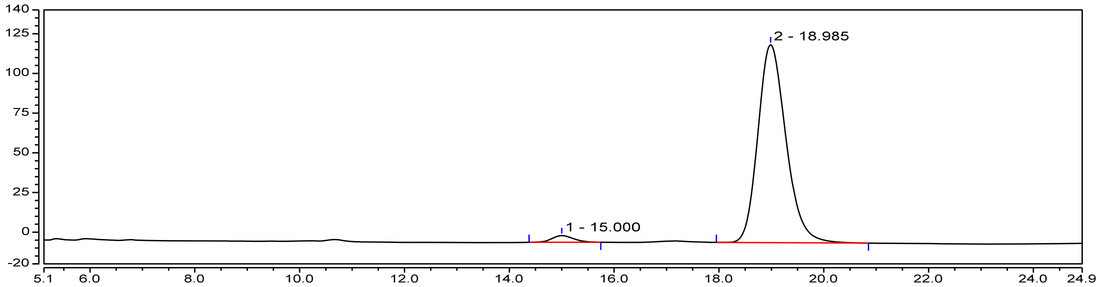

Entry	Retention Time	Area	Height	%Area
1	15.000	1.9852	4.19	2.50
2	18.985	77.4172	124.67	97.50

Enantiomerically enriched **2am** (In neat)

Supplementary Figure 81. HPLC spectra for compound **2am**

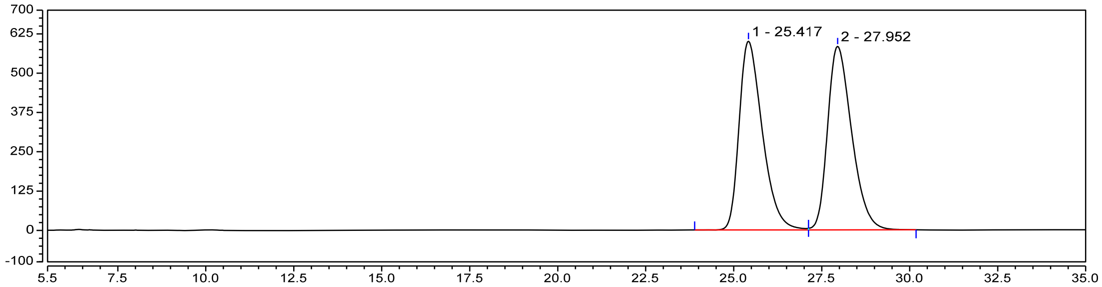

Entry	Retention Time	Area	Height	%Area
1	25.417	459.0478	599.91	49.76
2	27.952	463.5213	583.68	50.24

Racemic **2an**

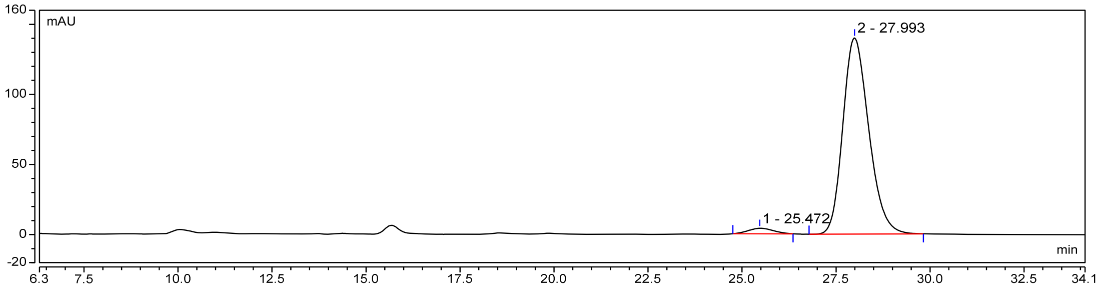

Entry	Retention Time	Area	Height	%Area
1	25.472	3.0503	3.95	2.65
2	27.993	112.0298	139.97	97.35

Enantiomerically enriched **2an** (In PhCl)

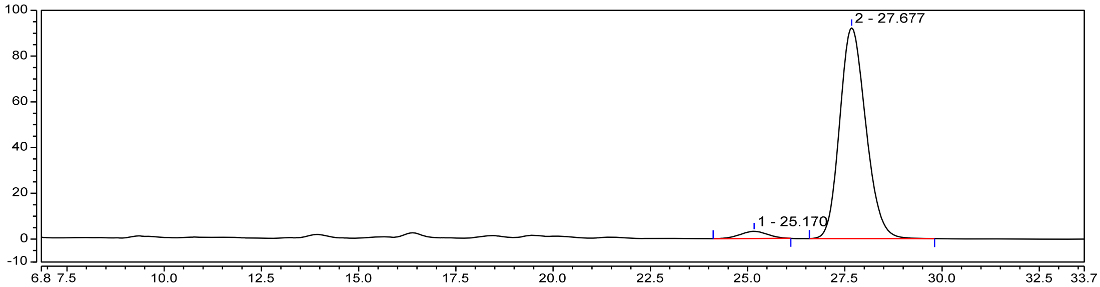

Entry	Retention Time	Area	Height	%Area

1	25.170	2.5971	3.13	3.49
2	27.677	71.8583	92.13	96.51

Enantiomerically enriched **2an** (In neat)

Supplementary Figure 82. HPLC spectra for compound **2an**

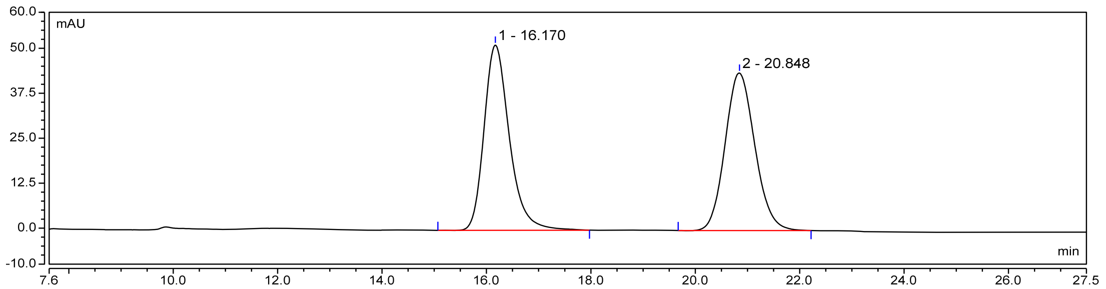

Entry	Retention Time	Area	Height	%Area
1	16.170	28.9476	51.50	50.02
2	20.848	28.9287	43.80	49.98

Racemic **2ao**

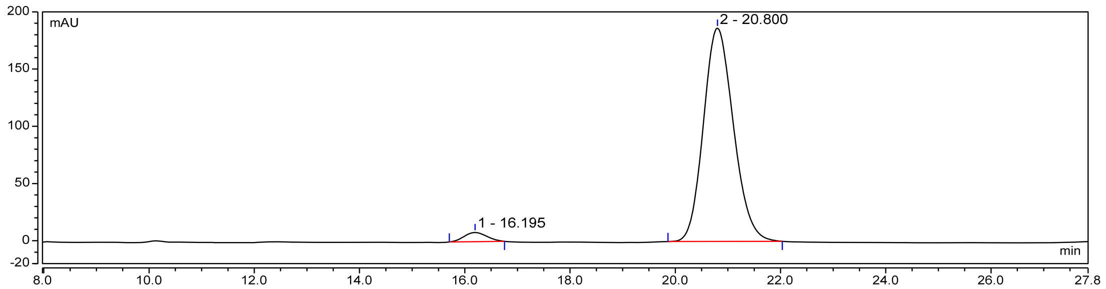

Entry	Retention Time	Area	Height	%Area
1	16.195	4.0183	8.04	3.16
2	20.800	123.2259	186.38	96.84

Enantiomerically enriched **2ao** (In PhCl)

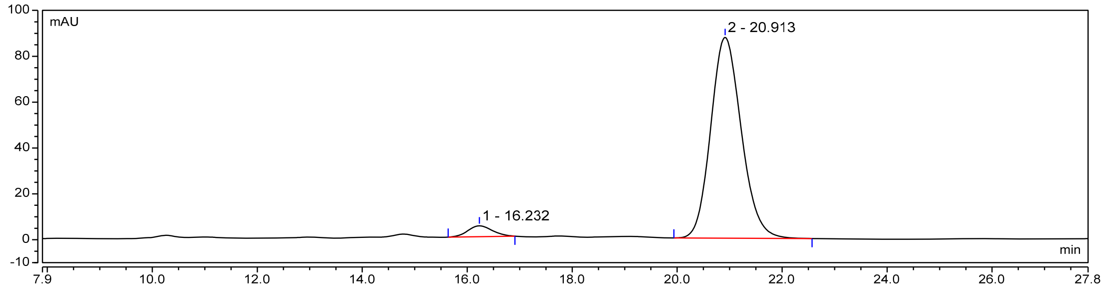

Entry	Retention Time	Area	Height	%Area
1	16.232	2.5583	4.73	4.15
2	20.913	59.1451	87.57	95.85

Enantiomerically enriched **2ao** (In neat)

Supplementary Figure 83. HPLC spectra for compound **2ao**

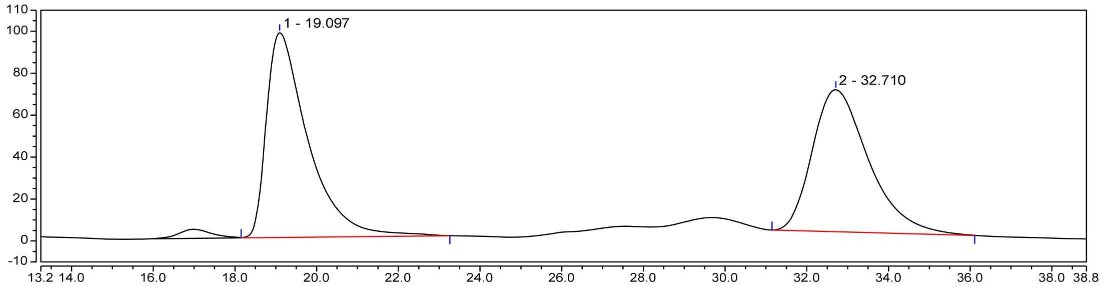

Entry	Retention Time	Area	Height	%Area
1	19.097	114.9233	97.61	51.51
2	32.710	108.1731	67.70	48.49

Racemic **2ap**

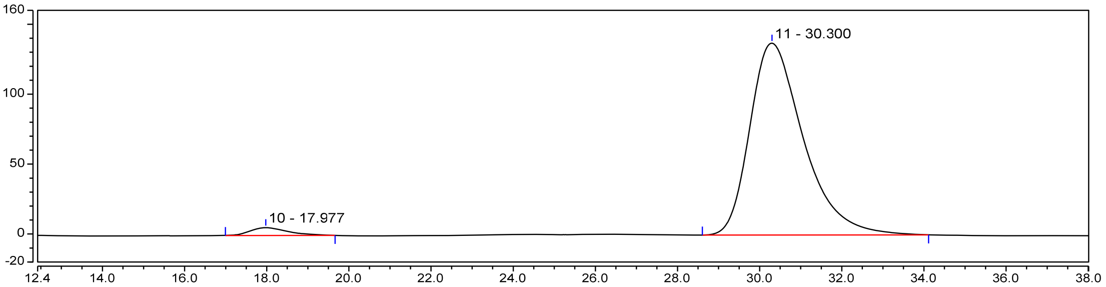

Entry	Retention Time	Area	Height	%Area
1	17.977	5.8992	5.57	2.77
2	30.300	207.3764	137.27	97.23

Enantiomerically enriched **2ap** (In PhCl)

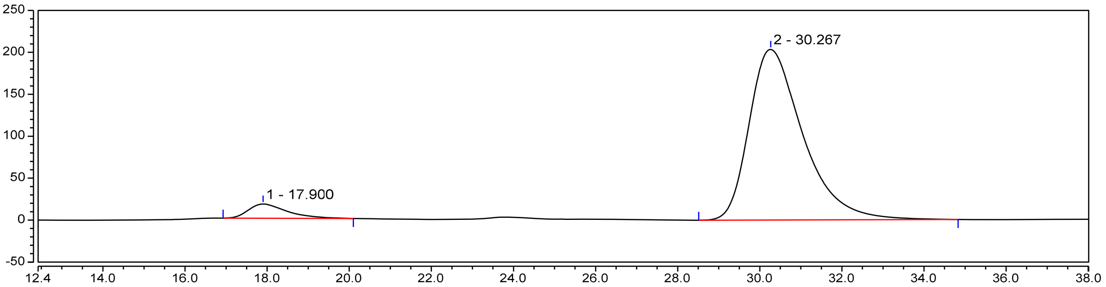

Entry	Retention Time	Area	Height	%Area
1	17.900	18.4598	16.92	5.64
2	30.267	308.9028	203.47	94.36

Enantiomerically enriched **2ap** (In neat)

Supplementary Figure 84. HPLC spectra for compound **2ap**

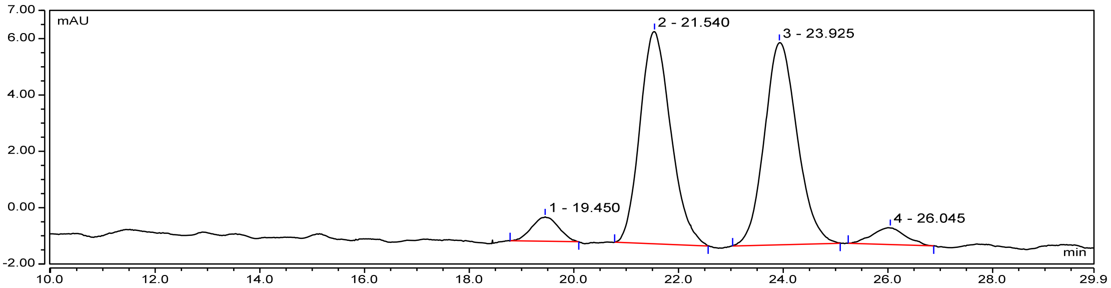

Entry	Retention Time	Area	Height	%Area
1	19.450	0.5114	0.87	4.53
2	21.540	5.1309	7.54	45.49
3	23.925	5.2024	7.18	46.12

4	26.045	0.4349	0.60	3.86

Racemic **3**

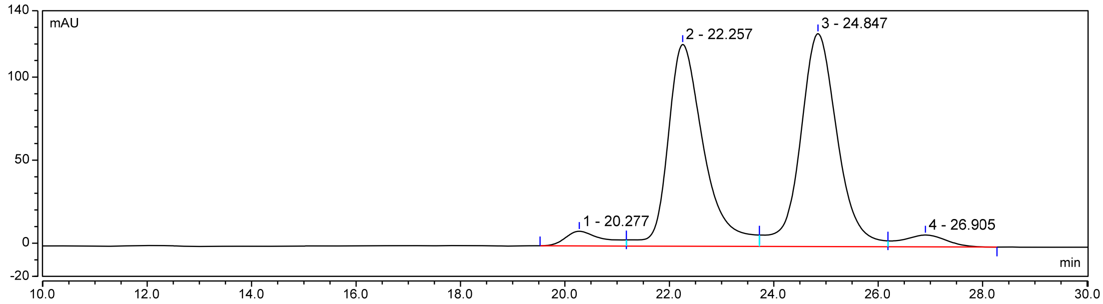

Entry	Retention Time	Area	Height	%Area
1	20.277	7.3757	8.80	3.40
2	22.257	97.2602	121.50	44.82
3	24.847	105.4337	128.23	48.58
4	26.905	6.9552	7.13	3.20

Enantiomerically enriched **3** (In PhCl)

Supplementary Figure 85. HPLC spectra for compound **3**

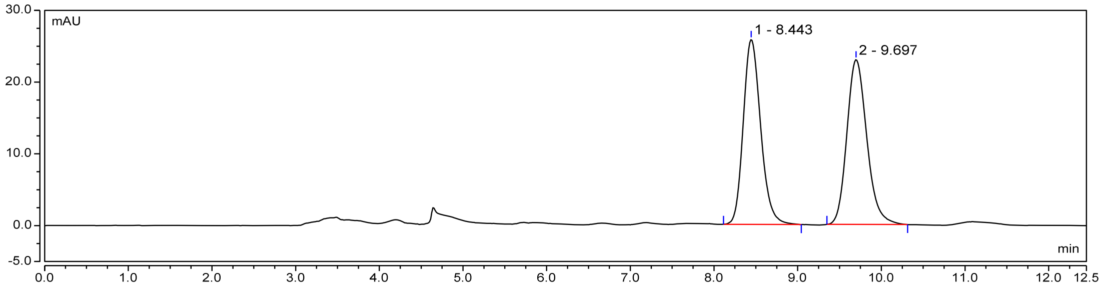

Entry	Retention Time	Area	Height	%Area
1	8.443	6.2212	25.79	49.52
2	9.697	6.3421	22.97	50.48

Racemic **7a**

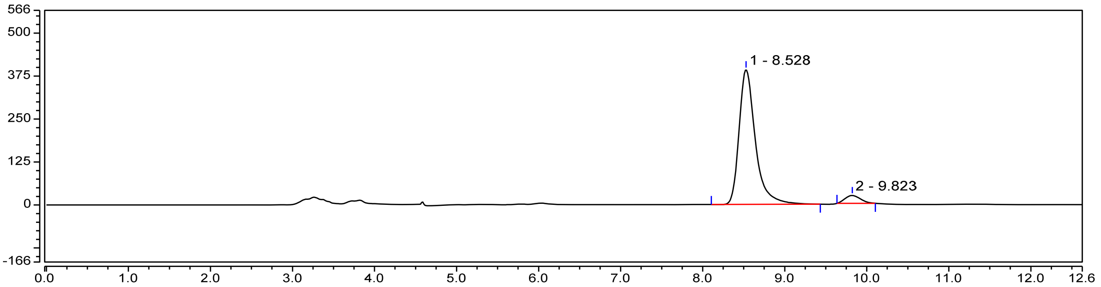

Entry	Retention Time	Area	Height	%Area
1	8.528	90.3712	391.86	94.76
2	9.823	5.0014	22.97	5.24

Enantiomerically enriched **7a** (In PhCl)

Supplementary Figure 86. HPLC spectra for compound **7a**

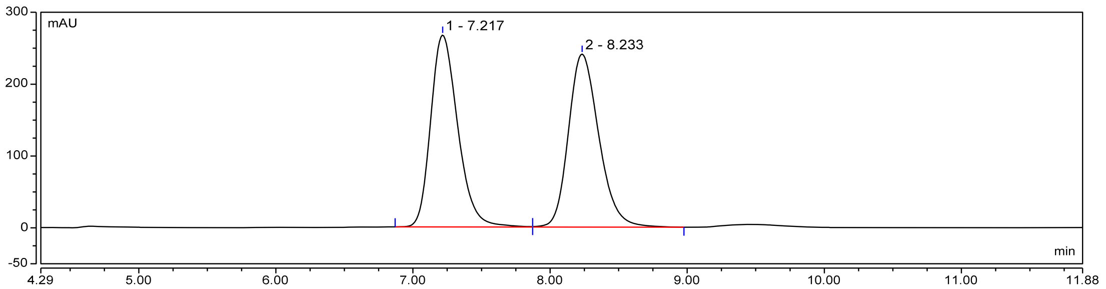

Entry	Retention Time	Area	Height	%Area
1	7.217	61.2072	266.92	49.73
2	8.233	61.8698	240.69	50.27

Racemic **7b**

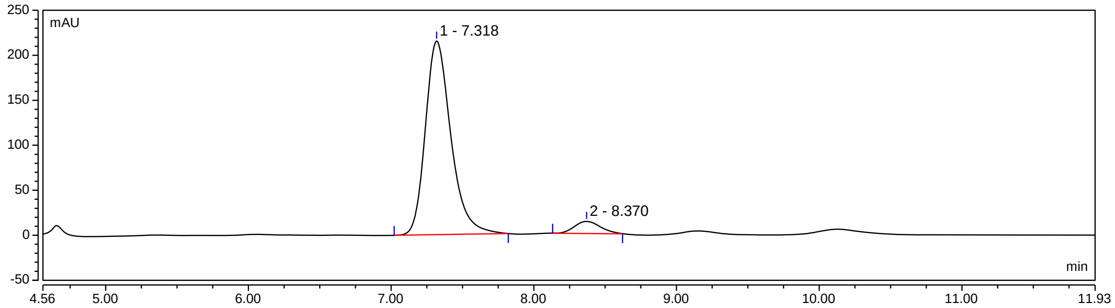

Entry	Retention Time	Area	Height	%Area
1	7.318	44.6757	215.21	94.06
2	8.370	2.8213	13.50	5.94

Enantiomerically enriched **7b** (In PhCl)

Supplementary Figure 87. HPLC spectra for compound **7b**

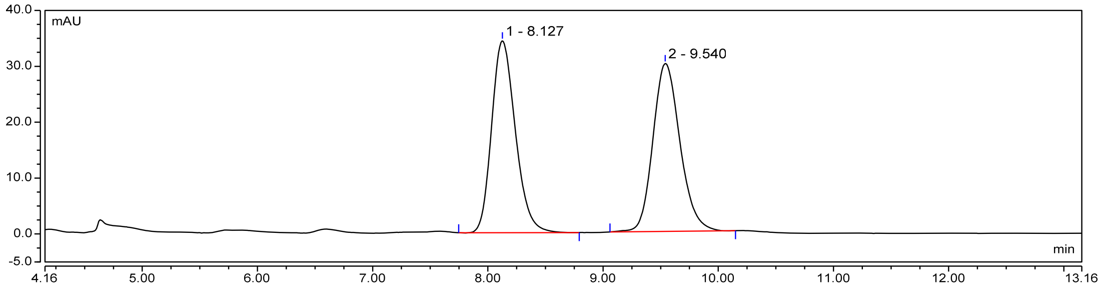

Entry	Retention Time	Area	Height	%Area
1	8.127	8.2114	34.35	50.12
2	9.540	8.1706	30.04	49.88

Racemic **7c**

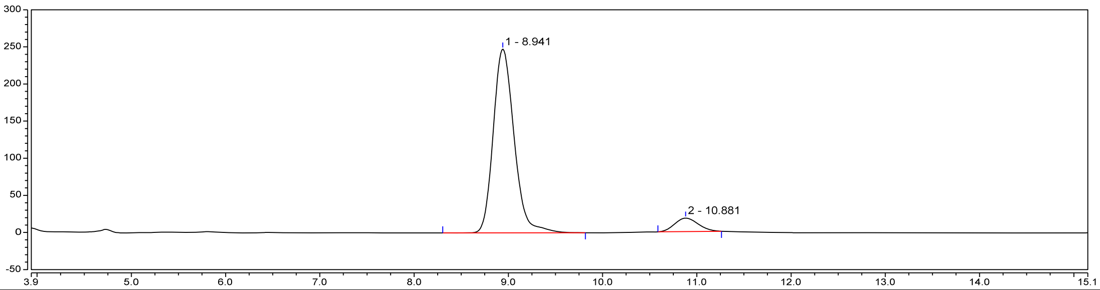

Entry	Retention Time	Area	Height	%Area
1	8.941	63.57	247.50	92.37

2	10.881	5.245	18.194	7.62

Enantiomerically enriched **7c** (In PhCl)

Supplementary Figure 88. HPLC spectra for compound **7c**

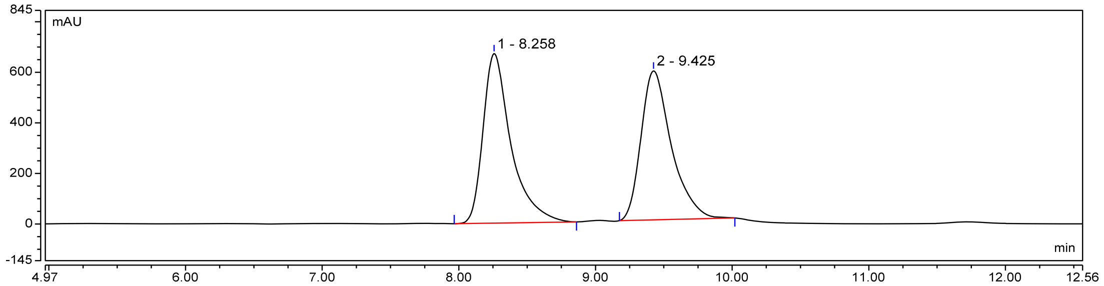

Entry	Retention Time	Area	Height	%Area
1	8.258	158.6270	670.20	51.12
2	9.425	151.7018	588.91	48.88

Racemic **7d**

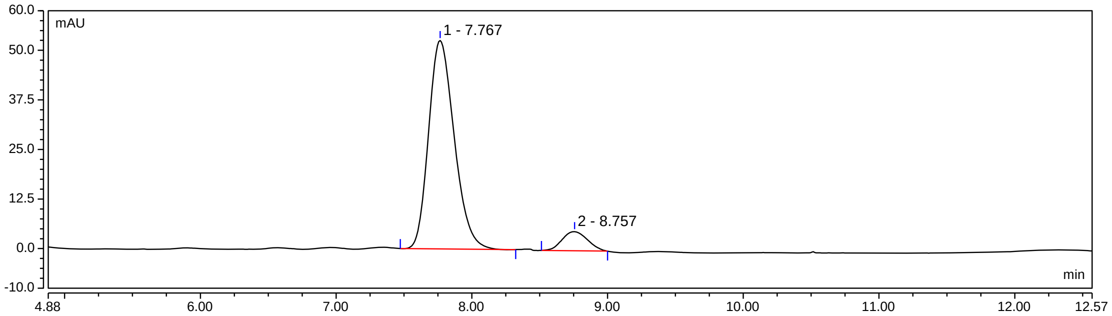

Entry	Retention Time	Area	Height	%Area
1	7.767	11.5168	52.57	91.69
2	8.757	1.0434	4.83	8.31

Enantiomerically enriched **7d** (In PhCl)

Supplementary Figure 89. HPLC spectra for compound **7d**

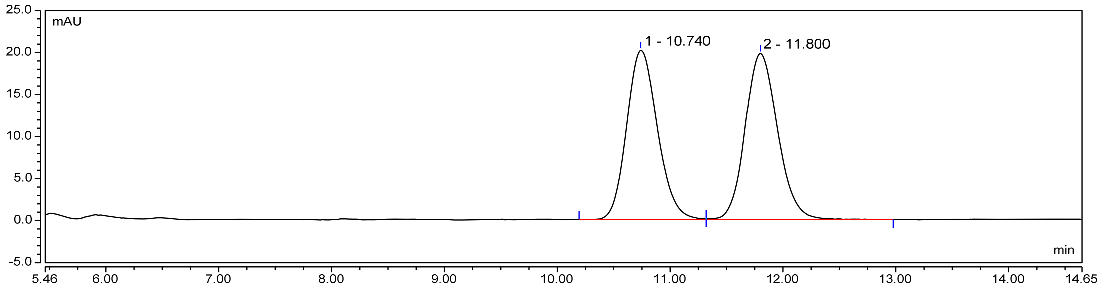

Entry	Retention Time	Area	Height	%Area
1	10.740	6.3589	20.11	49.20
2	11.800	6.5647	19.71	50.80

Racemic **7e**

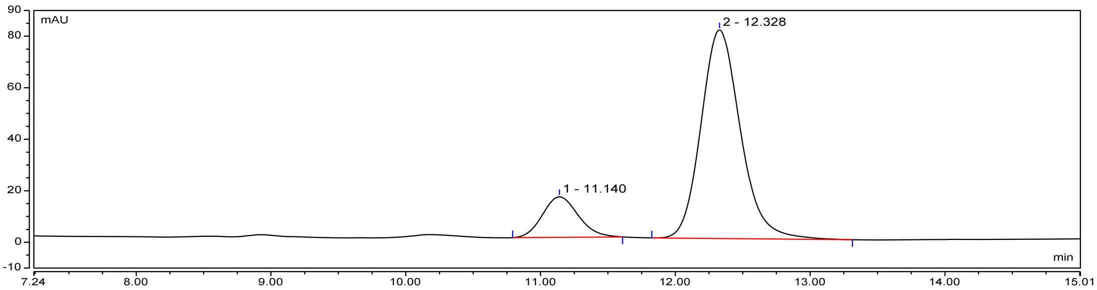

Entry	Retention Time	Area	Height	%Area
1	11.140	4.8400	15.75	14.96
2	12.328	27.5058	80.96	85.04

Enantiomerically enriched **7e** (In PhCl)

Supplementary Figure 90. HPLC spectra for compound **7e**

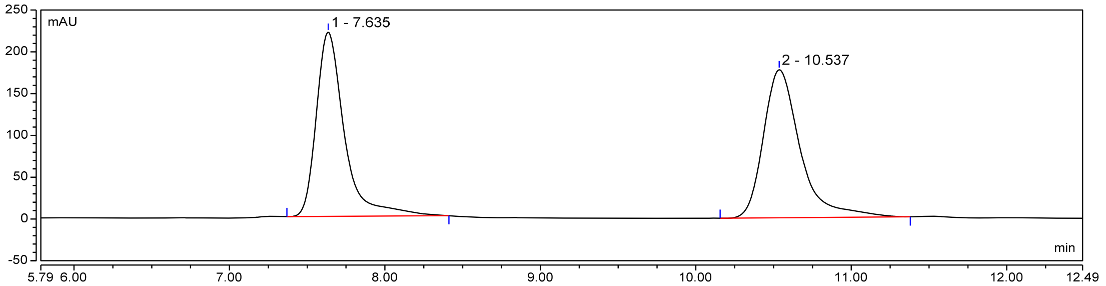

Entry	Retention Time	Area	Height	%Area
1	7.635	46.1763	220.42	48.63
2	10.537	48.7759	177.19	51.37

Racemic **7f**

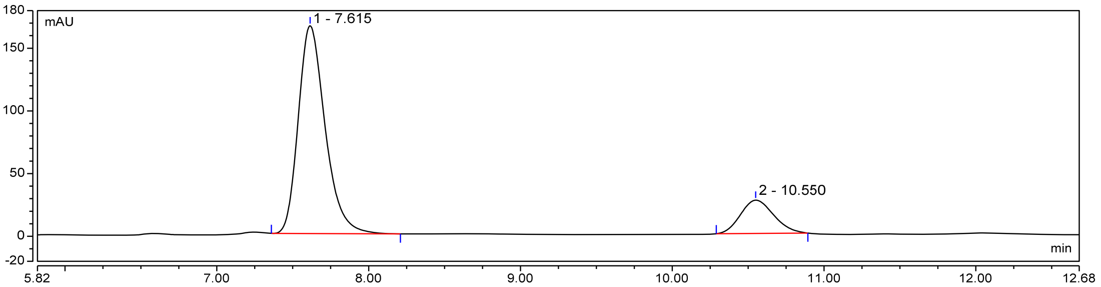

Entry	Retention Time	Area	Height	%Area
1	7.615	34.0349	165.74	83.47
2	10.550	6.7410	26.57	16.53

Enantiomerically enriched **7f** (In PhCl)

Supplementary Figure 91. HPLC spectra for compound **7f**

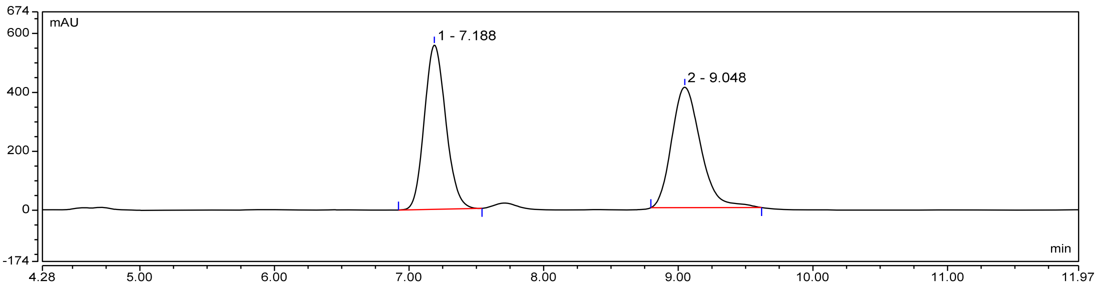

Entry	Retention Time	Area	Height	%Area
1	7.188	102.4449	557.34	50.05
2	9.048	102.2305	408.43	49.95

Racemic **8**

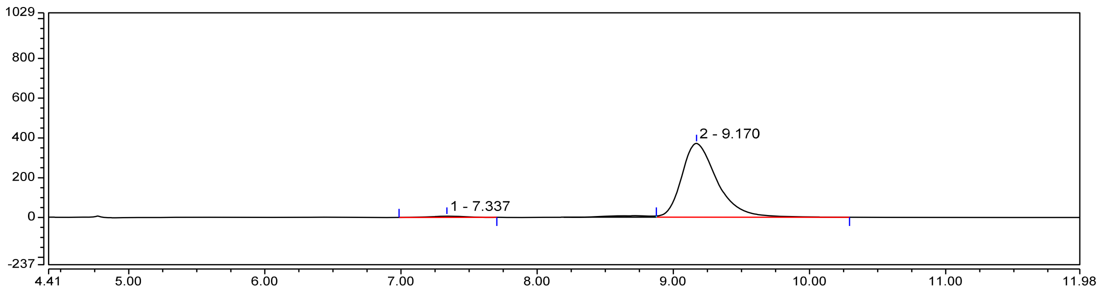

Entry	Retention Time	Area	Height	%Area
1	7.337	1.6084	6.81	1.41
2	9.170	112.3119	371.35	98.59

Enantiomerically enriched **8**

Supplementary Figure 92. HPLC spectra for compound **8**

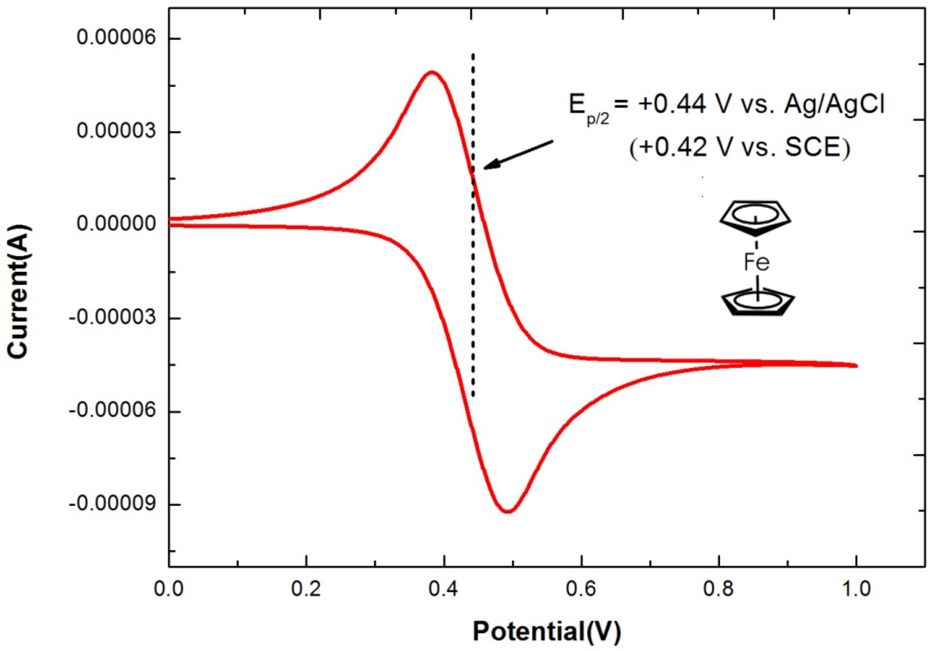

Supplementary Figure 93. Cyclic voltammogram of Ferrocene.

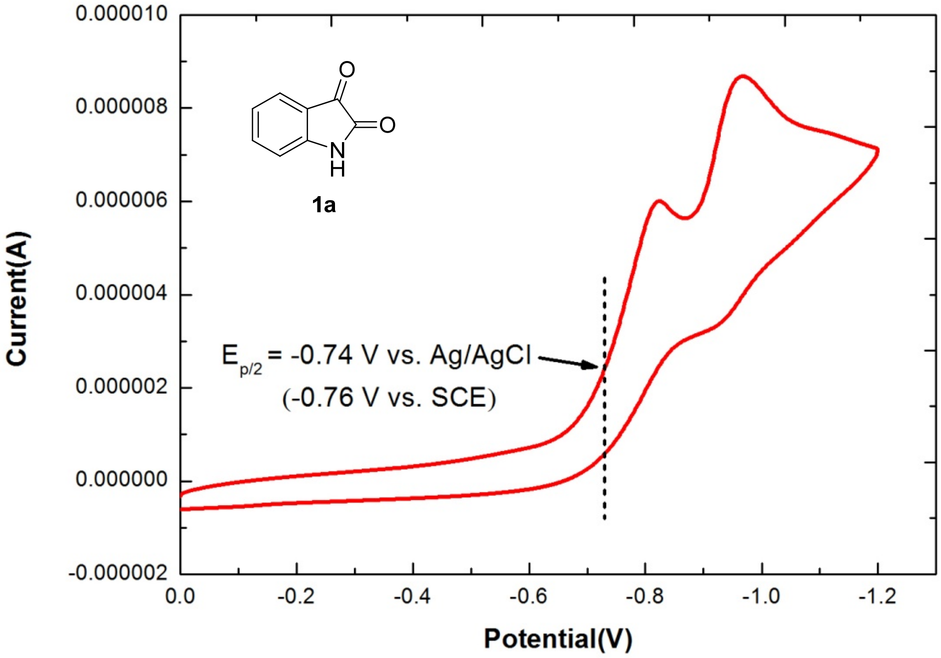

Supplementary Figure 94. Cyclic voltammogram of **1a**.

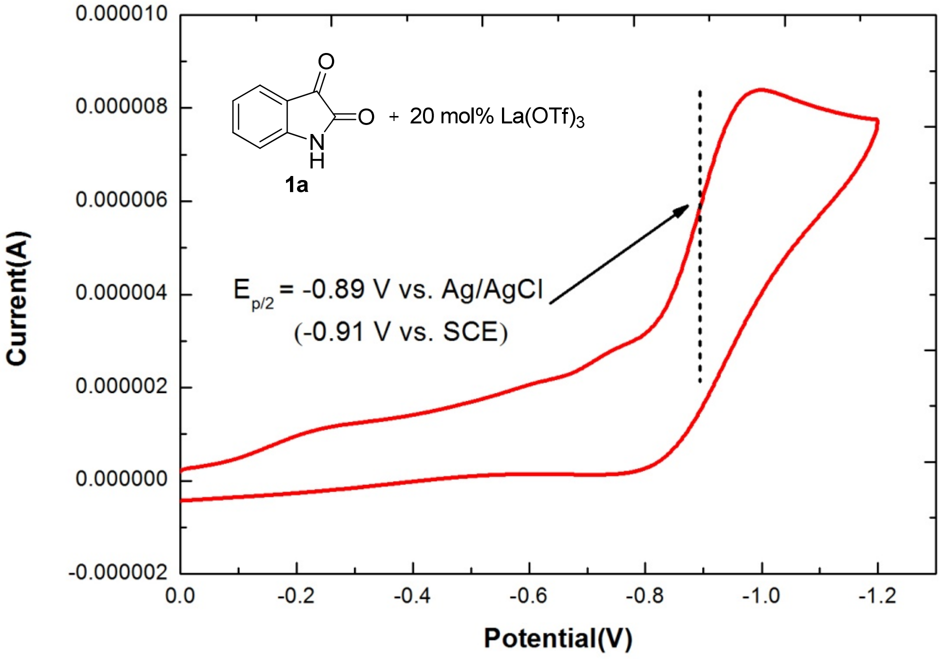

Supplementary Figure 95. Cyclic voltammogram of [**1a**+$La(OTf)_3$].

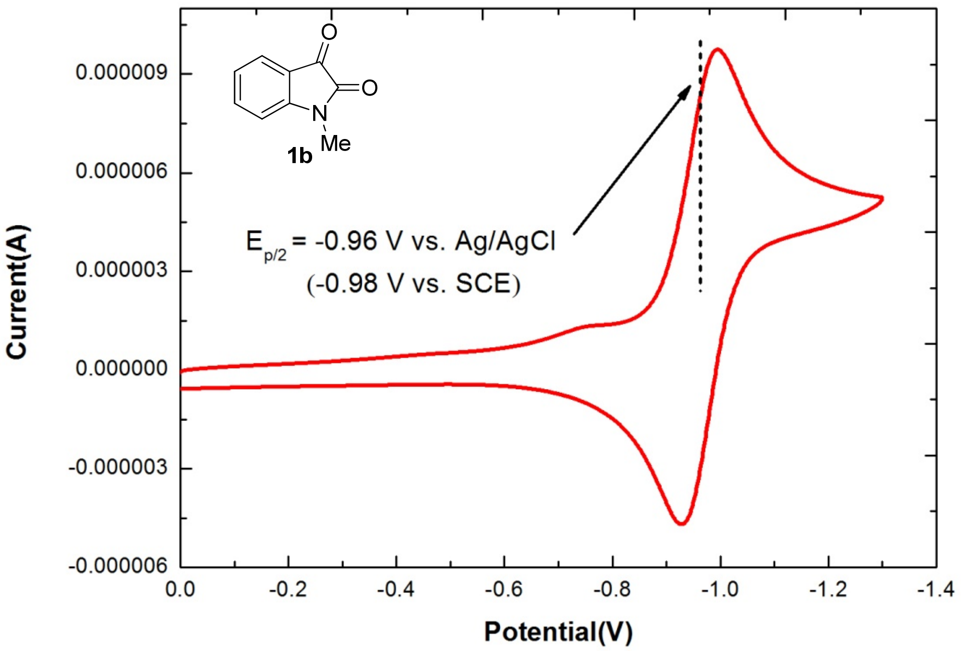

Supplementary Figure 96. Cyclic voltammogram of **1b**.

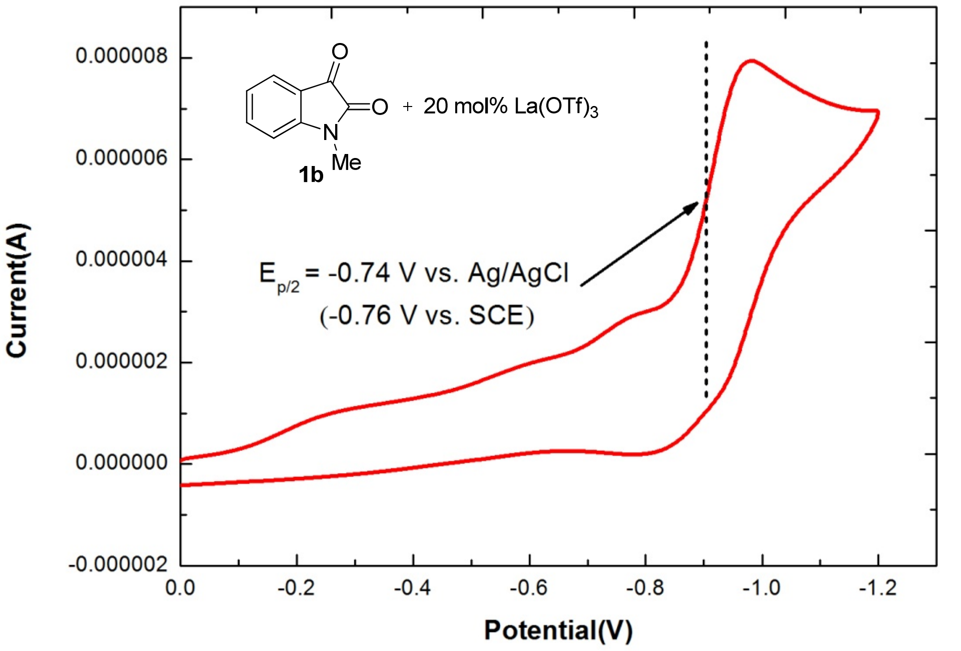

Supplementary Figure 97. Cyclic voltammogram of [**1b**+La(OTf)$_3$].

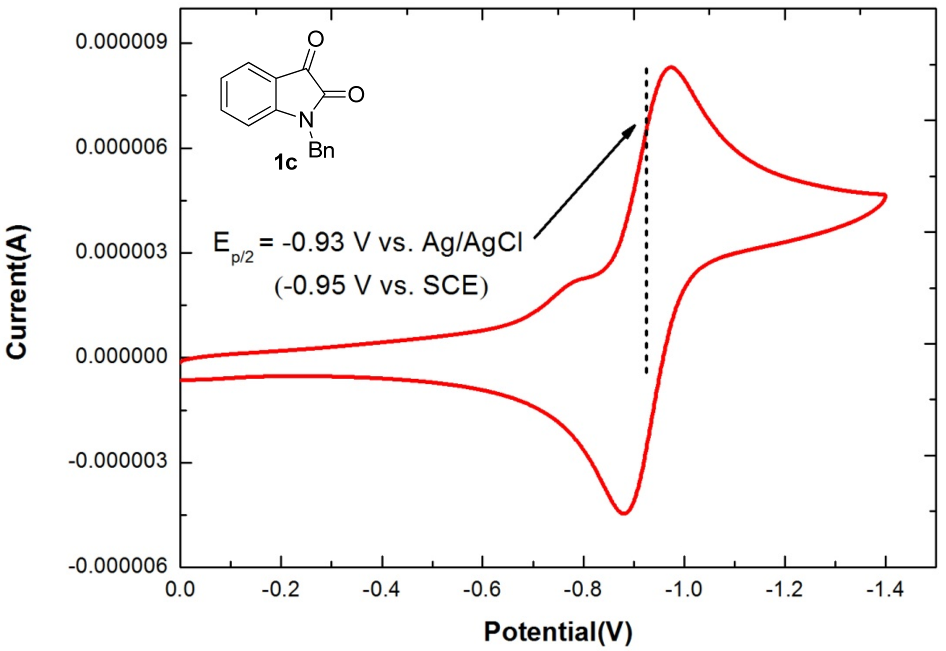

Supplementary Figure 98. Cyclic voltammogram of **1c**.

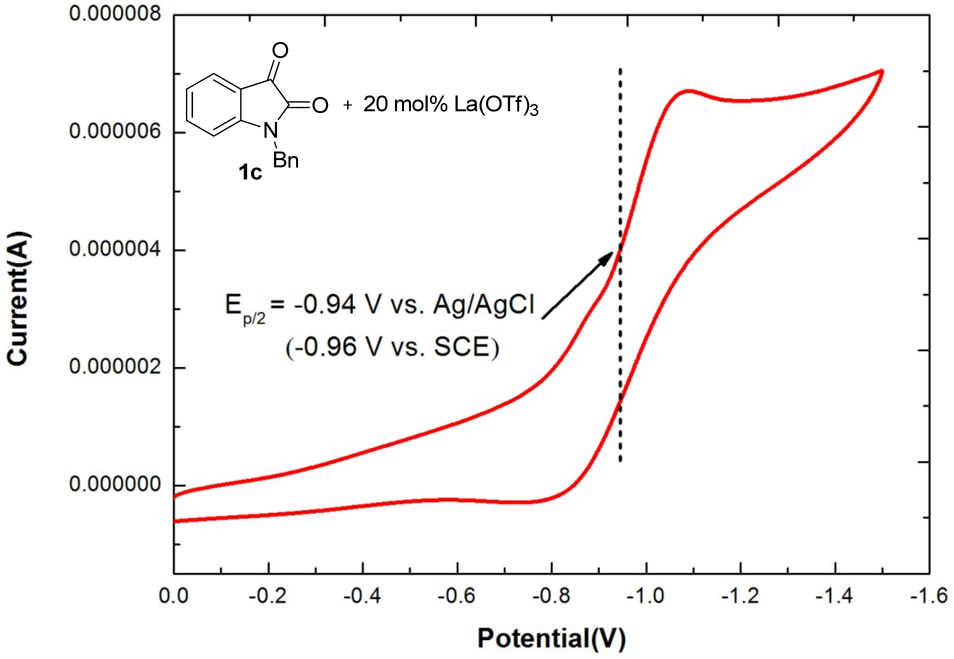

Supplementary Figure 99. Cyclic voltammogram of [**1c**+La(OTf)$_3$].

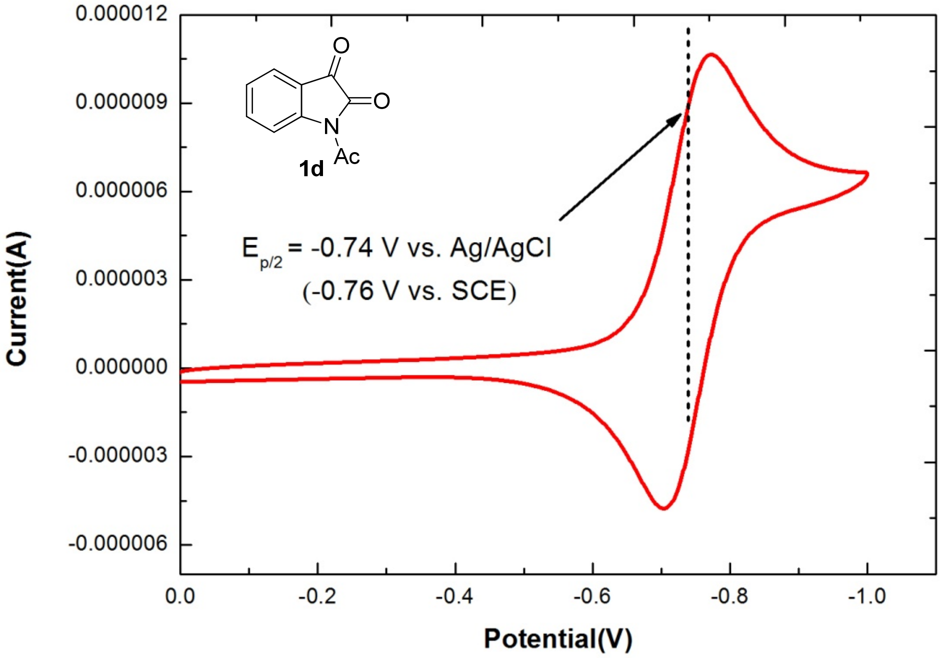

Supplementary Figure 100. Cyclic voltammogram of **1d**.

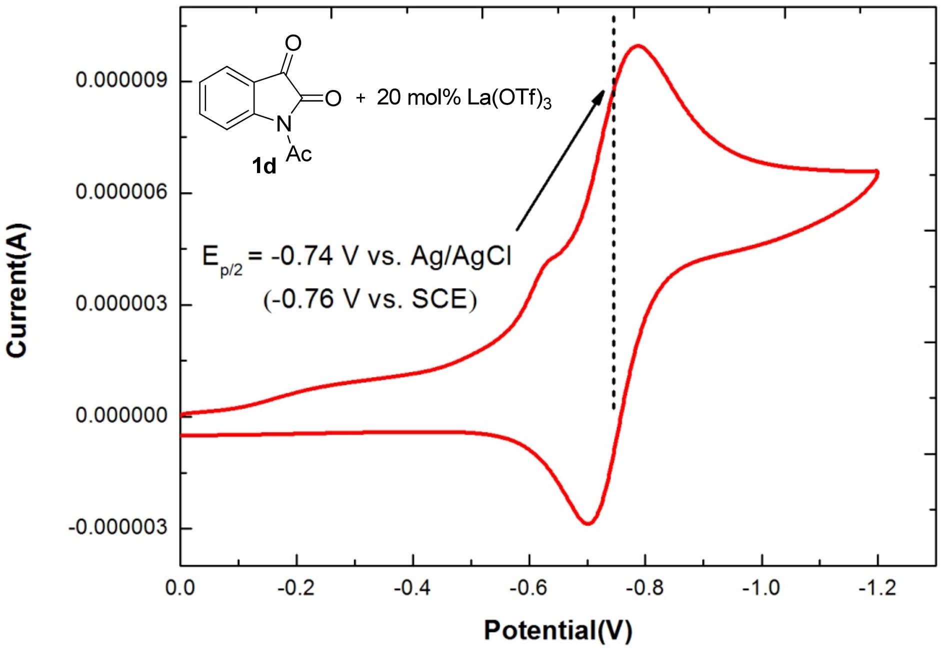

Supplementary Figure 101. Cyclic voltammogram of [**1d**+La(OTf)$_3$].

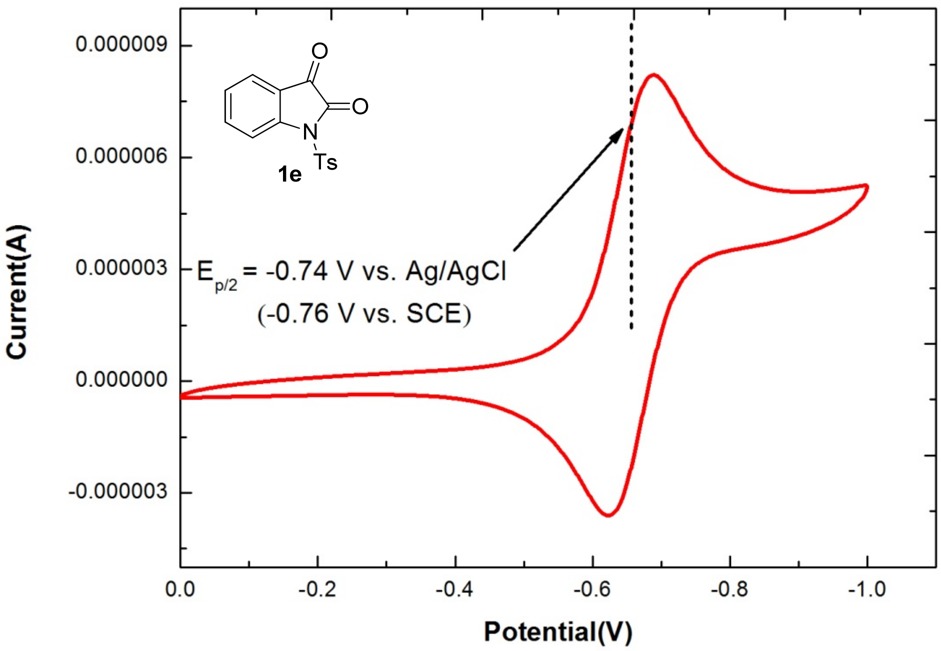

Supplementary Figure 102. Cyclic voltammogram of **1e**.

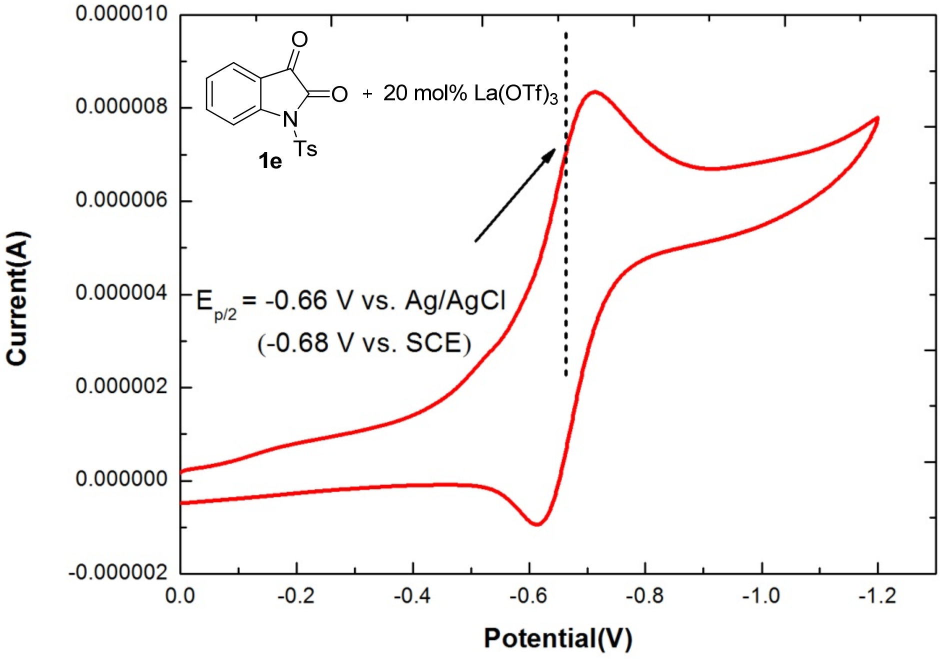

Supplementary Figure 103. Cyclic voltammogram of [**1e**+La(OTf)$_3$].

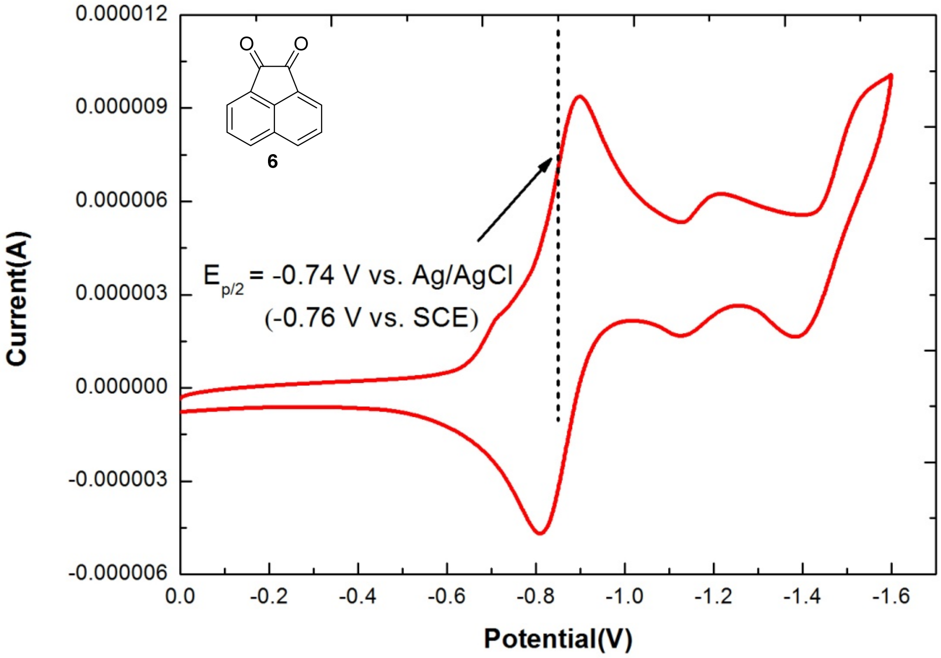

Supplementary Figure 104. Cyclic voltammogram of **6**.

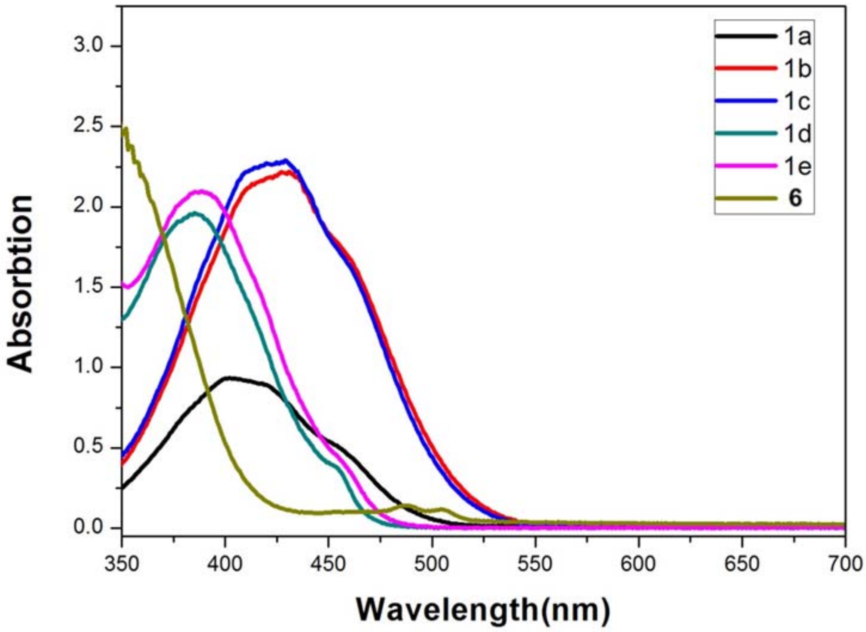

Supplementary Figure 105. UV-vis absorption spectrum of **1a-1e** and **6** in PhCl (1×10-3 M) at 25 ^{o}C.

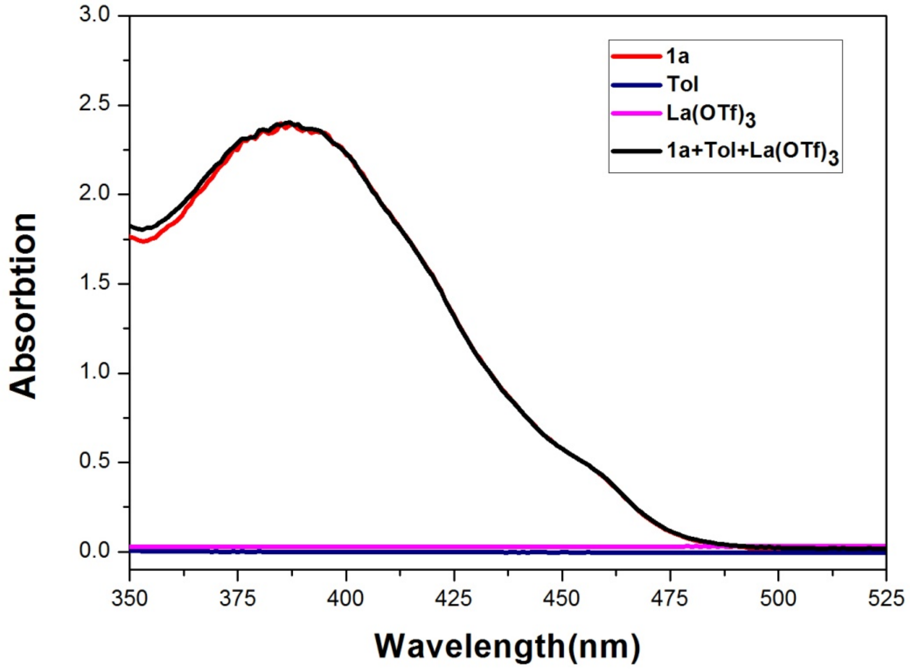

Supplementary Figure 106. UV-vis absorption spectrum of EDA experiment.

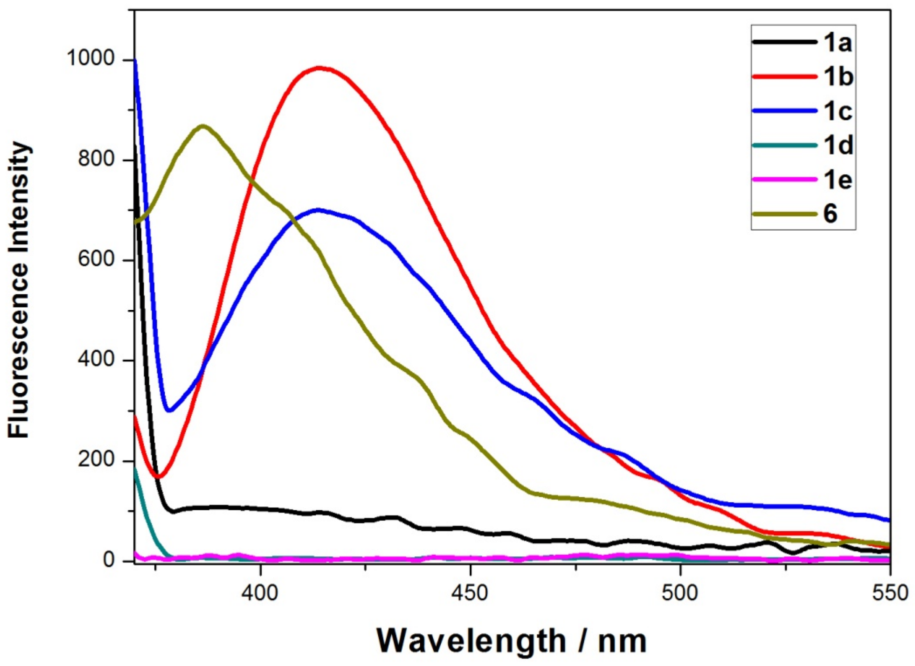

Supplementary Figure 107. Fluorescence spectra of **1a-1e** and **6** in DCM (1×10^{-3} M) at room temperature.

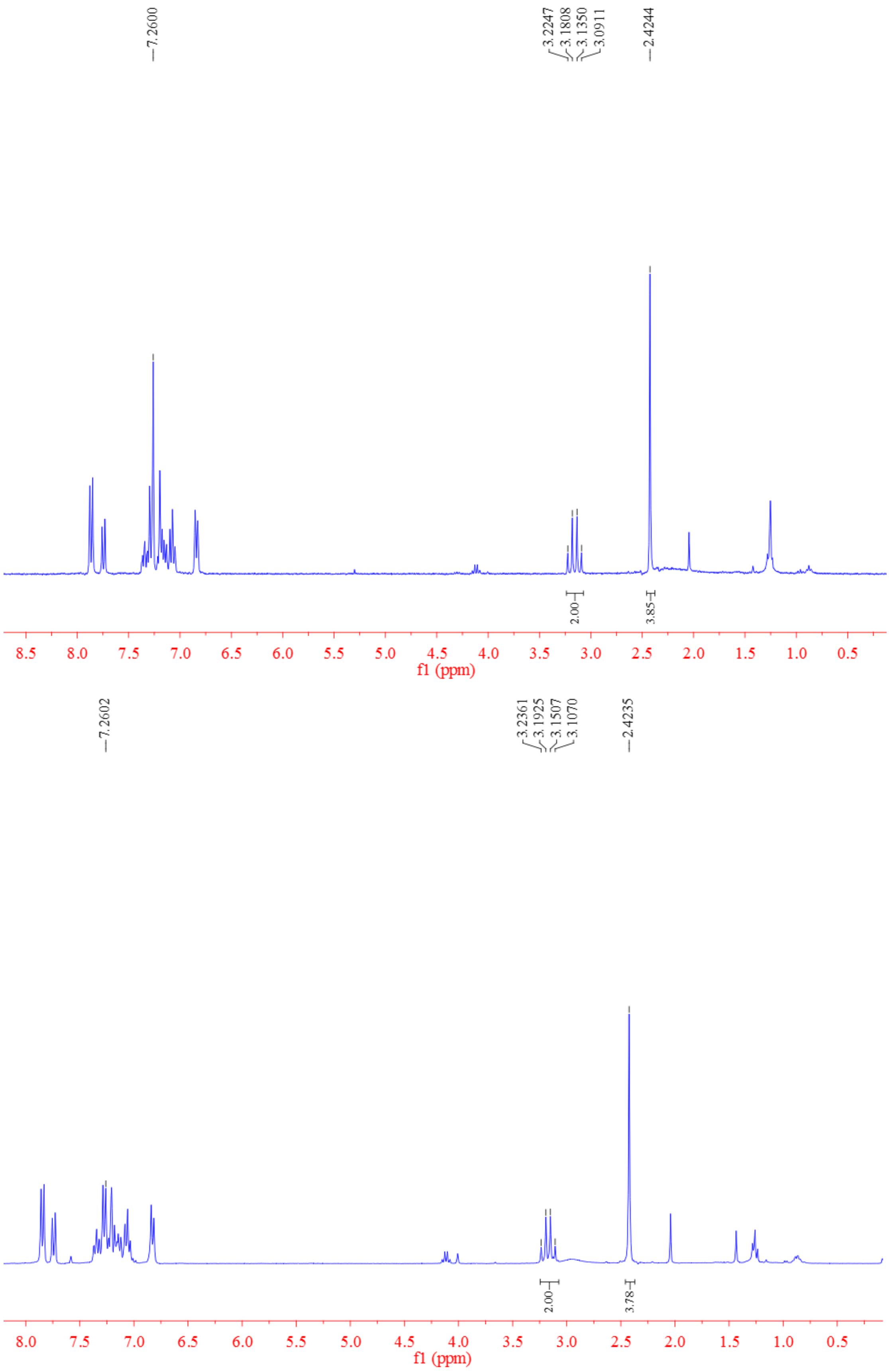

Supplementary Figure 108. ^{1}HNMR of intermolecular competition KIE experiment.

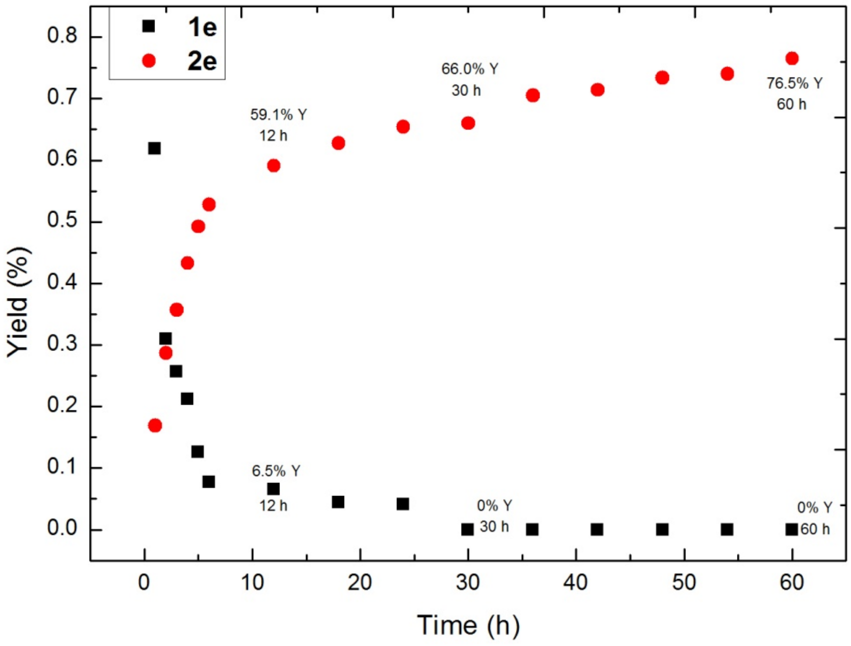

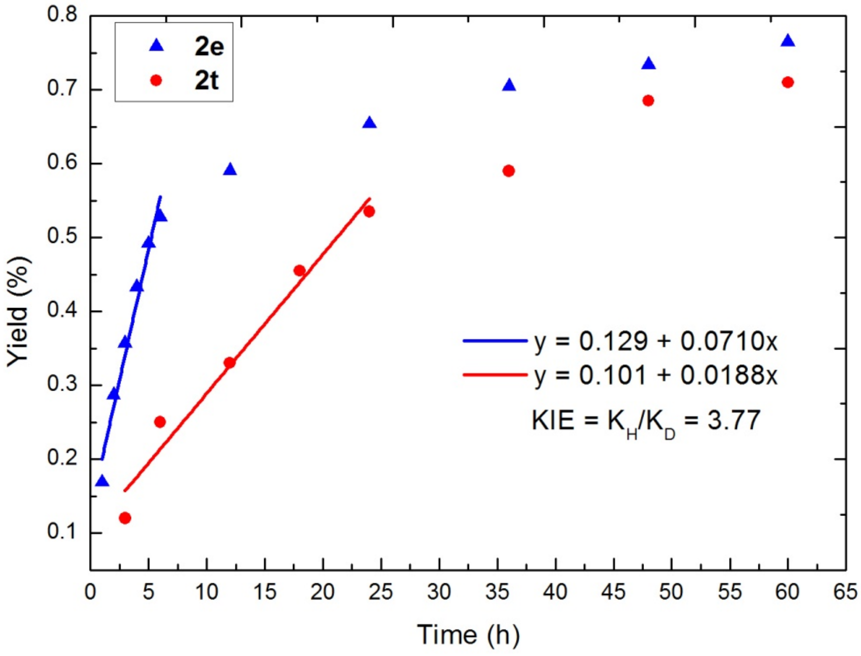

Supplementary Figure 109. Reaction time-course data for independent rate KIE. (Upper: the corresponding isolated yields of **1e** and **2e** at different times; Below: KIE determination before 6 h)

Supplementary Note 1

Supplementary Table 1. Optimization of reaction conditions (toluene as solvent)[a]

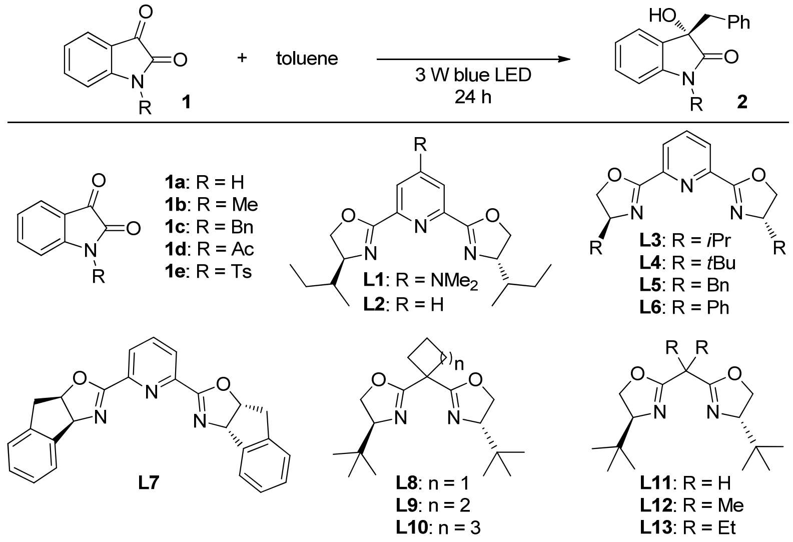

entry	1	LA (mol%)	L (mol%)	additive (mg)	T (°C)	x mL of toluene	2	ee (%)[b]
1	**1a**	--	--	--	25	2.0	**2a**	N.A.[c]
2	**1b**	--	--	--	25	2.0	**2b**	N.A.[d]
3	**1c**	--	--	--	25	2.0	**2c**	N.A.[d]
4	**1d**	--	--	--	25	2.0	**2d**	N.A.[e]
5	**1e**	--	--	--	25	2.0	**2e**	N.A.[f]
6	**1c**	$Yb(OTf)_3$ (10)	**L3** (11)	--	25	2.0	**2e**	5
7	**1b**	$Yb(OTf)_3$ (10)	**L3** (11)	--	25	2.0	**2e**	13
8	**1b**	$Yb(OTf)_3$ (10)	**L3** (11)	4Å MS (70)	25	2.0	**2e**	35
9	**1b**	$Yb(OTf)_3$ (10)	**L3** (11)	3Å MS (70)	25	2.0	**2e**	31
10	**1b**	$Yb(OTf)_3$ (10)	**L3** (11)	5Å MS (70)	25	2.0	**2e**	27
11	**1b**	$Sc(OTf)_3$ (10)	**L3** (11)	4Å MS (70)	25	2.0	**2e**	0
12	**1b**	$Gd(OTf)_3$ (10)	**L3** (11)	4Å MS (70)	25	2.0	**2e**	25
13	**1b**	$Nd(OTf)_3$ (10)	**L3** (11)	4Å MS (70)	25	2.0	**2e**	27
14	**1b**	$Ce(OTf)_3$ (10)	**L3** (11)	4Å MS (70)	25	2.0	**2e**	27
15	**1b**	$Eu(OTf)_3$ (10)	**L3** (11)	4Å MS (70)	25	2.0	**2e**	38
16	**1b**	$La(OTf)_3$ (10)	**L3** (11)	4Å MS (70)	25	2.0	**2e**	82
17	**1b**	$Cu(OTf)_2$ (10)	**L3** (11)	4Å MS (70)	25	2.0	**2e**	7
18	**1b**	$Zn(OTf)_2$ (10)	**L3** (11)	4Å MS (70)	25	2.0	**2e**	16
19	**1b**	$Mg(OTf)_2$ (10)	**L3** (11)	4Å MS (70)	25	2.0	**2e**	11
20	**1b**	$Ni(OTf)_2$ (10)	**L3** (11)	4Å MS (70)	25	2.0	**2e**	5
21	**1b**	$Ba(OTf)_2$ (10)	**L3** (11)	4Å MS (70)	25	2.0	**2e**	9
22	**1b**	AgOTf (10)	**L3** (11)	4Å MS (70)	25	2.0	**2e**	0
23	**1b**	$La(OTf)_3$ (10)	**L4** (11)	4Å MS (70)	25	2.0	**2e**	41
24	**1b**	$La(OTf)_3$ (10)	**L5** (11)	4Å MS (70)	25	2.0	**2e**	67
25	**1b**	$La(OTf)_3$ (10)	**L6** (11)	4Å MS (70)	25	2.0	**2e**	75
26	**1b**	$La(OTf)_3$ (10)	**L7** (11)	4Å MS (70)	25	2.0	**2e**	32
27	**1b**	$La(OTf)_3$ (10)	**L8** (11)	4Å MS (70)	25	2.0	**2e**	19
28	**1b**	$La(OTf)_3$ (10)	**L9** (11)	4Å MS (70)	25	2.0	**2e**	23
29	**1b**	$La(OTf)_3$ (10)	**L10** (11)	4Å MS (70)	25	2.0	**2e**	21
30	**1b**	$La(OTf)_3$ (10)	**L11** (11)	4Å MS (70)	25	2.0	**2b**	0
31	**1b**	$La(OTf)_3$ (10)	**L12** (11)	4Å MS (70)	25	2.0	**2e**	21
32	**1b**	$La(OTf)_3$ (10)	**L13** (11)	4Å MS (70)	25	2.0	**2e**	23

33	**1b**	La(OTf)$_3$ (10)	**L2** (11)	4Å MS (70)	25	2.0	**2e**	90
34	**1b**	La(OTf)$_3$ (10)	**L2** (11)	4Å MS (70)	40	2.0	**2e**	51
35	**1b**	La(OTf)$_3$ (10)	**L2** (11)	4Å MS (70)	10	2.0	**2e**	90[g]
36	**1b**	La(OTf)$_3$ (10)	**L2** (11)	4Å MS (90)	25	2.0	**2e**	86
37	**1b**	La(OTf)$_3$ (10)	**L2** (11)	4Å MS (50)	25	2.0	**2e**	85
38	**1b**	La(OTf)$_3$ (10)	**L2** (11)	4Å MS (70)	25	3.0	**2e**	90
39	**1b**	La(OTf)$_3$ (10)	**L2** (11)	4Å MS (70)	25	1.0	**2e**	81
40	**1b**	La(OTf)$_3$ (10)	**L2** (11)	4Å MS (70)	25	2.0	**2e**	77[h]
41	**1b**	La(OTf)$_3$ (10)	**L2** (11)	4Å MS (70)	25	2.0	**2e**	N.A.[i]
42	**1b**	La(OTf)$_3$ (10)	**L2** (11)	4Å MS (70)	25	2.0	**2e**	88[j]
43	**1b**	La(OTf)$_3$ (10)	**L2** (11)	4Å MS (70)	25	2.0	**2e**	85[k]
44	**1b**	La(OTf)$_3$ (15)	**L2** (16)	4Å MS (70)	25	2.0	**2e**	90
45	**1b**	La(OTf)$_3$ (20)	**L2** (22)	4Å MS (70)	25	2.0	**2e**	92
46	**1b**	La(OTf)$_3$ (20)	**L1** (22)	4Å MS (70)	25	2.0	**2e**	98[l]

[a]The reactions were performed on a 0.05 mmol scale using a 3 W blue LED (450-455 nm). [b]Determined by HPLC analysis on a chiral stationary phase. [c]The reaction worked in ~10% chemical conversion determined by TLC analysis. The reaction delivered a complex crude mixture and the desired product **2a** could not be obtained. [d]no reaction was observed. [e]~50% conversion, 28% yield. [f]~50% conversion, 24% yield. [g]Reaction became sluggish. [h]3W violet LED (410-420 nm) was used. [i]3W green LED (540-550 nm) was used. [j]3W white LED was used. [k]23 W CFL was used. [l]t = 60 h, 71% yield.

Supplementary Table 2. Optimization of reaction conditions neat in toluene[a]

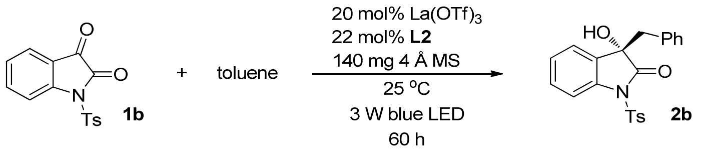

entry	solvent (mL)	equiv. of toluene	ee (%)[b]	yield (%)[c]
1	DCM (4.0)	30	95	11
2	CPME (4.0)	30	N.A.[d]	N.A.
3	MTBE (4.0)	30	93	24
4	DME (4.0)	30	N.A.[d]	N.A.
5	THF (4.0)	30	N.A.[d]	N.A.
6	ethyl acetate (4.0)	30	N.A.[d]	N.A.
7	*t*BuPh (4.0)	30	93	13
8	PhCl (4.0)	30	91	47
9	PhBr (4.0)	30	78	19
10	PhCl (4.0)	50	91	52
11	PhCl (4.0)	100	90	67
12[f]	PhCl (4.0)	50	98	58

[a]The reactions were performed on a 0.1 mmol scale using a 3 W blue LED (450-455 nm). [b]Determined by HPLC analysis on a chiral stationary phase. [c]Isolated yield. [d]Reaction is messy. [f]**L1** was used.

Supplementary Table 3. Optimization of reaction conditions with respect to acenaphthoquinone[a]

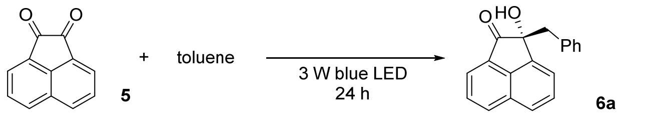

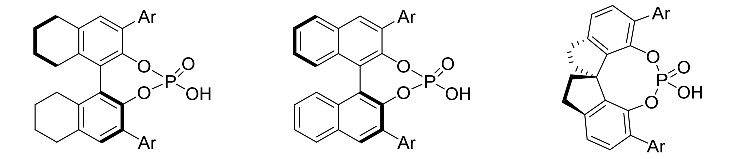

C1: Ar = 3,5-$Ph_2C_6H_3$
C2: Ar = 3,5-$(CF_3)_2C_6H_3$
C3: Ar = 2-naphthyl
C4: Ar = 4-pyrene
C5: Ar = 3,5-$t$$Bu_2C_6H_3$
C6: Ar = 4-$MeOC_6H_4$
C7: Ar = 4-PhC_6H_4
C8: Ar = 3,5-[(3,5-Ph_2)C_6H_3]$_2C_6H_3$
C9: Ar = 4-tBuPh
C10: Ar = 4-[(3,5-Ph_2)C_6H_3]C_6H_3

C11: Ar = 3,5-$i$$Pr_2C_6H_3$
C12: Ar = 9-phenanthryl
C13: Ar = 2-naphthyl
C14: Ar = 3,5-$Ph_2C_6H_3$
C15: Ar = 4-(2-naphthyl)C_6H_4
C16: Ar = 4-PhC_6H_4
C17: Ar = 3,5-(4-Ph-Ph)$_2C_6H_4$
C18: Ar = 1-naphthyl
C19: Ar = 3,5-$(NO_2)_2C_6H_4$

C20: Ar = 3,5-$Ph_2C_6H_3$
C21: Ar = 9-phenanthryl
C22: Ar = 3,5-$(CF_3)_2C_6H_3$
C23: Ar = 4-PhC_6H_4
C24: Ar = 2,3-$OMe_2C_6H_3$
C25: Ar = 3,5-$t$$Bu_2C_6H_3$
C26: Ar = 4-$PhOC_6H_4$
C27: Ar = 4-$MeOC_6H_4$
C28: Ar = 4-$i$$PrC_6H_4$
C29: Ar = 2,4,6-$Me_3C_6H_2$
C30: Ar = 3,4,5-$OMe_3C_6H_2$
C31: Ar = 4-pyrene
C32: Ar = 4-$t$$BuC_6H_4$
C33: Ar = 4-(2-naphthyl)C_6H_4

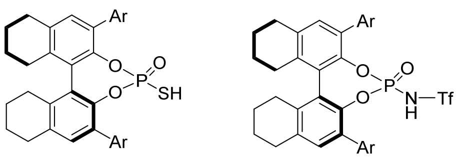

C34: Ar = 3,5-Ph_2Ph
C35: Ar = 3,5-Ph_2Ph

Note: **C** = **CPA** in the serial numbers of chiral catalysts

entry	catalyst (mol%)	*T* (°C)	additive (mg)	x mL of toluene	ee (%)[b]
1	**L1** (22)/La$(OTf)_3$ (20)	25	4Å MS (70)	2.0	57[c]
2	**CPA-1** (10)	25	4Å MS (40)	1.0	77
3	**CPA2** (10)	25	4Å MS (40)	1.0	5
4	**CPA-3** (10)	25	4Å MS (40)	1.0	30
5	**CPA-4** (10)	25	4Å MS (40)	1.0	17
6	**CPA-5** (10)	25	4Å MS (40)	1.0	7
7	**CPA-6** (10)	25	4Å MS (40)	1.0	35
8	**CPA-7** (10)	25	4Å MS (40)	1.0	36
9	**CPA-8** (10)	25	4Å MS (40)	1.0	0
10	**CPA-9** (10)	25	4Å MS (40)	1.0	7
11	**CPA-10** (10)	25	4Å MS (40)	1.0	3
12	**CPA-11** (10)	25	4Å MS (40)	1.0	12
13	**CPA-12** (10)	25	4Å MS (40)	1.0	5
14	**CPA-13** (10)	25	4Å MS (40)	1.0	5
15	**CPA-14** (10)	25	4Å MS (40)	1.0	74
16	**CPA-15** (10)	25	4Å MS (40)	1.0	13
17	**CPA-16** (10)	25	4Å MS (40)	1.0	33
18	**CPA-17** (10)	25	4Å MS (40)	1.0	73
19	**CPA-18** (10)	25	4Å MS (40)	1.0	2
20	**CPA-19** (10)	25	4Å MS (40)	1.0	5
21	**CPA-20** (10)	25	4Å MS (40)	1.0	21
22	**CPA-21** (10)	25	4Å MS (40)	1.0	10
23	**CPA-22** (10)	25	4Å MS (40)	1.0	11
24	**CPA-23** (10)	25	4Å MS (40)	1.0	43
25	**CPA-24** (10)	25	4Å MS (40)	1.0	7
26	**CPA-25** (10)	25	4Å MS (40)	1.0	0
27	**CPA-26** (10)	25	4Å MS (40)	1.0	9

28	**CPA-27** (10)	25	4Å MS (40)	1.0	23
29	**CPA-28** (10)	25	4Å MS (40)	1.0	21
30	**CPA-29** (10)	25	4Å MS (40)	1.0	21
31	**CPA-30** (10)	25	4Å MS (40)	1.0	7
32	**CPA-31** (10)	25	4Å MS (40)	1.0	3
33	**CPA-32** (10)	25	4Å MS (40)	1.0	17
34	**CPA-33** (10)	25	4Å MS (40)	1.0	0
35	**CPA-34** (10)	25	4Å MS (40)	1.0	10
36	**CPA-35** (10)	25	4Å MS (40)	1.0	57
37	**CPA-1** (10)	25	3Å MS (40)	1.0	71
38	**CPA-1** (10)	25	5Å MS (40)	1.0	53
39	**CPA-1** (10)	25	4Å MS (40)	0.8	77
40	**CPA-1** (10)	25	4Å MS (40)	1.5	75
41	**CPA-1** (10)	25	4Å MS (40)	2.0	72
42	**CPA-1** (10)	35	4Å MS (40)	1.0	70
43	**CPA-1** (10)	15	4Å MS (40)	1.0	80
44	**CPA-1** (10)	5	4Å MS (40)	1.0	82
45	**CPA-1** (10)	−5	4Å MS (40)	1.0	84
46	**CPA-1** (10)	−10	4Å MS (40)	1.0	79
47	**CPA-1** (5)	−5	4Å MS (40)	1.0	82
48	**CPA-1** (15)	−5	4Å MS (40)	1.0	85
49	**CPA-1** (20)	−5	4Å MS (40)	1.0	87
50	**CPA-1** (20)	−5	4Å MS (20)	1.0	81
51	**CPA-1** (20)	−5	4Å MS (60)	1.0	82
52	**CPA-1** (20)	−5	4Å MS (40)	1.0	90[d]

[a]The reactions were performed on a 0.05 mmol scale using a 3 W blue LED (450−455 nm). [b]Determined by HPLC analysis on a chiral stationary phase. [c]Toluene saturated with H_2O.[d] Yield = 36.4%. When using 2*3 W blue LEDs (450−455 nm): yield = 53.2%, ee = 90%.

Supplementary Note 2

Mechanism studies

Cyclic voltammetry measurement

Cyclic voltammograms of **1a-1e** and **6**: Measurements were performed for anhydrous acetonitrile solutions ([sample] = 1.0 mM, [$(NBu_4)PF_6$] = 0.10 M) with a radium glassy carbon (working electrode) and platinum wire (counter electrode), and a Ag/AgCl reference electrode under N_2 at room temperature. The scan rate was 50 mV/s. Ferrocene (Cp_2Fe) was used as a reference. (Supplementary Figs 93–104).

Supplementary Table 4. Photo- and electro-chemical data of **1a-1e** and **5**

Comp.	λ_{max}^{A} [nm][a]	λ_{max}^{F} [nm][b]	τ *(ns)*[c]	ΔG^{T1} (kcal/mol)	$E_{1/2(red)}$ (V)[d]	$E_{1/2(red)}$* (V)[e]
**1a**	500	--	7.2	58.5	−0.76	+1.78
**1b**	528	422	7.7	53.2	−0.98	+1.33
**1c**	519	420	7.3	56.1	−0.95	+1.48
**1d**	471	--	7.6	65.1	−0.76	+2.06
**1e**	481	--	6.2	61.8	−0.76	+1.92
**6**	412	380	7.6	50.4	−0.76	+1.42

[a]PhCl solutions (1×10^{-3} M), under air at room temperature. [b]CH_2Cl_2 solutions (1×10^{-3} M), under N_2 at room temperature. Excited at 370 nm for **1a-1e** and at 360 nm for **5**. [c]CH_2Cl_2 solutions (1×10^{-3} M), under N_2 at room temperature. Excited at 360 nm. (Decay time curves were performed on an EDINBURGH FLS 980 fluorescence spectrophotometer equipped with a monochromated 325 W Xe-arc excitation source and a visible detector (Hamamatsu R928P)) [d]Ferrocene, Cp_2Fe, was used as a reference. [e]$E_{1/2(red)}{*} = E_{1/2(red)} + \Delta G^{T1}/23.06$.

Supplementary Table 5. Electrochemical data of **1a-1e** in the presence of 20 mmol% $La(OTf)_3$.[a]

Comp.	$E_{1/2(red)}$ (V)[d,f]	$E_{1/2(red)}$* (V)[a]
**1a**	−0.91	+1.63
**1b**	−0.76	+1.55
**1c**	−0.96	+1.47
**1d**	−0.76	+2.06
**1e**	−0.68	+2.00

[a]$E_{1/2(red)}{*} = E_{1/2(red)} + \Delta G^{T1}/23.06$.[2]

DFT calculations

Supplementary Table 6. The calculated triplet-state energy of **1a-e** and **6**.[a]

Comp.	$E_{T\text{-}S}$ (kcal/mol)
**1a**	58.5
**1b**	53.2
**1c**	56.1
**1d**	65.1
**1e**	61.8
**6**	50.4

[a]Theory: M11/Def2-TZVP//B3LYP/6-31+G(d,p)

Computational methods for the triplet-state energy of 1a-e and 6

Density functional theory studies were carried out with Gaussian 16[1] computational chemistry suite. Geometries of gas phase minimum and transition state electronic structures were optimised using B3LYP[2-3] functional with Pople's basis set[4] 6-31+G(d,p). Frequency calculations were carried out at that level to ensure convergence (all positive eigenvalues for minima and single negative for saddle points). Thermochemical corrections and zero point vibrational energies, as well as the infrared, were determined at the gas phase B3LYP/6-31+G(d,p) level using the unscaled frequencies. Single point calculations with larger basis set on gas phase optimized structures were carried out with Minnesota functional M11[5] functional and Wiegend and Aldrich's triple zeta def2-TZVP[6-7] basis set. The M11/def2-TZVP energies together with zero point energies constitute the relative E, reported here and in the manuscript in kcal/mol. Triplet-singlet energy difference is denoted $E_{T\text{-}S}$.

Supplementary Table 7. The calculated BDEs of the O−H bond in **IV** generated from **1a-e**.

Comp.	ΔH (kcal/mol)
**IV** from **1a**	48.6
**IV** from **1b**	48.4
**IV** from **1c**	48.1
**IV** from **1d**	49.8
**IV** from **1e**	49.4

<u>Computational methods for the O−H bond in IV generated from 1a-e</u>

All DFT calculations were performed with Gaussian 09.[8] Geometry optimizations were performed in the gas phase with the M06-2X functional and the basis set of 6-31G(d).

UV-vis absorption spectroscopy:

UV-vis absorption spectroscopy was performed using a spectrophotometer, equipped with a temperature control unit at 25 °C. The samples were measured in a 1.5 mL quartz cuvettes fitted with a PTFE stopper, **1a-1e** and **6** were prepared as a 10.0 mM solution and used fresh for measurement (Supplementary Figs 105).

UV-vis absorption spectra of EDA experiment:

UV-vis absorption spectroscopy was performed using a spectrophotometer, equipped with a temperature control unit at 25 °C. The samples were measured in a 1.5 mL quartz cuvettes fitted with a PTFE stopper. Stock solutions of **1e**, toluene and $La(OTf)_3$ were prepared with the same concentration used in the reaction. The solutions were prepared in the presence of air using PhCl as solvent. The results show that toluene and $La(OTf)_3$ have no absorption (370−500 nm), and **1e** was the only absorbing species. When we mixed **1e**, toluene and $La(OTf)_3$ together, no another strong absorption appears (370−500 nm) was observed, indicating that the electron-donor acceptor (EDA) complex was not formed in the reaction (Supplementary Figs 106).

Fluorescence spectra:

Emission intensities were recorded on a Perkin-Elmer LS55 spectrofluorometer. The solution of **1a-1e** (1.0×10^{-3} M in DCM under N_2) was excited at 370 nm and **6** (1.0×10^{-3} M in DCM under N_2) was excited at 360 nm. The emission spectrum of the sample was then collected. It was found that nearly no fluorescence was detected for **1a**, **1d** and **1e** (Supplementary Figs 107).

Intermolecular Competition KIE Experiment

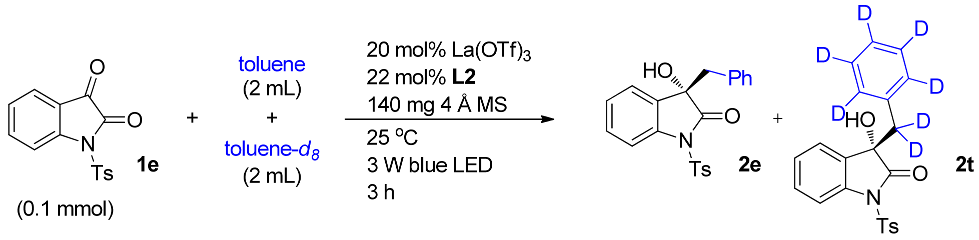

The reaction was conducted with **1e** as limiting reagent. A total of **1e** (0.1 mmol, 1.0 equiv), **L2** (0.022 mmol, 0.22 equiv), La(OTf)$_3$ (0.02 mmol, 0.2 equiv) and 4Å MS (140 mg) were added into a 25 mL Schlenk tube. Subsequently, toluene (2 mL) and toluene-d_8 (2.0 mL) were sequentially added, degassed three times by freeze-pump-thaw method. The reaction mixture was stirred under an argon atmosphere at 25 °C (the temperature was maintained in an incubator) in dark for 2 hours, then irradiated by a 3 W blue LED (λ = 450−455 nm) from a 3.0 cm distance for another 3 h at 25 °C. The reaction mixture was directly loaded onto a short silica gel column, followed by gradient elution with petroleum ether/ethyl acetate (20/1–5/1 ratio). Removing the solvent in vacuo afforded mixture of **2e** and **2t** (Supplementary Fig 108).

First run: (20% isolated yield) KIE = 3.85; Second run: (22% isolated yield) KIE = 3.78

	Ratio of the product (%)		
run	toluene	toluene-d_8	K_H/K_D
1	77.92	22.08	3.85
2	79.37	20.63	3.78
			Average 3.81
	KIE = k_H/k_D = 3.8 ± 0.05		

Independent-Rate KIE Experiments

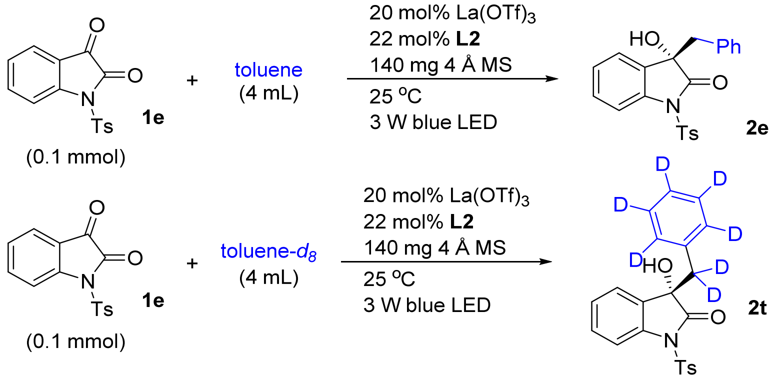

The reaction was conducted with **1e** as limiting reagent. A total of **1e** (0.1 mmol, 1.0 equiv), **L2** (0.022 mmol, 0.22 equiv), La(OTf)$_3$ (0.02 mmol, 0.2 equiv) and 4Å MS (140 mg) were added into a 25 mL Schlenk tube. Subsequently, toluene (4 mL) or toluene-d_8 (4.0 mL) were sequentially added, degassed three times by freeze-pump-thaw method. The reaction mixture was stirred under an argon atmosphere at 25 ^{o}C (the temperature was maintained in an incubator) in dark for 2 h, then irradiated by a 3 W blue LED (λ = 450−455 nm) from a 3.0 cm distance for another hours at 25 ^{o}C. The reaction mixture was directly loaded onto a short silica gel column, followed by gradient elution with petroleum ether/ethyl acetate (20/1–5/1 ratio). Removing the solvent in vacuo afforded mixture of **2e** or **2t** (Supplementary Fig 109).

Study of byproduct

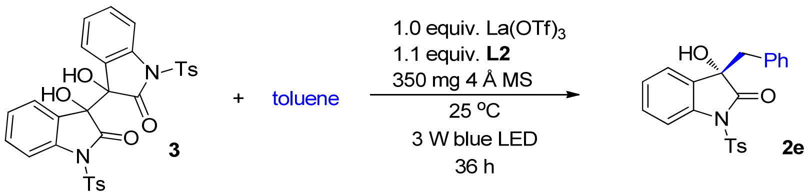

3 (0.05 mmol, 1.0 equiv), **L2** (0.055 mmol, 1.1 equiv), La(OTf)$_3$ (0.05 mmol, 1.0 equiv) and 4Å MS (350 mg) were added into a 50 mL Schlenk tube. Subsequently, toluene (16 mL) was added, degassed three times by freeze-pump-thaw method. The reaction mixture was stirred under an argon atmosphere at 25 ^{o}C (the temperature was maintained in an incubator) in dark for 2 h, then irradiated by a 3 W blue LED (λ = 450−455 nm) from a 3.0 cm distance for another 36 h at 25 ^{o}C. The reaction mixture was directly loaded onto a short silica gel column, followed by gradient elution with petroleum ether/ethyl acetate (20/1–5/1 ratio). Removing the solvent in vacuo, afforded **2e** in 73% yield with 90% ee. (Note: (1) Since the poor soluability even in DMSO-d_6 and instabilityin DMSO-d_6, NMR spectra of **3** did not be provided. To determine the structure, X-ray analysis was performed and its structure was confirmed, see: Supplementary Figure 111 in Supplementary Note 2; (2) 3-Hydroxy-2-oxindole should be a reasonable side product through proportionation of dioxindolyl radical

IV derived from **3**, however, other some unknown side products but not this one were detected in the reaction mixture, probably due to it further experiencing unknown transformations**)**

Supplementary Note 3

Determination of the absolute configurations

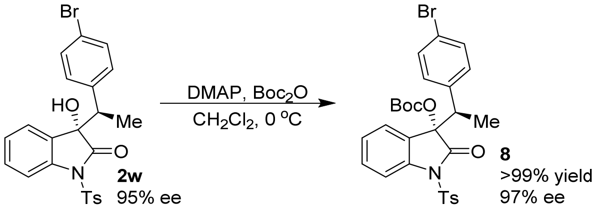

2w (0.02 mmol, 1.0 equiv) was dissolved in 1.0 mL DCM and DMAP (0.00 mmol, 0.1 equiv) were added at 25 °C. The mixture was stirred for 5 min and subsequently Boc_2O (0.022 mmol, 1.1 equiv) was added dropwise. The reaction was monitored by TLC. After completion of the reaction (0.5 h), the reaction mixture was directly loaded onto a short silica gel column, followed by gradient elution with petroleum ether/ethyl acetate (100/1–10/1 ratio). Removing the solvent in vacuo, afforded product **8**.

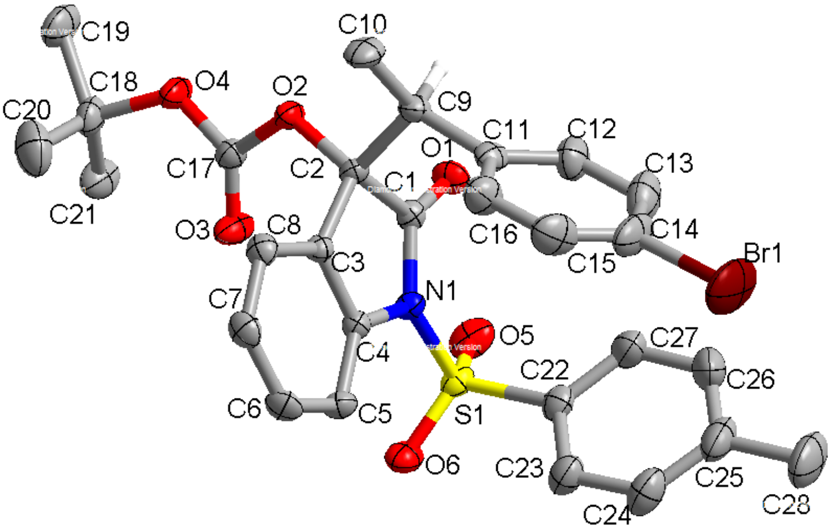

Supplementary Figure 110. Absolute configuration of **8** (CCDC 1877066)
Displacement ellipsoids are drawn at the 30% probability level.
(Solvent: ethyl acetate:*n*hexane = 1:1)

Supplementary Table 8. Crystal data and structure refinement for LFY18087P2.

Identification code	LFY18087P2
Empirical formula	$C_{28}H_{28}BrNO_6S$
Formula weight	586.48
Temperature/K	293(2)
Crystal system	orthorhombic

Space group	$P2_12_12_1$
a/Å	9.08395(19)
b/Å	15.4918(4)
c/Å	19.4437(4)
α/°	90
β/°	90
γ/°	90
Volume/Å^3	2736.23(10)
Z	4
ρ_{calc}g/cm^3	1.424
μ/mm^{-1}	3.114
F(000)	1208.0
Crystal size/mm^3	0.16 × 0.15 × 0.13
Radiation	CuKα (λ = 1.54184)
2Θ range for data collection/°	7.296 to 141.788
Index ranges	$-6 \leq h \leq 10$, $-18 \leq k \leq 18$, $-23 \leq l \leq 19$
Reflections collected	10329
Independent reflections	5157 [R_{int} = 0.0301, R_{sigma} = 0.0410]
Data/restraints/parameters	5157/2/352
Goodness-of-fit on F^2	1.030
Final R indexes [I>=2σ (I)]	R_1 = 0.0481, wR_2 = 0.1247
Final R indexes [all data]	R_1 = 0.0550, wR_2 = 0.1331
Largest diff. peak/hole / e Å^{-3}	0.47/-0.57
Flack parameter	-0.028(13)

Experimental

The crystal was kept at 293(2) K during data collection. Using Olex2, the structure was solved with the ShelXS structure solution program using Direct Methods and refined with the ShelXL refinement package using Least Squares minimisation.

Crystal structure determination

Crystal Data for $C_{28}H_{28}BrNO_6S$ (M =586.48 g/mol): orthorhombic, space group $P2_12_12_1$ (no. 19), a = 9.08395(19) Å, b = 15.4918(4) Å, c = 19.4437(4) Å, V = 2736.23(10) Å^3, Z = 4, T = 293(2) K, μ(CuKα) = 3.114 mm^{-1}, $Dcalc$ = 1.424 g/cm^3, 10329 reflections measured (7.296° ≤ 2Θ ≤ 141.788°), 5157 unique (R_{int} = 0.0301, R_{sigma} = 0.0410) which were used in all calculations. The final R_1 was 0.0481 (I > 2σ(I)) and wR_2 was 0.1331 (all data).

2) *X*-Ray structure analysis of **3**

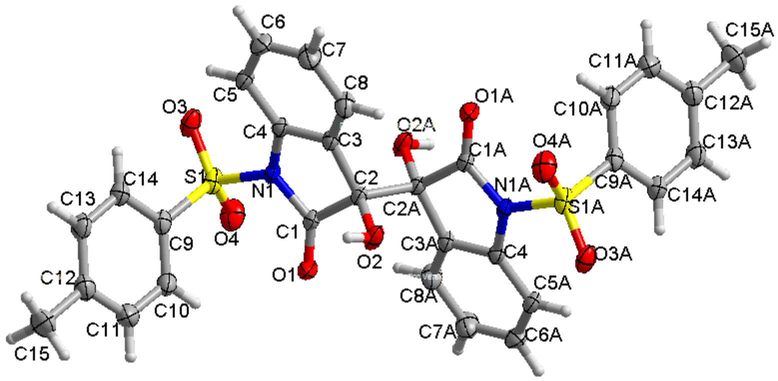

Supplementary Figure 111. Absolute configuration of **3** (CCDC 1896394)
Displacement ellipsoids are drawn at the 30% probability level.

(Solvent: EA)

Supplementary Table 9. Crystal data and structure refinement.

Identification code	LFY
Empirical formula	$C_{38}H_{48}N_2O_{12}S_6$
Formula weight	917.14
Temperature/K	293(2)
Crystal system	triclinic
Space group	P-1
a/Å	9.2978(7)
b/Å	10.5503(7)
c/Å	12.1758(9)
α/°	100.989(6)
β/°	95.793(6)
γ/°	104.675(6)
Volume/Å^3	1120.14(14)
Z	1
ρ_{calc}g/cm^3	1.360
μ/mm^{-1}	3.327
F(000)	482.0
Crystal size/mm^3	0.16 × 0.14 × 0.12
Radiation	CuKα (λ = 1.54184)
2Θ range for data collection/°	7.49 to 134.142
Index ranges	-11 ≤ h ≤ 11, -9 ≤ k ≤ 12, -14 ≤ l ≤ 14
Reflections collected	7892
Independent reflections	3998 [R_{int} = 0.0321, R_{sigma} = 0.0419]
Data/restraints/parameters	3998/28/283
Goodness-of-fit on F^2	1.044
Final R indexes [I>=2σ (I)]	R_1 = 0.0522, wR_2 = 0.1437
Final R indexes [all data]	R_1 = 0.0633, wR_2 = 0.1570
Largest diff. peak/hole / e Å^{-3}	0.42/-0.54

Experimental

The crystal was kept at 293(2) K during data collection. Using Olex2, the structure was solved with the ShelXS structure solution program using Direct Methods and refined with the ShelXL refinement package using Least Squares minimisation.

Crystal structure determination

Crystal Data for $C_{38}H_{48}N_2O_{12}S_6$ (M =917.14 g/mol): triclinic, space group P-1 (no. 2), a = 9.2978(7) Å, b = 10.5503(7) Å, c = 12.1758(9) Å, α = 100.989(6)°, β = 95.793(6)°, γ = 104.675(6)°, V = 1120.14(14) Å^3, Z = 1, T = 293(2) K, μ(CuKα) = 3.327 mm^{-1}, $Dcalc$ = 1.360 g/cm^3, 7892 reflections measured (7.49° ≤ 2Θ ≤ 134.142°), 3998 unique (R_{int} = 0.0321, R_{sigma} = 0.0419) which were used in all calculations. The final R_1 was 0.0522 (I > 2σ(I)) and wR_2 was 0.1570 (all data).

3) Absolute configurations of **6** are determined by X-ray structure analysis of the product **7b**

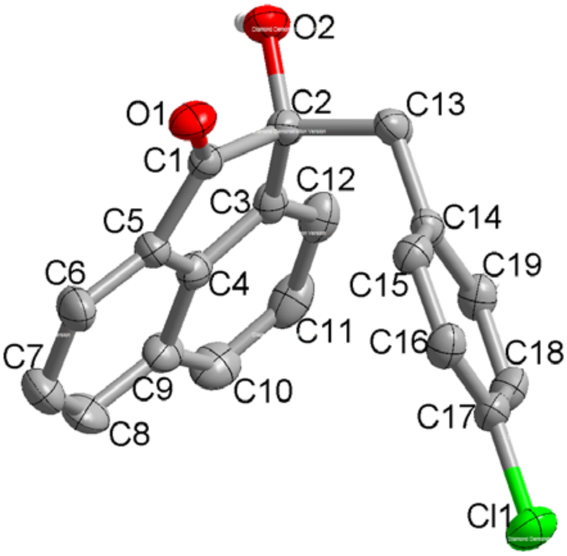

Supplementary Figure 112. Absolute configuration of **7b** (CCDC 1877077)

Displacement ellipsoids are drawn at the 30% probability level.

(Solvent: DCM/PE = 1:5)

Supplementary Table 10. Crystal data and structure refinement.

Identification code	1235644CL
Empirical formula	$C_{19}H_{13}ClO_2$
Formula weight	308.74
Temperature/K	293(2)
Crystal system	monoclinic
Space group	$P2_1$
a/Å	9.8402(6)

b/Å	12.9406(8)
c/Å	12.8070(8)
α/°	90
β/°	109.637(7)
γ/°	90
Volume/$Å^3$	1535.98(17)
Z	4
ρ_{calc}g/cm^3	1.335
μ/mm^{-1}	2.230
F(000)	640.0
Crystal size/mm^3	0.25 × 0.15 × 0.13
Radiation	CuKα (λ = 1.54184)
2Θ range for data collection/°	7.328 to 134.096
Index ranges	$-11 \leq h \leq 11$, $-15 \leq k \leq 15$, $-15 \leq l \leq 13$
Reflections collected	11323
Independent reflections	5456 [R_{int} = 0.0306, R_{sigma} = 0.0358]
Data/restraints/parameters	5456/45/385
Goodness-of-fit on F^2	1.049
Final R indexes [I>=2σ (I)]	R_1 = 0.0640, wR_2 = 0.1808
Final R indexes [all data]	R_1 = 0.0710, wR_2 = 0.1938
Largest diff. peak/hole / e $Å^{-3}$	0.48/-0.34
Flack parameter	0.027(12)

Experimental

The crystal was kept at 293(2) K during data collection. Using Olex2, the structure was solved with the Superflip structure solution program using Charge Flipping and refined with the ShelXL refinement package using Least Squares minimisation.

Crystal structure determination

Crystal Data for $C_{19}H_{13}ClO_2$ (M =308.74 g/mol): monoclinic, space group $P2_1$ (no. 4), a = 9.8402(6) Å, b = 12.9406(8) Å, c = 12.8070(8) Å, β = 109.637(7)°, V = 1535.98(17) $Å^3$, Z = 4, T = 293(2) K, μ(CuKα) = 2.230 mm^{-1}, $Dcalc$ = 1.335 g/cm^3, 11323 reflections measured (7.328° ≤ 2Θ ≤ 134.096°), 5456 unique (R_{int} = 0.0306, R_{sigma} = 0.0358) which were used in all calculations. The final $R_1$was 0.0640 (I > 2σ(I)) and wR_2 was 0.1938 (all data).

Supplementary Data

dio-H

1\1\GINC-STD1634\FOpt\RB3LYP\6-31+G(d,p)\C8H5N1O2\RZL501\08-Oct-2018\0
\\# b3lyp/6-31+g(d,p) opt=maxcyc=200 scf=maxcyc=200 freq\\Isatin N-H\\
0,1\C,-1.382414,1.399644,-0.000324\C,-0.254939,0.580659,-0.000128\C,-0
.381453,-0.82366,0.000176\C,-1.630757,-1.432378,0.000182\C,-2.757814,-
0.597235,0.000066\C,-2.645491,0.800146,-0.000182\H,-1.266341,2.479159,
-0.000476\H,-1.737607,-2.512676,0.000272\H,-3.744567,-1.051133,0.00010
5\H,-3.54067,1.41318,-0.000261\N,0.886399,-1.427448,0.000195\H,1.05623
3,-2.42346,-0.000051\C,1.181187,0.902477,0.000047\C,1.909004,-0.49363,
-0.00009\O,1.75066,1.972193,0.000501\O,3.099867,-0.713328,-0.000429\\V
ersion=ES64L-G16RevA.03\State=1-A\HF=-513.0949382\RMSD=7.448e-09\RMSF=
2.719e-05\Dipole=-2.2542116,-1.182066,-0.0001995\Quadrupole=-5.4991965
,5.1733666,0.3258299,-2.6790714,0.0004344,-0.0019284\PG=C01 [X(C8H5N1O
2)]\\@

dio-H (triplet)

1\1\GINC-STD1636\FOpt\UB3LYP\6-31+G(d,p)\C8H5N1O2(3)\RZL501\08-Oct-201
8\0\\# ub3lyp/6-31+g(d,p) opt=maxcyc=200 scf=maxcyc=200 freq\\Isatin N
-H\\0,3\C,-1.337625,1.419897,-0.055765\C,-0.216898,0.578571,0.003543\C
,-0.395455,-0.846984,-0.002078\C,-1.668356,-1.430081,0.035424\C,-2.765
468,-0.576185,0.019484\C,-2.598796,0.834277,-0.020871\H,-1.213131,2.49
6984,-0.072136\H,-1.790711,-2.508758,0.033707\H,-3.767734,-0.991778,0.
031531\H,-3.480593,1.467466,0.000568\N,0.837021,-1.431619,-0.051066\H,
1.028258,-2.407258,0.142765\C,1.202252,0.858084,0.056197\C,1.887594,-0
.469862,-0.015265\O,1.763724,1.985064,0.03622\O,3.076436,-0.765267,-0.
024093\\Version=ES64L-G16RevA.03\State=3-A\HF=-513.0162578\S2=2.011695
\S2-1=0.\S2A=2.000082\RMSD=3.649e-09\RMSF=7.748e-05\Dipole=-2.386907,-
1.2728623,0.0918556\Quadrupole=-5.1325207,5.4612192,-0.3286984,-2.8977
839,0.2849259,-0.8274343\PG=C01 [X(C8H5N1O2)]\\@

dio-Me

1\1\GINC-STD1634\FOpt\RB3LYP\6-31+G(d,p)\C9H7N1O2\RZL501\08-Oct-2018\0
\\# b3lyp/6-31+g(d,p) opt=maxcyc=200 scf=maxcyc=200 freq\\Isatin N-Me\
\0,1\C,-1.3804911202,1.4006972586,-0.0000365123\C,-0.2542114266,0.5831
380821,0.0000570538\C,-0.3759387876,-0.8225278995,0.0000059538\C,-1.62
63750072,-1.4316205975,-0.0002507633\C,-2.754897189,-0.59623459,-0.000
3405011\C,-2.6445139445,0.8000494806,-0.0002198411\H,-1.2649968069,2.4

803224063,0.0000051772\H,-1.7365631723,-2.5106095681,-0.0004099973\H,-
3.7411584956,-1.0513824875,-0.0005258867\H,-3.5400544945,1.4125671927,
-0.0002967173\N,0.8888150205,-1.4376712329,0.000239885\C,1.181091008,0
.9023867813,0.0001245627\C,1.9022347603,-0.4915790582,0.000198342\O,1.
7525826693,1.9714133139,0.0002794896\O,3.0966049593,-0.7073823727,0.00
01633894\C,1.1206275652,-2.8698865004,-0.0000436877\H,0.6859865737,-3.
3351038472,0.8915499922\H,0.690004311,-3.3343216856,-0.8940252499\H,2.
2001246768,-3.027191456,0.0022671909\\Version=ES64L-G16RevA.03\State=1
-A\HF=-552.4100749\RMSD=6.049e-09\RMSF=2.797e-05\Dipole=-2.1453534,-1.
3573678,-0.0002569\Quadrupole=-4.8156498,4.4233528,0.392297,-4.482618,
0.0002705,-0.0005521\PG=C01 [X(C9H7N1O2)]\\@

dio-Me (triplet)

1\1\GINC-STD1641\FOpt\UB3LYP\6-31+G(d,p)\C9H7N1O2(3)\RZL501\08-Oct-201
8\0\\# ub3lyp/6-31+g(d,p) opt=maxcyc=200 scf=maxcyc=200 freq\\Isatin N
-Me\\0,3\C,-1.3270625153,1.4206808014,-0.0581968436\C,-0.2017620397,0.
5857917161,-0.0239671328\C,-0.3979379019,-0.8536124602,0.0744811176\C,
-1.6828815947,-1.4352752057,0.1370499163\C,-2.7694560819,-0.582236743,
0.1012004409\C,-2.5860991683,0.8373908094,0.0041593875\H,-1.1983851177
,2.4951838502,-0.1314977416\H,-1.810124716,-2.5102395631,0.2099996374\
H,-3.776490934,-0.9824464068,0.1464042421\H,-3.4687742558,1.4701396469
,-0.02064154\N,0.8141506819,-1.4381602781,0.0900484273\C,1.2154187402,
0.8728419769,-0.0691564226\C,1.8940656827,-0.4289749271,0.0036820507\O
,1.7603689157,2.006101083,-0.1540233883\O,3.0682126852,-0.7800513346,0
.0047303643\C,1.1030667311,-2.8565920679,0.1801908066\H,2.1887653926,-
2.9630567316,0.1670517337\H,0.700249861,-3.2692185965,1.1106164083\H,0
.667159555,-3.3891126494,-0.671057234\\Version=ES64L-G16RevA.03\State=
3-A\HF=-552.3350955\S2=2.015097\S2-1=0.\S2A=2.000124\RMSD=9.247e-09\RM
SF=2.974e-05\Dipole=-2.8435996,-2.0322461,0.1878979\Quadrupole=-4.3902
742,3.9285479,0.4617263,-6.8392832,0.5442954,-0.1045406\PG=C01 [X(C9H7
N1O2)]\\@

dio-Ac

1\1\GINC-STD1652\FOpt\RB3LYP\6-31+G(d,p)\C10H7N1O3\RZL501\08-Oct-2018\
0\\# b3lyp/6-31+g(d,p) opt=maxcyc=200 scf=maxcyc=200 freq\\Isatin N-Ac
etyl\\0,1\C,-1.3660018374,1.3823222561,0.0328707012\C,-0.2513819384,0.
5443169684,-0.0040422364\C,-0.3825852306,-0.8586454844,-0.0275531166\C
,-1.6449201541,-1.448089751,-0.0143037735\C,-2.7573794686,-0.593881384

3,0.02288442\C,-2.6337461129,0.8016047696,0.0464109559\H,-1.2278813425
,2.45887993,0.0501760499\H,-1.7624547766,-2.5208399666,-0.0318930681\H
,-3.7478071535,-1.0395641683,0.0335930184\H,-3.5217783755,1.4245443047
,0.0749978918\N,0.9109930254,-1.4844765613,-0.0641767526\C,1.168626484
1,0.8845693304,-0.0252429589\C,1.9157094697,-0.483575437,-0.0651256412
\O,1.7327292421,1.9580244125,-0.0162515329\O,3.111861413,-0.6427841478
,-0.0912944861\C,1.1201736335,-2.888520955,-0.093469852\C,2.5419906737
,-3.3894278186,-0.1303368746\H,2.4943693838,-4.4782836485,-0.148820068
7\H,3.0733277893,-3.0167937036,-1.009841311\H,3.1079701899,-3.04789072
83,0.7400952021\O,0.1613058457,-3.640089787,-0.0882304868\\Version=ES6
4L-G16RevA.03\State=1-A\HF=-665.7533709\RMSD=5.834e-09\RMSF=8.767e-06\
Dipole=-1.3021498,-0.3117332,0.018907\Quadrupole=3.0706182,-4.5530513,
1.4824331,-10.1700099,-0.2153872,0.0887384\PG=C01 [X(C10H7N1O3)]\\@

dio-Ac (triplet)

1\1\GINC-STD1616\FOpt\UB3LYP\6-31+G(d,p)\C10H7N1O3(3)\RZL501\09-Oct-20
18\0\\# ub3lyp/6-31+g(d,p) opt=maxcyc=200 scf=maxcyc=200 freq\\Isatin
N-Acetyl\\0,3\C,-1.3350436342,1.4039940102,-0.0411971391\C,-0.23115388
12,0.5404630289,-0.0092218697\C,-0.3918718152,-0.8626228748,0.05676923
04\C,-1.6656040621,-1.4239550173,0.0918981452\C,-2.7651755955,-0.55495
56604,0.0596680073\C,-2.6073831212,0.8378652335,-0.005842963\H,-1.1926
685453,2.4776817351,-0.0918459286\H,-1.7928426368,-2.4953803472,0.1423
080801\H,-3.7639857831,-0.9793504924,0.0864719802\H,-3.4817075825,1.48
07357697,-0.0292320935\N,0.8842959208,-1.4743905326,0.0758166308\C,1.1
817707628,0.845155048,-0.0334852159\C,1.9021991942,-0.4775577173,0.022
3501795\O,1.7343371788,1.9728733727,-0.0896212198\O,3.1090142186,-0.66
47775967,0.0221740026\C,1.1136166917,-2.8745042992,0.1390869397\C,2.53
65727835,-3.3743687886,0.1520242718\H,3.07881436,-3.0627982101,-0.7442
315461\H,3.0917310842,-2.9809390499,1.007220622\H,2.486617108,-4.46197
20825,0.2031889434\O,0.1549097844,-3.624444959,0.180686073\\Version=ES
64L-G16RevA.03\State=3-A\HF=-665.6738043\S2=2.018153\S2-1=0.\S2A=2.000
202\RMSD=8.086e-09\RMSF=4.410e-06\Dipole=-0.792116,-0.0528,0.0082238\Q
uadrupole=2.2587606,-2.1764987,-0.0822619,-8.3658961,0.3709833,0.15788
13\PG=C01 [X(C10H7N1O3)]\\@

dio-Ts

1\1\GINC-STD1629\FOpt\RB3LYP\6-31+G(d,p)\C14H9N1O4S1\RZL501\08-Oct-201
8\0\\# b3lyp/6-31+g(d,p) opt=maxcyc=200 scf=maxcyc=200 freq\\Isatin N-

Ts\\0,1\C,3.877199631,-0.3130910918,-0.929147221\C,2.611107225,-0.5996
065001,-0.4189777012\C,1.763258211,0.4216998976,0.060687622\C,2.178862
7625,1.7498109538,0.0400251898\C,3.4530447431,2.022348693,-0.480263706
7\C,4.2997183433,1.0158240382,-0.9618951351\H,4.5053067889,-1.12303887
88,-1.2870579283\H,1.5505838519,2.5438551129,0.4162713521\H,3.78906399
77,3.0549998772,-0.501228732\H,5.2791277523,1.2708771341,-1.3528974195
\N,0.5353906904,-0.1503181096,0.5329518274\C,1.93792821,-1.888512731,-
0.2615140295\C,0.5505744067,-1.5503269384,0.3741072873\O,2.2966024135,
-3.0127696048,-0.5385389231\O,-0.3365238368,-2.3224868902,0.6456418147
\S,-0.8411042495,0.6828155644,1.2251601116\C,-2.1560088112,0.362671828
5,0.0534487975\C,-2.3359003176,1.2527622499,-1.010189794\C,-2.98356038
64,-0.7463098615,0.244744028\C,-3.3731945544,1.0136747381,-1.911865876
2\H,-1.6903228778,2.1177561195,-1.1143886724\C,-4.0178896029,-0.967821
0221,-0.6664252555\H,-2.8122768872,-1.4146759499,1.0795406459\C,-4.210
0321908,-0.0941696267,-1.7404440768\H,-3.5315679123,1.6959157636,-2.74
11809964\H,-4.6711617392,-1.8245114852,-0.5348757378\H,-5.016969757,-0
.2739236991,-2.444559126\O,-0.4886879389,2.1017726097,1.1639173641\O,-
1.1380639655,0.0208968085,2.4872842911\\Version=ES64L-G16RevA.03\State
=1-A\HF=-1292.7193472\RMSD=8.143e-09\RMSF=5.075e-06\Dipole=-0.104827,1
.423431,-1.6782119\Quadrupole=16.4299336,-10.8790043,-5.5509293,8.3869
579,4.9455164,-3.9612137\PG=C01 [X(C14H9N1O4S1)]\\@

dio-Ts (triplet)

1\1\GINC-STD1624\FOpt\UB3LYP\6-31+G(d,p)\C14H9N1O4S1(3)\RZL501\08-Oct-
2018\0\\# ub3lyp/6-31+g(d,p) opt=maxcyc=200 scf=maxcyc=200 freq\\Isati
n N-Ts\\0,3\C,4.0077470771,-0.431311791,-0.7015610046\C,2.679426103,-0
.654262217,-0.313872627\C,1.7930497356,0.4416314097,-0.0728231083\C,2.
2570423128,1.7604453991,-0.1393618625\C,3.591779856,1.9618679295,-0.48
78432393\C,4.4602655381,0.8821292354,-0.7630714815\H,4.6656169427,-1.2
702228748,-0.8992169926\H,1.592230341,2.5915656983,0.0474498174\H,3.96
7882609,2.9777092232,-0.5550100188\H,5.4994179579,1.0813879208,-1.0059
696402\N,0.5194677046,-0.0728328991,0.1843191236\C,1.9611814611,-1.891
7296126,-0.1124158887\C,0.5429295318,-1.5139318929,0.1645304234\O,2.38
63083295,-3.0642047691,-0.2731301534\O,-0.4321665117,-2.2265842763,0.3
475721056\S,-0.774097571,0.768900552,1.0498032848\C,-2.2206453092,0.35
91343659,0.0837121284\C,-2.5122473198,1.1358596937,-1.0419511726\C,-3.
043723172,-0.6890858521,0.5010477514\C,-3.661212487,0.8404205329,-1.77
49781423\H,-1.8635072434,1.9575396148,-1.3243270515\C,-4.1927641458,-0

.9655791476,-0.2409838409\H,-2.783181615,-1.27012477,1.3769102717\C,-4
.4976935654,-0.2074319267,-1.3748713114\H,-3.9064119823,1.432310647,-2
.6511353736\H,-4.845155847,-1.7765048076,0.0665106076\H,-5.3923035784,
-0.4309869374,-1.9483906271\O,-0.4654066302,2.186687728,0.861838517\O,
-0.8761015221,0.1756308238,2.3770065051\\Version=ES64L-G16RevA.03\Stat
e=3-A\HF=-1292.6404664\S2=2.012977\S2-1=0.\S2A=2.000103\RMSD=4.921e-09
\RMSF=2.409e-05\Dipole=-0.3762083,1.2424645,-1.4531717\Quadrupole=18.3
85937,-11.8991322,-6.4868048,7.808692,3.0663665,-4.049815\PG=C01 [X(C1
4H9N1O4S1)]\\@

dio-Bn

1\1\GINC-STD1636\FOpt\RB3LYP\6-31+G(d,p)\C15H11N1O2\RZL501\08-Oct-2018
\0\\# b3lyp/6-31+g(d,p) opt=maxcyc=200 scf=maxcyc=200 freq\\Isatin N-B
n\\0,1\C,-3.221065978,-1.2576685506,-0.6083988148\C,-2.3655782005,-0.2
289015847,-0.222892925\C,-1.106998417,-0.504514364,0.3518090201\C,-0.6
836655802,-1.8148539932,0.5468251498\C,-1.5559027534,-2.8430843561,0.1
566862546\C,-2.8080619403,-2.5796702181,-0.4135956575\H,-4.1858144242,
-1.0224111664,-1.0475125894\H,0.2873291568,-2.043644361,0.9701753303\H
,-1.243339788,-3.8735071062,0.299541714\H,-3.4534787138,-3.4021292916,
-0.7038584514\N,-0.4258260977,0.6896857985,0.6659795425\C,-2.514361432
7,1.2310948174,-0.2898128766\C,-1.1882118755,1.7974682448,0.3273019479
\O,-3.4228793749,1.9154433323,-0.7098506444\O,-0.8833260296,2.96233210
35,0.4833096451\C,0.8681533356,0.780782651,1.3330870151\H,0.8321817724
,0.1977602203,2.2610908418\H,0.9767328042,1.8349371247,1.6079648281\C,
2.0450875951,0.3319441219,0.482297797\C,3.0165229748,-0.5211381841,1.0
193746145\C,2.2018338406,0.7967780159,-0.8309324988\C,4.1266251888,-0.
9060540241,0.2611059425\H,2.9083791641,-0.8864607491,2.0384024896\C,3.
3060597167,0.4096490671,-1.5918558583\H,1.4599548368,1.4660433945,-1.2
578341723\C,4.2723917139,-0.4429601222,-1.0480518974\H,4.8714393282,-1
.5686893527,0.6923928989\H,3.4147940736,0.7773584031,-2.6080503605\H,5
.1314761039,-0.7426328712,-1.6409782854\\Version=ES64L-G16RevA.03\Stat
e=1-A\HF=-783.4749763\RMSD=9.899e-09\RMSF=8.147e-06\Dipole=1.126301,-2
.270084,0.3400312\Quadrupole=1.3194162,-5.8264301,4.5070139,7.7344008,
-0.81817,-0.8454206\PG=C01 [X(C15H11N1O2)]\\@

dio-Bn (triplet)

1\1\GINC-STD0117\FOpt\UB3LYP\6-31+G(d,p)\C15H11N1O2(3)\RZL501\10-Oct-2
018\0\\# ub3lyp/6-31+g(d,p) opt=maxcyc=200 scf=maxcyc=200 freq\\Isatin

N-Bn\\0,3\C,-3.3246745803,-0.8458053523,-0.7722493157\C,-2.3436965136
,0.0193588171,-0.2716644794\C,-1.1771907788,-0.5443571139,0.391680158\
C,-0.9973373998,-1.9384296593,0.5324460431\C,-1.9827411623,-2.76243623
55,0.0244156792\C,-3.1378562455,-2.2147122726,-0.624315479\H,-4.200423
8989,-0.4386120967,-1.2660190757\H,-0.1171491972,-2.3452307703,1.01683
00971\H,-1.8867787063,-3.8393710056,0.1106329751\H,-3.8852942446,-2.90
0584474,-1.0129230602\N,-0.3994618454,0.4763146541,0.8019962305\C,-2.2
421566234,1.4639364924,-0.2725139007\C,-0.9879312839,1.7778790667,0.42
30326632\O,-3.0661150677,2.2846135875,-0.7571908302\O,-0.4085136056,2.
8225706971,0.7074000037\C,0.8870679398,0.4008049141,1.4951183555\H,0.8
112881304,-0.3565603561,2.2804970534\H,1.0163010275,1.3815249976,1.960
9947393\C,2.0483084907,0.0946360848,0.5628740052\C,2.4024136307,1.0050
925092,-0.4439097357\C,2.7903414394,-1.0824929156,0.7139732503\C,3.477
5395164,0.7311332215,-1.2907735166\H,1.8403932842,1.9281001186,-0.5557
974196\C,3.8711665629,-1.353434525,-0.1305988945\H,2.5334125094,-1.787
8013563,1.5011548096\C,4.213656536,-0.4482329793,-1.1371955694\H,3.744
1617627,1.4423670611,-2.0669135599\H,4.4424966434,-2.2678111503,0.0001
360372\H,5.0514596798,-0.6572969591,-1.7957932641\\Version=ES64L-G16Re
vA.03\State=3-A\HF=-783.4005499\S2=2.014955\S2-1=0.\S2A=2.00012\RMSD=1
.269e-09\RMSF=7.304e-06\Dipole=1.2520654,-3.2114114,0.4462745\Quadrupo
le=2.6245294,-5.9358214,3.311292,11.8017042,-2.2300322,-0.6702095\PG=C
01 [X(C15H11N1O2)]\\@

dio-Napth

1\1\GINC-STD1655\FOpt\RB3LYP\6-31+G(d,p)\C12H6O2\RZL501\17-Oct-2018\0\
\# b3lyp/6-31+g(d,p) opt=maxcyc=200 scf=maxcyc=200 freq\\dione\\0,1\H,
1.0917895064,3.2154304233,-0.7930511341\C,0.7645236274,3.1264437802,0.
23832784\C,0.6510466367,4.2396982532,1.0459172413\C,0.021138468,1.7092
056311,2.103546446\C,0.2209066346,4.0915941912,2.3890565217\C,0.442387
6094,1.8574441991,0.7892565199\C,-0.1047941553,2.8405017268,2.95829500
83\C,0.1464468784,5.3103267824,3.1102611801\H,0.5289247245,0.976146340
9,0.1608124791\H,-0.7843000928,1.9263333912,4.8121925749\H,-0.21548169
86,0.71955233,2.4853368182\C,-0.2598971147,5.2998770828,4.4290423938\H
,-0.3245524118,6.2204192728,5.0010432479\C,-0.5925294634,4.0531004599,
5.0228146105\H,-0.9131816005,4.0357243105,6.0601170928\C,-0.5205898977
,2.8585072429,4.3195723222\C,0.5607201401,6.3992172357,2.1892186466\C,
0.8968284203,5.6858030243,0.8135732998\O,0.6415028825,7.5913341747,2.3
907672715\O,1.2721109064,6.253340147,-0.1891003805\\Version=ES64L-G16R

evA.03\State=1-A\HF=-611.3663594\RMSD=4.309e-09\RMSF=3.674e-05\Dipole=
-0.6190991,-2.3822927,1.0841922\Quadrupole=-0.3802986,-3.8731713,4.253
4699,-2.0196768,-0.7955993,5.2281591\PG=C01 [X(C12H6O2)]\\@

dio-Napth (triplet)

1\1\GINC-STD1659\FOpt\UB3LYP\6-31+G(d,p)\C12H6O2(3)\RZL501\17-Oct-2018
\0\\# ub3lyp/6-31+g(d,p) opt=maxcyc=200 scf=maxcyc=200 freq\\dione_tr\
\0,3\H,1.0919808931,3.2291850474,-0.7880919949\C,0.7649276473,3.140575
9174,0.242537348\C,0.6453764148,4.2423938537,1.0647899629\C,0.02084387
6,1.7015314504,2.1013925782\C,0.2162861695,4.074646622,2.3967177168\C,
0.4429261157,1.8655395837,0.7907674676\C,-0.1070622308,2.8321414811,2.
9620771166\C,0.1487118166,5.2961263173,3.0965215484\H,0.5339953278,0.9
926417715,0.1512374186\H,-0.7883287377,1.9280901629,4.8263163491\H,-0.
2136373784,0.7088075537,2.4753469178\C,-0.255278478,5.3050778902,4.416
036644\H,-0.3199498337,6.2247645477,4.9878712381\C,-0.589907488,4.0568
424409,5.0159380018\H,-0.908898414,4.053974619,6.0539526013\C,-0.52310
02639,2.8555628238,4.3265445458\C,0.5622285647,6.3838140341,2.17663283
13\C,0.890798363,5.6867335186,0.832555976\O,0.6372308528,7.5911092579,
2.4060646321\O,1.2738567833,6.2404411067,-0.1982088995\\Version=ES64L-
G16RevA.03\State=3-A\HF=-611.2960268\S2=2.01327\S2-1=0.\S2A=2.000102\R
MSD=6.760e-09\RMSF=8.895e-05\Dipole=-0.5044578,-1.9387413,0.8821636\Qu
adrupole=-1.8661773,-2.5224931,4.3886703,-1.476996,-1.4395928,4.658969
1\PG=C01 [X(C12H6O2)]\\@

1a

M06-2X enthalpy: -513.3035912 a.u.

ATOM	X	Y	Z
H	1.17887500	2.51033500	0.00005600
C	1.34283900	1.43777400	-0.00011300
C	1.74444900	-1.37769400	0.00002200
C	2.62130100	0.90274400	0.00003500
C	0.24756300	0.56127500	-0.00001500
C	0.46369400	-0.84822800	0.00018100
C	2.81733900	-0.48657500	0.00009300
H	3.48242700	1.56239300	0.00005100
H	3.82856800	-0.88073700	-0.00001700
H	1.90747700	-2.45072400	0.00008000
C	-1.15312100	0.73169100	-0.00032500

N	-0.76285200	-1.49857100	-0.00037400
C	-1.80131600	-0.57351300	-0.00021500
H	-0.90855600	-2.49655000	0.00143100
O	-1.86971300	1.85162200	0.00016500
H	-2.80644200	1.56953400	0.00059200
O	-3.01014600	-0.77775900	0.00014000

1a'

M06-2X enthalpy: -512.7318439 a.u.

ATOM	X	Y	Z
H	1.25319400	2.47537800	-0.00013800
C	1.37668800	1.39660800	-0.00009300
C	1.62861300	-1.42631500	0.00002700
C	2.63655900	0.80073300	0.00011400
C	0.25821500	0.57687700	-0.00033400
C	0.38186400	-0.82054500	-0.00022700
C	2.74882200	-0.59120100	0.00019800
H	3.53115900	1.41349000	0.00023500
H	3.73601600	-1.04364600	0.00039900
H	1.73446300	-2.50617500	0.00013400
C	-1.18163900	0.89540500	-0.00007400
N	-0.88222900	-1.42414300	-0.00015300
C	-1.90451600	-0.49300200	0.00004100
H	-1.05013000	-2.42006100	-0.00015200
O	-1.74924400	1.95522500	0.00011600
O	-3.08784800	-0.70289300	0.00022000

1b

M06-2X enthalpy: -552.5678205 a.u.

ATOM	X	Y	Z
H	1.77052400	-2.52671900	0.00001300
C	1.73028100	-1.44239900	0.00000500
C	1.60272300	1.39988500	-0.00000700
C	2.88808500	-0.67805100	0.00000400
C	0.49210100	-0.78598600	-0.00000600
C	0.44348800	0.64012200	-0.00001200
C	2.82345000	0.72287000	-0.00000200
H	3.85636500	-1.16733400	0.00001200

H	3.74414600	1.29763400	0.00000100
H	1.56237200	2.48453500	-0.00000800
C	-0.85467200	-1.20651100	0.00000000
N	-0.87640700	1.06662500	-0.00001300
C	-1.72268800	-0.03630800	-0.00002700
O	-1.36064700	-2.43640800	0.00001600
H	-2.33218100	-2.32520400	0.00000900
O	-2.95026400	-0.04472300	-0.00000600
C	-1.33390300	2.43413800	0.00002600
H	-0.98367500	2.96270100	-0.89249400
H	-0.98373600	2.96263800	0.89260700
H	-2.42487100	2.40786500	-0.00001200

1b'

M06-2X enthalpy: -551.9964014 a.u.

ATOM	X	Y	Z
H	1.90122800	-2.43220300	0.00000300
C	1.79830000	-1.35127500	0.00001400
C	1.46410800	1.46510900	-0.00000700
C	2.90938000	-0.50720800	0.00002100
C	0.53702900	-0.77930000	-0.00001000
C	0.36940200	0.61425600	-0.00000500
C	2.73307300	0.87710300	0.00001000
H	3.91103300	-0.92276400	0.00002800
H	3.60648000	1.52224000	0.00001800
H	1.34611200	2.54361800	-0.00000400
C	-0.80924500	-1.38141700	-0.00009700
N	-0.98834900	0.95859700	0.00001100
C	-1.79117500	-0.16621400	0.00008800
O	-1.15218700	-2.53410400	-0.00008400
O	-2.99522300	-0.19498200	0.00005600
C	-1.50063000	2.30822400	0.00000200
H	-1.16960700	2.84871100	-0.89270500
H	-1.16956100	2.84873400	0.89267600
H	-2.58940800	2.23850600	0.00003100

1c

M06-2X enthalpy: -783.4373585 a.u.

ATOM	X	Y	Z
H	-4.04869900	1.18975500	-1.10693700
C	-3.08818200	1.38131900	-0.63998500
C	-0.56313600	1.86229600	0.58357600
C	-2.62772200	2.67435100	-0.44287300
C	-2.28396300	0.31205900	-0.22105400
C	-1.02283600	0.56851400	0.39394400
C	-1.38236100	2.90937200	0.15874600
H	-3.23402400	3.51657600	-0.75904500
H	-1.04204200	3.93109300	0.29439500
H	0.40730100	2.05068600	1.03138100
C	-2.40835400	-1.09279900	-0.25531100
N	-0.41349000	-0.63343700	0.73249900
C	-1.22271400	-1.69344900	0.34001900
O	-3.39535800	-1.84514900	-0.73409000
H	-3.12315100	-2.77207200	-0.58340600
O	-0.99664900	-2.89520200	0.45757200
C	0.86683400	-0.79314400	1.38743800
H	0.93114700	-1.85482000	1.64829100
H	0.87020700	-0.21477200	2.31931300
C	2.03402200	-0.38496100	0.51369100
C	4.19529600	0.34136200	-1.10298600
C	2.98261700	0.52609100	0.97230200
C	2.17358200	-0.93242500	-0.76351500
C	3.24826800	-0.57099400	-1.56770300
C	4.06205100	0.88887600	0.16862100
H	2.87623300	0.95620000	1.96607800
H	1.43485400	-1.64690700	-1.12010800
H	3.35054500	-1.00275800	-2.55862900
H	4.79395300	1.60166400	0.53604100
H	5.03374500	0.62341400	-1.73218400

1c’

M06-2X enthalpy: -782.8663323 a.u.

ATOM	X	Y	Z
H	4.07386300	1.06942900	1.13474200
C	3.12613500	1.29003300	0.65289000
C	0.63613500	1.80166700	-0.60619400

C	2.69912300	2.59986400	0.43699300
C	2.30703000	0.25328000	0.23529700
C	1.07681400	0.50490800	-0.39096400
C	1.47116800	2.83987400	-0.18205500
H	3.31599600	3.43463500	0.75108600
H	1.14758200	3.86468800	-0.33793400
H	-0.32372300	2.00496200	-1.06859800
C	2.46520500	-1.20922500	0.32275700
N	0.43394800	-0.69457800	-0.73502500
C	1.16946900	-1.79085300	-0.32932900
O	3.35506800	-1.87705900	0.77897800
O	0.86301700	-2.95019000	-0.45066100
C	-0.85135200	-0.79183700	-1.39814500
H	-0.95543700	-1.84510500	-1.67825000
H	-0.82466700	-0.19619400	-2.31856400
C	-2.00312800	-0.35334500	-0.51900700
C	-4.13620300	0.42669600	1.10856200
C	-2.90706400	0.61178200	-0.95686500
C	-2.17376900	-0.92916300	0.74202100
C	-3.23472600	-0.54064100	1.55179400
C	-3.97259400	1.00148500	-0.14747000
H	-2.77884600	1.06077800	-1.93975900
H	-1.47341000	-1.68940500	1.08130300
H	-3.36177800	-0.99466300	2.52971600
H	-4.67097000	1.75483400	-0.49869700
H	-4.96438500	0.72893400	1.74188400

1d

M06-2X enthalpy: -665.860195 a.u.

ATOM	X	Y	Z
H	3.18221700	1.59618800	0.00001500
C	2.63612500	0.65875700	0.00000900
C	1.15918300	-1.77655100	-0.00000900
C	3.28777300	-0.56353100	0.00000800
C	1.23534600	0.66409700	0.00000200
C	0.50273400	-0.55407700	-0.00000700
C	2.55577400	-1.75820200	-0.00000100
H	4.37210300	-0.59856900	0.00001300

H	3.08677000	-2.70484500	-0.00000200
H	0.60503300	-2.70358900	-0.00001700
C	0.28153900	1.70390800	-0.00000100
N	-0.88294700	-0.26484100	-0.00001500
C	-1.04953100	1.14558600	-0.00000900
O	0.47758500	3.01698900	0.00000600
H	-0.41024000	3.42731100	0.00000100
O	-2.09020700	1.79380800	-0.00001100
C	-1.90552200	-1.23044600	-0.00002200
O	-1.62839200	-2.40814900	0.00000700
C	-3.32373400	-0.72174900	0.00002400
H	-3.51532000	-0.09762800	0.87549900
H	-3.97456200	-1.59525400	0.00006300
H	-3.51538000	-0.09766400	-0.87546300

1d'

M06-2X enthalpy: -665.2865623 a.u.

ATOM	X	Y	Z
H	3.20639400	1.56760000	0.00002300
C	2.64893500	0.63603600	0.00001400
C	1.11062900	-1.74905100	-0.00001300
C	3.27808400	-0.60387800	0.00001300
C	1.26135300	0.66790600	0.00000200
C	0.49228100	-0.50460100	-0.00001100
C	2.50812900	-1.76862700	0.00000000
H	4.36062700	-0.67012200	0.00002200
H	3.00751600	-2.73289600	-0.00000100
H	0.53627400	-2.66250200	-0.00002400
C	0.35588500	1.81939500	-0.00000100
N	-0.90308300	-0.18852100	-0.00002000
C	-1.07381900	1.21137900	-0.00001100
O	0.57623200	3.00160500	0.00000900
O	-2.10354100	1.82683200	-0.00001400
C	-1.93375200	-1.15515300	-0.00003500
O	-1.65594400	-2.33148100	0.00001600
C	-3.35226000	-0.64938600	0.00002800
H	-3.54882500	-0.02787400	0.87624300
H	-3.99826000	-1.52642200	0.00007500

H	-3.54890500	-0.02791100	-0.87619500

1e

M06-2X enthalpy: -1331.902228 a.u.

ATOM	X	Y	Z
H	4.45514500	1.42323700	-1.60676700
C	3.91650600	0.54633800	-1.26303300
C	2.47589100	-1.72317700	-0.32519000
C	4.37031400	-0.73534300	-1.52412000
C	2.73034800	0.69912700	-0.53205100
C	2.01414700	-0.44069400	-0.06875900
C	3.65667000	-1.84822700	-1.05925000
H	5.28673300	-0.88176400	-2.08589600
H	4.03283300	-2.84472500	-1.26793700
H	1.94384600	-2.59198600	0.03640600
C	2.01247200	1.83511900	-0.10218300
N	0.87665000	0.00631100	0.64222400
C	0.83768100	1.42174200	0.63131800
O	2.28884900	3.11846000	-0.30428200
H	1.57556100	3.62749000	0.12863800
O	-0.01820000	2.15270200	1.11150100
S	-0.36302200	-0.91640700	1.35946500
O	-0.54610700	-0.41188400	2.69982400
O	0.00187900	-2.29664200	1.10180600
C	-1.78165200	-0.50372800	0.38784900
C	-4.00993600	0.13567700	-1.13732500
C	-2.06373300	-1.25971000	-0.74714900
C	-2.58592500	0.56061700	0.78297600
C	-3.69780000	0.87177800	0.00899600
C	-3.18040200	-0.92974600	-1.50386200
H	-1.42491600	-2.09479700	-1.01630600
H	-2.32974600	1.12865100	1.66989600
H	-4.33492300	1.70186500	0.30093600
H	-3.41742600	-1.51036100	-2.39116000
C	-5.23604400	0.46047100	-1.94995400
H	-5.07203300	0.25878900	-3.01184100
H	-6.08571300	-0.15036000	-1.62533800
H	-5.52014700	1.50973600	-1.83802400

1e'

M06-2X enthalpy: -1331.329256 a.u.

ATOM	X	Y	Z
H	4.48936700	1.47778100	-1.55829700
C	3.95508100	0.59111000	-1.23059900
C	2.50000400	-1.66848600	-0.32157100
C	4.42101900	-0.69318500	-1.49096900
C	2.76827800	0.73163000	-0.52550500
C	2.04186000	-0.38149700	-0.07469500
C	3.69457200	-1.79476900	-1.03628600
H	5.34594700	-0.84178700	-2.03728800
H	4.06845700	-2.79446000	-1.23612700
H	1.96305100	-2.53816400	0.02889300
C	2.06699600	1.95371700	-0.11509800
N	0.87607200	0.05690100	0.62471600
C	0.80382600	1.46006400	0.64711200
O	2.33172400	3.11286800	-0.29260800
O	-0.06763100	2.12932200	1.12667100
S	-0.35464300	-0.90321500	1.33694400
O	-0.52905000	-0.42542800	2.68757900
O	0.03048300	-2.27170200	1.04993500
C	-1.78043000	-0.49020900	0.37896500
C	-4.02326900	0.14013700	-1.12732700
C	-2.04631400	-1.22167100	-0.77628300
C	-2.60869400	0.54392700	0.80442000
C	-3.72756300	0.85090900	0.03919400
C	-3.17048600	-0.89620300	-1.52316300
H	-1.39015100	-2.03530500	-1.06846500
H	-2.36716000	1.09316700	1.70720700
H	-4.38255400	1.65831900	0.35389700
H	-3.39501800	-1.45749600	-2.42590300
C	-5.25652100	0.46056300	-1.93036700
H	-5.09550100	0.27318200	-2.99519000
H	-6.09719600	-0.16383800	-1.60814200
H	-5.55181600	1.50504200	-1.80429000

H radical

M06-2X enthalpy: -0.494305677 a.u.

ATOM	X	Y	Z
H	0.00000000	0.00000000	0.00000000

Supplementary Methods

General procedures and methods

Experiments involving moisture and/or air sensitive components were performed under a positive pressure of argon in oven-dried glassware equipped with a rubber septum inlet. Dried solvents and liquid reagents were transferred by oven-dried syringes or hypodermic syringe cooled to ambient temperature in a desiccator. Reactions mixtures were stirred in 10 mL sample vial with Teflon-coated magnetic stirring bars unless otherwise stated. Moisture in non-volatile reagents/compounds was removed in high *vacuo* by means of an oil pump and subsequent purging with nitrogen. Solvents were removed *in vacuo* under ~30 mmHg and heated with a water bath at 30–35 ^{o}C using rotary evaporator with aspirator. The condenser was cooled with running water at 0 ^{o}C.

All experiments were monitored by analytical thin layer chromatography (TLC). TLC was performed on pre-coated plates, 60 F_{254}. After elution, plate was visualized under UV illumination at 254 nm for UV active material. Further visualization was achieved by staining $Ce(SO_4)_2$ and anisaldehyde solution. For those using the aqueous stains, the TLC plates were heated on a hot plate.

Columns for flash chromatography (FC) contained *silica gel* 200–300 mesh. Columns were packed as slurry of *silica gel* in petroleum ether and equilibrated solution using the appropriate solvent system. The elution was assisted by applying pressure of about 2 atm with an air pump.

Instrumentations

Proton nuclear magnetic resonance (^{1}H NMR) and carbon NMR (^{13}C NMR) were recorded in $CDCl_3$ otherwise stated. Chemical shifts are reported in parts per million (ppm), using the residual solvent signal as an internal standard: $CDCl_3$ (^{1}H NMR: δ 7.26, singlet; ^{13}C NMR: δ 77.0, triplet). Multiplicities were given as: *s* (singlet), *d* (doublet), *t* (triplet), *q* (quartet), *quintet*, *m* (multiplets), *dd* (doublet of doublets), *dt* (doublet of triplets), and *br* (broad).

Coupling constants (*J*) were recorded in Hertz (Hz). The number of proton atoms (*n*) for a given resonance was indicated by *n*H. The number of carbon atoms (*n*) for a given resonance was indicated by *n*C. HRMS (Analyzer: TOF) was reported in units of mass of charge ratio (m/z). Mass samples were dissolved in CH_3CN (HPLC Grade) unless otherwise stated. Optical rotations were recorded on a polarimeter with a sodium lamp of wavelength 589 nm and reported as follows; $[\alpha]_{\lambda}^{T °C}$ (*c* = g/100 mL, solvent). Melting points were determined on a melting point apparatus.

Enantiomeric excesses were determined by chiral High Performance Liquid Chromatography (HPLC) analysis. UV detection was monitored at 254 nm and 210 nm at the same time. HPLC samples were dissolved in HPLC grade isopropanol (IPA) unless otherwise stated.

Materials

All commercial reagents were purchased with the highest purity grade. They were used without further purification unless specified. All solvents used, mainly petroleum ether (PE) and ethyl acetate (EtOAc) were distilled. Chlorobenzene, *p*-xylene, *o*-xylene, *m*-xylene and ethylbenzene were purchased with super dry grade. Toluene was freshly distilled from sodium/benzophenone before use. All compounds synthesized were stored in a −20 °C freezer and light-sensitive compounds were protected with aluminium foil.

General experimental procedures

(1) General procedure for asymmetric C(sp^3)−C(sp^3) coupling of *N*-tosyl isatins with toluene and its derivatives

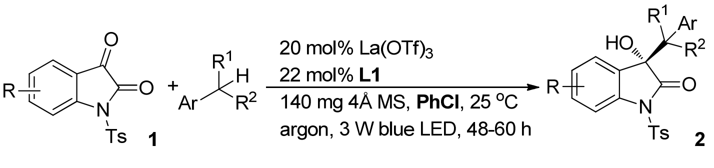

1 (0.1 mmol, 1.0 equiv), **L1** (0.022 mmol, 0.22 equiv), $La(OTf)_3$ (0.02 mmol, 0.2 equiv) and 4Å MS (140 mg) were added into a 25 mL Schlenk tube. Subsequently, toluene or its derivatives (5.0 mmol, 50.0 equiv) and PhCl (4.0 mL) were sequentially added, degassed

three times by freeze-pump-thaw method. The reaction mixture was stirred under an argon atmosphere at 30 °C (the temperature was maintained in an incubator) in dark for 2 hours, then irradiated by a 3 W blue LED (λ = 450−455 nm) from a 3.0 cm distance for another 48−60 hours at 25 °C. The reaction mixture was directly loaded onto a short silica gel column, followed by gradient elution with petroleum ether/ethyl acetate (20/1–5/1 ratio). Removing the solvent in vacuo, afforded products **2e-2ap**.

For the transformations in neat toluene or its derivatives: **1** (0.1 mmol, 1.0 equiv), **L1** (0.022 mmol, 0.22 equiv), $La(OTf)_3$ (0.02 mmol, 0.2 equiv) and 4Å MS (140 mg) were added into a 25 mL Schlenk tube. Subsequently, toluene or its derivatives (4.0 mL) was sequentially added, degassed three times by freeze-pump-thaw method. The reaction mixture was stirred under argon atmosphere at 30 °C (the temperature was maintained in an incubator) in dark for 2 hours, then irradiated by a 3 W blue LED (λ = 450−455 nm) from a 3.0 cm distance for another 48−60 hours at 25 °C. The reaction mixture was directly loaded onto a short silica gel column, followed by gradient elution with petroleum ether/ethyl acetate (20/1–5/1 ratio). Removing the solvent in vacuo, afforded products **2e-2i, 2l-2p, 2r-2u, 2w-2x, 2z-2aa, 2ac, 2ah-2ap**.

(2) General procedure for asymmetric C(sp^3)−C(sp^3) coupling of acenaphthoquinone with toluene and its derivatives

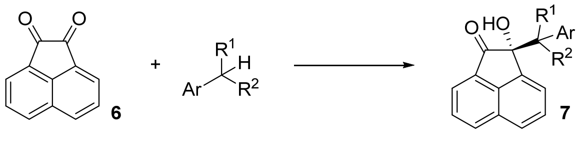

For the preparation of 7a: 6 (0.1 mmol, 1.0 equiv), **CPA-1** (0.02 mmol, 0.20 equiv) and 4Å MS (80 mg) were added into a 25 mL Schlenk tube. Subsequently, toluene (saturated aqueous solution) (2.0 mL) wad added, degassed three times by freeze-pump-thaw method. The reaction mixture was stirred under argon atmosphere at −5 °C (the temperature was maintained in an incubator) for 30 min in dark, then irradiated by 2*3 W blue LEDs (λ = 450−455 nm) from a 6.0 cm distance for another 60 hours at −5 °C. The reaction mixture was

directly loaded onto a short silica gel column, followed by elution with petroleum ether/dichloromethane (1/1 ratio) to petroleum ether/ethyl acetate (5/1 ratio). Removing the solvent in vacuo, afforded product **7a**.

For the preparation of 7b: 6 (0.1 mmol, 1.0 equiv), **CPA-1** (0.2 mmol, 0.20 equiv) and 4Å MS (140 mg) were added into a 25 mL Schlenk tube. Subsequently, 4-chlorotoluene (0.8 mL) and *tert*-butylbenzene (3.0 mL) were added, degassed three times by freeze-pump-thaw method. The reaction mixture was stirred under argon atmosphere at −5 °C (the temperature was maintained in an incubator) for 30 min in dark, then irradiated by 2*3 W blue LEDs (λ = 420−430 nm) from a 6.0 cm distance for another 60 hours at −5 °C. The reaction mixture was directly loaded onto a short silica gel column, followed by elution with petroleum ether/ dichloromethane (1/1 ratio) to petroleum ether/ethyl acetate (5/1 ratio). Removing the solvent in vacuo, afforded product **7b**.

For the preparation of 7c: 6 (0.1 mmol, 1.0 equiv), **CPA-1** (0.02 mmol, 0.20 equiv) and 4Å MS (140 mg) were added into a 25 mL Schlenk tube. Subsequently, *p*-xylene (0.8 mL) and *tert*-butylbenzene (3.0 mL) were added, degassed three times by freeze-pump-thaw method. The reaction mixture was stirred under argon atmosphere at −5 °C (the temperature was maintained in an incubator) for 30 min in dark, then irradiated by 2*3 W blue LEDs (λ = 450−455 nm) from a 6.0 cm distance for another 60 hours at −5 °C. The reaction mixture was directly loaded onto a short silica gel column, followed by elution with petroleum ether/ dichloromethane (1/1 ratio) to petroleum ether/ethyl acetate (5/1 ratio). Removing the solvent in vacuo, afforded product **7c**.

For the preparation of 7d: 6 (0.1 mmol, 1.0 equiv), **CPA-1** (0.02 mmol, 0.20 equiv) and 4Å MS (140 mg) were added into a 25 mL Schlenk tube. Subsequently, *m*-xylene (2.0 mL) was added, degassed three times by freeze-pump-thaw method. The reaction mixture was stirred under argon atmosphere at −5 °C (the temperature was maintained in an incubator) for 30 min in dark, then irradiated by 2*3 W blue LEDs (λ = 450−455 nm) from a 6.0 cm distance for

another 60 hours at −5 °C. The reaction mixture was directly loaded onto a short silica gel column, followed by elution with petroleum ether/ dichloromethane (1/1 ratio) to petroleum ether/ethyl acetate (5/1 ratio). Removing the solvent in vacuo, afforded product **7d**.

For the preparation of 7e: 6 (0.1 mmol, 1.0 equiv), **CPA-24** (0.02 mmol, 0.20 equiv), fluorine (0.5 mmol, 5.0 equiv), 4Å MS (140 mg) was added into a 25 mL Schlenk tube. Subsequently, PhCl (2.0 mL) were sequentially added, degassed three times by freeze-pump-thaw method. The reaction mixture was stirred under argon atmosphere at −20 °C (the temperature was maintained in an incubator) for 30 min without light, then irradiated by a 2*3 W blue LED (λ = 450−455 nm) from 6.0 cm distance for another 60 hours at −20 °C. The reaction mixture was directly loaded onto a short silica gel column, followed by elution with petroleum ether/ dichloromethane (1/1 ratio) to petroleum ether/ethyl acetate (5/1 ratio). Removing the solvent in vacuo, afforded product **7e**.

For the preparation of 7f: 6 (0.1 mmol, 1.0 equiv), **CPA-13** (0.02 mmol, 0.20 equiv), 4Å MS (140 mg) was added into a 25 mL Schlenk tube. Subsequently, cumene (2.0 mL) were sequentially added, degassed three times by freeze-pump-thaw method. The reaction mixture was stirred under argon atmosphere at 10 °C (the temperature was maintained in an incubator) for 30 min without light, then irradiated by a 2*3 W blue LED (λ = 450−455 nm) from 6.0 cm distance for another 60 hours at 10 °C. The reaction mixture was directly loaded onto a short silica gel column, followed by elution with petroleum ether/ dichloromethane (1/1 ratio) to petroleum ether/ethyl acetate (5/1 ratio). Removing the solvent in vacuo, afforded product **7f.**

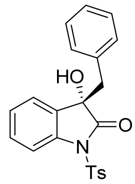

(*S*)-3-benzyl-3-hydroxy-1-tosylindolin-2-one (2e):

In PhCl: 58% yield, 98% ee; in neat: 76% yield, 97% ee.

Light yellow solid, Mp 116.9−118.3 °C, $[\alpha]_D^{22}$ +31.5 (*c* 1.0, $CHCl_3$). ^{1}H NMR (300 MHz, $CDCl_3$) δ 7.85 (d, *J* = 8.3 Hz, 2H), 7.74 (d, *J* = 8.2 Hz, 1H), 7.37−7.31 (m, 1H), 7.29 (s, 1H), 7.17 (dt, *J* = 14.9, 7.3 Hz, 3H), 7.06 (t, *J* = 7.3 Hz, 2H), 6.83 (d, *J* = 7.2 Hz, 2H), 3.16 (q, *J* = 13.1 Hz, 2H), 2.42 (s, 3H). ^{13}C NMR (75 MHz, $CDCl_3$) δ 176.1, 145.7, 138.6, 134.8, 132.5, 130.4, 130.2, 129.8, 128.2, 128.1, 127.9, 127.2, 124.9, 124.7, 113.4, 76.8, 45.5, 21.7. HRMS (ESI) m/z 262.0833 ($M+Na^+$), calc. for $C_{15}H_{13}NO_2Na$ 262.0838.

In PhCl: the ee was determined by HPLC analysis: CHIRALPAK ID (4.6 mm i.d. x 250 mm); Hexane/2-propanol = 80/20; flow rate 1.0 mL/min; 30 °C; 210 nm; retention time: 18.4 min (minor) and 19.4 min (major).

In neat: the ee was determined by HPLC analysis: CHIRALPAK ID (4.6 mm i.d. x 250 mm); Hexane/2-propanol = 80/20; flow rate 1.0 mL/min; 30 °C; 210 nm; retention time: 18.4 min (minor) and 19.4 min (major).

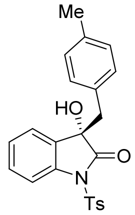

(*S*)-3-hydroxy-3-(4-methylbenzyl)-1-tosylindolin-2-one (2f):

In PhCl: 80% yield, 94% ee; in neat: 86 % yield, 96% ee.

Light yellow solid, Mp 168.9−170.3 °C, $[\alpha]_D^{22}$ +6.9 (*c* 1.0, $CHCl_3$). ^{1}H NMR (300 MHz, $CDCl_3$) δ 7.82 (d, *J* = 8.2 Hz, 2H), 7.72 (d, *J* = 8.2 Hz, 1H), 7.32 (s, 1H), 7.26−7.16 (m, 4H), 6.82 (d, *J* = 7.7 Hz, 2H), 6.68 (d, *J* = 7.8 Hz, 2H), 3.10 (q, *J* = 13.2 Hz, 2H), 2.85 (s, 1H), 2.40 (s, 3H), 2.22 (s, 3H). ^{13}C NMR (75 MHz, $CDCl_3$) δ 176.2, 145.6, 138.6, 136.7, 134.8, 130.3, 130.0, 129.7, 129.3, 128.8, 128.4, 127.9, 124.9, 124.7, 113.4, 76.8, 45.0, 21.7, 21.0. HRMS (ESI) m/z 430.1079 ($M+Na^+$), calc. for $C_{23}H_{21}NO_4SNa$ 430.1084.

In PhCl: the ee was determined by HPLC analysis: CHIRALPAK ID (4.6 mm i.d. x 250 mm); Hexane/2-propanol = 80/20; flow rate 1.0 mL/min; 30 °C; 210 nm; retention time: 17.2 min (minor) and 26.3 min (major).

In neat: the ee was determined by HPLC analysis: CHIRALCEL OZ−H (4.6 mm i.d. x 250

mm); Hexane/2-propanol = 80/20; flow rate 1.0 mL/min; 25 °C; 210 nm; retention time: 17.6 min (minor) and 26.1 min (major).

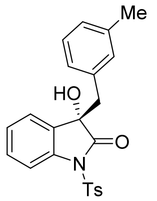

(*S*)-3-hydroxy-3-(3-methylbenzyl)-1-tosylindolin-2-one (2g):

In PhCl: 50% yield, 93% ee; in neat: 56% yield, 97% ee.

Light yellow solid, Mp 124.1−125.6 °C, $[\alpha]_D^{22}$ +7.4 (*c* 1.0, $CHCl_3$). ^{1}H NMR (300 MHz, $CDCl_3$) δ 7.83 (d, *J* = 8.3 Hz, 2H), 7.72 (d, *J* = 8.2 Hz, 1H), 7.36−7.28 (m, 1H), 7.26−7.12 (m, 4H), 6.94 (d, *J* = 5.0 Hz, 2H), 6.68 (s, 1H), 6.59 (d, *J* = 3.8 Hz, 1H), 3.11 (q, *J* = 13.1 Hz, 2H), 2.39 (s, 3H), 2.17 (s, 3H). ^{13}C NMR (75 MHz, $CDCl_3$) δ 176.2, 145.6, 138.5, 137.7, 134.8, 132.4, 131.0, 130.3, 129.8, 128.4, 128.0, 127.9, 127.8, 127.2, 124.9, 124.8, 113.4, 76.8, 45.5, 21.7, 21.2. HRMS (ESI) m/z 430.1079 (M+Na$^+$), calc. for $C_{23}H_{21}NO_4SNa$ 430.1084.

In PhCl: the ee was determined by HPLC analysis: CHIRALPAK ID (4.6 mm i.d. x 250 mm); Hexane/2-propanol = 85/15; flow rate 1.0 mL/min; 25 °C; 210 nm; retention time: 22.0 min (minor) and 24.0 min (major).

In neat: the ee was determined by HPLC analysis: CHIRALPAK ID (4.6 mm i.d. x 250 mm); Hexane/2-propanol = 85/15; flow rate 1.0 mL/min; 25 °C; 210 nm; retention time: 23.0 min (minor) and 25.2 min (major).

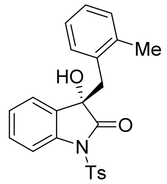

(*S*)-3-hydroxy-3-(2-methylbenzyl)-1-tosylindolin-2-one (2h):

In PhCl: 52% yield, 97% ee; in neat: 69 % yield, 89% ee.

Light yellow solid, Mp 152.6−154.1 °C, $[\alpha]_D^{22}$ +6.9 (*c* 1.0, $CHCl_3$). ^{1}H NMR (300 MHz, $CDCl_3$) δ 7.83 (d, *J* = 8.1 Hz, 2H), 7.72 (d, *J* = 8.1 Hz, 1H), 7.36−7.28 (m, 1H), 7.26−7.11 (m, 4H), 6.94 (d, *J* = 4.5 Hz, 2H), 6.67 (s, 1H), 6.59 (d, *J* = 3.7 Hz, 1H), 3.10 (q, *J* = 13.1 Hz, 2H), 2.39 (s, 3H), 2.17 (s, 3H). ^{13}C NMR (75 MHz, $CDCl_3$) δ 176.2, 145.6, 138.54, 137.7, 134.9, 132.4, 131.0, 130.3, 129.8, 128.4, 128.0, 127.9, 127.8, 127.2, 124.9, 124.8, 113.4, 76.8, 45.5, 21.7, 21.2. HRMS (ESI) m/z 430.1079 (M+Na$^+$), calc. for $C_{23}H_{21}NO_4SNa$ 430.1084.

In PhCl: the ee was determined by HPLC analysis: CHIRALPAK IE (4.6 mm i.d. x 250 mm); Hexane/2-propanol = 80/20; flow rate 1.0 mL/min; 30 °C; 210 nm; retention time: 22.8 min (major) and 37.7 min (minor).

In neat: the ee was determined by HPLC analysis: CHIRALPAK IE (4.6 mm i.d. x 250 mm); Hexane/2-propanol = 80/20; flow rate 1.0 mL/min; 25 °C; 210 nm; retention time: 23.5 min (major) and 41.5 min (minor).

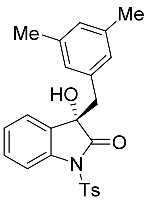

(*S*)-3-(3,5-dimethylbenzyl)-3-hydroxy-1-tosylindolin-2-one (2i):

In PhCl: 55% yield, 97% ee; in neat: 87 % yield, 94% ee.

Light yellow solid, Mp 152.6−154.1 °C, $[\alpha]_D^{22}$ +17.5 (*c* 1.0, $CHCl_3$). ^{1}H NMR (300 MHz, $CDCl_3$) δ 7.94 (d, *J* = 8.2 Hz, 2H), 7.82 (d, *J* = 8.1 Hz, 1H), 7.45−7.40 (m, 1H), 7.35 (d, *J* = 8.0 Hz, 2H), 7.27 (q, *J* = 7.6 Hz, 2H), 6.88 (s, 1H), 6.56 (s, 2H), 3.17 (q, *J* = 13.1 Hz, 2H), 2.79 (s, 1H), 2.49 (s, 3H), 2.24 (s, 6H). ^{13}C NMR (75 MHz, $CDCl_3$) δ 176.3, 145.6, 138.5, 137.5, 134.9, 132.2, 130.2, 129.8, 128.9, 128.6, 128.1, 127.7, 124.8, 124.8, 113.3, 76.8, 45.5, 26.9, 21.7, 21.1. HRMS (ESI) m/z 444.1238 (M+Na$^+$), calc. for $C_{24}H_{23}NO_4SNa$ 444.1240.

In PhCl: the ee was determined by HPLC analysis: CHIRALPAK ID (4.6 mm i.d. x 250 mm); Hexane/2-propanol = 80/20; flow rate 1.0 mL/min; 30 °C; 210 nm; retention time: 13.6 min (minor) and 16.1 min (major).

In neat: the ee was determined by HPLC analysis: CHIRALPAK ID (4.6 mm i.d. x 250 mm); Hexane/2-propanol = 80/20; flow rate 1.0 mL/min; 30 oC; 210 nm; retention time: 13.7 min (minor) and 16.2 min (major).

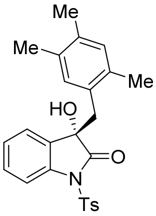

(*S*)-3-hydroxy-1-tosyl-3-(2,4,5-trimethylbenzyl)indolin-2-one (2j):

In PhCl: 54% yield, 97% ee.

White solid, Mp 186.9−187.7 °C, $[\alpha]_D^{22}$ −46.8 (*c* 1.0, $CHCl_3$). ^{1}H NMR (300 MHz, $CDCl_3$) δ 7.95 (d, *J* = 8.3 Hz, 2H), 7.87 (d, *J* = 8.2 Hz, 1H), 7.40 (t, *J* = 7.8 Hz, 1H), 7.31 (d, *J* = 8.9 Hz, 2H), 7.17 (t, *J* = 7.4 Hz, 1H), 7.08 (d, *J* = 7.3 Hz, 1H), 6.83

(d, J = 14.2 Hz, 2H), 3.09 (q, J = 14.0 Hz, 2H), 2.56 (s, 1H), 2.44 (s, 3H), 2.19 (d, J = 14.0 Hz, 6H), 1.94 (s, 3H). ^{13}C NMR (75 MHz, $CDCl_3$) δ 176.5, 145.7, 138.2, 135.7, 134.8, 134.7, 133.7, 132.3, 131.9, 130.2, 129.8, 129.0, 128.2, 127.9, 124.9, 124.7, 113.4, 76.3, 41.4, 21.7, 19.3, 19.1, 19.0. HRMS (ESI) m/z 458.1391 (M+Na$^+$), calc. for $C_{25}H_{25}NO_4SNa$ 458.1397.

The ee was determined by HPLC analysis: CHIRALCEL OZ−H (4.6 mm i.d. x 250 mm); Hexane/2-propanol = 80/20; flow rate 1.0 mL/min; 30 °C; 210 nm; retention time: 10.6 min (minor) and 15.8 min (major).

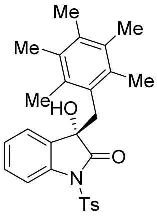

(*S*)-3-hydroxy-3-(2,3,4,5,6-pentamethylbenzyl)-1-tosylindolin-2-one (2k):

In PhCl: 52% yield, 83% ee.

White solid, Mp 186.0−187.3 °C, $[\alpha]_D^{22}$ −22.6 (*c* 1.0, $CHCl_3$). ^{1}H NMR (300 MHz, $CDCl_3$) δ 7.98 (d, J = 8.1 Hz, 2H), 7.91 (d, J = 8.1 Hz, 1H), 7.38 (t, J = 7.8 Hz, 1H), 7.29 (d, J = 8.2 Hz, 2H), 7.10 (t, J = 7.5 Hz, 1H), 6.96 (d, J = 7.5 Hz, 1H), 3.33 (dd, J = 40.6, 15.3 Hz, 2H), 2.57 (s, 1H), 2.40 (s, 3H), 2.25 (s, 3H), 2.12 (d, J = 45.8 Hz, 12H). ^{13}C NMR (75 MHz, $CDCl_3$) δ 176.6, 145.8, 137.9, 134.6, 134.4, 133.0, 130.1, 129.8, 129.7, 128.0, 126.7, 125.0, 124.7, 113.3, 76.9, 38.6, 21.7, 18.0, 17.0. HRMS (ESI) m/z 486.1706 (M+Na$^+$), calc. for $C_{27}H_{29}NO_4S$ Na 486.1710.

The ee was determined by HPLC analysis: CHIRALPAK ID (4.6 mm i.d. x 250 mm); Hexane/2-propanol = 80/20; flow rate 1.0 mL/min; 30 °C; 210 nm; retention time: 21.1 min (major) and 37.9 min (minor).

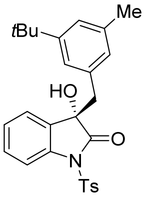

(*S*)-3-(3-(tert-butyl)-5-methylbenzyl)-3-hydroxy-1-tosylindolin-2-one (2l):

In PhCl: 69% yield, 97% ee; in neat: 37% yield, 63% ee.

Light yellow oil, $[\alpha]_D^{22}$ −39.1 (*c* 1.0, $CHCl_3$). ^{1}H NMR (300 MHz, $CDCl_3$) δ 7.73 (d, J = 8.2 Hz, 2H), 7.61 (d, J = 8.1 Hz, 1H), 7.24 (t, J = 7.8 Hz, 1H), 7.16 (d, J = 7.6 Hz, 3H), 7.12−7.08 (m, 1H), 6.90 (s, 1H), 6.56 (s, 1H), 6.39 (s, 1H), 3.13−2.99 (m, 3H), 2.31 (s, 3H), 2.14 (s, 3H), 1.03 (s, 9H). ^{13}C NMR (75 MHz, $CDCl_3$) δ 176.3, 150.5, 145.5, 138.5, 137.3, 134.8, 131.8, 130.2, 129.7, 128.5, 128.3, 127.6, 125.0, 124.8, 124.3, 113.3, 77.1, 45.8, 34.2,

31.1, 21.6, 21.4. HRMS (ESI) m/z 486.1709 (M+Na$^+$), calc. for $C_{27}H_{29}NO_4SNa$ 486.1710.

In PhCl: the ee was determined by HPLC analysis: CHIRALPAK ID (4.6 mm i.d. x 250 mm); Hexane/2-propanol = 80/20; flow rate 1.0 mL/min; 30 °C; 210 nm; retention time: 9.1 min (minor) and 10.1 min (major).

In neat: the ee was determined by HPLC analysis: CHIRALPAK ID (4.6 mm i.d. x 250 mm); Hexane/2-propanol = 80/20; flow rate 1.0 mL/min; 30 °C; 210 nm; retention time: 9.1 min (minor) and 10.1 min (major).

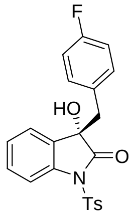

(*S*)-3-(4-fluorobenzyl)-3-hydroxy-1-tosylindolin-2-one (2m):

In PhCl: 55% yield, 97% ee; in neat: 64% yield, 95% ee.

Light yellow solid, Mp 129.6−129.8 °C, $[\alpha]_D^{22}$ −11.9 (*c* 1.0, $CHCl_3$). ^{1}H NMR (300 MHz, $CDCl_3$) δ 7.81 (d, *J* = 8.3 Hz, 2H), 7.76 (d, *J* = 8.2 Hz, 1H), 7.36 (m, 1H), 7.39−7.34 (s, 2H), 7.23−7.18 (m, 1H), 6.74−6.63 (m, 4H), 3.15 (q, *J* = 13.2 Hz, 2H), 2.85 (s, 1H), 2.44 (s, 3H); ^{13}C NMR (75 MHz, $CDCl_3$) δ 175.8, 161.9 (d, J_{C-F} = 245.9 Hz), 145.8, 138.7, 134.7, 131.6 (d, J_{C-F} = 8.1 Hz), 130.6, 129.8, 128.2 (d, J_{C-F} = 3.2 Hz), 128.0, 127.8, 125.1, 124.7, 114.9 (d, J_{C-F} = 21.3 Hz), 113.5, 44.5, 21.7. HRMS (ESI) m/z 434.0832 (M+Na$^+$), calc. for $C_{22}H_{18}FNO_4SNa$ 434.0833.

In PhCl: the ee was determined by HPLC analysis: CHIRALPAK ID (4.6 mm i.d. x 250 mm); Hexane/2-propanol = 80/20; flow rate 1.0 mL/min; 30 °C; 210 nm; retention time: 14.4 min (minor) and 16.1 min (major).

In neat: the ee was determined by HPLC analysis: CHIRALPAK ID (4.6 mm i.d. x 250 mm); Hexane/2-propanol = 80/20; flow rate 1.0 mL/min; 30 °C; 210 nm; retention time: 14.4 min (minor) and 16.2 min (major).

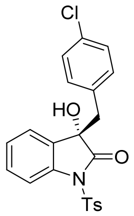

(*S*)-3-(4-chlorobenzyl)-3-hydroxy-1-tosylindolin-2-one (2n):

In PhCl: 43% yield, 94% ee; in neat: 52% yield, 97% ee.

White solid, Mp 155.6−157.2 °C, $[\alpha]_D^{22}$ −16.7 (*c* 1.0, $CHCl_3$). ^{1}H NMR (300 MHz,

$CDCl_3$) δ 7.68 (t, J = 7.6 Hz, 3H), 7.31–7.25 (m, 1H), 7.22 (s, 1H), 7.21–7.19 (m, 2H), 7.15 (d, J = 7.5 Hz, 1H), 6.79 (dd, J = 8.4, 3.4 Hz, 2H), 6.54 (dd, J = 8.1, 5.8 Hz, 2H), 3.06 (q, J = 13.0 Hz, 2H), 2.37 (s, 3H). ^{13}C NMR (75 MHz, $CDCl_3$) δ 175.8, 145.8, 138.7, 134.6, 133.0, 131.3, 131.0, 130.6, 129.8, 128.0, 127.9, 127.7, 125.1, 124.7, 113.5, 76.8, 44.4, 21.8. HRMS (ESI) m/z 450.0530 (M+Na$^+$), calc. for $C_{22}H_{18}ClNO_4SNa$ 450.0537.

In PhCl: the ee was determined by HPLC analysis: CHIRALPAK ID (4.6 mm i.d. x 250 mm); Hexane/2-propanol = 80/20; flow rate 1.0 mL/min; 30 °C; 210 nm; retention time: 13.3 min (minor) and 15.6 min (major).

In neat: the ee was determined by HPLC analysis: CHIRALPAK ID (4.6 mm i.d. x 250 mm); Hexane/2-propanol = 80/20; flow rate 1.0 mL/min; 30 oC; 210 nm; retention time: 13.3 min (minor) and 15.6 min (major).

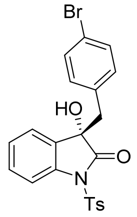

(*S*)-3-(4-bromobenzyl)-3-hydroxy-1-tosylindolin-2-one (2o):

In PhCl 49% yield, 92% ee; in neat: 59% yield, 97% ee.

White solid, Mp 174.7−176.0 °C, $[\alpha]_D^{22}$ −21.5 (c 1.0, $CHCl_3$). ^{1}H NMR (300 MHz, $CDCl_3$) δ 7.74−7.67 (m, 3H), 7.29 (t, J = 7.9 Hz, 1H), 7.23 (d, J = 8.2 Hz, 3H), 7.14 (t, J = 7.4 Hz, 1H), 6.97 (d, J = 8.3 Hz, 2H), 6.52 (d, J = 8.3 Hz, 2H), 3.05 (q, J = 13.0 Hz, 2H), 2.85 (s, 1H), 2.39 (s, 3H). ^{13}C NMR (75 MHz, $CDCl_3$) δ 175.7, 145.8, 138.7, 134.7, 131.7, 131.6, 131.0, 130.6, 129.8, 1279 127.8, 125.1, 124.7, 121.3, 113.6, 76.7, 44.5, 21.8. HRMS (ESI) m/z 494.0024 (M+Na$^+$), calc. for $C_{22}H_{18}BrNO_4SNa$ 494.0032.

In PhCl: the ee was determined by HPLC analysis: CHIRALPAK ID (4.6 mm i.d. x 250 mm); Hexane/2-propanol = 80/20; flow rate 1.0 mL/min; 30 °C; 210 nm; retention time: 15.3 min (minor) and 17.9 min (major).

In neat: the ee was determined by HPLC analysis: CHIRALPAK ID (4.6 mm i.d. x 250 mm); Hexane/2-propanol = 80/20; flow rate 1.0 mL/min; 30 oC; 210 nm; retention time: 14.0 min (minor) and 16.5 min (major).

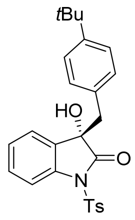

(*S*)-3-(4-(tert-butyl)benzyl)-3-hydroxy-1-tosylindolin-2-one (2p):

In PhCl: 65% yield, 92% ee; in neat: 56% yield, 74% ee.

Light yellow solid, Mp 113.7−115.1 °C, $[\alpha]_D^{22}$ −8.9 (*c* 1.0, $CHCl_3$). ^{1}H NMR (300 MHz, $CDCl_3$) δ 7.92 (d, *J* = 8.3 Hz, 2H), 7.77 (d, *J* = 8.2 Hz, 1H), 7.36−7.33 (m, 1H), 7.30 (d, *J* = 8.3 Hz, 2H), 7.18−7.14 (m, 4H), 6.84 (d, *J* = 8.2 Hz, 2H), 3.10 (q, *J* = 13.3 Hz, 2H), 2.42 (s, 3H), 1.27 (s, 9H). ^{13}C NMR (75 MHz, $CDCl_3$) δ 176.3, 150.2, 145.7, 138.5, 134.9, 130.3, 130.1, 129.8, 129.4, 128.6, 127.9, 125.1, 124.9, 124.7, 113.4, 44.9, 344, 31.3, 29.7, 21.7. HRMS (ESI) m/z 472.1552 (M+Na$^+$), calc. for $C_{26}H_{27}NO_4SNa$ 472.1553.

In PhCl: the ee was determined by HPLC analysis: CHIRALPAK ID (4.6 mm i.d. x 250 mm); Hexane/2-propanol = 80/20; flow rate 1.0 mL/min; 30 °C; 210 nm; retention time: 12.8 min (minor) and 15.3 min (major).

In neat: the ee was determined by HPLC analysis: CHIRALPAK ID (4.6 mm i.d. x 250 mm); Hexane/2-propanol = 80/20; flow rate 1.0 mL/min; 30 oC; 210 nm; retention time: 12.6 min (minor) and 15.4 min (major).

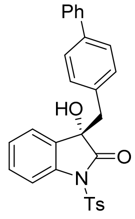

(*S*)-3-([1,1'-biphenyl]-4-ylmethyl)-3-hydroxy-1-tosylindolin-2-one (2q):

In PhCl: 51% yield, 96% ee.

White solid, Mp 169.1−170.4 °C, $[\alpha]_D^{22}$ −11.8 (*c* 1.0, $CHCl_3$). ^{1}H NMR (300 MHz, $CDCl_3$) δ 7.81 (d, *J* = 8.2 Hz, 2H), 7.76 (d, *J* = 8.3 Hz, 1H), 7.51 (d, *J* = 7.2 Hz, 2H), 7.43 (t, *J* = 7.5 Hz, 2H), 7.35 (d, *J* = 8.1 Hz, 2H), 7.30 (d, *J* = 7.3 Hz, 1H), 7.25−7.17 (m, 5H), 6.86 (d, *J* = 8.0 Hz, 2H), 3.22 (q, *J* = 13.1 Hz, 2H), 2.42 (s, 1H), 2.27 (s, 3H). ^{13}C NMR (75 MHz, $CDCl_3$) δ 176.0, 145.6, 140.5, 139.8, 138.7, 134.8, 131.6, 130.6, 130.4, 129.7, 128.7, 128.3, 127.8, 127.3, 126.9, 126.6, 125.0, 124.8, 113.5, 76.9, 45.0, 21.5. HRMS (ESI) m/z 492.1237 (M+Na$^+$), calc. for $C_{28}H_{23}NO_4SNa$ 492.1240.

The ee was determined by HPLC analysis: CHIRALPAK ID (4.6 mm i.d. x 250 mm); Hexane/2-propanol = 80/20; flow rate 1.0 mL/min; 30 °C; 210 nm; retention time: 24.1 min (minor) and 28.5 min (major).

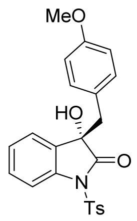

(*S*)-3-hydroxy-3-(4-methoxybenzyl)-1-tosylindolin-2-one (2r):

In PhCl: 78% yield, 97% ee; in neat: 82% yield, 93% ee.

Light yellow solid, Mp 141.2−142.3 °C, $[\alpha]_D^{22}$ +16.3 (*c* 1.0, $CHCl_3$). ^{1}H NMR (300 MHz, $CDCl_3$) δ 7.81 (d, *J* = 8.3 Hz, 2H), 7.73 (d, *J* = 8.2 Hz, 1H), 7.36−7.30 (m, 1H), 7.26−7.18 (m, 4H), 6.72 (d, *J* = 8.6 Hz, 2H), 6.56 (d, *J* = 8.6 Hz, 2H), 3.72 (s, 3H), 3.11 (q, *J* = 13.3 Hz, 2H), 2.41 (s, 3H). ^{13}C NMR (75 MHz, $CDCl_3$) δ 176.2, 158.6, 145.6, 138.6, 134.8, 131.2, 130.3, 129.7, 128.4, 127.8, 124.9, 124.7, 124.5, 113.5, 113.4, 76.9, 55.0, 44.5, 21.7. HRMS (ESI) m/z 446.1031 ($M+Na^+$), calc. for $C_{23}H_{21}NO_5SNa$ 446.1033.

In PhCl: the ee was determined by HPLC analysis: CHIRALPAK ID (4.6 mm i.d. x 250 mm); Hexane/2-propanol = 80/20; flow rate 1.0 mL/min; 30 °C; 210 nm; retention time: 25.6 min (minor) and 28.7 min (major).

In neat: the ee was determined by HPLC analysis: CHIRALPAK ID (4.6 mm i.d. x 250 mm); Hexane/2-propanol = 80/20; flow rate 1.0 mL/min; 30 oC; 210 nm; retention time: 25.3 min (minor) and 28.6 min (major).

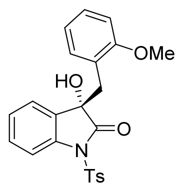

(*S*)-3-hydroxy-3-(2-methoxybenzyl)-1-tosylindolin-2-one (2s):

In PhCl: 45% yield, 92% ee; in neat: 82% yield, 97% ee.

Light yellow solid, Mp 168.2−170.1 °C, $[\alpha]_D^{22}$ −55.3 (*c* 1.0, $CHCl_3$). ^{1}H NMR (300 MHz, $CDCl_3$) δ 7.95 (d, *J* = 8.3 Hz, 2H), 7.84 (d, *J* = 8.2 Hz, 1H), 7.34−7.22 (m, 4H), 7.06 (t, *J* = 7.5 Hz, 1H), 6.91 (d, *J* = 6.7 Hz, 1H), 6.86 (d, *J* = 8.3 Hz, 1H), 6.82 (d, *J* = 3.9 Hz, 2H), 3.78 (s, 3H), 3.47 (d, *J* = 13.8 Hz, 1H), 2.86 (d, *J* = 13.8 Hz, 1H), 2.38 (s, 3H). ^{13}C NMR (75 MHz, $CDCl_3$) δ 176.7, 157.7, 145.6, 138.1, 134.9, 132.5, 129.9, 129.8, 129.2, 129.1, 127.9, 125.0, 124.5, 121.8, 120.7, 113.2, 110.6, 76.9, 55.4, 39.6. HRMS (ESI) m/z 446.1031 ($M+Na^+$), calc. for $C_{23}H_{21}NO_5SNa$ 446.1033.

In PhCl: the ee was determined by HPLC analysis: CHIRALPAK ID (4.6 mm i.d. x 250 mm); Hexane/2-propanol = 80/20; flow rate 1.0 mL/min; 30 °C; 210 nm; retention time: 24.3 min (minor) and 25.8 min (major).

In neat: the ee was determined by HPLC analysis: CHIRALPAK ID (4.6 mm i.d. x 250 mm); Hexane/2-propanol = 80/20; flow rate 1.0 mL/min; 30 oC; 210 nm; retention time: 24.9 min (minor) and 26.5 min (major).

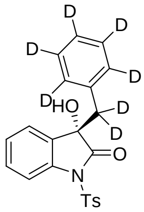

(*S*)-3-hydroxy-3-((phenyl-d5)methyl-d2)-1-tosylindolin-2-one (2t):

In PhCl: 36% yield, 97% ee; in neat: 71% yield, 96% ee.

Light yellow solid, Mp 79.2−80.4 °C, $[\alpha]_D^{22}$ −55.3 (*c* 1.0, CHCl$_3$). ^{1}H NMR (300 MHz, CDCl$_3$) δ 7.84 (d, *J* = 8.3 Hz, 2H), 7.73 (d, *J* = 8.2 Hz, 1H), 7.37−7.31 (m, 1H), 7.28 (s, 1H), 7.25 (s, 1H), 7.23−7.15 (m, 2H), 2.42 (s, 3H). ^{13}C NMR (75 MHz, CDCl$_3$) δ 176.2, 145.6, 138.5, 134.7, 132.2, 130.3, 129.8, 128.2, 127.8, 124.9, 124.7, 113.4, 76.8, 21.7. HRMS (ESI) m/z 423.1361 (M+Na$^+$), calc. for $C_{22}H_{12}D_7NO_4SNa$ 423.1366.

In PhCl: the ee was determined by HPLC analysis: CHIRALPAK ID (4.6 mm i.d. x 250 mm); Hexane/2-propanol = 80/20; flow rate 1.0 mL/min; 30 °C; 210 nm; retention time: 18.8 min (minor) and 19.8 min (major).

In neat: the ee was determined by HPLC analysis: CHIRALPAK ID (4.6 mm i.d. x 250 mm); Hexane/2-propanol = 80/20; flow rate 1.0 mL/min; 30 oC; 210 nm; retention time: 18.8 min (minor) and 19.8 min (major).

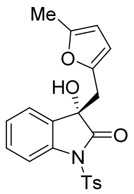

(*S*)-3-hydroxy-3-((5-methylfuran-2-yl)methyl)-1-tosylindolin-2-one (2u):

In PhCl: 56% yield, 78% ee; in neat: 71% yield, 50% ee.

Yellow oil, $[\alpha]_D^{22}$ −20.1 (*c* 1.0, CHCl$_3$). ^{1}H NMR (300 MHz, CDCl$_3$) δ 7.93 (d, *J* = 8.2 Hz, 2H), 7.81 (d, *J* = 8.2 Hz, 1H), 7.38−7.32 (m, 1H), 7.29 (d, *J* = 8.2 Hz, 2H), 7.23−7.14 (m, 2H), 5.69 (d, *J* = 3.6 Hz, 2H), 3.16 (s, 2H), 2.40 (s, 3H), 2.12 (s, 3H). ^{13}C NMR (75 MHz, CDCl$_3$) δ 175.8, 151.9, 145.7, 145.6, 138.5, 134.8, 130.3, 129.8, 128.5, 127.9 125.0,

124.6, 113.3, 109.7, 106.2, 75.7, 37.8, 21.6, 13.4. HRMS (ESI) m/z 420.0867 (M+Na$^+$), calc. for $C_{21}H_{19}NO_5SNa$ 420.0876.

In PhCl: the ee was determined by HPLC analysis: CHIRALPAK IE (4.6 mm i.d. x 250 mm); Hexane/2-propanol = 80/20; flow rate 1.0 mL/min; 30 ^oC; 210 nm; retention time: 27.0 min (major) and 36.4 min (minor).

In neat: the ee was determined by HPLC analysis: CHIRALPAK IE (4.6 mm i.d. x 250 mm); Hexane/2-propanol = 80/20; flow rate 1.0 mL/min; 30 oC; 210 nm; retention time: 27.1 min (major) and 36.6 min (minor).

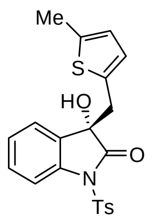

(*S*)-3-hydroxy-3-((5-methylthiophen-2-yl)methyl)-1-tosylindolin-2-one (2v):

In PhCl: 65% yield, 87% ee.

Yellow solid, Mp 72.2–73.1 °C, $[\alpha]_D^{22}$ −50.5 (*c* 1.0, $CHCl_3$). ^{1}H NMR (300 MHz, $CDCl_3$) δ 7.84 (dd, *J* = 13.1, 8.2 Hz, 3H), 7.39 (t, *J* = 7.7 Hz, 1H), 7.26 (t, *J* = 7.2 Hz, 3H), 7.22−7.17 (m, 1H), 6.33 (d, *J* = 10.7 Hz, 2H), 3.30 (s, 2H), 2.98 (s, 1H), 2.42 (s, 3H), 2.30 (s, 3H). ^{13}C NMR (75 MHz, $CDCl_3$) δ 175.7, 145.6, 139.6, 138.9, 134.7, 131.4, 130.6, 129.7, 128.1, 128.0, 127.8, 125.1, 124.7, 124.7, 113.5, 76.2, 39.5, 21.7, 15.2. HRMS (ESI) m/z 436.0647 (M+Na$^+$), calc. for $C_{21}H_{19}NO_4S_2Na$ 436.0648.

The ee was determined by HPLC analysis: CHIRALPAK IE (4.6 mm i.d. x 250 mm); Hexane/2-propanol = 80/20; flow rate 1.0 mL/min; 30 °C; 210 nm; retention time: 30.2 min (major) and 35.0 min (minor).

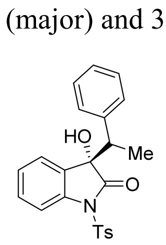

(*S*)-3-hydroxy-3-((*S*)-1-phenylethyl)-1-tosylindolin-2-one (2w):

In PhCl: 46% yield, 1.5:1 dr; in neat: 71% yield, 1.4:1 dr.

Major diastereomer: in PhCl: 98% ee; in neat: 97% ee.

Light yellow oil, $[\alpha]_D^{22}$ +15.6 (*c* 1.0, $CHCl_3$). ^{11}H NMR (300 MHz, $CDCl_3$) δ 7.98−7.95 (m, 2H), 7.79 (d, *J* = 8.1 Hz, 1H), 7.33 (t, *J* = 8.5 Hz, 3H), 7.23 (d, *J* = 5.3 Hz, 3H), 7.07 (dd, *J* = 17.1, 7.4 Hz, 3H), 6.79 (d, *J* = 7.5 Hz, 1H), 3.33 (q, *J* = 6.9 Hz, 1H), 2.77 (s, 1H), 2.42 (s, 3H), 1.18 (d, *J* = 7.1 Hz, 3H). ^{13}C NMR (75 MHz, $CDCl_3$) δ 176.6, 145.8, 138.9, 138.0, 134.8,

130.2, 129.8, 129.2, 128.0, 127.8, 127.4, 127.3, 125.5, 124.5, 113.1, 78.6, 47.4, 21.7, 13.6. HRMS (ESI) m/z 430.1083 (M+Na$^+$), calc. for $C_{23}H_{21}NO_4SNa$ 430.1084.

In PhCl: the ee was determined by HPLC analysis: CHIRALCEL OD−H (4.6 mm i.d. x 250 mm); Hexane/2-propanol = 80/20; flow rate 1.0 mL/min; 25 °C; 210 nm; retention time: 11.3 min (major) and 16.3 min (minor).

In neat: the ee was determined by HPLC analysis: CHIRALCEL OD−H (4.6 mm i.d. x 250 mm); Hexane/2-propanol = 80/20; flow rate 1.0 mL/min; 25 °C; 210 nm; retention time: 11.3 min (major) and 16.4 min (minor).

Minor diastereomer: in PhCl: 98% ee; in neat: 98% ee.

Light yellow solid, Mp 132.1−132.7 °C, $[\alpha]_D^{22}$ +19.1 (*c* 1.0, $CHCl_3$).^{1}H NMR (300 MHz, $CDCl_3$) δ 7.98−7.95 (m, 2H), 7.79 (d, *J* = 8.1 Hz, 1H), 7.33 (t, *J* = 8.5 Hz, 3H), 7.23 (d, *J* = 5.3 Hz, 3H), 7.07 (dd, *J* = 17.1, 7.4 Hz, 3H), 6.79 (d, *J* = 7.5 Hz, 1H), 3.33 (q, *J* = 6.9 Hz, 1H), 2.77 (s, 1H), 2.42 (s, 3H), 1.18 (d, *J* = 7.1 Hz, 3H). ^{13}C NMR (75 MHz, $CDCl_3$) δ 176.4, 145.6, 143.7, 139.2, 134.8, 134.7, 130.3, 129.7, 128.9, 128.0, 127.8, 127.6, 124.9, 124.8, 113.2, 78.6, 48.5, 28.4, 21.7, 15.4, 14.2.

In PhCl: the ee was determined by HPLC analysis: CHIRALCEL OD−H (4.6 mm i.d. x 250 mm); Hexane/2-propanol = 80/20; flow rate 1.0 mL/min; 25 °C; 210 nm; retention time:11.6 min (major) and 24.7 min (minor).

In neat: the ee was determined by HPLC analysis: CHIRALCEL OD−H (4.6 mm i.d. x 250 mm); Hexane/2-propanol = 80/20; flow rate 1.0 mL/min; 25 °C; 210 nm; retention time: 11.4 min (major) and 24.3 min (minor).

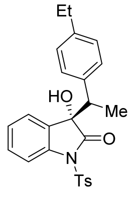

(*S*)-3-((*S*)-1-(4-ethylphenyl)ethyl)-3-hydroxy-1-tosylindolin-2-one (2x):

In PhCl: 59% yield, 1.2:1 dr; in neat: 59% yield, 1.3:1 dr.

Major diastereomer: in PhCl: 99% ee; in neat: 98% ee.

Light yellow oil, $[\alpha]_D^{22}$ +18.7 (*c* 1.0, $CHCl_3$). ^{1}H NMR (300 MHz, $CDCl_3$) δ 7.97 (d, *J* = 8.2 Hz, 2H), 7.81 (d, *J* = 8.2 Hz, 1H), 7.34 (t, *J* = 9.5 Hz, 3H), 7.11−7.05 (m, 3H), 6.97 (d, *J* = 8.0 Hz,

2H), 6.78 (d, J = 7.4 Hz, 1H), 3.30 (q, J = 7.1 Hz, 1H), 2.62 (q, J = 7.6 Hz, 2H), 2.42 (s, 3H), 1.22 (t, J = 7.6 Hz, 3H), 1.13 (d, J = 7.1 Hz, 3H). ^{13}C NMR (75 MHz, $CDCl_3$) δ 176.7, 145.8, 143.5, 139.0, 135.1, 134.8, 130.2, 129.8, 129.2, 128.0, 127.4, 127.3, 125.6, 124.5, 113.1, 78.6, 45.0, 28.4, 21.7, 15.4, 13.7. HRMS (ESI) m/z 458.1393 ($M+Na^+$), calc. for $C_{25}H_{25}NO_4SNa$ 458.1397.

Minor diastereomer: in PhCl: 90% ee; in neat: 81% ee.

Light yellow solid, Mp 78.3−79.9 °C, $[\alpha]_D^{22}$ −8.22 (*c* 1.0, $CHCl_3$). ^1H NMR (300 MHz, $CDCl_3$) δ 7.90 (d, J = 8.2 Hz, 2H), 7.81 (d, J = 8.1 Hz, 1H), 7.43 (t, J = 7.6 Hz, 2H), 7.32−7.29 (m, 3H), 7.04 (d, J = 7.9 Hz, 2H), 6.90 (d, J = 8.0 Hz, 2H), 3.27 (q, J = 7.1 Hz, 1H), 2.71−2.60 (m, 3H), 2.45 (s, 3H), 1.39 (d, J = 7.2 Hz, 3H), 1.25 (t, J = 7.6 Hz, 3H). ^{13}C NMR (75 MHz, $CDCl_3$) δ 176.4, 145.6, 143.7, 139.1, 134.8, 134.7, 130.3 129.7, 128.9, 128.0, 127.8, 127.6, 124.9 124.8, 113.2, 78.6, 48.5, 28.4, 21.7, 15.4, 14.2.

In PhCl: the ee was determined by HPLC analysis: CHIRALPAK IE (4.6 mm i.d. x 250 mm); Hexane/2-propanol = 80/20; flow rate 1.0 mL/min; 30 °C; 210 nm; retention time: 20.9 min (major) and 25.9 min (minor).

In neat: the ee was determined by HPLC analysis: CHIRALPAK IE (4.6 mm i.d. x 250 mm); Hexane/2-propanol = 80/20; flow rate 1.0 mL/min; 30 °C; 210 nm; retention time: 21.0 min (minor) and 24.0 min (major).

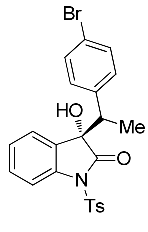

(*S*)-3-((*S*)-1-(4-bromophenyl)ethyl)-3-hydroxy-1-tosylindolin-2-one (2y):

In PhCl: 42% yield, 1.3:1 dr.

Major diastereomer: 96% ee.

Light yellow solid, Mp 65.5−67.1 °C, $[\alpha]_D^{22}$ −54.5 (*c* 1.0, $CHCl_3$). ^1H NMR (300 MHz, $CDCl_3$) δ 7.92 (d, J = 8.3 Hz, 2H), 7.80 (d, J = 8.2 Hz, 1H), 7.35 (dd, J = 14.6, 4.6 Hz, 3H), 7.25 (d, J = 8.4 Hz, 2H), 7.10 (t, J = 7.5 Hz, 1H), 6.86 (d, J = 8.4 Hz, 2H), 6.81 (d, J = 7.5 Hz, 1H), 3.29 (q, J = 7.0 Hz, 1H), 2.43 (s, 3H), 1.15 (dd, J = 7.1, 2.8 Hz, 3H). ^{13}C NMR (75 MHz, $CDCl_3$) δ 176.3, 145.9, 139.0, 137.2, 134.8, 130.9, 130.8, 130.4, 129.8, 128.0,

127.2, 125.3, 124.6, 121.3, 113.3, 78.3, 46.8, 21.7, 13.4. HRMS (ESI) m/z 508.0181 ($M+Na^+$), calc. for $C_{23}H_{20}BrNO_4SNa$ 508.0189.

The ee was determined by HPLC analysis: CHIRALCEL OD−H (4.6 mm i.d. x 250 mm); Hexane/2-propanol = 80/20; flow rate 1.0 mL/min; 30 °C; 210 nm; retention time: 9.9 min (major) and 11.2 min (minor).

Minor diastereomer: 95% ee.

Light yellow solid, Mp 72.0−71.3 °C, $[\alpha]_D^{22}$ +41.4 (*c* 1.0, $CHCl_3$). ^{1}H NMR (300 MHz, $CDCl_3$) δ 7.95 (d, *J* = 8.1 Hz, 2H), 7.82 (d, *J* = 8.4 Hz, 1H), 7.36 (t, *J* = 9.0 Hz, 3H), 7.29−7.27 (m, 2H), 7.12 (t, *J* = 7.6 Hz, 1H), 6.86 (dd, *J* = 13.3, 7.2 Hz, 3H), 3.31 (q, *J* = 7.0 Hz, 1H), 2.45 (s, 3H), 1.17 (d, *J* = 7.1 Hz, 3H). ^{13}C NMR (75 MHz, $CDCl_3$) δ 176.2, 145.7, 139.2, 137.1, 134.7, 131.5, 131.0, 130.6 130.3, 129.8, 127.8, 127.2, 127.1, 125.1, 124.8, 121.5, 113.4, 78.4, 47.9, 21.8, 14.2.

The ee was determined by HPLC analysis: CHIRALCEL OD−H (4.6 mm i.d. x 250 mm); Hexane/2-propanol = 80/20; flow rate 1.0 mL/min; 30 °C; 210 nm; retention time: 9.0 min (major) and 11.3 min (minor).

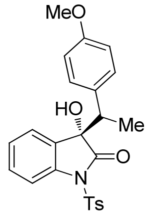

(*S*)-3-hydroxy-3-((*S*)-1-(4-methoxyphenyl)ethyl)-1-tosylindolin-2-one (2z):

In PhCl: 51% yield, 1.6:1 dr; in neat: 81% yield, 1.5:1 dr.

Major diastereomer: in PhCl: 98% ee; in neat: 97% ee (cannot be separated).

Minor diastereomer: in PhCl: 97% ee; in neat: 94% ee (cannot be separated).

Light yellow oil. ^{1}H NMR (300 MHz, $CDCl_3$) δ 7.85 (d, *J* = 8.2 Hz, 1.1H), 7.68 (dd, *J* = 14.8, 8.3 Hz, 1.7H), 7.29−7.26 (m, 1H), 7.21 (d, *J* = 8.2 Hz, 1.5H), 7.16 (s, 1H), 7.12−7.09 (m, 1H), 6.98 (t, *J* = 7.5 Hz, 1.1H), 6.86 (d, *J* = 8.5 Hz, 1.1H), 6.70−6.63 (m, 2.5H), 6.53 (d, *J* = 8.5 Hz, 0.8H), 3.68 (s, 1.8H), 3.64 (s, 1.1H), 3.22−3.08 (m, 1H), 2.63 (s, 1H), 2.31 (s, 1.7H), 2.30 (s, 1.1H), 1.25 (d, *J* = 7.2 Hz, 1.2H), 1.02 (d, *J* = 7.1 Hz, 1.8H). ^{13}C NMR (75 MHz, $CDCl_3$) δ 176.6, 176.4, 158.9, 158.8, 145.8, 145.6, 139.1, 139.0 134.8, 134.8, 130.3, 130.2, 130.2, 130.0, 129.8, 129.8, 129.7, 128.0, 127.8, 127.4, 125.5, 124.9, 124.8, 124.5, 113.5, 113.2, 113.1, 78.6,

78.6, 55.2, 55.1, 47.9, 46.6, 21.7, 21.7, 14.4, 13.8. HRMS (ESI) m/z 460.1183 ($M+Na^+$), calc. for $C_{24}H_{23}NO_5SNa$ 460.1189.

In PhCl: the ee was determined by HPLC analysis: CHIRALPAK AD−H (4.6 mm i.d. x 250 mm); Hexane/2-propanol = 80/20; flow rate 1.0 mL/min; 30 °C; 210 nm; retention time: major diastereomer: 17.2 min (major) and 44.1 min (minor); minor diastereomer: 12.4 min (minor) and 22.0 min (major).

In neat: the ee was determined by HPLC analysis: CHIRALPAK AD−H (4.6 mm i.d. x 250 mm); Hexane/2-propanol = 80/20; flow rate 1.0 mL/min; 30 °C; 210 nm; retention time: major diastereomer: 17.3 min (major) and 44.2 min (minor); minor diastereomer: 12.6 min (minor) and 22.1 min (major);

(*S*)-3-hydroxy-3-((*S*)-1-phenylpropyl)-1-tosylindolin-2-one (2aa):

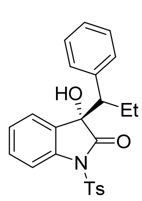

In PhCl: 51% yield, 2.9:1 dr; in neat: 62% yield, 1.5:1 dr.

Major diastereomer: in PhCl: 92% ee; in neat: 96% ee (cannot be separated).

Minor diastereomer: in PhCl: 88% ee; in neat: 96% ee (cannot be separated).

Light yellow oil, ^1H NMR (300 MHz, $CDCl_3$) δ 7.96 (d, J = 8.3 Hz, 1.5H), 7.79 (d, J = 8.5 Hz, 0.5H), 7.74 (t, J = 8.8 Hz, 1H), 7.38 (t, J = 7.6 Hz, 0.5H), 7.31 (t, J = 7.9 Hz, 2.5H), 7.22−7.15 (m, 3H), 7.10 (t, J = 7.6 Hz, 1H), 7.01−6.94 (m, 2.5H), 6.82 (d, J = 7.2 Hz, 0.5H), 3.06 (dd, J = 10.5, 5.0 Hz, 0.75H), 2.95 (dd, J = 12.3, 2.8 Hz, 0.25H), 2.70 (s, 0.75H), 2.65 (s, 0.25H), 2.42 (s, 2.2H), 2.40 (s, 0.7H), 1.68−1.62 (m, 2H), 0.67 (dt, J = 14.5, 7.3 Hz, 3H). ^{13}C NMR (75 MHz, $CDCl_3$) δ 176.6, 176.4, 145.8, 145.5, 139.2, 138.8, 135.7, 135.6, 134.8, 130.4, 130.2, 129.8, 129.8, 129.8, 129.4, 128.1, 128.1, 128.0, 127.8, 127.6, 127.5, 125.3, 125.0, 124.8, 124.5, 113.2, 78.6, 78.4, 56.9, 56.0, 21.7, 21.7, 20.8, 20.0, 12.1, 11.8. HRMS (ESI) m/z 444.1238 ($M+Na^+$), calc. for $C_{24}H_{23}NO_4SNa$ 444.1240.

In PhCl: the ee was determined by HPLC analysis: CHIRALPAK IE (4.6 mm i.d. x 250 mm); Hexane/2-propanol = 80/20; flow rate 1.0 mL/min; 30 °C; 210 nm; retention time: major

diastereomer: 19.6 min (major) and 25.5 min (minor); minor diastereomer: 21.0 min (minor) and 23.2 min (major);

In neat: the ee was determined by HPLC analysis: CHIRALPAK IE (4.6 mm i.d. x 250 mm); Hexane/2-propanol = 80/20; flow rate 1.0 mL/min; 30 °C; 210 nm; retention time: major diastereomer: 19.7 min (major) and 25.4 min (minor); minor diastereomer: 21.3 min (minor) and 23.3 min (major).

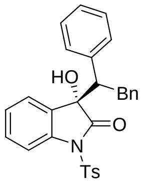

(*S*)-3-((*S*)-1,2-diphenylethyl)-3-hydroxy-1-tosylindolin-2-one (2ab):

In PhCl: 52% yield. 1.6:1 dr.

Major diastereomer: 95% ee (cannot be separated).

Minor diastereomer: 88% ee (cannot be separated).

Light yellow solid.^{1}H NMR (300 MHz, $CDCl_3$) δ 8.07 (d, *J* = 8.2 Hz, 1H), 7.88−7.81 (m, 2H), 7.62 (d, *J* = 7.5 Hz, 0.5H), 7.53 (t, *J* = 7.7 Hz, 0.5H), 7.41 (d, *J* = 7.3 Hz, 2H), 7.36 (t, *J* = 5.1 Hz, 2H), 7.16 (dd, *J* = 15.6, 8.1 Hz, 5H), 7.06 (dd, *J* = 14.6, 7.3 Hz, 3H), 6.97 (d, *J* = 7.0 Hz, 1H), 6.80 (d, *J* = 7.4 Hz, 1H), 3.61 (d, *J* = 11.3 Hz, 1H), 3.46 (d, *J* = 11.9 Hz, 0.4H), 3.25 (dd, *J* = 13.9, 3.6 Hz, 0.6H), 3.07 (dt, *J* = 20.3, 10.0 Hz, 2H), 2.48 (d, *J* = 7.2 Hz, 3H). ^{13}C NMR (75 MHz, $CDCl_3$) δ 176.2, 145.9, 145.5, 139.2, 139.1, 139.1, 138.6, 135.4, 135.2, 134.7, 134.7, 130.5, 130.3, 129.8, 129.8, 129.6, 129.4, 128.9, 128.9, 128.3, 128.1, 128.1, 128.0, 127.8, 127.8, 127.6, 127.4, 127.3, 126.0, 125.2, 124.9, 124.7, 113.4, 113.3, 78.7, 78.4, 56.9, 56.1, 34.5, 33.4, 21.7, 21.6. HRMS (ESI) m/z 506.1392 (M+Na^+), calc. for $C_{29}H_{25}NO_4SNa$ 506.1397.

The ee was determined by HPLC analysis: CHIRALPAK IE (4.6 mm i.d. x 250 mm); Hexane/2-propanol = 80/20; flow rate 1.0 mL/min; 30 °C; 254 nm; retention time: major diastereomer: 18.7 min (major) and 23.5 min (minor); minor diastereomer: 19.7 min (minor) and 22.5 min (major).

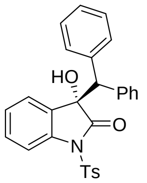

(*S*)-3-benzhydryl-3-hydroxy-1-tosylindolin-2-one (2ac):

In PhCl: 50% yield, 97% ee; in neat: 77% yield; 99% ee.

White solid, Mp 165.1−166.4 °C, $[\alpha]_D^{22}$ −24.3 (*c* 1.0, $CHCl_3$). ^1H NMR (300 MHz, $CDCl_3$) δ 7.79 (d, *J* = 8.3 Hz, 3H), 7.37 (t, *J* = 7.9 Hz, 1H), 7.22 (d, *J* = 7.2 Hz, 6H), 7.16 (s, 5H), 7.09 (t, *J* = 7.5 Hz, 1H), 6.90 (d, *J* = 7.5 Hz, 1H), 4.47 (s, 1H), 2.41 (s, 3H). ^{13}C NMR (75 MHz, $CDCl_3$) δ 175.6, 145.5, 139.2, 136.7, 134.8 130.6, 129.9, 129.7, 128.5, 128.1, 128.1, 127.8, 127.6, 127.4, 125.6, 124.7, 113.3, 78.1, 60.1, 21.7. HRMS (ESI) m/z 492.1237 ($M+Na^+$), calc. for $C_{28}H_{23}NO_4SNa$ 492.1240.

In PhCl: the ee was determined by HPLC analysis: CHIRALPAK ID (4.6 mm i.d. x 250 mm); Hexane/2-propanol = 80/20; flow rate 1.0 mL/min; 25 °C; 210 nm; retention time: 33.5 min (major) and 40.6 min (minor).

In neat: the ee was determined by HPLC analysis: CHIRALPAK ID (4.6 mm i.d. x 250 mm); Hexane/2-propanol = 80/20; flow rate 1.0 mL/min; 25 °C; 210 nm; retention time: 35.8 min (major) and 43.7 min (minor).

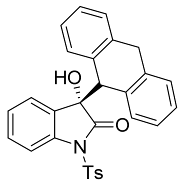

(*S*)-3-(9,10-dihydroanthracen-9-yl)-3-hydroxy-1-tosylindolin-2-one (2ad):

In PhCl: 63% yield, 97% ee.

White solid, Mp 85.6−86.5 °C, $[\alpha]_D^{22}$ +17.6 (*c* 1.0, $CHCl_3$). ^1H NMR (300 MHz, $CDCl_3$) δ 7.82 (d, *J* = 8.3 Hz, 2H), 7.55 (d, *J* = 8.2 Hz, 1H), 7.39−7.27 (m, 2H), 7.25−7.00 (m, 10H), 6.77 (d, *J* = 7.0 Hz, 1H), 4.44 (s, 1H), 3.48 (dd, *J* = 55.0, 19.5 Hz, 2H), 3.05 (s, 1H), 2.38 (s, 3H). ^{13}C NMR (75 MHz, $CDCl_3$) δ 176.6, 145.6, 138.9, 137.7, 137.5, 134.8, 131.6, 130.9, 130.4, 130.1, 130.0, 129.7, 128.0, 127.9, 127.8, 127.6, 127.5, 127.3, 126.3, 125.7, 125.2, 124.5, 112.7, 79.2, 55.4, 35.2, 21.6. HRMS (ESI) m/z 504.1238 ($M+Na^+$), calc. for $C_{29}H_{23}NO_4SNa$ 504.1240.

The ee was determined by HPLC analysis: CHIRALCEL OD−H (4.6 mm i.d. x 250 mm); Hexane/2-propanol = 80/20; flow rate 1.0 mL/min; 30 °C; 210 nm; retention time: 8.1 min (major) and 49.1 min (minor).

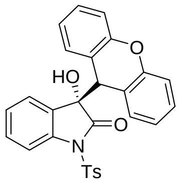

(*S*)-3-hydroxy-1-tosyl-3-(9*H*-xanthen-9-yl)indolin-2-one (2ae):

In PhCl: 73% yield, 80% ee.

White solid, Mp 188.4−186.1 °C, $[\alpha]_D^{22}$ +14.6 (*c* 1.0, $CHCl_3$). ^{1}H NMR (300 MHz, $CDCl_3$) δ 7.88 (d, *J* = 8.3 Hz, 2H), 7.54 (d, *J* = 8.2 Hz, 1H), 7.35 − 7.24 (m, 5H), 7.23 − 7.09 (m, 3H), 7.09 − 6.99 (m, 3H), 6.98 − 6.85 (m, 2H), 6.69 (d, *J* = 6.9 Hz, 1H), 4.46 (s, 1H), 2.41 (s, 3H). ^{13}C NMR (75 MHz, $CDCl_3$) δ 176.1, 153.1, 152.7, 145.7, 138.9, 134.9, 130.7, 130.5, 129.8, 129.7, 129.2, 129.1, 128.0, 126.6, 125.0, 124.7, 123.4, 122.8, 117.9, 117.1, 116.3, 112.6, 78.8, 48.6, 21.7. HRMS (ESI) m/z 506.1032 (M+Na$^+$), calc. for $C_{28}H_{21}NO_5SNa$ 506.1033.

The ee was determined by HPLC analysis: CHIRALCEL OD−H (4.6 mm i.d. x 250 mm); Hexane/2-propanol = 80/20; flow rate 1.0 mL/min; 25 °C; 210 nm; retention time: 6.4 min (major) and 9.6 min (minor).

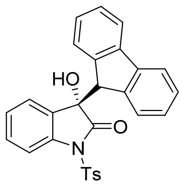

(*S*)-3-(9*H*-fluoren-9-yl)-3-hydroxy-1-tosylindolin-2-one (2af):

In PhCl: 52% yield, 95% ee.

White solid, Mp 171.4−172.1 °C, $[\alpha]_D^{22}$ −125.5 (*c* 1.0, $CHCl_3$). ^{1}H NMR (300 MHz, $CDCl_3$) δ 7.99 (d, *J* = 8.3 Hz, 2H), 7.72 (d, *J* = 7.6 Hz, 1H), 7.69 − 7.61 (m, 2H), 7.52 − 7.40 (m, 2H), 7.33 (dd, *J* = 16.5, 7.8 Hz, 3H), 7.24 − 7.06 (m, 3H), 6.92 (t, *J* = 7.6 Hz, 1H), 6.73 (t, *J* = 7.6 Hz, 1H), 6.15 (d, *J* = 7.3 Hz, 1H), 4.52 (s, 1H), 2.47 (s, 3H). ^{13}C NMR (75 MHz, $CDCl_3$) δ 175.5, 145.9, 142.4, 141.6, 141.2, 139.5, 138.8, 135.0, 130.4, 129.9, 128.4, 128.2, 128.0, 127.0, 127.0, 126.9, 126.0, 124.5, 124.5, 124.2, 119.7, 119.7, 113.0, 78.0, 54.0, 21.8. HRMS (ESI) m/z 490.1081 (M+Na$^+$), calc. for $C_{28}H_{21}NO_4SNa$ 490.1084.

The ee was determined by HPLC analysis: CHIRALCEL OZ−H (4.6 mm i.d. x 250 mm); Hexane/2-propanol = 80/20; flow rate 1.0 mL/min; 30 °C; 210 nm; retention time: 14.5 min (minor) and 18.5 min (major).

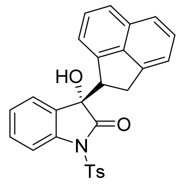

(*S*)-3-((*S*)-1,2-dihydroacenaphthylen-1-yl)-3-hydroxy-1-tosylindolin-2-one (2ag):

In PhCl: 63% yield, 1.4:1 dr.

Major diastereomer: 84% ee.

Light yellow solid, Mp 146.2−147.1 °C, $[\alpha]_D^{22}$ −64.9 (*c* 1.0, $CHCl_3$). ^{1}H NMR (300 MHz, $CDCl_3$) δ 7.87 (d, *J* = 8.2 Hz, 1H), 7.79 (d, *J* = 8.2 Hz, 2H), 7.62 (d, *J* = 8.2 Hz, 1H), 7.52 (d, *J* = 8.2 Hz, 1H), 7.39−7.29 (m, 3H), 7.20 (dd, *J* = 12.0, 7.7 Hz, 3H), 6.99 (d, *J* = 6.8 Hz, 1H), 6.84 (t, *J* = 7.6 Hz, 1H), 6.56 (d, *J* = 7.3 Hz, 1H), 4.31 (dd, *J* = 8.2, 3.3 Hz, 1H), 3.21 (m, 2H), 2.77 (d, *J* = 17.8 Hz, 1H), 2.42 (s, 3H). ^{13}C NMR (75 MHz, $CDCl_3$) δ 175.7, 145.7, 142.6, 141.7, 139.3, 139.3, 134.5, 131.1, 130.5, 129.7, 127.7, 127.5, 126.7, 125.2, 125.1, 124.0, 122.3, 121.5, 119.1, 113.4, 77.6, 50.8, 31.9, 21.7. HRMS (ESI) m/z 478.1082 ($M+Na^+$), calc. for $C_{27}H_{21}NO_4SNa$ 478.1084.

The ee was determined by HPLC analysis: CHIRALPAK ID2010 (4.6 mm i.d. x 250 mm); Hexane/2-propanol = 80/20; flow rate 1.0 mL/min; 30 °C; 210 nm; retention time: 35.4min (minor) and 43.7min (major).

Minor diastereomer: 96% ee.

Light yellow oil, $[\alpha]_D^{22}$ −85.9 (*c* 1.0, $CHCl_3$). ^{1}H NMR (300 MHz, $CDCl_3$) δ 8.05 (d, *J* = 8.2 Hz, 2H), 7.79 (d, *J* = 8.3 Hz, 1H), 7.55 (d, *J* = 8.3 Hz, 2H), 7.43 (dd, *J* = 12.3, 7.6 Hz, 3H), 7.33 (s, 1H), 7.29 (d, *J* = 6.3 Hz, 1H), 7.21 (t, *J* = 7.6 Hz, 2H), 6.98 (d, *J* = 6.8 Hz, 1H), 6.91 (t, *J* = 7.5 Hz, 1H), 4.40 (d, *J* = 7.1 Hz, 1H), 3.71 (d, *J* = 17.3 Hz, 1H), 3.55 (dd, *J* = 18.1, 8.4 Hz, 1H), 2.53 (s, 3H). ^{13}C NMR (75 MHz, $CDCl_3$) δ 175.9, 145.9, 142.7, 141.0, 138.9, 138.8, 134.9, 131.2, 130.3, 129.9, 128.1, 127.7, 127.4, 127.0, 124.8, 124.3, 124.0, 122.6, 119.9, 119.1, 113.1, 77.8, 51.3, 32.6, 21.8.

The ee was determined by HPLC analysis: CHIRALPAK ID2010 (4.6 mm i.d. x 250 mm); Hexane/2-propanol = 80/20; flow rate 1.0 mL/min; 30 °C; 210 nm; retention time: 25.4 min (minor) and 29.8 min (major).

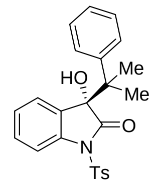

(*S*)-3-hydroxy-3-(2-phenylpropan-2-yl)-1-tosylindolin-2-one (2ah):

In PhCl: 29% yield, 92% ee; in neat: 57% yield, 98% ee.

Light yellow solid, Mp 150.6−151.3 °C, $[\alpha]_D^{22}$ −31.7 (*c* 1.0, $CHCl_3$). ^1H NMR (300 MHz, $CDCl_3$) δ 7.92 (d, *J* = 8.3 Hz, 2H), 7.73 (d, *J* = 8.2 Hz, 1H), 7.36 – 7.13 (m, 9H), 6.96 (t, *J* = 7.6 Hz, 1H), 6.50 (d, *J* = 7.4 Hz, 1H), 2.70 (s, 1H), 2.39 (s, 3H), 1.53 (s, 3H), 1.38 (s, 3H). ^{13}C NMR (75 MHz, $CDCl_3$) δ 176.6, 145.7, 141.8, 139.1, 134.8, 130.1, 129.8, 128.0, 127.6, 127.1, 126.0, 124.1, 112.6, 80.5, 45.2, 22.4, 22.1, 21.7. HRMS (ESI) m/z 444.1238 ($M+Na^+$), calc. for $C_{24}H_{23}NO_4SNa$ 444.1240.

In PhCl: the ee was determined by HPLC analysis: CHIRALPAK IE (4.6 mm i.d. x 250 mm); Hexane/2-propanol = 80/20; flow rate 1.0 mL/min; 30 °C; 210 nm; retention time: 15.2 min (major) and 24.5 min (minor).

In neat: the ee was determined by HPLC analysis: CHIRALPAK IE (4.6 mm i.d. x 250 mm); Hexane/2-propanol = 80/20; flow rate 1.0 mL/min; 30 °C; 210 nm; retention time: 15.1 min (major) and 24.2 min (minor).

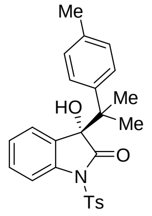

(*S*)-3-hydroxy-3-(2-(p-tolyl)propan-2-yl)-1-tosylindolin-2-one (2ai):

In PhCl: 18% yield, 94% ee; in neat: 19% yield, 76% ee.

Light yellow oil, $[\alpha]_D^{22}$ +34.8 (*c* 1.0, $CHCl_3$). ^1H NMR (300 MHz, $CDCl_3$) δ 7.90 (d, *J* = 8.3 Hz, 2H), 7.76 (d, *J* = 8.2 Hz, 1H), 7.37−7.32 (m, 1H), 7.29 (d, *J* = 8.2 Hz, 2H), 7.20−7.16 (m, 2H), 6.97 (d, *J* = 8.0 Hz, 2H), 6.81 (d, *J* = 8.0 Hz, 2H), 3.11 (q, *J* = 13.3 Hz, 2H), 2.82 (dt, *J* = 13.7, 6.9 Hz, 2H), 2.42 (s, 3H), 1.62 (s, 1H), 1.19 (d, *J* = 6.9 Hz, 6H). ^{13}C NMR (75 MHz, $CDCl_3$) δ 176.3, 147.9, 145.7, 138.5, 134.9, 130.3, 129.8, 129.7, 128.5, 127.9, 126.2, 124.9, 124.7, 113.4, 45.0, 33.6, 23.9, 23.8, 21.7. HRMS (ESI) m/z 458.1393 ($M+Na^+$), calc. for $C_{25}H_{25}NO_4SNa$ 458.1397.

In PhCl: the ee was determined by HPLC analysis: CHIRALPAK ID (4.6 mm i.d. x 250 mm); Hexane/2-propanol = 80/20; flow rate 1.0 mL/min; 30 °C; 210 nm; retention time: 17.7 min (major) and 19.6 min (minor).

In neat: the ee was determined by HPLC analysis: CHIRALPAK ID (4.6 mm i.d. x 250 mm); Hexane/2-propanol = 80/20; flow rate 1.0 mL/min; 30 °C; 210 nm; retention time: 17.9 min (major) and 19.6 min (minor).

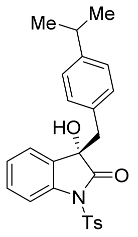

(*S*)-3-hydroxy-3-(4-isopropylbenzyl)-1-tosylindolin-2-one (2aj):

In PhCl: 16% yield, 99% ee; in neat: 16% yield, 87% ee.

Light yellow oil, $[\alpha]_D^{22}$ +17.1 (*c* 1.0, $CHCl_3$). ^{1}H NMR (300 MHz, $CDCl_3$) δ 7.94 (d, *J* = 8.3 Hz, 2H), 7.76 (d, *J* = 8.1 Hz, 1H), 7.30 (t, *J* = 10.1 Hz, 3H), 7.09 (q, *J* = 8.3 Hz, 4H), 6.99 (t, *J* = 7.5 Hz, 1H), 6.58 (d, *J* = 7.7 Hz, 1H), 2.66 (s, 1H), 2.40 (s, 3H), 2.34 (s, 3H), 1.51 (s, 3H), 1.37 (s, 3H). ^{13}C NMR (75 MHz, $CDCl_3$) δ 176.6, 145.7, 139.1, 138.6, 136.8, 134.8, 130.1, 129.8, 128.4, 128.0, 127.9, 127.7, 126.1, 124.1, 112.6, 80.5, 44.8, 22.6, 22.2, 21.7, 21.0. HRMS (ESI) m/z 458.1394 (M+Na$^+$), calc. for $C_{25}H_{25}NO_4SNa$ 458.1397.

In PhCl: the ee was determined by HPLC analysis: CHIRALPAK ID (4.6 mm i.d. x 250 mm); Hexane/2-propanol = 80/20; flow rate 1.0 mL/min; 30 °C; 210 nm; retention time: 17.5 min (minor) and 19.5 min (major).

In neat: the ee was determined by HPLC analysis: CHIRALPAK ID (4.6 mm i.d. x 250 mm); Hexane/2-propanol = 80/20; flow rate 1.0 mL/min; 30 °C; 210 nm; retention time: 17.3 min (minor) and 19.9 min (major).

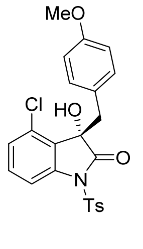

(*S*)-4-chloro-3-hydroxy-3-(4-methoxybenzyl)-1-tosylindolin-2-one (2ak):

In PhCl: 61% yield, 92% ee; in neat: 63% yield, 88% ee.

Light yellow oil, $[\alpha]_D^{22}$ +64.7 (*c* 1.0, $CHCl_3$). ^{1}H NMR (300 MHz, $CDCl_3$) δ 7.80 (d, *J* = 8.3 Hz, 2H), 7.61 (d, *J* = 8.3 Hz, 1H), 7.23 (s, 1H), 7.14 (d, *J* = 8.3 Hz, 1H), 7.05 (s, 1H), 6.73 (d, *J* = 8.6 Hz, 2H), 6.58 (d, *J* = 8.6 Hz, 2H), 3.73 (s, 3H), 3.09 (q, *J* = 13.3 Hz, 2H), 2.41 (s, 3H), 2.33 (s, 3H). ^{13}C NMR (75 MHz, $CDCl_3$) δ 176.3, 158.7, 145.5, 136.2, 134.8, 134.8, 131.2, 130.7, 129.7, 128.4, 127.7 125.2, 124.5, 113.4 113.2, 76.9, 55.1, 44.5, 21.7, 24.0. HRMS (ESI) m/z 480.0638 (M+Na$^+$), calc. for $C_{23}H_{20}ClNO_5SNa$ 480.0643.

In PhCl: the ee was determined by HPLC analysis: CHIRALPAK ID (4.6 mm i.d. x 250 mm); Hexane/2-propanol = 80/20; flow rate 1.0 mL/min; 30 ^{o}C; 210 nm; retention time: 20.9 min (minor) and 27.4 min (major).

In neat: the ee was determined by HPLC analysis: CHIRALPAK ID (4.6 mm i.d. x 250 mm); Hexane/2-propanol = 80/20; flow rate 1.0 mL/min; 30 ^{o}C; 210 nm; retention time: 21.0 min (minor) and 27.4 min (major).

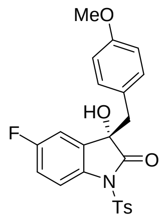

(*S*)-5-fluoro-3-hydroxy-3-(4-methoxybenzyl)-1-tosylindolin-2-one (2al):

In PhCl: 56% yield, 98% ee; in neat: 63% yield, 92% ee.

Light yellow solid, Mp 137.6−138.3 ^{o}C, $[\alpha]^{22}_{D}$ −55.8 (*c* 1.0, $CHCl_3$). ^{1}H NMR (300 MHz, $CDCl_3$) δ 7.81 (d, *J* = 8.2 Hz, 2H), 7.71 (dd, *J* = 8.9, 4.2 Hz, 1H), 7.27 (d, *J* = 8.4 Hz, 2H), 7.03 (td, *J* = 8.9, 2.3 Hz, 1H), 6.94 (dd, *J* = 7.4, 2.4 Hz, 1H), 6.74 (d, *J* = 8.5 Hz, 2H), 6.59 (d, *J* = 8.5 Hz, 2H), 3.73 (s, 3H), 3.09 (q, *J* = 13.4 Hz, 2H), 2.42 (s, 3H); ^{13}C NMR (75 MHz, $CDCl_3$) δ 176.0, 160.1 (d, J_{C-F} = 245.5 Hz), 158.8, 145.8, 134.6, 134.4 (d, J_{C-F} = 2.5 Hz), 131.2, 130.4 (d, J_{C-F} = 8.0 Hz), 129.8, 127.8, 124.0, 116.8 (d, J_{C-F} = 23.3 Hz), 114.8 (d, J_{C-F} = 7.9 Hz), 113.6, 112.4 (d, J_{C-F} = 24.6 Hz) 76.9, 76.9, 55.1, 44.6, 21.7. HRMS (ESI) m/z 464.0930 (M+Na^{+}), calc. for $C_{23}H_{20}FNO_5SNa$ 464.0938.

In PhCl: the ee was determined by HPLC analysis: CHIRALPAK ID (4.6 mm i.d. x 250 mm); Hexane/2-propanol = 80/20; flow rate 1.0 mL/min; 30 ^{o}C; 210 nm; retention time: 15.4 min (minor) and 20.3 min (major).

In neat: the ee was determined by HPLC analysis: CHIRALPAK ID (4.6 mm i.d. x 250 mm); Hexane/2-propanol = 80/20; flow rate 1.0 mL/min; 25 ^{o}C; 210 nm; retention time: 16.9 min (minor) and 22.3 min (major).

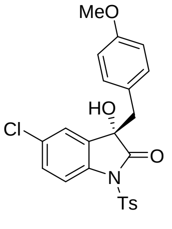

(*S*)-5-chloro-3-hydroxy-3-(4-methoxybenzyl)-1-tosylindolin-2-one (2am):

In PhCl: 54% yield, 94% ee; in neat: 61% yield, 95% ee.

Light yellow solid, Mp 155.6−157.3 ^{o}C, $[\alpha]^{22}_{D}$ +60.6 (*c* 1.0, $CHCl_3$). ^{1}H NMR

(300 MHz, $CDCl_3$) δ 7.82 (d, *J* = 8.3 Hz, 2H), 7.70 (d, *J* = 8.7 Hz, 1H), 7.32 (dd, *J* = 14.7, 5.1 Hz, 3H), 7.24 (d, *J* = 2.0 Hz, 1H), 6.75 (d, *J* = 8.6 Hz, 2H), 6.61 (d, *J* = 8.6 Hz, 2H), 3.76 (s, 3H), 3.17–3.06 (m, 2H), 2.45 (s, 3H). ^{13}C NMR (75 MHz, $CDCl_3$) δ 175.8, 158.7, 145.9, 137.0, 134.4, 131.1, 130.6, 130.3, 130.2, 129.8, 127.8, 125.0, 123.9, 114.6, 113.6, 76.8, 55.1, 44.5, 21.7. HRMS (ESI) m/z 480.0639 (M+Na$^+$), calc. for $C_{23}H_{20}ClNO_5SNa$ 480.0643.

In PhCl: the ee was determined by HPLC analysis: CHIRALPAK ID (4.6 mm i.d. x 250 mm); Hexane/2-propanol = 80/20; flow rate 1.0 mL/min; 30 °C; 210 nm; retention time: 15.1 min (minor) and 19.2 min (major).

In neat: the ee was determined by HPLC analysis: CHIRALPAK ID (4.6 mm i.d. x 250 mm); Hexane/2-propanol = 80/20; flow rate 1.0 mL/min; 30 °C; 210 nm; retention time: 15.0 min (minor) and 19.0 min (major).

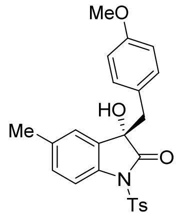

(*S*)-3-hydroxy-3-(4-methoxybenzyl)-5-methyl-1-tosylindolin-2-one (2an): In PhCl: 61% yield, 95% ee; in neat: 81% yield, 93% ee.

Light yellow solid, Mp 146.1−147.9 °C, $[\alpha]_D^{22}$ +33.4 (*c* 1.0, $CHCl_3$). ^{1}H NMR (300 MHz, $CDCl_3$) δ 7.81 (d, *J* = 8.3 Hz, 2H), 7.61 (d, *J* = 8.3 Hz, 1H), 7.25 (d, *J* = 8.4 Hz, 2H), 7.13 (d, *J* = 8.4 Hz, 1H), 7.05 (s, 1H), 6.73 (d, *J* = 8.6 Hz, 2H), 6.59 (d, *J* = 8.6 Hz, 2H), 3.73 (s, 3H), 3.08 (q, *J* = 13.3 Hz, 2H), 2.41 (s, 3H), 2.33 (s, 3H). ^{13}C NMR (75 MHz, $CDCl_3$) δ 176.3, 158.7, 145.5, 136.2, 134.9, 134.8, 131.2, 130.7, 129.7, 128.4, 127.7, 125.2, 124.6, 113.5, 113.2, 76.9, 55.1, 44.6, 21.6, 21.0. HRMS (ESI) m/z 460.1185 (M+Na$^+$), calc. for $C_{24}H_{23}NO_5SNa$ 460.1189.

In PhCl: the ee was determined by HPLC analysis: CHIRALPAK ID (4.6 mm i.d. x 250 mm); Hexane/2-propanol = 80/20; flow rate 1.0 mL/min; 30 °C; 210 nm; retention time: 25.5 min (minor) and 28.0 min (major).

In neat: the ee was determined by HPLC analysis: CHIRALPAK ID (4.6 mm i.d. x 250 mm); Hexane/2-propanol = 80/20; flow rate 1.0 mL/min; 30 °C; 210 nm; retention time: 25.2 min (minor) and 27.7 min (major).

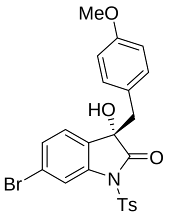

(*S*)-6-bromo-3-hydroxy-3-(4-methoxybenzyl)-1-tosylindolin-2-one (2ao):

In PhCl: 27% yield, 94% ee; in neat: 61% yield, 92% ee.

Light yellow solid, Mp 103.2−104.9°C, $[\alpha]_D^{22}$ +63.7 (*c* 1.0, $CHCl_3$). ^{1}H NMR (300 MHz, $CDCl_3$) δ 7.94 (d, *J* = 1.3 Hz, 1H), 7.81 (d, *J* = 8.3 Hz, 2H), 7.34−7.30 (m, 2H), 7.27 (s, 1H), 7.06 (d, *J* = 8.0 Hz, 1H), 6.71 (d, *J* = 8.6 Hz, 2H), 6.57 (d, *J* = 8.6 Hz, 2H), 3.73 (s, 3H), 3.15−3.01 (m, 3H), 2.44 (s, 3H). ^{13}C NMR (75 MHz, $CDCl_3$) δ 175.8, 158.7, 146.0, 139.5, 134.4, 131.2, 129.8, 128.0, 127.9, 127.4, 126.0, 124.1, 124.0, 116.8, 113.6, 76.6, 55.1, 44.3, 21.7. HRMS (ESI) m/z 524.0136 ($M+Na^+$), calc. for $C_{23}H_{20}BrNO_5SNa$ 524.0138.

In PhCl: the ee was determined by HPLC analysis: CHIRALPAK ID (4.6 mm i.d. x 250 mm); Hexane/2-propanol = 80/20; flow rate 1.0 mL/min; 30 °C; 210 nm; retention time: 16.2 min (minor) and 20.8 min (major).

In neat: the ee was determined by HPLC analysis: CHIRALPAK ID (4.6 mm i.d. x 250 mm); Hexane/2-propanol = 80/20; flow rate 1.0 mL/min; 30 °C; 210 nm; retention time: 16.2 min (minor) and 20.9 min (major).

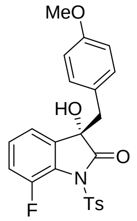

(*S*)-7-fluoro-3-hydroxy-3-(4-methoxybenzyl)-1-tosylindolin-2-one (2ap):

In PhCl: 42% yield, 94% ee; in neat: 43% yield, 89% ee.

Light yellow solid, Mp 135.2−136.9°C, $[\alpha]_D^{22}$ −18.3 (*c* 1.0, $CHCl_3$). ^{1}H NMR (300 MHz, $CDCl_3$) δ 7.85 (d, *J* = 7.9 Hz, 2H), 7.29 (d, *J* = 8.1 Hz, 2H), 7.17−7.11 (m, 1H), 7.03 (dd, *J* = 13.0, 6.0 Hz, 2H), 6.88 (d, *J* = 8.5 Hz, 2H), 6.71 (d, *J* = 8.6 Hz, 2H), 3.75 (s, 3H), 3.25−3.15 (m, 3H), 2.42 (s, 3H). ^{13}C NMR (75 MHz, $CDCl_3$) δ 176.0, 159.0, 150.0, 146.6, 145.4, 135.6, 131.7 (d, J_{C-F}= 1.4 Hz), 131.5, 129.7, 127.90 (d, J_{C-F}= 2.4 Hz), 126.3 (d, J_{C-F}= 7.0 Hz), 125.3 (d, J_{C-F}= 9.9 Hz), 124.2, 120.3 (d, J_{C-F}= 3.5 Hz), 118.6 (d, J_{C-F}= 21.6 Hz), 113.8, 55.2, 45.1, 21.7. HRMS (ESI) m/z 464.0934 ($M+Na^+$), calc. for $C_{23}H_{20}FNO_5SNa$ 464.0938.

In PhCl: the ee was determined by HPLC analysis: CHIRALCEL OZ−H (4.6 mm i.d. x 250 mm); Hexane/2-propanol = 80/20; flow rate 1.0 mL/min; 30 °C; 210 nm; retention time: 18.0 min (minor) and 30.3 min (major).

In neat: the ee was determined by HPLC analysis: CHIRALCEL OZ−H (4.6 mm i.d. x 250 mm); Hexane/2-propanol = 80/20; flow rate 1.0 mL/min; 30 °C; 210 nm; retention time: 17.9 min (minor) and 30.3 min (major).

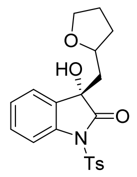

(3*S*)-3-hydroxy-3-((tetrahydrofuran-2-yl)methyl)-1-tosylindolin-2-one (3):

In PhCl: 21% yield, 3.0:1 dr.

Major diastereomer: 4% ee(cannot be separated).

Minor diastereomer: 0% ee (cannot be separated).

Light yellow oil. ^{1}H NMR (300 MHz, $CDCl_3$) δ 8.00–7.90 (m, 3H), 7.36 (dt, *J* = 13.7, 7.8 Hz, 4H), 7.18 (dd, *J* = 13.9, 6.6 Hz, 1H), 5.48 (d, *J* = 3.8 Hz, 0.2H), 5.35 (d, *J* = 4.3 Hz, 0.2H), 5.14 (s, 0.8H), 5.07 (s, 0.8H), 4.06 (dd, *J* = 13.1, 7.8 Hz, 0.2H), 3.85 (dd, *J* = 13.8, 7.7 Hz, 0.2H), 3.60–3.53 (m, 0.8H), 3.39 (dd, *J* = 14.8, 7.1 Hz, 0.8H), 2.41 (s, 3H), 1.97 (dt, *J* = 14.0, 6.5 Hz, 2H), 1.89–1.69 (m, 2H). ^{13}C NMR (75 MHz, $CDCl_3$) δ 173.2, 145.6, 145.5, 139.9, 135.0, 130.4, 130.1, 129.8, 129.6, 128.1, 127.9, 125.8, 125.1, 124.8, 124.7, 123.4, 113.8, 113.6, 103.6, 101.4, 71.4, 71.3, 67.8, 7.47, 32.5, 32.2, 23.1, 2.8, 21.7. HRMS (ESI) m/z 396.0873 (M+Na^{+}), calc. for $C_{19}H_{19}NO_5S$ Na 396.0876.

The ee was determined by HPLC analysis: CHIRALPAK IC (4.6 mm i.d. x 250 mm); Hexane/2-propanol = 80/20; flow rate 1.5 mL/min; 30 °C; 210 nm; retention time: major diastereomer: 22.3 min (minor) and 24.8 min (major); minor diastereomer: 20.3 min (major) and 26.9 min (minor).

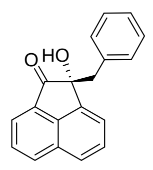

(*R*)-2-benzyl-2-hydroxyacenaphthylen-1(2*H*)-one (7a):

In neat: 51% yield, 96% ee.

Light yellow solid, Mp 147.2−149.2°C, $[\alpha]_D^{22}$ +43.2 (*c* 1.0, $CHCl_3$). ^{1}H NMR (300 MHz, $CDCl_3$) δ 8.05 (d, *J* = 8.1 Hz, 1H), 7.86 (dd, *J* = 12.2, 7.8 Hz, 2H), 7.66 (t, *J* = 7.6

Hz, 1H), 7.60 (t, J = 7.7 Hz, 1H), 7.29 (d, J = 6.9 Hz, 1H), 7.10 (d, J = 3.1 Hz, 3H), 7.00 (d, J = 3.4 Hz, 2H), 3.42−3.12 (m, 3H). ^{13}C NMR (75 MHz, $CDCl_3$) δ 205.2, 141.4, 138.6, 134.6, 131.9, 130.5, 130.4, 128.4, 128.2, 127.8, 126.8, 125.3, 122.0, 121.3, 80.4, 44.3. HRMS (ESI) m/z 297.0883 ($M+Na^+$), calc. for $C_{19}H_{14}O_2Na$ 297.0886.

The ee was determined by HPLC analysis: CHIRALPAK ID (4.6 mm i.d. x 250 mm); Hexane/2-propanol = 80/20; flow rate 1.0 mL/min; 30 °C; 210 nm; retention time: 8.5 min (major) and 9.8 min (minor).

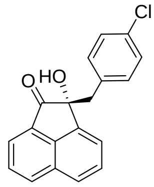

(*R*)-2-(4-chlorobenzyl)-2-hydroxyacenaphthylen-1(2*H*)-one (7b):

In tert-butylbenzene: 40% yield, 88% ee.

Light yellow solid, Mp 172.5−173.3°C, $[\alpha]_D^{22}$ +11.1 (c 1.0, $CHCl_3$). ^{1}H NMR (300 MHz, $CDCl_3$) δ 8.08 (d, J = 8.1 Hz, 1H), 7.88 (dd, J = 11.0, 7.7 Hz, 2H), 7.71−7.67 (m, 1H), 7.63−7.58 (m, 1H), 7.29 (d, J = 6.9 Hz, 1H), 7.07 (d, J = 8.4 Hz, 2H), 6.94 (d, J = 8.4 Hz, 2H), 3.23 (dd, J = 77.5, 13.4 Hz, 2H), 2.52 (s, 1H). ^{13}C NMR (75 MHz, $CDCl_3$) δ 204.98, 141.36, 138.23, 133.17, 132.76, 132.16, 131.77, 130.49, 130.34, 128.45, 128.34, 127.94, 125.58, 122.25, 121.24, 80.25, 43.56. HRMS (ESI) m/z 331.0491 ($M+Na^+$), calc. for $C_{19}H_{13}ClO_2Na$ 331.0496.

The ee was determined by HPLC analysis: CHIRALPAK ID (4.6 mm i.d. x 250 mm); Hexane/2-propanol = 80/20; flow rate 1.0 mL/min; 30 °C; 210 nm; retention time: 7.3 min (major) and 8.4 min (minor).

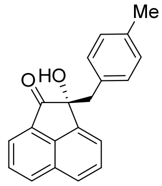

(*R*)-2-hydroxy-2-(4-methylbenzyl)acenaphthylen-1(2*H*)-one (7c):

In tert-butylbenzene: 42% yield, 85% ee.

Light yellow solid, Mp 114.6−115.8°C, $[\alpha]_D^{22}$ +13.9 (c 1.0, $CHCl_3$). ^{1}H NMR (300 MHz, $CDCl_3$) δ 8.06 (d, J = 8.1 Hz, 1H), 7.90 (d, J = 7.0 Hz, 1H), 7.85 (d, J = 8.4 Hz, 1H), 7.68 (t, J = 7.6 Hz, 1H), 7.61 (t, J = 7.6 Hz, 1H), 7.32 (d, J = 6.9 Hz, 1H), 6.92 (s, 4H), 3.23 (dd, J = 71.3, 13.4 Hz, 2H), 2.23 (s, 3H). ^{13}C NMR (75 MHz, $CDCl_3$) δ 205.18, 141.38, 138.79, 136.38, 131.92, 131.41, 130.58, 130.48, 130.39, 128.55, 128.43, 128.17, 125.31,

122.05, 121.26, 80.32, 43.88, 20.97. HRMS (ESI) m/z 271.1113 ($M+H^{+}-H_2O$), calc. for $C_{20}H_{15}O_1$ 271.1117.

The ee was determined by HPLC analysis: CHIRALPAK ID (4.6 mm i.d. x 250 mm); Hexane/2-propanol = 80/20; flow rate 1.0 mL/min; 30 °C; 210 nm; retention time: 8.9 min (major) and 10.9 min (minor).

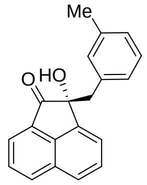

(*R*)-2-hydroxy-2-(3-methylbenzyl)acenaphthylen-1(2*H*)-one (7d):

In neat: 85% yield, 83% ee.

Light yellow solid, Mp 87.6−89.1°C, 85% yield, 83% ee. $[\alpha]_D^{22}$ +22.5 (*c* 1.0, $CHCl_3$); ^{1}H NMR (300 MHz, $CDCl_3$) δ 8.07 (d, *J* = 8.1 Hz, 1H), 7.91−7.83 (m, 1H), 7.68 (t, *J* = 7.6 Hz, 1H), 7.61 (t, *J* = 7.7 Hz, 1H), 7.30 (d, *J* = 6.9 Hz, 1H), 7.01 (t, *J* = 7.4 Hz, 1H), 6.93 (d, *J* = 7.4 Hz, 1H), 6.85 (s, 1H), 6.80 (d, *J* = 7.4 Hz, 1H), 3.34 (d, *J* = 13.3 Hz, 1H), 3.10 (d, *J* = 13.3 Hz, 1H), 2.94 (s, 1H), 2.20 (s, 3H). ^{13}C NMR (75 MHz, $CDCl_3$) δ 205.1, 141.3, 138.7, 137.3, 134.4, 131.9, 131.3, 130.5, 130.4, 128.4, 128.2, 127.7, 127.6, 127.5, 125.3, 122.0, 121.3, 80.3, 44.3, 21.2. HRMS (ESI) m/z 271.1113 ($M+H^{+}-H_2O$), calc. for $C_{20}H_{15}O_1$ 271.1117.

The ee was determined by HPLC analysis: CHIRALPAK ID (4.6 mm i.d. x 250 mm); Hexane/2-propanol = 80/20; flow rate 1.0 mL/min; 30 °C; 210 nm; retention time: 7.8 min (major) and 8.8 min (minor).

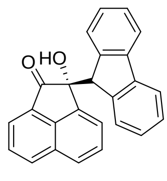

(*R*)-2-(9*H*-fluoren-9-yl)-2-hydroxyacenaphthylen-1(2*H*)-one (7e):

In PhCl: 76% yield, 70% ee.

Light yellow solid, Mp 171.6−172.3°C, $[\alpha]_D^{22}$ +57.2 (*c* 1.0, $CHCl_3$). ^{1}H NMR (300 MHz, $CDCl_3$) δ 7.96 (dd, *J* = 16.0, 7.6 Hz, 3H), 7.70−7.59 (m, 3H), 7.47 (dd, *J* = 11.9, 7.4 Hz, 2H), 7.35 (t, *J* = 7.4 Hz, 1H), 7.17 (dd, *J* = 16.0, 7.7 Hz, 2H), 7.02 (t, *J* = 7.5 Hz, 1H), 6.80 (t, *J* = 7.5 Hz, 1H), 6.36 (d, *J* = 7.0 Hz, 1H), 4.78 (s, 1H), 3.18 (s, 1H). ^{13}C NMR (75 MHz, $CDCl_3$) δ 205.1, 143.2, 142.2, 141.5, 140.4, 136.2, 132.0, 131.0, 130.0, 128.2, 128.0,

127.4, 127.2, 126.8, 126.3, 125.4, 124.6, 121.8, 120.9, 119.6, 119.5, 81.7, 53.8. HRMS (ESI) m/z 371.1041 (M+Na$^+$), calc. for $C_{25}H_{16}O_2Na$ 371.1043.

The ee was determined by HPLC analysis: CHIRALPAK ID (4.6 mm i.d. x 250 mm); Hexane/2-propanol = 80/20; flow rate 1.0 mL/min; 30 °C; 210 nm; retention time: 11.1 min (minor) and 12.3 min (major).

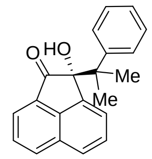

(*R*)-2-hydroxy-2-(2-phenylpropan-2-yl)acenaphthylen-1(2*H*)-one (7f):

In neat: 76% yield, 67% ee.

Light yellow solid, Mp 95.6−96.7°C, $[\alpha]_D^{22}$ −15.9 (*c* 1.0, $CHCl_3$). ^{1}H NMR (300 MHz, $CDCl_3$) δ 8.03 (d, *J* = 8.1 Hz, 1H), 7.81 (d, *J* = 7.5 Hz, 2H), 7.65 (t, *J* = 7.6 Hz, 1H), 7.49 (t, *J* = 7.7 Hz, 1H), 7.27 (s, 2H), 7.20−7.18 (m, 3H), 6.79 (d, *J* = 7.0 Hz, 1H), 3.03 (s, 1H), 1.62 (s, 3H), 1.33 (s, 3H). ^{13}C NMR (75 MHz, $CDCl_3$) δ 205.7, 143.7, 142.0, 138.1, 131.8, 131.5, 130.0, 128.1, 127.9, 127.8, 127.4, 126.6, 125.1, 122.3, 121.2, 8.25, 44.8, 23.6, 23.3. HRMS (ESI) m/z 325.1196 (M+Na$^+$), calc. for $C_{21}H_{18}O_2Na$ 325.1199.

The ee was determined by HPLC analysis: CHIRALPAK IE (4.6 mm i.d. x 250 mm); Hexane/2-propanol = 80/20; flow rate 1.0 mL/min; 30 °C; 210 nm; retention time: 7.6 min (major) and 10.6 min (minor).

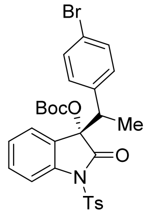

(*S*)-3-((*R*)-1-(4-bromophenyl)ethyl)-2-oxo-1-tosylindolin-3-yl tert-butyl carbonate (8):

>99% yield, 97% ee; white solid, Mp 155.2−156.3 °C, $[\alpha]_D^{22}$ +27.7 (*c* 0.5, $CHCl_3$). ^{1}H NMR (300 MHz, $CDCl_3$) δ 7.77 (dd, *J* = 8.2, 4.6 Hz, 3H), 7.38 (t, *J* = 7.5 Hz, 1H), 7.18 (td, *J* = 15.3, 8.7 Hz, 3H), 7.08 (d, *J* = 8.3 Hz, 2H), 6.51 (d, *J* = 8.4 Hz, 2H), 3.32 (q, *J* = 7.0 Hz, 1H), 2.41 (s, 3H), 1.41−1.46 (m, 4H), 1.21 (s, 8H). ^{13}C NMR (75 MHz, $CDCl_3$) δ 172.6, 150.7, 145.2, 140.0, 136.2, 134.6, 130.9, 130.5, 130.4, 129.3, 128.2, 125.2, 124.3, 124.0, 121.6, 113.4, 83.9, 81.9, 45.7, 27.3, 21.7, 14.7. HRMS (ESI) m/z 608.0704 (M+Na$^+$), calc. for $C_{28}H_{28}BrNO_6SNa$ 608.0713.

The ee was determined by HPLC analysis: CHIRALPAK ID (4.6 mm i.d. x 250 mm); Hexane/2-propanol = 80/20; flow rate 1.0 mL/min; 30 ^{o}C; 210 nm; retention time: 7.3 min (minor) and 9.2 min (major).

Supplementary References

1. Gaussian 16, Revision B.01, Frisch, M. J.; Trucks, G. W.; Schlegel, H. B.; Scuseria, G. E.; Robb, M. A.; Cheeseman, J. R.; Scalmani, G.; Barone, V.; Petersson, G. A.; Nakatsuji, H.; Li, X.; Caricato, M.; Marenich, A. V.; Bloino, J.; Janesko, B. G.; Gomperts, R.; Mennucci, B.; Hratchian, H. P.; Ortiz, J. V.; Izmaylov, A. F.; Sonnenberg, J. L.; Williams-Young, D.; Ding, F.; Lipparini, F.; Egidi, F.; Goings, J.; Peng, B.; Petrone, A.; Henderson, T.; Ranasinghe, D.; Zakrzewski, V. G.; Gao, J.; Rega, N.; Zheng, G.; Liang, W.; Hada, M.; Ehara, M.; Toyota, K.; Fukuda, R.; Hasegawa, J.; Ishida, M.; Nakajima, T.; Honda, Y.; Kitao, O.; Nakai, H.; Vreven, T.; Throssell, K.; Montgomery, J. A., Jr.; Peralta, J. E.; Ogliaro, F.; Bearpark, M. J.; Heyd, J. J.; Brothers, E. N.; Kudin, K. N.; Staroverov, V. N.; Keith, T. A.; Kobayashi, R.; Normand, J.; Raghavachari, K.; Rendell, A. P.; Burant, J. C.; Iyengar, S. S.; Tomasi, J.; Cossi, M.; Millam, J. M.; Klene, M.; Adamo, C.; Cammi, R.; Ochterski, J. W.; Martin, R. L.; Morokuma, K.; Farkas, O.; Foresman, J. B.; Fox, D. J. Gaussian, Inc., Wallingford CT, 2016.
2. Lee, C.; Yang, W.; Parr, R. G., Development of the Colle-Salvetti correlation-energy formula into a functional of the electron density. *Phys. Rev. B* **37**, 785 (1988).
3. Becke, A. D., Density‐functional thermochemistry. III. The role of exact exchange. *J. Chem. Phys.* **98**, 5648 (1993).
4. Ditchfield, R.; Hehre, W. J.; Pople, J. A., Self‐Consistent Molecular‐Orbital Methods. IX. An Extended Gaussian‐Type Basis for Molecular‐Orbital Studies of Organic Molecules. *J. Chem. Phys.* **54**, 724 (1971).
5. Peverati, R.; Truhlar, D. G., Improving the Accuracy of Hybrid Meta-GGA Density Functionals by Range Separation. *J. Phys. Chem. Lett.* **2**, 2810 (2011).
6. Weigend, F.; Ahlrichs, R., Balanced basis sets of split valence, triple zeta valence and quadruple zeta valence quality for H to Rn: Design and assessment of accuracy. *Phys. Chem. Chem. Phys.* **7**, 3297 (2005).
7. Weigend, F., Accurate Coulomb-fitting basis sets for H to Rn. *Phys. Chem. Chem. Phys.* **8**,

1057 (2006).

8. Frisch, M. J.; Trucks, G. W.; Schlegel, H. B.; Scuseria, G. E.; Robb, M. A.; Cheeseman, J. R.; Scalmani, G.; Barone, V.; Mennucci, B.; Petersson, G. A.; Nakatsuji, H.; Caricato, M.; Li, X.; Hratchian, H. P.; Izmaylov, A. F.; Bloino, J.; Zheng, G.; Sonnenberg, J. L.; Hada, M.; Ehara, M.; Toyota, K.; Fukuda, R.; Hasegawa, J.; Ishida, M.; Nakajima, T.; Honda, Y.; Kitao, O.; Nakai, H.; Vreven, T.; Montgomery, J. A., Jr.; Peralta, J. E.; Ogliaro, F.; Bearpark, M.; Heyd, J. J.; Brothers, E.; Kudin, K. N.; Staroverov, V. N.; Kobayashi, R.; Normand, J.; Raghavachari, K.; Rendell, A.; Burant, J. C.; Iyengar, S. S.; Tomasi, J.; Cossi, M.; Rega, N.; Millam, N. J.; Klene, M.; Knox, J. E.; Cross, J. B.; Bakken, V.; Adamo, C.; Jaramillo, J.; Gomperts, R.; Stratmann, R. E.; Yazyev, O.; Austin, A. J.; Cammi, R.; Pomelli, C.; Ochterski, J. W.; Martin, R. L.; Morokuma, K.; Zakrzewski, V. G.; Voth, G. A.; Salvador, P.; Dannenberg, J. J.; Dapprich, S.; Daniels, A. D.; Farkas, Ö.; Foresman, J. B.; Ortiz, J. V.; Cioslowski, J.; Fox, D. J. Gaussian 09, Revision D.01; Gaussian, Inc.: Wallingford, CT, 2009.
